# Human Milk Oligosaccharides in the Milk of Mothers Delivering Term versus Preterm Infants

**DOI:** 10.3390/nu11061282

**Published:** 2019-06-05

**Authors:** Sean Austin, Carlos A. De Castro, Norbert Sprenger, Aristea Binia, Michael Affolter, Clara L. Garcia-Rodenas, Lydie Beauport, Jean-François Tolsa, Céline J. Fischer Fumeaux

**Affiliations:** 1 Nestlé Research, Vers-Chez-Les-Blanc, 1000 Lausanne, Switzerland; norbert.sprenger@rdls.nestle.com (N.S.); Aristea.Binia@rdls.nestle.com (A.B.); michael.affolter@rdls.nestle.com (M.A.); Clara.Garcia@rdls.nestle.com (C.L.G.-R.); 2 Nestle Research Singapore, 29 Quality Road, 618802 Singapore, Singapore; CarlosAntonio.DeCastro@rdsg.nestle.com; 3 Centre Hospitalier Universitaire Vaudois, 1011 Lausanne, Switzerland; Lydie.Beauport@chuv.ch (L.B.); Jean-Francois.Tolsa@chuv.ch (J.-F.T.); Celine-Julie.Fischer@chuv.ch (C.J.F.F.)

**Keywords:** 2′-fucosyllactose (2′FL), 3′-sialyllactose (3′SL), disialyllacto-N-tetraose (DSLNT), human milk oligosaccharides (HMO), milk group, secretor, Lewis, lactation, preterm

## Abstract

Human milk oligosaccharides (HMOs) are a major component of human milk, and play an important role in protecting the infant from infections. Preterm infants are particularly vulnerable, but have improved outcomes if fed with human milk. This study aimed to determine if the HMO composition of preterm milk differed from that of term milk at equivalent stage of lactation and equivalent postmenstrual age. In all, 22 HMOs were analyzed in 500 samples of milk from 25 mothers breastfeeding very preterm infants (< 32 weeks of gestational age, < 1500 g of birthweight) and 28 mothers breastfeeding term infants. The concentrations of most HMOs were comparable at equivalent postpartum age. However, HMOs containing α-1,2-linked fucose were reduced in concentration in preterm milk during the first month of lactation. The concentrations of a number of sialylated oligosaccharides were also different in preterm milk, in particular 3′-sialyllactose concentrations were elevated. At equivalent postmenstrual age, the concentrations of a number of HMOs were significantly different in preterm compared to term milk. The largest differences manifest around 40 weeks of postmenstrual age, when the milk of term infants contains the highest concentrations of HMOs. The observed differences warrant further investigation in view of their potential clinical impact.

## 1. Introduction

Human milk is the optimal source of nutrition for infants, and it is widely recommended that infants are exclusively or predominantly breastfed for the first 6 months of life [1,2]. In preterm neonates, human milk feeding is known to have several important specific protective actions and it is strongly encouraged too [1,3,4]. Preterm infants consuming human milk have notably improved immunity [5], are less likely to develop necrotizing enterocolitis (NEC) [6], have improved neurodevelopmental outcomes [1,7] and a better long term health outcome [8,9].

However, as nutritional needs of preterm infants are higher compared to those of term infants, human milk composition may not match the nutritional needs of very preterm infants (< 32 weeks of gestational age, < 1500g of birthweight) during the first weeks of life. In this context, human milk fortification in energy, proteins and minerals is commonly recommended in routine nutritional neonatal care of very preterm infants [4]. To optimize current practices, additional knowledge on human milk composition and opportunities in nutrient supplementation remain to be further explored.

While the composition of preterm milk has been investigated [10], and it is reported to be slightly different from that of term milk [11,12], there are relatively few studies focusing on the human milk oligosaccharide (HMO) composition of preterm milk.

HMOs are the 4th most abundant component of human milk after water, lipids and lactose, and may be present at concentrations up to 25 g/L in colostrum and between 10 to 15 g/L in mature milk [13,14,15]. This family of over 160 compounds [16] is postulated to play an important role in protecting infants from infection, by acting as decoy receptors or through modulation of the gut microbiota. They may also modulate the immune system through direct interactions [17,18,19,20]. Furthermore, HMOs may act as a dietary source of sialic acid [21,22], potentially important for learning and memory [23,24]. Recent evidence from animal studies suggests non-sialylated HMOs may also be important for learning and memory [25]. Today, randomized placebo controlled trials with HMO supplementation are scarce and only done with individual HMOs. Trials that have been carried out indicate that 2′FL is associated with infant immunity [26] and 2′FL together with lacto-N-neotetraose (LNnT) relate to protection from illnesses of the lower respiratory tract and a reduction in antibiotic use [27].

Of specific relevance to preterm infants, HMOs have been linked to the prevention of gut dysfunction [28] and development of NEC [28,29]. Disialyllacto-N-tetraose (DSLNT) and 2′FL have been shown to protect against the development of NEC in rat models [30,31]. It was also observed that DSLNT was present in lower concentrations in the milk fed to preterm infants who developed NEC compared to those who did not [32], leading to the proposal that DSLNT could be used as a marker to predict the likelihood of an infant developing NEC [32].

HMOs are built from lactose, the lactose can be elongated with residues of galactose and N-acetylglucosamine to produce at least 13 different core oligosaccharides [16,33,34]. The core oligosaccharides can be further decorated with sialic acid residues by the action of sialylltransferases, and fucose residues by the action of fucosyltransferases. Due to genetic polymorphisms, two fucosyltransferases, fucosyltransferase-2 (FUT2) and fucosyltransferase-3 (FUT3) are not active in 100% of the population. Fucosyltransferase-2 (FUT2) is responsible for the attachment of fucose to core oligosaccharides through an α-1,2-linkage creating HMOs such as 2′-fucosyllactose (2′FL) or lacto-N-fucopentaose-I (LNFP-I). When FUT2 is inactive such HMOs are absent from the milk. Fucosyltransferase-3 (FUT3), attaches fucose to the core oligosaccharides through an α-1,4 linkage creating oligosaccharides such as LNFP-II, and along with other fucosyltransferases HMOs containing α-1,3 linkages. When FUT3 is inactive HMOs containing α-1,4 linkages are absent from the milk. This results in there being four major milk groups with differing HMO compositions depending on the activities of the FUT2 and FUT3 enzymes:

- Milk group 1, in which both enzymes are active
- Milk group 2, in which FUT3 is active but FUT2 is not
- Milk group 3, in which FUT2 is active but FUT3 is not
- Milk group 4, in which both enzymes are inactive.

In general, milk group 1 seems to be the most common milk group, and milk group 4 is rare [35], however the distribution of milk groups in different populations varies depending on genetic background [36]. Based on the HMO composition of different milk samples, there is also evidence that further subgroups may exist [37,38]. 

There have been a few previous investigations looking at the HMO composition of preterm milk [17,32,36,39,40,41,42,43], in most cases it is reported that the HMO composition of preterm milk is comparable to that of term milk. However, Coppa [39] reported that preterm milk contained a higher total HMO concentration compared to term milk and De Leoz [36] reported that some HMO features were more variable in preterm milk than in term milk. Kunz [43] pointed out that the Lewis and secretor status should be taken in to account when making studies on HMOs, since these are important factors determining the HMO composition, but milk group has been considered in only a few studies [40,41,42].

Here we report the composition and trajectories of several different HMOs analyzed in milk collected from mothers of preterm infants and term infants. The HMO composition was compared at equivalent stage of lactation (i.e., equivalent infant postnatal age) and at equivalent postmenstrual age (i.e., equivalent infant developmental stage). In addition the milk group for each donor could be identified from the HMO composition of the milk, thus comparisons could be performed on samples with matched milk groups.

## 2. Materials and Methods

### 2.1. Trial Design

This research is part of a prospective cohort study to compare the nutritional composition of human milk from mothers giving birth at term or preterm. The detailed study design has already been published [44].

The study was conducted at the Neonatal Intensive Care Unit (NICU) and at the maternity ward of the University Hospital in Lausanne (CHUV), Switzerland, between October 2013 and July 2014. In this cohort, human milk was longitudinally collected from mothers of preterm infants (gestational age 28 0/7 weeks to 32 6/7 weeks) and mothers of term infants (gestational age 37 0/7 weeks to 41 6/7 weeks). A dedicated research nurse, qualified as a lactation consultant, followed and supported the subjects during the study period. Neonatal demographic and delivery data were collected from the medical charts upon enrollment.

### 2.2. Milk Collection & Storage

**Figure 1:**
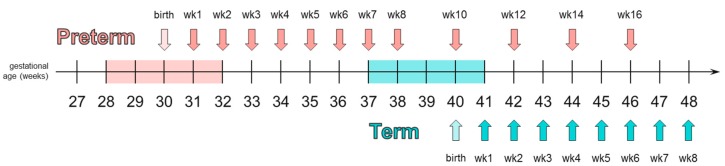
Example of milk sampling scheme from term and preterm mothers delivering at postmenstrual weeks 40 and 30 respectively. Reprinted from Garcia-Rodenas, et al. Clinical Nutrition, 2018 [44] with kind permission from Elsevier.

Preterm human milk samples were collected once per week at 7 ± 1 day intervals during the first 8 weeks after delivery. An additional 4 samples were collected until 16 weeks after delivery with sample collection at 14 ± 1 day intervals. Term human milk samples were collected once per week at 7 ± 1 day intervals during the first 8 weeks after delivery (Figure 1). With such a sampling design, it was possible to compare preterm milk with term milk at the same stage of lactation (in Figure 1 the preterm sample at wk1 with the term sample at wk1, etc) or at the equivalent postmenstrual age of the infant (in Figure 1 samples at 42, 44 and 46 weeks would be compared ie. preterm wk12 with term wk2, preterm wk14 with term wk4, etc.)

Samples were collected at the first milk expression in the morning between 06:00 and 12:00 using an electric, double breast pump (Symphony^®^, Medela, 6340 Baar Switzerland). Full milk expression from a single breast was collected, homogenized, and a 10 mL sample was taken for analysis (except for the first 2 visits, when the volume of sample for analysis was 1–3 mL). Milk for analysis was transferred to a 15 mL polypropylene tube and stored at −18 °C for up to 1 week until hospital transfer and then at −80 °C. Samples were thawed once, homogenized, and split in to 15 different aliquots for different analyses. Those aliquots were then stored at −80 °C until analysis or shipment. Aliquots for HMO analysis were shipped to Neotron S.p.A, Modena, Italy on dry ice. 

### 2.3. Ethical & Legal Considerations

The study was conducted according to the guidelines in the Declaration of Helsinki. The study was approved by the local ethics committee (Commission cantonale d’éthique de la recherche sur l’être humain du Canton de Vaud, Switzerland; Protocol 69/13, clinical study 11.39.NRC). Written informed consent was obtained from all subjects participating in the study. The study was registered on ClinicalTrials.gov with the identifier NCT02052245.

### 2.4. Analytical Method

HMOs were analyzed by liquid chromatography with fluorescence detection after labelling with 2-aminobenzamide using the protocol described by Austin & Benet [45]. Ten HMOs were quantified against genuine standards with known purity; 2′-fucosyllactose (2′FL), 3-fucosyllactose (3FL), A-tetrasaccharide (A-Tetra), Lacto-N-tetraose (LNT), Lacto-N-neotetraose (LNnT), 3′-sialyllactose (3′SL), 6′-sialyllactose (6′SL), Lacto-N-fucopentaose-I (LNFP-I), LNFP-V and Lacto-N-neofucopentaose (LNnFP). All other HMOs were quantified against maltotriose with known purity, assuming equimolar response factors (graphical representations of the HMOs studied can be found in supplementary Figure S1). A pooled human milk sample (Lee Biosolutions, St Louis, USA) was analyzed with every batch of analysis and at least every 25 injections as a quality control (QC) sample to ensure the method was performing consistently between analytical batches. 

### 2.5. Assignment to FUT2 and FUT3-Dependant Milk Group

The milk samples were assigned to one of 4 milk groups depending on the levels of 2′FL (a marker for FUT2 activity) and LNFP-II (a marker for FUT3 activity) in the sample at visit 2 (2 weeks postpartum). Samples in milk group 1 have high levels (> 25 mg/L) of 2′FL and (>35 mg/L) LNFP-II, samples in milk group 2 have high levels (> 35 mg/L) of LNFP-II and low levels (< 25 mg/L) of 2′FL, samples in milk group 3 have high levels (> 25 mg/L) of 2′FL and low levels (< 35mg/L) of LNFP-II, samples in milk group 4 have low levels (< 25mg/L) of 2′FL and (< 35 mg/L) LNFP-II.

### 2.6. Data Analysis

All statistical analyses were done with the statistical software R 3.2.3. Prior to statistical analysis all results below the method limit of quantification (LoQ) were set to a value of 0.5 × LoQ. A mixed linear model was used in comparing the two groups (preterm and term) in which the group, infant age (postpartum or postmenstrual) and mode of delivery were considered as fixed effects. The “within subject” variability is taken into account by declaring the subjects as random effects. The main point of comparison is preterm vs term infants. An adjustment for mode of delivery was made because it is a confounding effect with term status given that there are higher proportion of preterm infants delivered by C-section. A logarithmic transformation was applied to some of the HMO concentrations when modelling as the distribution was skewed and according to Box-Cox and QQ plots a log-transformation seemed adequate. Contrast estimates of the model were used to assess significant differences between preterm and term infants at specified lactation and postmenstrual weeks.

## 3. Results

### 3.1. Subject Characteristics

This study included 27 mothers with a total of 33 preterm infants, and 34 mothers with a total of 34 term infants. Two of the 27 mothers with preterm infants and six of the 34 mothers with term infants dropped out of the study before completion. No serious adverse events were reported during the study. A total of 500 milk samples were collected and analyzed for HMO content; 280 preterm samples and 220 term samples. The study flow chart and detailed demographic and anthropometric data have already been reported [44]. In summary, baseline maternal characteristics were not different between the two groups, and all mothers in both groups were healthy. Delivery by cesarean section was more common in the preterm group, as were multiple deliveries (twins). Unsurprisingly, term and preterm infants differed in gestational age, birth weight, height and head circumference. There was no difference in gender distribution.

### 3.2. Milk Groups

**Figure 2:**
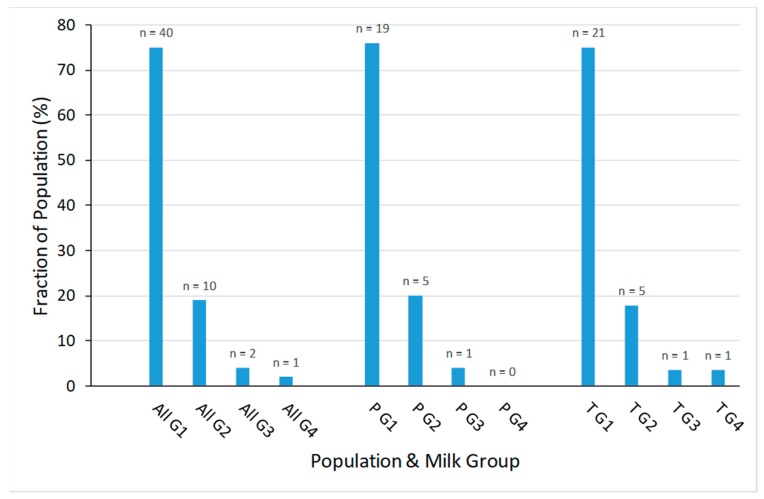
Distribution of milk groups in the different populations: All = all participants, P = participants with preterm infants, T = participants with term infants, G1 = milk group 1, G2 = milk group 2, G3 = milk group 3, G4 = milk group 4.

The milk samples were assigned to one of 4 milk groups. Overall, 75% of samples were assigned to milk group 1, 19% to milk group 2, 4% to milk group 3 and 2% (1 individual) to milk group 4 (Figure 2). Milk group distribution was similar in the preterm and term populations. 

### 3.3. Changes in HMO Concentration During Lactation

**Figure 3:**
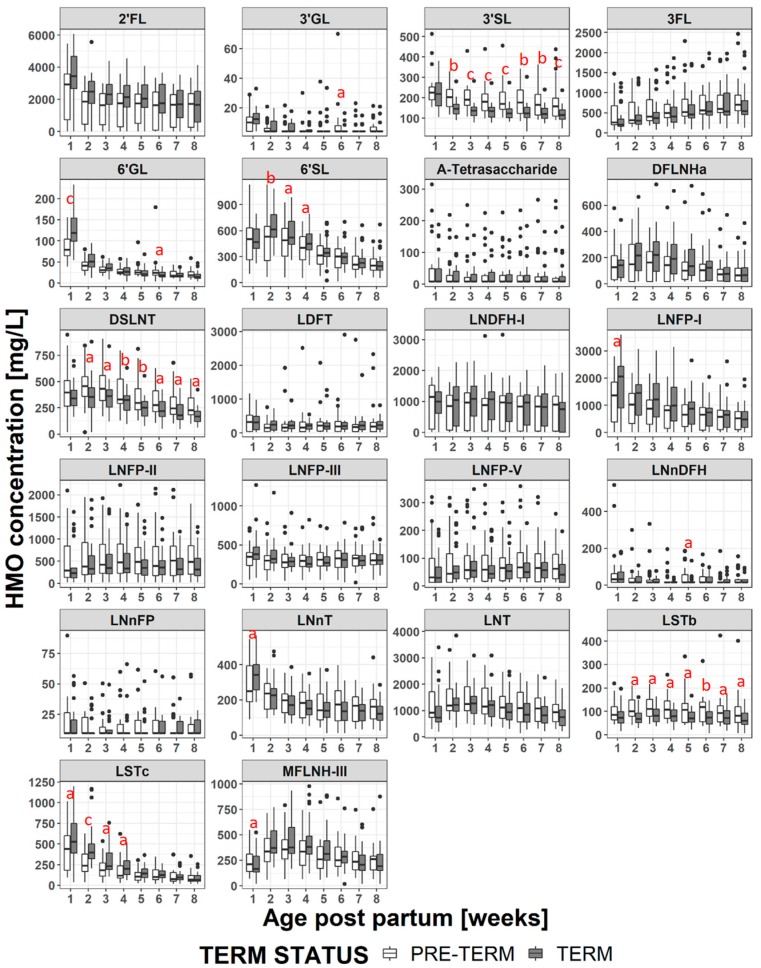
Mean concentration of each HMO at each visit for term (grey) and preterm (white) infants, letters indicate if difference between term and preterm is significant; a: *p* < 0.05, b: *p* < 0.005, c: *p* < 0.0005.

For both term and preterm milk, the concentration of many of the HMOs decreased at later stages of lactation (Figure 3, and Supplementary Table S1), of those LNT, DSLNT, LNDFH-I, MFLNH-III and DFLNHa reached their maximum concentration after the first week postpartum (i.e., at weeks 2 or 3 postpartum). The only HMO to increase in concentration at later stages of lactation was 3FL.

### 3.4. HMO Concentration in Term versus Preterm Milk at Equivalent Postpartum Age (Lactation Stages) 

Considering all samples, when the children were the same age postpartum, the concentration of each HMO in the mother’s milk was generally comparable between term and preterm groups (Figure 3 and Supplementary Table S1). The main exceptions to this were 3′SL, LSTb and DSLNT all of which were at significantly higher concentrations in the preterm milk at postpartum weeks 2 to 8. The concentrations of LSTc (at weeks 1 to 4) and 6′SL (at weeks 2 to 4) were higher in term milk during the first month, but were not significantly different at later time points. 

**Figure 4:**
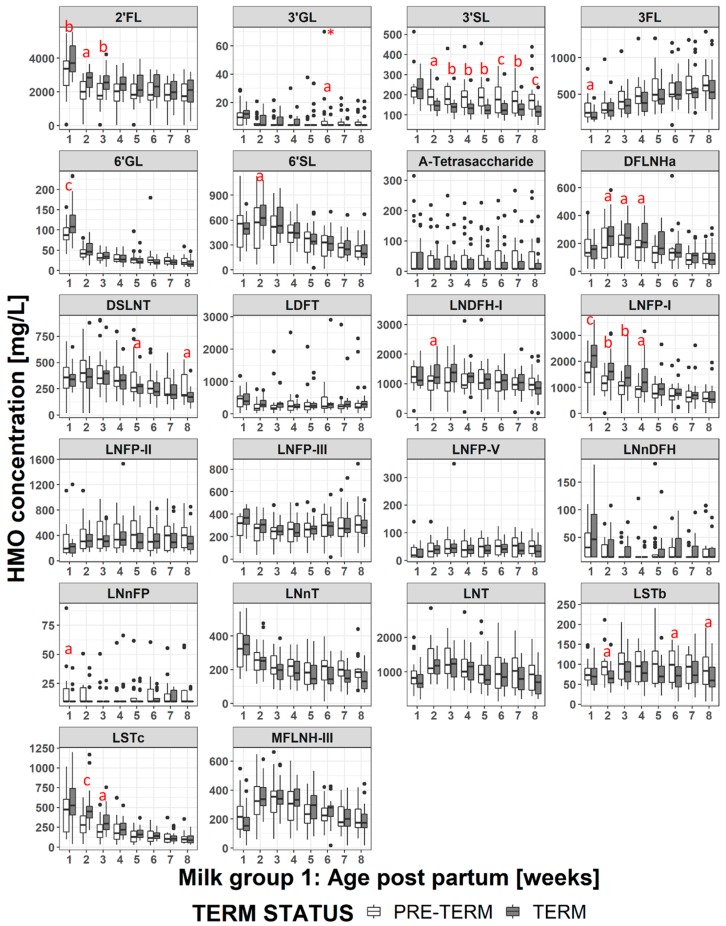
Comparison of HMO concentrations in group 1 term (grey bars) and preterm (white bars) milk. a: *p* < 0.05, b: *p* < 0.005 c: *p* < 0.0005.

**Figure 5:**
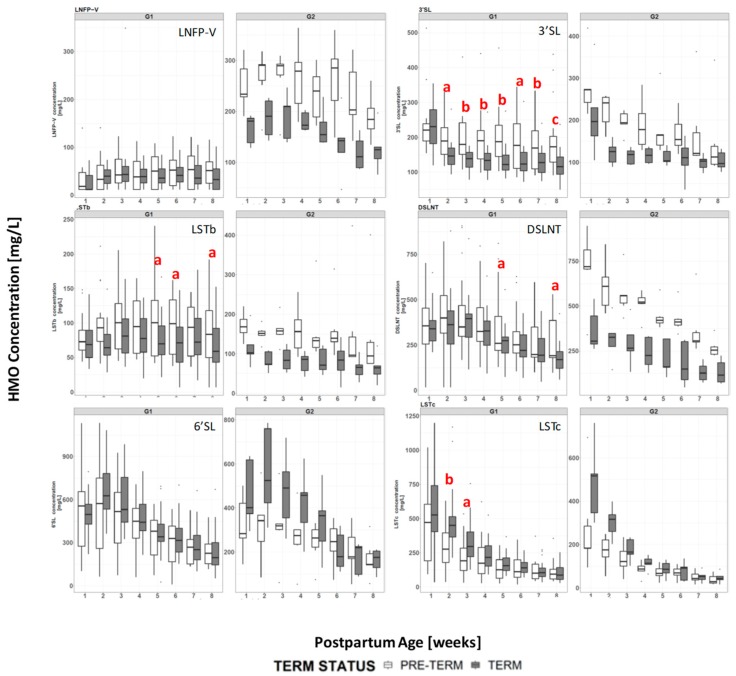
Comparison of selected HMO concentrations in group 1 (G1) and group 2 (G2) term (grey bars) and preterm (white bars) milk. a: *p* < 0.05, b: *p* < 0.005 c: *p* < 0.0005. Significance not tested in G2 milk due to low number of subjects (5 for each arm).

Comparing term and preterm milk at the same age postpartum, but restricting the comparison to milks within the same milk group the picture changes slightly (Figure 4, Figure 5, Supplementary Figure S2, and Supplementary Table S2). Within samples from milk group 1, LNFP-I concentrations are significantly higher in term milk at weeks 1-4, 2′FL concentrations are significantly higher in term milk samples at weeks 1-3 and DFLNHa concentrations are significantly higher in term milk samples at weeks 3-4 (Figure 4). These three HMO do not occur in group 2 or group 4 milks. The concentration of 3′SL remains significantly higher in group 1 preterm milk at weeks 2-8, but for DSLNT the difference between term and preterm milk is only significant at weeks 5 and 8 and for LSTb only at weeks 5, 6 and 8 (Figure 5). For 6′SL, the differences observed between term and preterm is lost when considering only group 1 milk and for LSTc the difference between term and preterm group 1 milk is only significant at weeks 2 to 3 (Figure 5). For the group 2 milks (Supplementary Figure S2) there are only 5 subjects in the term and 5 subjects in the preterm group so statistical significance was not tested at each visit. However from Figure 5 it can be seen that LNFP-V, 3′SL, 6′SL and DSLNT concentrations appear numerically much higher in preterm group 2 milk than term group 2 milk, while 6′SL and LSTc, appear numerically higher in term group 2 milk, especially in the first month. For group 3 milk there was only 1 subject in the term group and 1 subject in the preterm group so a comparison was not made, and for group 4 milk we had only one subject in the whole study. 

### 3.5. HMO Concentration in Term versus Preterm Milk at Equivalent Postmenstrual Age (Developmental Status)

**Figure 6:**
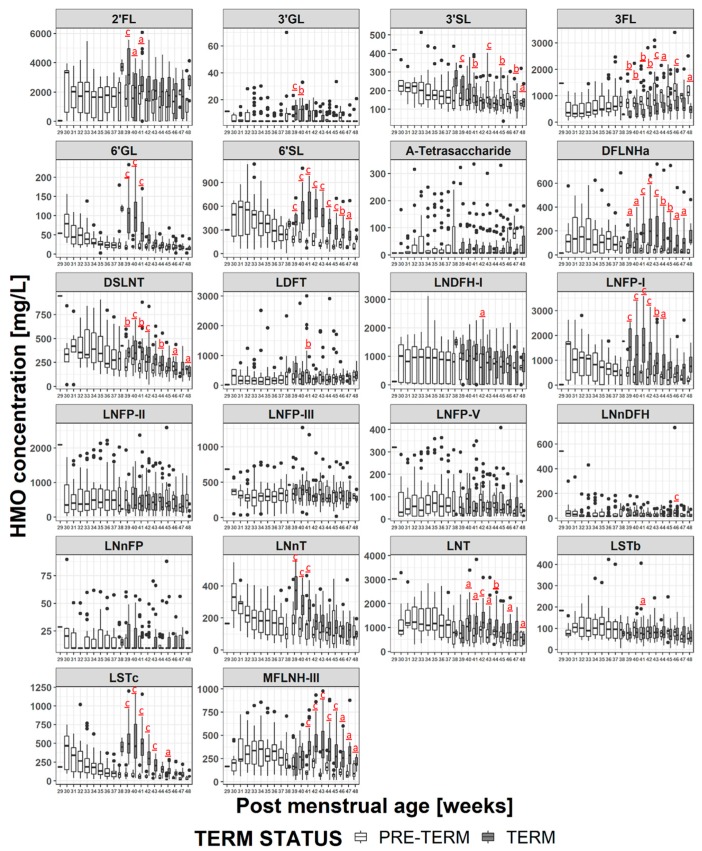
Comparison of the HMO concentration in the milk of mothers giving birth to term (grey bars) or preterm (white bars) infants at equivalent postmenstrual age. a: *p* < 0.05, b: *p* < 0.005 c: *p* < 0.0005.

**Figure 7:**
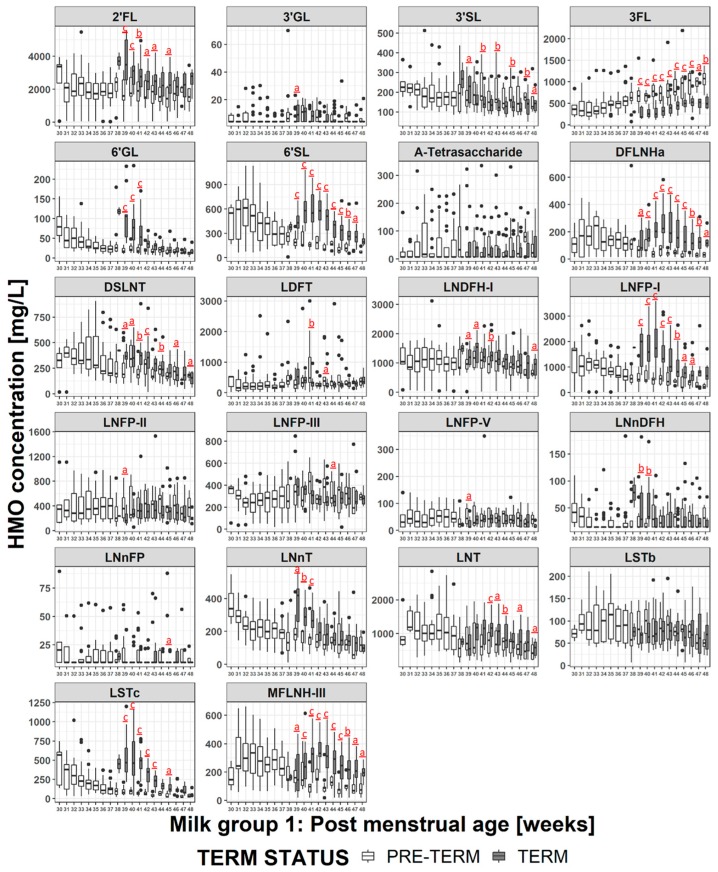
Comparison of the HMO concentration in group 1 milk of mothers giving birth to term (grey bars) or preterm (white bars) infants at equivalent gestational age. a: *p* < 0.05, b: *p* < 0.005 c: *p* < 0.0005.

When the milks are compared at infants’ equivalent postmenstrual age (Figure 6 and Supplementary Table S3), the concentrations of several HMOs (2′FL, 3′GL, 3′SL, 3FL, 6′GL, 6′SL, DFLNHa, DSLNT, LNFP-I, LNnDFH, LNnT, LNT, LSTc, MFLNH-III) are different at 2 or more visits in the preterm milk compared to term milk. The differences tend to manifest at weeks 38-41 where the concentration of several HMOs in term milk are at their maximum, and the concentration of 3FL is at the minimum. Thus, at equivalent developmental status, preterm infants consume higher concentrations of 3FL in their milk than term infants. However the preterm infants consume lower concentrations of most other HMOs. Making the same exercise only on milk group 1 samples (Figure 7 and Supplementary Table S4) doesn’t change this a lot although the magnitude and the duration of some differences (especially for 2′FL and LNFP-I) does change. Similar plots for milk group 2 are reported in Supplementary Figure S3, but no statistical analyses have been performed due to the low number of subjects. 

## 4. Discussion

In this study we have compared the content of 22 HMOs of 500 breast milk samples from mothers of preterm infants and term infants over time. The comparison has been performed both by equivalent stage of lactation (postpartum age) or equivalent postmenstrual age (developmental stage).

### 4.1. HMO in Term vs Preterm Milk at Equivalent Postpartum Age (Lactation Stages)

The HMO concentrations and trajectories over lactation were generally comparable in the milk of mothers giving birth preterm and term, but several important differences were observed, in particular for the sialylated oligosaccharides. This contrasts some of the previous studies [40,43,46] in which no significant differences were observed in HMO concentrations between term and preterm milk. Coppa et al. [39] reported that the sum of the measured HMOs in preterm milk was significantly higher than that of term milk at day 4 postpartum, but not at days 10 or 30 postpartum. Gabrielli et al. [41] also reported that concentrations of HMOs were higher in preterm milk, but did not go in to details. In this study, we observed the HMOs carrying sialic acid residues, 3′SL, LSTb and DSLNT, were present at significantly higher concentrations in preterm milk between weeks 2-8, while 6′SL (at weeks 2-4) and LSTc (at weeks 1-4) were higher in term milk. Wang et al. [47] reported that the total sialic acid content of preterm milk was significantly higher than that of term milk up to 45 days postpartum, but not beyond 92 days postpartum. In this study we did not collect milk from term infants beyond 56 days postpartum. Although our data appear to be in line with the observations of Wang et al. [47], they contradict the data of Kunz et al. [43] and Spevacek et al. [46] who did not report differences in concentrations of sialylated HMO between term and preterm milk. Variability in HMO concentration between individuals is high, and both of these studies were slightly smaller than ours, which is itself rather small. As pointed out by Kunz et al. [43] it is difficult to compare quantitative HMO results between studies, due to the lack of standardized methods of analysis, milk collection timing and milk collection methodology. Further, although some studies have considered the impact of milk group and stage of lactation on HMO concentrations, they have not matched both stage of lactation and milk group prior to comparing term vs preterm milk, which could mask (or enhance) any compositional differences.

As to why we observed higher concentrations of several sialylated HMOs in preterm milk and lower concentrations of other sialylated HMOs, we can only speculate. From a structural viewpoint, 6′SL and LSTc, both at higher concentrations in term milk, both contain the structural motif α-Neu5Ac(2→6)β-D-Gal- which is absent from 3′SL, LSTb and DSLNT. 3′SL, LSTb and DSLNT all contain the structural motifs α-Neu5Ac(2→6)β-D-GlcNAc- and/or α-Neu5Ac(2→3)β-D-Gal-. Therefore we may envisage that the sialyltransferase responsible for attaching sialic acid to galactose through an α-1,6-linkage has reduced activity after preterm delivery while those responsible for attaching sialic acid through an α-1,3 linkage to galactose or an α-1,6-linkage to N-acetylglucosamine have increased activity. During the final trimester of gestation, the ganglioside density of the cerebral cortex increases significantly [48], and sialic acids are an important component of the gangliosides. One may also postulate that at this stage of gestation the mother has an increased production of sialic acid to help with the baby’s brain development. If the baby is born prematurely, and lactation begins early, the upregulation of the system to produce sialic acids may result in an increased production of sialylated HMOs. Dietary sources of sialic acid have been demonstrated to be processed by the body and the sialic acids incorporated in to glycoproteins [49]. Data from Wang et al. [24] have also demonstrated that supplementing pigs’ diets with a dietary source of sialic acid improves learning and memory. The higher concentration of sialylated HMOs in preterm milk may be a factor that contributes to the important benefits experienced by preterm infants consuming their own mothers’ milk.

DSLNT has been observed to protect neonatal rats from development of NEC [30]. An observational study in preterm human infants also observed that infants receiving milk with higher levels of DSLNT were less likely to develop NEC [32] leading to the proposition that DSLNT concentrations could be used as a possible marker for risk of developing NEC. This work demonstrates that the mothers own milk of preterm infants is probably a good source of DSLNT. Our data also suggest children receiving group 2 milk may receive higher concentrations of DSLNT than those receiving group 1 milk, but a larger study would be needed to test if the difference is significant, and to determine if infants receiving group 2 milk may be better protected from NEC.

### 4.2. HMO at Equivalent Postmenstrual Age (Developmental Status)

As shown above, when comparisons were made at equivalent postpartum age, there were few differences in concentrations of most HMOs between term milk and preterm milk and the trajectories of concentration during lactation were comparable. This can indicate that birth sets off a program that defines the HMO trajectory with most HMOs showing a decrease in concentration with stage of lactation with the exception of 3FL, which increases. 

Since the HMO concentration varies with stage of lactation, when the milk is compared at infants’ equivalent postmenstrual age, the concentrations of most of the HMOs in preterm milk are lower than in the term milk, with the exception of 3FL (since it increases during lactation) and 3′SL (since it is generally in higher concentrations in preterm milk). The largest differences manifest around 40 weeks of postmenstrual age, which is when the milk for term infants contains the highest concentrations of HMOs. Interestingly, as the postmenstrual age increases, the differences reduce, and for some HMOs the concentrations are equivalent by 44-46 weeks postmenstrual age, while for some others a significant difference was maintained for the duration of the study. The biological relevance of these differences is unclear, and would be worthy of future investigation. Making the hypothesis that infants need to be exposed to a certain amount of HMOs at specific stages of their development, we can presume that preterm infants may be losing out by being exposed to lower amounts of HMOs than term infants as similar stages of development. In such a case supplementing preterm milk with HMOs is likely to be beneficial. Alternatively, perhaps the HMOs help the infant adjust to life outside the womb, and the concentrations in the mothers’ milk are appropriate. However a very preterm infant may not receive full enteral feeding until several days or even weeks after delivery, meaning that the actual amount of HMOs they receive will be below what they would be receiving if they could be immediately fully breast fed. In such cases HMO supplementation may also be beneficial. Clinical evidence shows that premature infants greatly benefit from being fed own mother’s milk [3,50], or, alternatively, being fed donor human milk [50]. For very preterm infants the milk needs to be fortified with energy, proteins and minerals [3]. Now that HMOs start to be commercially available, HMO fortification is also possible, but further work is needed to confirm the potential benefits and the appropriate timing and dosages for intervention.

### 4.3. Impact of Milk Group

In general, when comparing HMO compositions, it is important to consider the milk group. In this study the distribution of milk groups is comparable between mothers giving birth term or preterm, with the majority of mothers producing group 1 milk (milk containing oligosaccharides indicating both FUT2 and FUT3 were active). Milk group 2 was next most common with 5 mothers in each group. The equal distributions suggest that there is no link between the genetic factors controlling the milk groups, and the likelihood of giving birth prematurely, although this study is too small to properly address this question. 

In this study, when combining data from all milk groups at the same postpartum stages it appears that there was relatively little difference in the concentrations of the neutral HMOs between term or preterm milk, similar to what has been observed previously [40,43,46]. However when only the group 1 milks were compared, three neutral HMOs, 2′FL, LNFP-I, and DFLNHa, were at significantly lower concentrations in preterm milk during the first month of lactation. 2′FL was 700-800 mg/L (20–27%) lower, LNFP-I was 420–590 mg/L (27–32%) lower and DFLNHa was 60-70 mg/L (26–30%) lower. These differences were hidden by the increased variability introduced by the inclusion of the data from the other milk groups, especially milk group 2 with inactive FUT2. All of these structures contain α-1,2-linked fucose residues and are present in significant concentrations only in milk of groups 1 and 3. The observation that these structures are at lower concentrations in group 1 preterm milk implies that the fucosyltransferase-2 enzyme (FUT2) is not fully active during the first month of lactation when an infant is born very preterm as suggested previously [36]. Higher levels of fucosylation have been shown to increase protection against infection [18] but the clinical relevance of the magnitude of the differences remains to be assessed.

Interestingly in group 1 milk, 3′SL is the only sialylated HMO that remains higher in preterm milk across weeks 2-8 (as observed when studying all milk groups together). The differences in concentrations of LSTb and DSLNT between term and preterm milks are largely lost, the differences being significant only at 2 or 3 non-consecutive time points. Likewise for 6′SL and LSTc, which were observed as being significantly higher in term milk when all milk groups were combined, the significance is largely lost when considering only milk group 1 data. Most of the differences in concentrations of sialylated structures appears to be driven by their concentrations in the group 2 milks (Figure 5). The reasons for this are not clear, but one may postulate that FUT2 outcompetes the other enzymes for access to the core structures, and so when FUT2 is active (even if slightly less active than normal for preterm milks), the difference in activity of the sialyltransferases is masked. However, when FUT2 is not active, the difference in sialyltransferase activities becomes more apparent. 

Looking at differences in HMO concentrations at equivalent postmenstrual age, and considering only milk group 1 (Figure 7), does not much change the observations made considering all milk groups (Figure 6) with most of the differences being driven by the stage of lactation. However, it does emphasize the differences in the HMOs containing α-1,2-linked fucose. For example looking at all milk groups, 2′FL concentrations appear significantly different only between weeks 39-41, but when considering only milk group 1 then the concentration differences are significant between weeks 39-43 and at week 45. Similar phenomena can be observed for LNFP-I and LNDFH-I. This highlights the importance of considering milk group when making comparisons between different milk samples. 

### 4.4. Strengths and Limitations of the Study

The main limitations of this study are the limited sample size and the monocentric design of the study. One may also argue that collecting milk at only one time of the day, the first milk expression in the morning, may mean the data are not representative of the milk composition during the whole day. Conversely one may see this as a strength of the study, since the main aim was to compare term versus preterm milk and sampling at different times of the day could have introduced additional variation not specifically related to the comparison of term versus preterm milk. Certainly a strength of the study was the very tight collection windows at each stage of lactation, each window being only ±1 day. It is well established that the HMO concentration changes at different stages of lactation [51,52,53,54,55,56], especially during the first few months of lactation. Keeping the milk collection window tight reduces variability due to differences in sampling day. The study protocol places quite a burden on the mothers who need to collect the full milk expression from a single breast at every sampling point. Despite this, we had very few drop outs from the study, the support provided to the mothers by the lactation nurse was surely a key factor in the successful retention of the volunteers. Importantly, the analytical method used for determination of the HMOs has been extensively validated [45]. To ensure the method performance was maintained day to day, a reference sample of pooled human milk was analysed with every batch of analyses, and at least every 25 samples. Such rigor assured that the variability introduced by the analytical method was also kept to a minimum. Milk group assignments have been done based on the concentrations of 2′FL and LNFP-II in the milk. Appropriate cut-off concentrations for making milk group assignments have not yet been established, so we have based our cut-offs based on the performance of the analytical method. Unfortunately, due to the small sample size, it was not possible to make statistical comparisons between term and preterm milk of milk groups 2,3 or 4. In this study we have only determined the concentrations of 22 out of over 160 HMOs. Although those 22 include the most abundant HMOs, concentrations of a large number of HMOs and how they are impacted by preterm birth remains unknown.

## 5. Conclusions

The HMO composition and concentration trajectory of term and preterm milk are largely comparable at equivalent infant postpartum age, suggesting that birth triggers a program that defines the HMO trajectory during lactation. Consequently, significant differences exist between preterm and term infants milk when comparing HMO concentrations at equivalent postmenstrual ages. Nevertheless some differences were observed when the comparison was made at the same postpartum age, in particular for the sialylated oligosaccharides and oligosaccharides containing α-1,2-linked fucose. The data suggest that when an infant is born preterm, FUT2 is not fully active during the first month of lactation, leading to reduced concentrations of HMOs such as 2′FL and LNFP-I. In addition the expression of enzymes responsible for sialyllation of HMOs is also perturbed leading to differences in concentrations of the sialylated HMOs. Interestingly, these observations are not fully consistent with previous studies in preterm infants. This study was small, thus the observations need corroboration in larger cohorts. The possible physiological significance of the observations remain to be determined.

## Supplementary Materials

The following are available online at https://www.mdpi.com/2072-6643/11/6/1282/s1, Figure S1: Structures of the Determined HMO Depicted Using Symbol Nomenclature, Figure S2: Mean Concentration of each HMO in Group 2 Milk for Term and Preterm Infants at Equivalent Stage of Lactation, Figure S3: Mean Concentration of each HMO in Group 2 Milk for Term and Preterm Infants at Equivalent Postmenstrual Age, Table S1: Concentration of Human Milk Oligosaccharides in Term or Preterm Milk At Different Weeks Postpartum, Table S2: Concentration of Human Milk Oligosaccharides in Term or Preterm Milk At Different Weeks Postpartum Separated By Milk Group, Table S3: Concentration of Human Milk Oligosaccharides in Term or Preterm Milk At Specified Postmenstrual Age, Table S4: Concentration of Human Milk Oligosaccharides in Term or Preterm Milk At Specified Postmenstrual Age Separated By Milk Group.
