# Supplementary material for: Human Milk Oligosaccharides in the Milk of Mothers Delivering Term versus Preterm Infants
Source: Nutrients. 2019 Jun 5;11(6):1282. doi: 10.3390/nu11061282 (PMC6627155; doi:10.3390/nu11061282)
Supplement: Supplementary file 1 [file nutrients-11-01282-s001.zip › nutrients-512673-supplementary/Supp_Fig_S3_G2_postmens.pdf]

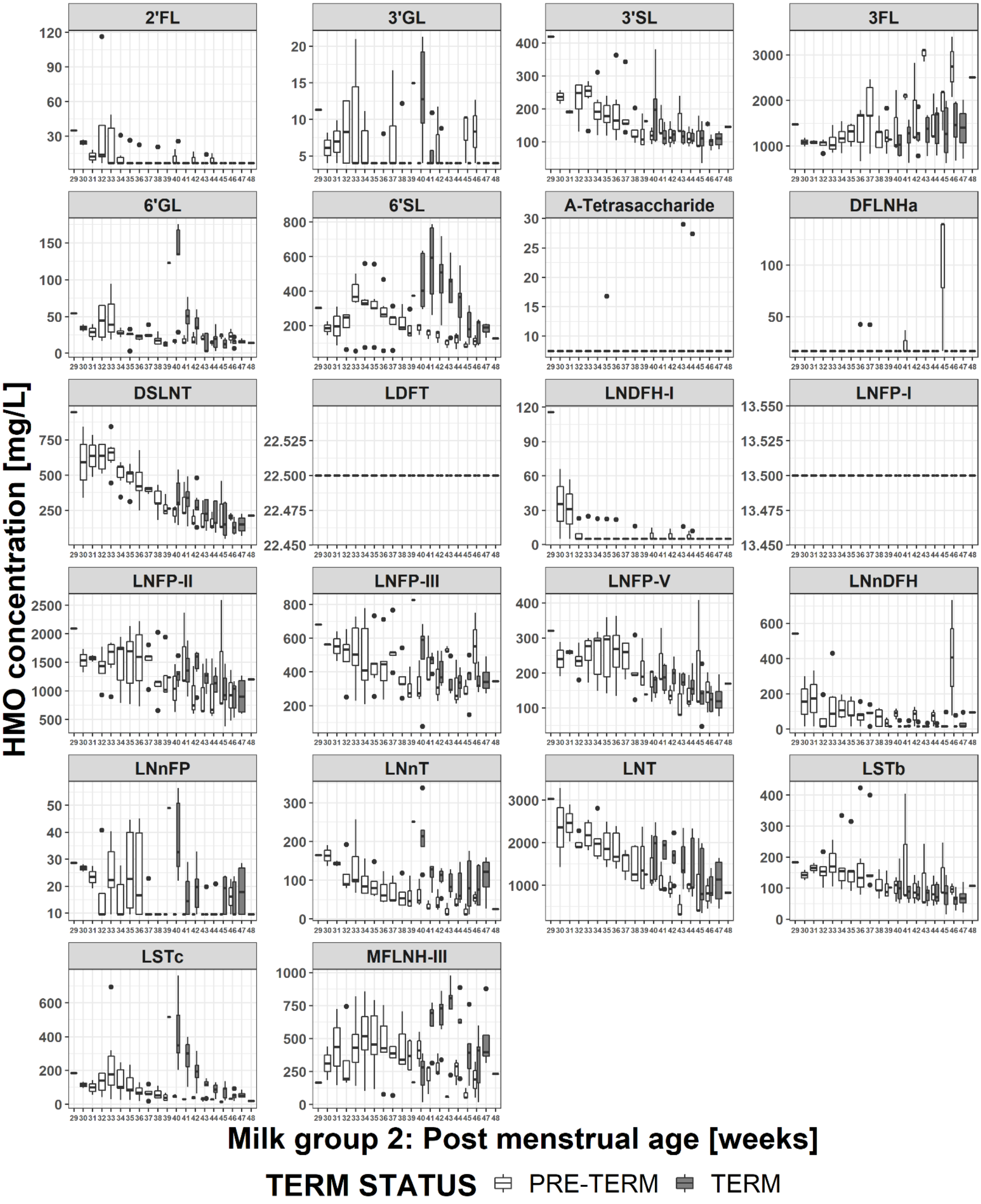

Figure S3: Mean concentration of each HMO in group 2 milk for term (grey) and preterm (white) infants at equivalent postmenstrual age.
