# Supplementary material for: Human Milk Oligosaccharides in the Milk of Mothers Delivering Term versus Preterm Infants
Source: Nutrients. 2019 Jun 5;11(6):1282. doi: 10.3390/nu11061282 (PMC6627155; doi:10.3390/nu11061282)
Supplement: Supplementary file 1 [file nutrients-11-01282-s001.zip › nutrients-512673-supplementary/Supp_Table_S1_HMO_PP_All.pdf]

Table S1 Concentration of Human Milk Oligosaccharides in Term or Preterm Milk At Different Weeks Postpartum

** When there are results below the method limit of quanfification (LoQ) the result has been assigned value of 0.5 × LoQ, hence the minimum value appears to be the same in many cases. When a large number of datapoints are below LoQ this can also have the effect that the median = minimum.*

HMO	Study Arm	Week Post Partum	N	HMO Concentration (mg/L) min *	max	mean	sd	median *	Quartile 1 (25%)	Quartile 3 (75%)
2'FL	PRE-TERM	1	25	6.500	5478	2504	1633	2923	1402	3633
2'FL	PRE-TERM	2	25	6.500	3465	1672	1124	1867	832.5	2606
2'FL	PRE-TERM	3	25	6.500	2995	1582	1058	1723	824.5	2433
2'FL	PRE-TERM	4	24	6.500	3592	1651	1146	1966	620.7	2414
2'FL	PRE-TERM	5	25	6.500	3063	1595	1040	1815	944.5	2463
2'FL	PRE-TERM	6	24	6.500	3335	1578	1133	1686	195.6	2519
2'FL	PRE-TERM	7	24	6.500	2969	1533	1034	1714	722.5	2365
2'FL	PRE-TERM	8	24	6.500	3353	1556	1084	1705	720.6	2378
2'FL	PRE-TERM	10	23	6.500	3369	1499	999.1	1552	1019	2139
2'FL	PRE-TERM	12	21	6.500	3574	1306	1032	1427	19.65	1921
2'FL	PRE-TERM	14	21	6.500	2946	1294	1017	1323	32.90	2124
2'FL	PRE-TERM	16	19	6.500	3455	1389	985.1	1461	767.1	2062
2'FL	TERM	1	28	6.500	6076	3157	1908	3443	2673	4674
2'FL	TERM	2	26	6.500	5572	2207	1429	2470	1746	3118
2'FL	TERM	3	28	6.500	4390	2139	1328	2309	1699	2920
2'FL	TERM	4	28	6.500	4556	2007	1250	2128	1525	2875
2'FL	TERM	5	28	6.500	4090	1938	1247	2043	1499	2905
2'FL	TERM	6	27	6.500	3666	1801	1187	1752	1137	2938
2'FL	TERM	7	27	6.500	3530	1664	1135	1703	852.3	2567
2'FL	TERM	8	28	6.500	4133	1621	1179	1650	596.7	2505
3'GL	PRE-TERM	1	25	4.000	28.82	11.69	7.993	10.44	4.000	15.69
3'GL	PRE-TERM	2	25	4.000	20.70	6.209	4.194	4.000	4.000	8.204
3'GL	PRE-TERM	3	25	4.000	21.56	5.801	4.235	4.000	4.000	4.000
3'GL	PRE-TERM	4	24	4.000	30.06	5.580	5.476	4.000	4.000	4.000
3'GL	PRE-TERM	5	25	4.000	37.74	6.624	7.134	4.000	4.000	4.000
3'GL	PRE-TERM	6	24	4.000	70.00	9.044	13.78	4.000	4.000	9.442
3'GL	PRE-TERM	7	24	4.000	23.04	6.206	5.114	4.000	4.000	4.000
3'GL	PRE-TERM	8	24	4.000	21.24	6.525	4.695	4.000	4.000	8.039
3'GL	PRE-TERM	10	23	4.000	21.49	5.887	4.554	4.000	4.000	4.000

Table S1 Concentration of Human Milk Oligosaccharides in Term or Preterm Milk At Different Weeks Postpartum

** When there are results below the method limit of quanfification (LoQ) the result has been assigned value of 0.5 × LoQ, hence the minimum value appears to be the same in many cases. When a large number of datapoints are below LoQ this can also have the effect that the median = minimum.*

				HMO Concentration (mg/L)						
**HMO**	**Study Arm**	**Week Post Partum**	**N**	**min ***	**max**	**mean**	**sd**	**median ***	**Quartile 1 (25%)**	**Quartile 3 (75%)**
3'GL	PRE-TERM	12	21	4.000	15.76	6.068	3.922	4.000	4.000	4.000
3'GL	PRE-TERM	14	21	4.000	15.21	6.057	3.544	4.000	4.000	8.889
3'GL	PRE-TERM	16	19	4.000	12.84	5.001	2.500	4.000	4.000	4.000
3'GL	TERM	1	28	4.000	32.97	12.96	6.346	12.34	8.979	16.16
3'GL	TERM	2	26	4.000	19.72	7.139	4.928	4.000	4.000	10.78
3'GL	TERM	3	28	4.000	17.67	6.645	3.740	4.000	4.000	9.328
3'GL	TERM	4	28	4.000	16.81	5.535	3.064	4.000	4.000	5.053
3'GL	TERM	5	28	4.000	33.42	6.181	6.387	4.000	4.000	4.000
3'GL	TERM	6	27	4.000	16.35	4.898	2.814	4.000	4.000	4.000
3'GL	TERM	7	27	4.000	14.81	4.754	2.380	4.000	4.000	4.000
3'GL	TERM	8	28	4.000	20.73	5.247	3.945	4.000	4.000	4.000
3'SL	PRE-TERM	1	25	154.6	513.3	238.9	82.49	223.9	189.8	241.0
3'SL	PRE-TERM	2	25	117.4	329.8	198.2	55.49	198.8	151.8	228.2
3'SL	PRE-TERM	3	25	107.7	430.3	196.5	65.79	184.5	166.8	223.3
3'SL	PRE-TERM	4	24	98.75	439.9	193.4	73.45	180.8	146.7	220.9
3'SL	PRE-TERM	5	25	103.5	455.8	194.4	77.36	177.6	154.4	219.4
3'SL	PRE-TERM	6	24	85.96	344.4	186.1	67.69	176.8	131.7	239.0
3'SL	PRE-TERM	7	24	91.34	362.9	182.8	77.81	165.5	118.6	223.5
3'SL	PRE-TERM	8	24	91.08	438.1	185.0	97.06	157.3	116.3	205.9
3'SL	PRE-TERM	10	23	88.38	403.2	171.8	75.52	151.9	123.5	169.5
3'SL	PRE-TERM	12	21	92.61	382.6	182.1	77.90	169.9	123.5	219.3
3'SL	PRE-TERM	14	21	82.71	291.3	163.9	65.70	146.0	119.6	224.8
3'SL	PRE-TERM	16	19	86.41	325.0	175.9	74.26	155.6	120.5	230.4
3'SL	TERM	1	28	105.2	380.6	222.5	74.17	219.5	160.7	272.8
3'SL	TERM	2	26	89.66	280.3	147.0	40.84	146.0	121.8	167.0
3'SL	TERM	3	28	78.49	282.3	135.4	39.88	137.2	111.4	154.1
3'SL	TERM	4	28	77.94	272.4	134.1	38.85	133.8	104.6	153.6
3'SL	TERM	5	28	82.07	274.9	129.1	40.01	123.9	103.1	144.5
3'SL	TERM	6	27	35.33	303.0	130.7	48.23	124.1	103.3	152.7

Table S1 Concentration of Human Milk Oligosaccharides in Term or Preterm Milk At Different Weeks Postpartum

** When there are results below the method limit of quanfification (LoQ) the result has been assigned value of 0.5 × LoQ, hence the minimum value appears to be the same in many cases. When a large number of datapoints are below LoQ this can also have the effect that the median = minimum.*

				HMO Concentration (mg/L)						
HMO	Study Arm	Week Post Partum	N	min *	max	mean	sd	median *	Quartile 1 (25%)	Quartile 3 (75%)
3'SL	TERM	7	27	74.58	240.1	124.0	38.26	120.2	98.57	148.9
3'SL	TERM	8	28	50.25	236.8	119.6	38.17	115.1	93.69	140.5
3FL	PRE-TERM	1	25	64.12	1472	461.1	405.7	246.6	186.2	515.3
3FL	PRE-TERM	2	25	148.3	1367	479.2	367.5	308.8	239.2	547.4
3FL	PRE-TERM	3	25	181.0	1359	565.8	375.5	404.7	305.3	616.5
3FL	PRE-TERM	4	24	158.0	1649	628.9	418.1	494.0	375.7	683.3
3FL	PRE-TERM	5	25	215.6	2284	729.5	497.7	516.1	428.2	829.2
3FL	PRE-TERM	6	24	75.94	1663	690.0	409.6	573.1	466.0	873.5
3FL	PRE-TERM	7	24	232.4	1825	757.7	423.4	632.8	506.1	867.7
3FL	PRE-TERM	8	24	224.9	2465	830.9	538.5	700.4	547.9	910.1
3FL	PRE-TERM	10	23	241.8	2821	868.7	595.6	745.6	515.8	926.9
3FL	PRE-TERM	12	21	278.6	3101	1074	654.3	838.2	689.8	1202
3FL	PRE-TERM	14	21	298.6	3398	1205	823.2	865.1	653.8	1250
3FL	PRE-TERM	16	19	325.1	2505	1037	554.7	967.0	650.1	1180
3FL	TERM	1	28	39.33	1240	346.4	328.3	207.4	166.9	345.8
3FL	TERM	2	26	66.65	1361	455.7	381.4	305.1	212.6	443.6
3FL	TERM	3	28	82.63	1577	493.6	377.8	368.8	235.0	514.2
3FL	TERM	4	28	99.24	1861	557.8	435.2	410.9	269.4	640.6
3FL	TERM	5	28	95.34	1720	621.8	431.9	456.7	377.0	705.0
3FL	TERM	6	27	102.9	1983	683.0	434.9	538.7	439.9	783.6
3FL	TERM	7	27	118.2	1978	720.8	473.4	536.2	448.1	989.3
3FL	TERM	8	28	131.6	2013	725.8	489.6	543.3	459.9	804.6
6'GL	PRE-TERM	1	25	39.10	156.4	86.74	29.58	81.58	75.28	104.1
6'GL	PRE-TERM	2	25	17.55	80.30	42.88	15.72	40.93	31.35	51.04
6'GL	PRE-TERM	3	25	16.66	61.99	33.86	11.85	32.89	25.85	39.49
6'GL	PRE-TERM	4	24	18.30	57.48	29.78	11.07	25.41	21.74	33.27
6'GL	PRE-TERM	5	25	16.16	96.94	29.85	16.34	25.75	21.56	30.35
6'GL	PRE-TERM	6	24	3.150	179.6	31.70	33.08	25.25	19.44	30.44
6'GL	PRE-TERM	7	24	11.33	43.71	22.49	9.416	18.39	15.21	26.61

Table S1 Concentration of Human Milk Oligosaccharides in Term or Preterm Milk At Different Weeks Postpartum

** When there are results below the method limit of quanfification (LoQ) the result has been assigned value of 0.5 × LoQ, hence the minimum value appears to be the same in many cases. When a large number of datapoints are below LoQ this can also have the effect that the median = minimum.*

				HMO Concentration (mg/L)						
HMO	Study Arm	Week Post Partum	N	min *	max	mean	sd	median *	Quartile 1 (25%)	Quartile 3 (75%)
6'GL	PRE-TERM	8	24	7.746	59.26	22.25	11.18	18.91	14.64	25.77
6'GL	PRE-TERM	10	23	9.255	48.40	20.02	9.070	17.89	13.98	22.46
6'GL	PRE-TERM	12	21	6.997	44.84	19.92	9.116	17.17	13.83	24.69
6'GL	PRE-TERM	14	21	8.465	52.92	19.11	10.17	15.52	12.97	22.66
6'GL	PRE-TERM	16	19	3.150	46.05	16.45	9.199	14.07	11.70	20.14
6'GL	TERM	1	28	53.98	234.0	128.2	46.10	118.9	99.42	154.1
6'GL	TERM	2	26	12.64	94.87	53.25	20.51	50.45	38.31	68.33
6'GL	TERM	3	28	8.541	59.24	36.37	12.85	34.45	29.50	45.54
6'GL	TERM	4	28	3.150	59.96	26.99	13.04	27.01	19.04	36.29
6'GL	TERM	5	28	3.150	68.27	23.44	13.82	21.06	17.05	29.50
6'GL	TERM	6	27	3.150	39.81	19.79	8.848	18.16	14.78	25.80
6'GL	TERM	7	27	3.150	38.09	18.74	8.369	16.93	14.22	23.12
6'GL	TERM	8	28	3.150	47.47	17.71	10.64	14.55	11.59	21.24
6'SL	PRE-TERM	1	25	103.1	1132	492.2	255.8	501.0	292.8	656.3
6'SL	PRE-TERM	2	25	63.98	1135	505.5	279.6	559.2	266.6	654.3
6'SL	PRE-TERM	3	25	61.39	926.1	455.1	228.0	489.0	309.8	639.8
6'SL	PRE-TERM	4	24	53.89	705.7	395.5	174.6	432.2	288.0	531.3
6'SL	PRE-TERM	5	25	74.89	569.3	320.4	138.9	331.6	218.7	417.6
6'SL	PRE-TERM	6	24	10.00	534.3	291.3	136.9	317.6	190.5	385.9
6'SL	PRE-TERM	7	24	55.02	524.7	247.5	117.8	253.4	150.7	315.7
6'SL	PRE-TERM	8	24	57.52	476.6	226.2	105.3	208.7	145.3	287.2
6'SL	PRE-TERM	10	23	91.24	309.4	174.0	65.07	163.8	119.9	190.7
6'SL	PRE-TERM	12	21	65.69	256.2	143.5	49.62	140.1	105.5	184.7
6'SL	PRE-TERM	14	21	60.09	212.2	111.6	43.37	99.71	77.58	145.4
6'SL	PRE-TERM	16	19	46.43	196.6	99.87	44.93	97.87	60.33	129.2
6'SL	TERM	1	28	222.0	794.7	497.6	141.8	469.7	396.2	621.3
6'SL	TERM	2	26	311.3	1084	646.4	191.1	612.6	526.0	783.4
6'SL	TERM	3	28	262.0	985.4	567.1	178.1	521.6	441.8	705.6
6'SL	TERM	4	28	202.8	797.3	477.1	155.4	450.4	381.0	561.3

Table S1 Concentration of Human Milk Oligosaccharides in Term or Preterm Milk At Different Weeks Postpartum

** When there are results below the method limit of quanfification (LoQ) the result has been assigned value of 0.5 × LoQ, hence the minimum value appears to be the same in many cases. When a large number of datapoints are below LoQ this can also have the effect that the median = minimum.*

				HMO Concentration (mg/L)						
**HMO**	**Study Arm**	**Week Post Partum**	**N**	**min ***	**max**	**mean**	**sd**	**median ***	**Quartile 1 (25%)**	**Quartile 3 (75%)**
6'SL	TERM	5	28	24.60	693.3	357.8	151.5	342.7	299.7	397.6
6'SL	TERM	6	27	112.5	700.9	306.4	136.1	294.2	218.2	342.3
6'SL	TERM	7	27	89.66	662.3	257.7	135.0	227.1	173.3	281.3
6'SL	TERM	8	28	53.06	671.2	219.8	134.1	191.9	144.8	241.8
A-Tetrasaccharide	PRE-TERM	1	25	7.500	315.1	49.87	83.15	7.500	7.500	54.83
A-Tetrasaccharide	PRE-TERM	2	25	7.500	218.2	35.69	57.71	7.500	7.500	27.92
A-Tetrasaccharide	PRE-TERM	3	25	7.500	250.0	38.09	64.00	7.500	7.500	23.53
A-Tetrasaccharide	PRE-TERM	4	24	7.500	224.7	39.43	62.08	7.500	7.500	28.80
A-Tetrasaccharide	PRE-TERM	5	25	7.500	226.0	37.74	58.76	7.500	7.500	35.77
A-Tetrasaccharide	PRE-TERM	6	24	7.500	232.6	40.88	62.88	7.500	7.500	32.20
A-Tetrasaccharide	PRE-TERM	7	24	7.500	266.6	40.79	68.30	7.500	7.500	27.95
A-Tetrasaccharide	PRE-TERM	8	24	7.500	262.8	43.99	75.96	7.500	7.500	22.59
A-Tetrasaccharide	PRE-TERM	10	23	7.500	322.7	43.80	77.66	7.500	7.500	22.78
A-Tetrasaccharide	PRE-TERM	12	21	7.500	334.5	40.48	80.88	7.500	7.500	7.500
A-Tetrasaccharide	PRE-TERM	14	21	7.500	300.7	40.66	71.61	7.500	7.500	29.61
A-Tetrasaccharide	PRE-TERM	16	19	7.500	329.8	48.97	81.90	7.500	7.500	57.85
A-Tetrasaccharide	TERM	1	28	7.500	188.2	38.15	52.25	7.500	7.500	47.73
A-Tetrasaccharide	TERM	2	26	7.500	149.0	28.35	35.70	7.500	7.500	40.12
A-Tetrasaccharide	TERM	3	28	7.500	153.9	25.51	35.48	7.500	7.500	30.76
A-Tetrasaccharide	TERM	4	28	7.500	162.3	27.80	36.68	7.500	7.500	37.78
A-Tetrasaccharide	TERM	5	28	7.500	138.9	25.40	32.20	7.500	7.500	29.91
A-Tetrasaccharide	TERM	6	27	7.500	183.8	26.51	41.05	7.500	7.500	27.78
A-Tetrasaccharide	TERM	7	27	7.500	165.4	25.18	38.09	7.500	7.500	23.67
A-Tetrasaccharide	TERM	8	28	7.500	181.0	25.64	40.27	7.500	7.500	24.06
DFLNHa	PRE-TERM	1	25	16.50	577.3	169.0	147.9	132.0	47.48	230.6
DFLNHa	PRE-TERM	2	25	16.50	495.2	187.0	152.2	155.5	16.50	311.1
DFLNHa	PRE-TERM	3	25	16.50	430.4	164.7	126.8	184.4	16.50	253.9
DFLNHa	PRE-TERM	4	24	16.50	583.6	153.2	134.3	147.2	16.50	216.1
DFLNHa	PRE-TERM	5	25	16.50	623.8	125.1	125.7	118.0	52.33	151.3

Table S1 Concentration of Human Milk Oligosaccharides in Term or Preterm Milk At Different Weeks Postpartum

** When there are results below the method limit of quanfification (LoQ) the result has been assigned value of 0.5 × LoQ, hence the minimum value appears to be the same in many cases. When a large number of datapoints are below LoQ this can also have the effect that the median = minimum.*

				HMO Concentration (mg/L)						
HMO	Study Arm	Week Post Partum	N	min *	max	mean	sd	median *	Quartile 1 (25%)	Quartile 3 (75%)
DFLNHa	PRE-TERM	6	24	16.50	685.1	140.7	162.2	103.9	31.82	145.6
DFLNHa	PRE-TERM	7	24	16.50	418.7	97.66	94.38	77.90	35.96	119.6
DFLNHa	PRE-TERM	8	24	16.50	351.9	96.41	85.53	68.39	33.80	126.4
DFLNHa	PRE-TERM	10	23	16.50	249.7	53.43	53.14	37.05	16.50	75.45
DFLNHa	PRE-TERM	12	21	16.50	179.2	42.18	37.44	35.46	16.50	49.76
DFLNHa	PRE-TERM	14	21	16.50	139.6	41.28	29.94	39.21	16.50	53.54
DFLNHa	PRE-TERM	16	19	16.50	60.97	31.15	16.71	16.50	16.50	44.38
DFLNHa	TERM	1	28	16.50	488.4	139.5	113.4	142.0	49.77	182.3
DFLNHa	TERM	2	26	16.50	664.0	229.7	177.1	216.4	100.6	310.1
DFLNHa	TERM	3	28	16.50	759.1	223.0	174.1	219.6	96.56	321.9
DFLNHa	TERM	4	28	16.50	709.4	207.3	166.5	191.6	76.56	300.2
DFLNHa	TERM	5	28	16.50	747.0	174.6	163.0	135.3	64.30	264.9
DFLNHa	TERM	6	27	16.50	563.0	135.0	128.2	123.5	25.38	172.4
DFLNHa	TERM	7	27	16.50	525.5	106.9	111.2	76.69	16.50	125.0
DFLNHa	TERM	8	28	16.50	463.1	99.16	107.9	66.48	16.50	123.3
DSLNT	PRE-TERM	1	25	17.50	946.4	422.7	231.9	394.9	271.2	531.0
DSLNT	PRE-TERM	2	25	17.50	843.2	465.5	186.7	457.2	381.5	557.9
DSLNT	PRE-TERM	3	25	169.1	908.0	467.8	197.8	433.2	337.3	555.3
DSLNT	PRE-TERM	4	24	128.4	796.0	415.6	177.9	355.8	292.5	530.7
DSLNT	PRE-TERM	5	25	129.9	811.9	378.1	178.7	334.8	248.2	443.0
DSLNT	PRE-TERM	6	24	105.5	628.2	323.0	147.8	284.7	209.6	416.5
DSLNT	PRE-TERM	7	24	100.2	677.8	306.1	157.3	277.3	185.3	366.9
DSLNT	PRE-TERM	8	24	97.12	530.6	271.9	120.3	249.8	184.5	361.7
DSLNT	PRE-TERM	10	23	84.32	435.1	222.5	98.47	191.0	159.3	256.2
DSLNT	PRE-TERM	12	21	72.56	452.7	209.6	90.96	175.2	155.6	272.7
DSLNT	PRE-TERM	14	21	97.17	321.5	185.0	67.76	169.3	132.9	235.7
DSLNT	PRE-TERM	16	19	88.23	459.9	185.4	83.12	173.3	132.6	215.7
DSLNT	TERM	1	28	163.6	697.9	361.2	126.2	338.8	271.7	418.0
DSLNT	TERM	2	26	17.50	880.1	360.4	172.1	354.1	252.8	440.0

Table S1 Concentration of Human Milk Oligosaccharides in Term or Preterm Milk At Different Weeks Postpartum

** When there are results below the method limit of quanfification (LoQ) the result has been assigned value of 0.5 × LoQ, hence the minimum value appears to be the same in many cases. When a large number of datapoints are below LoQ this can also have the effect that the median = minimum.*

				HMO Concentration (mg/L)						
**HMO**	**Study Arm**	**Week Post Partum**	**N**	**min ***	**max**	**mean**	**sd**	**median ***	**Quartile 1 (25%)**	**Quartile 3 (75%)**
DSLNT	TERM	3	28	117.1	836.5	363.1	141.9	360.0	257.8	421.3
DSLNT	TERM	4	28	94.18	630.2	305.7	115.8	324.5	224.4	364.2
DSLNT	TERM	5	28	75.20	555.7	251.2	101.8	251.2	171.1	311.0
DSLNT	TERM	6	27	48.35	426.1	231.5	100.0	218.2	165.3	308.3
DSLNT	TERM	7	27	48.04	437.5	202.9	91.41	178.1	138.9	279.7
DSLNT	TERM	8	28	60.14	422.7	172.9	77.97	166.7	117.0	216.0
LDFT	PRE-TERM	1	25	22.50	1174	345.6	306.9	239.0	56.66	529.4
LDFT	PRE-TERM	2	25	22.50	759.0	202.0	188.8	146.1	69.93	265.3
LDFT	PRE-TERM	3	25	22.50	1926	268.6	419.8	164.7	89.82	255.1
LDFT	PRE-TERM	4	24	22.50	2513	302.9	504.1	177.7	52.94	334.3
LDFT	PRE-TERM	5	25	22.50	2072	294.0	420.2	212.7	72.07	337.6
LDFT	PRE-TERM	6	24	22.50	987.2	286.2	275.8	215.8	76.99	367.5
LDFT	PRE-TERM	7	24	22.50	2758	291.6	543.3	173.8	63.75	258.4
LDFT	PRE-TERM	8	24	22.50	2333	353.9	575.4	184.7	63.59	317.4
LDFT	PRE-TERM	10	23	22.50	1827	341.3	417.9	210.5	93.99	421.8
LDFT	PRE-TERM	12	21	22.50	3003	455.1	730.3	249.3	22.50	459.8
LDFT	PRE-TERM	14	21	22.50	1797	296.3	382.7	251.7	22.50	351.7
LDFT	PRE-TERM	16	19	22.50	1825	419.3	456.1	305.0	123.2	508.1
LDFT	TERM	1	28	22.50	983.9	340.4	286.8	314.3	101.5	487.9
LDFT	TERM	2	26	22.50	732.1	255.1	198.5	239.8	87.03	349.7
LDFT	TERM	3	28	22.50	965.0	258.1	232.9	217.8	124.7	332.7
LDFT	TERM	4	28	22.50	549.3	202.5	140.4	211.1	108.3	271.3
LDFT	TERM	5	28	22.50	1270	255.6	287.3	193.6	113.2	298.3
LDFT	TERM	6	27	22.50	2907	298.5	534.6	197.9	115.7	305.8
LDFT	TERM	7	27	22.50	1713	272.1	323.3	224.0	97.50	332.1
LDFT	TERM	8	28	22.50	818.6	248.3	192.7	225.4	112.0	334.3
LNDFH-I	PRE-TERM	1	25	5.000	1781	975.6	660.0	1145	115.7	1518
LNDFH-I	PRE-TERM	2	25	5.000	1629	807.5	559.0	892.9	75.44	1222
LNDFH-I	PRE-TERM	3	25	5.000	2269	869.3	655.7	970.1	56.94	1391

Table S1 Concentration of Human Milk Oligosaccharides in Term or Preterm Milk At Different Weeks Postpartum

** When there are results below the method limit of quanfification (LoQ) the result has been assigned value of 0.5 × LoQ, hence the minimum value appears to be the same in many cases. When a large number of datapoints are below LoQ this can also have the effect that the median = minimum.*

				HMO Concentration (mg/L)						
HMO	Study Arm	Week Post Partum	N	min *	max	mean	sd	median *	Quartile 1 (25%)	Quartile 3 (75%)
LNDFH-I	PRE-TERM	4	24	5.000	3123	871.2	751.9	919.2	36.13	1160
LNDFH-I	PRE-TERM	5	25	5.000	3161	907.7	764.2	1000	41.75	1220
LNDFH-I	PRE-TERM	6	24	5.000	1624	786.1	587.6	857.5	35.07	1239
LNDFH-I	PRE-TERM	7	24	5.000	1500	756.5	541.3	862.4	33.04	1178
LNDFH-I	PRE-TERM	8	24	5.000	1910	797.7	600.2	898.9	29.31	1179
LNDFH-I	PRE-TERM	10	23	5.000	1663	761.3	525.2	814.3	317.8	1105
LNDFH-I	PRE-TERM	12	21	5.000	1606	699.4	572.6	751.4	14.75	1159
LNDFH-I	PRE-TERM	14	21	5.000	1768	636.4	524.6	630.9	14.03	1048
LNDFH-I	PRE-TERM	16	19	5.000	1431	647.2	455.3	676.3	282.1	970.2
LNDFH-I	TERM	1	28	5.000	2122	943.1	652.9	990.0	620.7	1308
LNDFH-I	TERM	2	26	5.000	2264	987.3	704.9	1043	207.8	1484
LNDFH-I	TERM	3	28	5.000	2313	1025	700.3	1088	525.8	1495
LNDFH-I	TERM	4	28	5.000	1937	913.0	619.2	1068	410.4	1309
LNDFH-I	TERM	5	28	5.000	1838	841.2	595.7	949.4	351.1	1255
LNDFH-I	TERM	6	27	5.000	2024	826.1	616.9	958.9	231.9	1214
LNDFH-I	TERM	7	27	5.000	2167	792.5	602.2	824.2	194.8	1249
LNDFH-I	TERM	8	28	5.000	1935	669.2	548.9	748.8	5.000	964.3
LNFP-I	PRE-TERM	1	25	13.50	2806	1337	914.6	1424	773.5	2005
LNFP-I	PRE-TERM	2	25	13.50	2195	981.6	662.0	1027	654.5	1448
LNFP-I	PRE-TERM	3	25	13.50	2178	875.5	606.6	876.2	600.4	1307
LNFP-I	PRE-TERM	4	24	13.50	2062	762.1	546.9	878.9	439.5	1007
LNFP-I	PRE-TERM	5	25	13.50	1624	703.9	503.7	673.9	422.9	1093
LNFP-I	PRE-TERM	6	24	13.50	1840	625.2	486.8	657.3	229.8	934.6
LNFP-I	PRE-TERM	7	24	13.50	1773	568.1	441.9	576.6	265.2	843.4
LNFP-I	PRE-TERM	8	24	13.50	1123	529.3	366.9	541.0	295.2	800.4
LNFP-I	PRE-TERM	10	23	13.50	1436	465.7	340.5	443.9	316.2	656.9
LNFP-I	PRE-TERM	12	21	13.50	966.6	358.4	307.6	356.3	13.50	513.5
LNFP-I	PRE-TERM	14	21	13.50	1014	362.4	314.4	327.9	13.50	549.4
LNFP-I	PRE-TERM	16	19	13.50	933.6	334.1	283.6	273.0	145.5	540.5

Table S1 Concentration of Human Milk Oligosaccharides in Term or Preterm Milk At Different Weeks Postpartum

** When there are results below the method limit of quanfification (LoQ) the result has been assigned value of 0.5 × LoQ, hence the minimum value appears to be the same in many cases. When a large number of datapoints are below LoQ this can also have the effect that the median = minimum.*

				HMO Concentration (mg/L)						
**HMO**	**Study Arm**	**Week Post Partum**	**N**	**min ***	**max**	**mean**	**sd**	**median ***	**Quartile 1 (25%)**	**Quartile 3 (75%)**
LNFP-I	TERM	1	28	13.50	3597	1743	1125	2061	901.6	2431
LNFP-I	TERM	2	26	13.50	3073	1317	922.2	1454	635.8	1761
LNFP-I	TERM	3	28	13.50	3018	1214	854.0	1214	565.8	1803
LNFP-I	TERM	4	28	13.50	3156	1061	817.3	994.6	426.1	1665
LNFP-I	TERM	5	28	13.50	2655	821.6	648.2	879.1	307.0	1125
LNFP-I	TERM	6	27	13.50	2045	680.9	513.9	735.2	244.7	891.8
LNFP-I	TERM	7	27	13.50	2617	642.1	584.9	646.1	192.7	796.0
LNFP-I	TERM	8	28	13.50	1956	561.7	497.9	481.2	192.2	774.6
LNFP-II	PRE-TERM	1	25	17.50	2094	542.4	594.0	226.1	146.0	576.0
LNFP-II	PRE-TERM	2	25	17.50	1950	616.0	591.1	326.3	191.5	828.9
LNFP-II	PRE-TERM	3	25	17.50	1922	625.8	559.0	372.4	258.2	812.2
LNFP-II	PRE-TERM	4	24	17.50	2220	620.0	561.9	401.7	245.9	819.9
LNFP-II	PRE-TERM	5	25	17.50	1775	586.0	477.3	469.5	230.0	761.0
LNFP-II	PRE-TERM	6	24	17.50	2138	602.6	589.7	393.9	190.9	805.7
LNFP-II	PRE-TERM	7	24	17.50	2116	618.3	540.9	513.2	207.1	796.6
LNFP-II	PRE-TERM	8	24	17.50	1804	562.8	445.8	464.1	219.5	770.7
LNFP-II	PRE-TERM	10	23	17.50	1432	474.9	350.3	385.2	216.0	659.2
LNFP-II	PRE-TERM	12	21	17.50	2373	564.2	525.0	510.0	249.7	633.7
LNFP-II	PRE-TERM	14	21	17.50	1476	502.6	371.7	427.7	281.3	601.3
LNFP-II	PRE-TERM	16	19	17.50	2593	516.3	583.7	378.9	218.0	522.9
LNFP-II	TERM	1	28	17.50	1616	415.3	472.5	229.4	121.7	345.1
LNFP-II	TERM	2	26	17.50	1884	523.0	508.7	326.9	210.0	622.0
LNFP-II	TERM	3	28	17.50	1672	507.3	473.6	343.1	214.6	653.9
LNFP-II	TERM	4	28	17.50	1565	531.3	461.9	336.3	243.4	673.9
LNFP-II	TERM	5	28	17.50	1411	456.2	385.9	349.5	183.8	530.3
LNFP-II	TERM	6	27	17.50	1476	438.3	360.8	357.0	199.0	510.1
LNFP-II	TERM	7	27	17.50	1171	422.7	312.8	316.3	199.8	560.0
LNFP-II	TERM	8	28	17.50	1272	402.5	338.4	309.9	171.8	564.5
LNFP-III	PRE-TERM	1	25	55.72	713.1	346.2	160.2	347.4	236.0	404.4

Table S1 Concentration of Human Milk Oligosaccharides in Term or Preterm Milk At Different Weeks Postpartum

** When there are results below the method limit of quanfification (LoQ) the result has been assigned value of 0.5 × LoQ, hence the minimum value appears to be the same in many cases. When a large number of datapoints are below LoQ this can also have the effect that the median = minimum.*

				HMO Concentration (mg/L)						
**HMO**	**Study Arm**	**Week Post Partum**	**N**	**min ***	**max**	**mean**	**sd**	**median ***	**Quartile 1 (25%)**	**Quartile 3 (75%)**
LNFP-III	PRE-TERM	2	25	35.70	779.2	317.1	174.4	297.5	188.2	364.3
LNFP-III	PRE-TERM	3	25	40.94	733.6	307.8	160.6	279.9	211.2	397.0
LNFP-III	PRE-TERM	4	24	49.93	727.3	302.9	143.9	294.9	234.5	360.3
LNFP-III	PRE-TERM	5	25	52.14	742.0	321.0	145.9	316.3	232.4	402.6
LNFP-III	PRE-TERM	6	24	49.25	543.6	328.0	137.8	344.8	218.2	433.7
LNFP-III	PRE-TERM	7	24	17.50	711.8	336.7	159.4	331.3	241.4	397.1
LNFP-III	PRE-TERM	8	24	52.97	847.4	357.1	179.4	308.5	249.6	416.0
LNFP-III	PRE-TERM	10	23	60.20	707.5	350.2	147.2	329.7	271.7	427.3
LNFP-III	PRE-TERM	12	21	50.94	653.0	389.0	163.8	385.3	259.4	472.4
LNFP-III	PRE-TERM	14	21	74.13	750.3	368.5	166.3	352.7	233.2	498.0
LNFP-III	PRE-TERM	16	19	89.27	774.0	343.3	166.9	338.5	231.1	395.1
LNFP-III	TERM	1	28	76.48	1268	420.6	227.8	382.0	314.1	475.1
LNFP-III	TERM	2	26	163.3	1169	370.8	194.3	320.7	266.9	432.4
LNFP-III	TERM	3	28	153.7	911.0	305.1	142.8	286.4	224.1	337.9
LNFP-III	TERM	4	28	153.3	823.9	296.8	139.4	256.1	215.7	348.6
LNFP-III	TERM	5	28	165.5	714.0	301.5	119.7	270.4	227.6	346.7
LNFP-III	TERM	6	27	17.50	786.1	322.5	151.1	308.5	212.8	395.0
LNFP-III	TERM	7	27	190.9	722.3	331.6	136.1	299.0	238.4	362.7
LNFP-III	TERM	8	28	108.0	569.7	318.6	109.8	300.5	253.3	377.2
LNFP-V	PRE-TERM	1	25	12.00	320.9	75.84	97.85	30.53	12.00	58.18
LNFP-V	PRE-TERM	2	25	12.00	317.8	85.10	99.20	43.27	12.00	92.28
LNFP-V	PRE-TERM	3	25	12.00	309.0	90.49	95.38	48.72	26.19	95.79
LNFP-V	PRE-TERM	4	24	12.00	363.8	90.01	98.88	52.31	25.15	107.9
LNFP-V	PRE-TERM	5	25	12.00	301.0	84.27	81.58	64.56	25.18	94.75
LNFP-V	PRE-TERM	6	24	12.00	359.5	92.44	99.90	57.93	24.20	112.5
LNFP-V	PRE-TERM	7	24	12.00	321.2	88.74	88.39	64.66	21.81	105.4
LNFP-V	PRE-TERM	8	24	12.00	260.0	79.40	69.93	60.23	27.32	108.3
LNFP-V	PRE-TERM	10	23	12.00	182.5	61.18	52.12	44.56	12.00	85.80
LNFP-V	PRE-TERM	12	21	12.00	328.8	72.40	73.90	60.76	26.89	80.43

Table S1 Concentration of Human Milk Oligosaccharides in Term or Preterm Milk At Different Weeks Postpartum

** When there are results below the method limit of quanfification (LoQ) the result has been assigned value of 0.5 × LoQ, hence the minimum value appears to be the same in many cases. When a large number of datapoints are below LoQ this can also have the effect that the median = minimum.*

				HMO Concentration (mg/L)						
**HMO**	**Study Arm**	**Week Post Partum**	**N**	**min ***	**max**	**mean**	**sd**	**median ***	**Quartile 1 (25%)**	**Quartile 3 (75%)**
LNFP-V	PRE-TERM	14	21	12.00	202.6	67.25	54.22	53.26	28.01	85.62
LNFP-V	PRE-TERM	16	19	12.00	408.7	68.82	92.08	40.12	27.85	62.47
LNFP-V	TERM	1	28	12.00	203.7	58.55	64.01	28.29	12.00	68.00
LNFP-V	TERM	2	26	12.00	268.1	75.17	75.12	48.35	25.88	72.19
LNFP-V	TERM	3	28	12.00	349.1	81.09	83.30	55.34	31.19	88.38
LNFP-V	TERM	4	28	12.00	202.6	67.55	60.03	43.41	27.20	72.89
LNFP-V	TERM	5	28	12.00	228.2	64.93	56.04	52.87	26.25	77.17
LNFP-V	TERM	6	27	12.00	226.4	63.81	52.32	49.38	29.57	82.53
LNFP-V	TERM	7	27	12.00	162.9	57.73	41.46	56.51	28.73	83.85
LNFP-V	TERM	8	28	12.00	196.7	53.36	47.74	40.27	12.00	77.09
LNnDFH	PRE-TERM	1	25	14.00	543.1	76.02	129.9	29.06	14.00	65.59
LNnDFH	PRE-TERM	2	25	14.00	299.7	43.36	65.62	14.00	14.00	37.78
LNnDFH	PRE-TERM	3	25	14.00	333.0	42.54	71.15	14.00	14.00	40.63
LNnDFH	PRE-TERM	4	24	14.00	195.3	33.73	43.93	14.00	14.00	21.17
LNnDFH	PRE-TERM	5	25	14.00	186.3	50.19	56.12	14.00	14.00	67.69
LNnDFH	PRE-TERM	6	24	14.00	167.1	37.55	39.51	14.00	14.00	39.95
LNnDFH	PRE-TERM	7	24	14.00	184.6	28.69	36.84	14.00	14.00	19.35
LNnDFH	PRE-TERM	8	24	14.00	156.3	34.93	38.53	14.00	14.00	30.21
LNnDFH	PRE-TERM	10	23	14.00	119.8	33.37	28.06	29.34	14.00	35.68
LNnDFH	PRE-TERM	12	21	14.00	115.8	39.61	31.74	14.00	14.00	67.08
LNnDFH	PRE-TERM	14	21	14.00	733.1	67.62	155.3	14.00	14.00	54.35
LNnDFH	PRE-TERM	16	19	14.00	109.1	40.20	32.35	30.74	14.00	51.30
LNnDFH	TERM	1	28	14.00	181.8	51.69	47.26	33.17	14.00	71.98
LNnDFH	TERM	2	26	14.00	107.6	31.69	25.56	14.00	14.00	45.90
LNnDFH	TERM	3	28	14.00	77.46	23.79	15.98	14.00	14.00	31.02
LNnDFH	TERM	4	28	14.00	42.02	19.38	9.758	14.00	14.00	17.79
LNnDFH	TERM	5	28	14.00	132.3	23.26	23.73	14.00	14.00	17.69
LNnDFH	TERM	6	27	14.00	98.17	31.78	27.37	14.00	14.00	44.89
LNnDFH	TERM	7	27	14.00	94.89	26.79	22.32	14.00	14.00	30.46

Table S1 Concentration of Human Milk Oligosaccharides in Term or Preterm Milk At Different Weeks Postpartum

** When there are results below the method limit of quanfification (LoQ) the result has been assigned value of 0.5 × LoQ, hence the minimum value appears to be the same in many cases. When a large number of datapoints are below LoQ this can also have the effect that the median = minimum.*

				HMO Concentration (mg/L)						
**HMO**	**Study Arm**	**Week Post Partum**	**N**	**min ***	**max**	**mean**	**sd**	**median ***	**Quartile 1 (25%)**	**Quartile 3 (75%)**
LNnDFH	TERM	8	28	14.00	94.07	25.98	23.65	14.00	14.00	30.37
LNnFP	PRE-TERM	1	25	9.500	89.76	18.66	17.60	9.500	9.500	25.09
LNnFP	PRE-TERM	2	25	9.500	50.62	15.70	10.02	9.500	9.500	21.61
LNnFP	PRE-TERM	3	25	9.500	50.34	14.95	10.76	9.500	9.500	19.18
LNnFP	PRE-TERM	4	24	9.500	59.82	15.23	13.45	9.500	9.500	9.500
LNnFP	PRE-TERM	5	25	9.500	61.67	15.23	12.87	9.500	9.500	9.500
LNnFP	PRE-TERM	6	24	9.500	60.47	14.67	11.70	9.500	9.500	12.13
LNnFP	PRE-TERM	7	24	9.500	55.24	14.16	10.88	9.500	9.500	9.500
LNnFP	PRE-TERM	8	24	9.500	57.65	15.24	13.45	9.500	9.500	11.88
LNnFP	PRE-TERM	10	23	9.500	60.19	15.78	13.58	9.500	9.500	9.500
LNnFP	PRE-TERM	12	21	9.500	53.23	14.96	12.16	9.500	9.500	9.500
LNnFP	PRE-TERM	14	21	9.500	70.04	15.47	15.66	9.500	9.500	9.500
LNnFP	PRE-TERM	16	19	9.500	88.05	16.84	20.45	9.500	9.500	9.500
LNnFP	TERM	1	28	9.500	56.56	17.07	14.28	9.500	9.500	20.33
LNnFP	TERM	2	26	9.500	38.38	14.49	9.620	9.500	9.500	9.500
LNnFP	TERM	3	28	9.500	49.35	13.55	8.781	9.500	9.500	11.97
LNnFP	TERM	4	28	9.500	66.17	14.45	12.37	9.500	9.500	9.500
LNnFP	TERM	5	28	9.500	52.21	13.47	9.429	9.500	9.500	9.500
LNnFP	TERM	6	27	9.500	56.13	14.81	10.03	9.500	9.500	19.40
LNnFP	TERM	7	27	9.500	37.85	13.88	7.109	9.500	9.500	19.21
LNnFP	TERM	8	28	9.500	28.50	13.31	6.325	9.500	9.500	20.40
LNnT	PRE-TERM	1	25	90.25	546.3	274.1	122.2	250.6	186.6	381.7
LNnT	PRE-TERM	2	25	96.36	380.7	222.6	89.83	222.0	147.3	292.5
LNnT	PRE-TERM	3	25	66.51	353.8	189.8	77.97	192.5	127.6	232.0
LNnT	PRE-TERM	4	24	63.43	349.0	178.4	84.85	179.7	105.5	233.0
LNnT	PRE-TERM	5	25	43.09	383.2	158.4	91.21	140.5	88.14	225.3
LNnT	PRE-TERM	6	24	12.00	397.8	168.9	99.48	152.7	91.30	232.2
LNnT	PRE-TERM	7	24	35.89	314.7	163.0	84.18	166.7	90.65	214.9
LNnT	PRE-TERM	8	24	33.45	440.8	159.0	92.27	161.2	92.85	202.4

Table S1 Concentration of Human Milk Oligosaccharides in Term or Preterm Milk At Different Weeks Postpartum

** When there are results below the method limit of quanfification (LoQ) the result has been assigned value of 0.5 × LoQ, hence the minimum value appears to be the same in many cases. When a large number of datapoints are below LoQ this can also have the effect that the median = minimum.*

				HMO Concentration (mg/L)						
HMO	Study Arm	Week Post Partum	N	min *	max	mean	sd	median *	Quartile 1 (25%)	Quartile 3 (75%)
LNnT	PRE-TERM	10	23	24.44	464.0	154.7	106.3	128.3	78.35	202.8
LNnT	PRE-TERM	12	21	12.00	379.4	122.4	86.34	104.6	66.88	162.7
LNnT	PRE-TERM	14	21	12.00	444.3	125.3	100.1	86.79	68.35	149.4
LNnT	PRE-TERM	16	19	25.55	437.3	126.2	103.5	90.89	54.01	166.0
LNnT	TERM	1	28	187.1	565.3	333.4	94.38	341.0	257.0	399.8
LNnT	TERM	2	26	113.9	475.4	227.4	95.75	227.1	149.5	264.0
LNnT	TERM	3	28	67.58	385.9	182.3	81.57	171.6	114.7	222.7
LNnT	TERM	4	28	52.63	350.3	165.8	76.29	150.4	115.4	196.6
LNnT	TERM	5	28	12.00	369.5	152.7	84.04	138.1	101.7	186.4
LNnT	TERM	6	27	12.00	311.2	144.4	74.93	136.2	84.46	187.7
LNnT	TERM	7	27	33.31	270.0	138.1	65.19	137.7	81.85	173.3
LNnT	TERM	8	28	27.23	284.9	132.8	65.28	121.3	85.20	164.4
LNT	PRE-TERM	1	25	298.8	3029	1179	673.8	912.8	803.4	1684
LNT	PRE-TERM	2	25	644.9	3296	1522	725.9	1186	965.1	1821
LNT	PRE-TERM	3	25	510.5	2903	1448	627.8	1245	997.4	1715
LNT	PRE-TERM	4	24	469.1	2737	1319	633.2	1156	892.1	1862
LNT	PRE-TERM	5	25	356.6	2468	1162	541.6	1119	712.6	1506
LNT	PRE-TERM	6	24	144.1	2428	1158	655.4	1049	583.8	1649
LNT	PRE-TERM	7	24	312.8	2376	1120	592.7	1078	592.9	1499
LNT	PRE-TERM	8	24	294.8	2184	995.9	503.2	961.9	601.9	1268
LNT	PRE-TERM	10	23	223.6	1829	796.7	392.3	704.7	528.9	1015
LNT	PRE-TERM	12	21	175.3	1554	754.7	362.0	763.9	588.8	1013
LNT	PRE-TERM	14	21	232.8	1191	669.1	289.9	675.4	447.8	827.3
LNT	PRE-TERM	16	19	145.2	2113	660.3	438.9	604.4	391.5	824.2
LNT	TERM	1	28	199.8	3400	993.4	742.0	737.9	570.5	1164
LNT	TERM	2	26	358.7	3848	1334	660.5	1214	970.3	1474
LNT	TERM	3	28	447.8	3092	1286	576.3	1272	891.8	1536
LNT	TERM	4	28	384.6	3087	1216	584.5	1207	768.9	1370
LNT	TERM	5	28	307.2	2480	1056	586.1	937.8	639.4	1269

Table S1 Concentration of Human Milk Oligosaccharides in Term or Preterm Milk At Different Weeks Postpartum

** When there are results below the method limit of quanfification (LoQ) the result has been assigned value of 0.5 × LoQ, hence the minimum value appears to be the same in many cases. When a large number of datapoints are below LoQ this can also have the effect that the median = minimum.*

				HMO Concentration (mg/L)						
**HMO**	**Study Arm**	**Week Post Partum**	**N**	**min ***	**max**	**mean**	**sd**	**median ***	**Quartile 1 (25%)**	**Quartile 3 (75%)**
LNT	TERM	6	27	280.7	2109	938.5	513.8	843.1	561.9	1270
LNT	TERM	7	27	217.7	2251	877.3	460.2	813.3	529.7	1152
LNT	TERM	8	28	213.7	1852	803.9	468.0	753.0	431.3	1029
LSTb	PRE-TERM	1	25	44.25	219.5	100.4	47.32	88.16	63.18	125.0
LSTb	PRE-TERM	2	25	41.49	211.3	110.9	44.94	100.1	79.15	151.4
LSTb	PRE-TERM	3	25	45.44	217.4	117.8	47.69	110.5	84.13	151.3
LSTb	PRE-TERM	4	24	47.34	256.1	112.6	50.84	107.4	74.61	136.3
LSTb	PRE-TERM	5	25	42.09	334.7	115.8	63.93	106.0	86.22	134.2
LSTb	PRE-TERM	6	24	37.86	314.7	110.7	58.66	118.1	64.81	135.3
LSTb	PRE-TERM	7	24	36.96	423.9	107.2	76.22	96.84	61.79	125.7
LSTb	PRE-TERM	8	24	7.000	400.7	102.0	76.47	87.16	54.05	123.8
LSTb	PRE-TERM	10	23	34.87	195.0	85.69	41.71	77.14	50.40	111.9
LSTb	PRE-TERM	12	21	31.54	405.6	94.92	80.22	80.04	49.06	104.5
LSTb	PRE-TERM	14	21	25.18	243.3	83.89	43.96	78.78	62.11	94.78
LSTb	PRE-TERM	16	19	23.33	247.3	78.39	46.98	75.40	52.56	91.57
LSTb	TERM	1	28	15.52	196.6	81.57	41.01	71.90	50.83	98.18
LSTb	TERM	2	26	28.89	160.5	75.69	31.50	67.04	52.67	92.92
LSTb	TERM	3	28	36.75	163.7	82.69	31.37	81.26	55.62	109.3
LSTb	TERM	4	28	20.15	136.8	80.44	31.01	79.26	57.63	106.6
LSTb	TERM	5	28	21.68	166.6	76.87	33.05	70.04	53.87	96.73
LSTb	TERM	6	27	7.000	147.4	73.95	39.28	73.21	45.75	98.08
LSTb	TERM	7	27	17.27	177.0	76.51	38.73	71.49	45.40	102.9
LSTb	TERM	8	28	7.000	152.6	66.24	35.92	60.43	44.76	93.16
LSTc	PRE-TERM	1	25	95.01	1019	432.6	254.8	439.1	184.0	605.1
LSTc	PRE-TERM	2	25	37.98	629.5	274.7	153.5	248.2	176.2	378.9
LSTc	PRE-TERM	3	25	31.79	534.2	208.0	125.3	183.5	121.2	274.7
LSTc	PRE-TERM	4	24	28.75	622.9	178.3	133.6	123.7	96.69	230.8
LSTc	PRE-TERM	5	25	24.49	305.6	128.1	75.25	119.0	64.51	162.3
LSTc	PRE-TERM	6	24	20.21	345.5	126.1	80.54	106.3	71.02	182.7

Table S1 Concentration of Human Milk Oligosaccharides in Term or Preterm Milk At Different Weeks Postpartum

** When there are results below the method limit of quanfification (LoQ) the result has been assigned value of 0.5 × LoQ, hence the minimum value appears to be the same in many cases. When a large number of datapoints are below LoQ this can also have the effect that the median = minimum.*

				HMO Concentration (mg/L)						
**HMO**	**Study Arm**	**Week Post Partum**	**N**	**min ***	**max**	**mean**	**sd**	**median ***	**Quartile 1 (25%)**	**Quartile 3 (75%)**
LSTc	PRE-TERM	7	24	17.24	371.1	105.7	76.80	84.04	56.32	154.0
LSTc	PRE-TERM	8	24	17.82	355.6	95.58	72.49	77.71	54.36	121.6
LSTc	PRE-TERM	10	23	24.55	209.1	75.71	45.34	62.11	46.86	87.28
LSTc	PRE-TERM	12	21	20.28	125.3	54.96	27.90	48.35	34.47	79.20
LSTc	PRE-TERM	14	21	10.91	102.5	40.31	26.27	32.26	24.47	53.72
LSTc	PRE-TERM	16	19	9.258	96.78	36.68	26.72	22.43	17.96	55.87
LSTc	TERM	1	28	36.80	1199	578.2	266.1	526.1	390.4	748.1
LSTc	TERM	2	26	204.1	1169	481.0	267.3	395.1	321.7	499.1
LSTc	TERM	3	28	103.3	755.6	306.0	162.3	233.6	191.5	392.1
LSTc	TERM	4	28	64.34	524.8	215.6	109.2	199.2	131.6	297.1
LSTc	TERM	5	28	32.73	367.7	157.6	76.72	141.3	97.24	196.6
LSTc	TERM	6	27	30.19	267.6	132.0	59.12	125.0	92.28	173.9
LSTc	TERM	7	27	20.07	271.5	107.6	64.41	96.05	65.56	127.3
LSTc	TERM	8	28	16.92	254.8	89.79	57.66	66.11	53.06	117.7
MFLNH-III	PRE-TERM	1	25	70.38	548.6	254.5	131.4	221.4	166.0	325.6
MFLNH-III	PRE-TERM	2	25	58.75	714.1	373.4	161.5	346.8	278.5	488.6
MFLNH-III	PRE-TERM	3	25	64.26	790.5	379.0	180.0	355.5	271.3	461.7
MFLNH-III	PRE-TERM	4	24	65.81	743.2	356.1	173.2	338.3	240.4	454.5
MFLNH-III	PRE-TERM	5	25	57.17	820.2	289.6	159.9	257.9	201.3	385.5
MFLNH-III	PRE-TERM	6	24	87.89	856.2	286.8	169.6	248.7	189.0	351.5
MFLNH-III	PRE-TERM	7	24	77.34	745.1	268.8	155.3	248.1	159.8	347.5
MFLNH-III	PRE-TERM	8	24	64.97	753.0	245.6	153.2	260.1	146.3	300.6
MFLNH-III	PRE-TERM	10	23	53.68	707.1	188.3	139.3	150.6	105.1	246.1
MFLNH-III	PRE-TERM	12	21	17.50	551.6	144.3	120.3	117.7	77.56	165.5
MFLNH-III	PRE-TERM	14	21	17.50	386.3	120.4	91.85	96.61	64.53	126.7
MFLNH-III	PRE-TERM	16	19	17.50	337.9	105.8	80.24	81.30	53.19	116.4
MFLNH-III	TERM	1	28	17.50	524.0	201.4	131.6	164.1	133.1	288.9
MFLNH-III	TERM	2	26	180.0	772.4	425.3	166.9	369.6	320.9	538.7
MFLNH-III	TERM	3	28	166.7	934.4	443.2	203.0	374.6	316.9	572.9

Table S1 Concentration of Human Milk Oligosaccharides in Term or Preterm Milk At Different Weeks Postpartum

** When there are results below the method limit of quanfification (LoQ) the result has been assigned value of 0.5 × LoQ, hence the minimum value appears to be the same in many cases. When a large number of datapoints are below LoQ this can also have the effect that the median = minimum.*

				HMO Concentration (mg/L)						
**HMO**	**Study Arm**	**Week Post Partum**	**N**	**min ***	**max**	**mean**	**sd**	**median ***	**Quartile 1 (25%)**	**Quartile 3 (75%)**
MFLNH-III	TERM	4	28	121.1	976.4	432.7	219.8	380.4	310.0	487.0
MFLNH-III	TERM	5	28	81.47	886.4	370.5	210.7	312.8	250.3	440.1
MFLNH-III	TERM	6	27	17.50	758.8	300.0	138.8	284.8	223.1	339.5
MFLNH-III	TERM	7	27	17.50	600.2	249.6	144.7	206.4	149.5	302.2
MFLNH-III	TERM	8	28	17.50	875.6	246.8	166.4	193.6	149.2	307.4
