# Supplementary material for: Human Milk Oligosaccharides in the Milk of Mothers Delivering Term versus Preterm Infants
Source: Nutrients. 2019 Jun 5;11(6):1282. doi: 10.3390/nu11061282 (PMC6627155; doi:10.3390/nu11061282)
Supplement: Supplementary file 1 [file nutrients-11-01282-s001.zip › nutrients-512673-supplementary/Supp_Table_S2_HMO_PP_Milk_Group.pdf]

Table S2 Concentration of Human Milk Oligosaccharides in Term or Preterm Milk At Different Weeks Postpartum Separated By Milk Group

** When there are results below the method limit of quanfification (LoQ) the result has been assigned value of 0.5 × LoQ, hence the minimum value appears to be the same in many cases. When a large number of datapoints are below LoQ this can also have the effect that the median = minimum.*

					HMO Concentration (mg/L)						
HMO	Milk Group	Study Arm	Week Post Partum	N	min *	max	mean	sd	median *	Quartile 1 (25%)	Quartile 3 (75%)
2'FL	G1	PRE-TERM	1	19	53.00	5478	3113	1192	3370	2384	3878
2'FL	G1	PRE-TERM	2	19	68.21	3095	2015	782.8	2047	1678	2622
2'FL	G1	PRE-TERM	3	19	40.96	2995	1927	763.8	1958	1589	2569
2'FL	G1	PRE-TERM	4	18	35.03	3592	2046	881.8	2132	1591	2507
2'FL	G1	PRE-TERM	5	19	37.54	3063	1937	719.5	1852	1634	2470
2'FL	G1	PRE-TERM	6	18	32.13	3335	1930	866.6	1890	1577	2568
2'FL	G1	PRE-TERM	7	18	30.17	2969	1898	749.0	1833	1524	2558
2'FL	G1	PRE-TERM	8	18	27.93	3353	1944	832.2	1741	1505	2487
2'FL	G1	PRE-TERM	10	18	22.31	3369	1794	808.6	1588	1328	2370
2'FL	G1	PRE-TERM	12	15	19.65	3574	1686	849.5	1529	1270	1999
2'FL	G1	PRE-TERM	14	15	32.90	2946	1663	827.5	1464	1159	2138
2'FL	G1	PRE-TERM	16	15	30.28	3455	1622	867.5	1569	962.3	2149
2'FL	G1	TERM	1	21	2571	5570	3917	956.8	3701	3186	4731
2'FL	G1	TERM	2	19	1676	3664	2724	561.0	2838	2259	3143
2'FL	G1	TERM	3	21	1518	4225	2641	705.2	2551	2140	2945
2'FL	G1	TERM	4	21	1387	3711	2457	609.8	2461	2085	2888
2'FL	G1	TERM	5	21	1325	3962	2387	732.6	2111	1752	2989
2'FL	G1	TERM	6	20	1028	3323	2246	720.9	2313	1716	3018
2'FL	G1	TERM	7	20	622.0	3067	2068	733.8	1986	1575	2622
2'FL	G1	TERM	8	21	241.7	3201	1963	810.1	2102	1399	2701
2'FL	G2	PRE-TERM	1	5	6.500	47.07	25.87	16.25	26.98	13.76	35.03
2'FL	G2	PRE-TERM	2	5	6.500	21.88	9.576	6.879	6.500	6.500	6.500
2'FL	G2	PRE-TERM	3	5	6.500	18.12	8.82	5.196	6.500	6.500	6.500
2'FL	G2	PRE-TERM	4	5	6.500	116.4	28.48	49.14	6.500	6.500	6.500
2'FL	G2	PRE-TERM	5	5	6.500	48.64	16.27	18.33	6.500	6.500	13.19
2'FL	G2	PRE-TERM	6	5	6.500	30.75	11.35	10.84	6.500	6.500	6.500
2'FL	G2	PRE-TERM	7	5	6.500	26.23	10.45	8.824	6.500	6.500	6.500
2'FL	G2	PRE-TERM	8	5	6.500	22.44	9.689	7.131	6.500	6.500	6.500
2'FL	G2	PRE-TERM	10	4	6.500	20.67	10.04	7.083	6.500	6.500	10.04
2'FL	G2	PRE-TERM	12	5	6.500	18.86	8.972	5.528	6.500	6.500	6.500
2'FL	G2	PRE-TERM	14	5	6.500	16.58	8.517	4.509	6.500	6.500	6.500
2'FL	G2	PRE-TERM	16	3	6.500	15.22	9.406	5.034	6.500	6.500	10.86
2'FL	G2	TERM	1	5	6.500	25.68	10.34	8.578	6.500	6.500	6.500
2'FL	G2	TERM	2	5	6.500	6.500	6.500	0.0000	6.500	6.500	6.500

Table S2 Concentration of Human Milk Oligosaccharides in Term or Preterm Milk At Different Weeks Postpartum Separated By Milk Group

** When there are results below the method limit of quanfification (LoQ) the result has been assigned value of 0.5 × LoQ, hence the minimum value appears to be the same in many cases. When a large number of datapoints are below LoQ this can also have the effect that the median = minimum.*

					HMO Concentration (mg/L)						
HMO	Milk Group	Study Arm	Week Post Partum	N	min *	max	mean	sd	median *	Quartile 1 (25%)	Quartile 3 (75%)
2'FL	G2	TERM	3	5	6.500	6.500	6.500	0.0000	6.500	6.500	6.500
2'FL	G2	TERM	4	5	6.500	14.11	8.023	3.405	6.500	6.500	6.500
2'FL	G2	TERM	5	5	6.500	6.500	6.500	0.0000	6.500	6.500	6.500
2'FL	G2	TERM	6	5	6.500	6.500	6.500	0.0000	6.500	6.500	6.500
2'FL	G2	TERM	7	5	6.500	6.500	6.500	0.0000	6.500	6.500	6.500
2'FL	G2	TERM	8	5	6.500	6.500	6.500	0.0000	6.500	6.500	6.500
2'FL	G3	PRE-TERM	1	1	3314	3314	3314	NA	3314	3314	3314
2'FL	G3	PRE-TERM	2	1	3465	3465	3465	NA	3465	3465	3465
2'FL	G3	PRE-TERM	3	1	2900	2900	2900	NA	2900	2900	2900
2'FL	G3	PRE-TERM	4	1	2645	2645	2645	NA	2645	2645	2645
2'FL	G3	PRE-TERM	5	1	3001	3001	3001	NA	3001	3001	3001
2'FL	G3	PRE-TERM	6	1	3071	3071	3071	NA	3071	3071	3071
2'FL	G3	PRE-TERM	7	1	2571	2571	2571	NA	2571	2571	2571
2'FL	G3	PRE-TERM	8	1	2316	2316	2316	NA	2316	2316	2316
2'FL	G3	PRE-TERM	10	1	2139	2139	2139	NA	2139	2139	2139
2'FL	G3	PRE-TERM	12	1	2079	2079	2079	NA	2079	2079	2079
2'FL	G3	PRE-TERM	14	1	2189	2189	2189	NA	2189	2189	2189
2'FL	G3	PRE-TERM	16	1	2024	2024	2024	NA	2024	2024	2024
2'FL	G3	TERM	1	1	6076	6076	6076	NA	6076	6076	6076
2'FL	G3	TERM	2	1	5572	5572	5572	NA	5572	5572	5572
2'FL	G3	TERM	3	1	4390	4390	4390	NA	4390	4390	4390
2'FL	G3	TERM	4	1	4556	4556	4556	NA	4556	4556	4556
2'FL	G3	TERM	5	1	4090	4090	4090	NA	4090	4090	4090
2'FL	G3	TERM	6	1	3666	3666	3666	NA	3666	3666	3666
2'FL	G3	TERM	7	1	3530	3530	3530	NA	3530	3530	3530
2'FL	G3	TERM	8	1	4133	4133	4133	NA	4133	4133	4133
2'FL	G4	TERM	1	1	26.81	26.81	26.81	NA	26.81	26.81	26.81
2'FL	G4	TERM	2	1	16.16	16.16	16.16	NA	16.16	16.16	16.16
2'FL	G4	TERM	3	1	6.500	6.500	6.500	NA	6.500	6.500	6.500
2'FL	G4	TERM	4	1	6.500	6.500	6.500	NA	6.500	6.500	6.500
2'FL	G4	TERM	5	1	6.500	6.500	6.500	NA	6.500	6.500	6.500
2'FL	G4	TERM	6	1	6.500	6.500	6.500	NA	6.500	6.500	6.500
2'FL	G4	TERM	7	1	6.500	6.500	6.500	NA	6.500	6.500	6.500
2'FL	G4	TERM	8	1	6.500	6.500	6.500	NA	6.500	6.500	6.500

Table S2 Concentration of Human Milk Oligosaccharides in Term or Preterm Milk At Different Weeks Postpartum Separated By Milk Group

** When there are results below the method limit of quanfification (LoQ) the result has been assigned value of 0.5 × LoQ, hence the minimum value appears to be the same in many cases. When a large number of datapoints are below LoQ this can also have the effect that the median = minimum.*

					HMO Concentration (mg/L)						
HMO	Milk Group	Study Arm	Week Post Partum	N	min *	max	mean	sd	median *	Quartile 1 (25%)	Quartile 3 (75%)
3'GL	G1	PRE-TERM	1	19	4.000	28.82	11.65	8.458	10.11	4.000	15.28
3'GL	G1	PRE-TERM	2	19	4.000	20.70	6.310	4.559	4.000	4.000	6.308
3'GL	G1	PRE-TERM	3	19	4.000	21.56	6.059	4.692	4.000	4.000	4.000
3'GL	G1	PRE-TERM	4	18	4.000	30.06	6.107	6.277	4.000	4.000	4.000
3'GL	G1	PRE-TERM	5	19	4.000	37.74	6.783	7.762	4.000	4.000	6.015
3'GL	G1	PRE-TERM	6	18	4.000	70.00	9.943	15.79	4.000	4.000	7.968
3'GL	G1	PRE-TERM	7	18	4.000	23.04	6.942	5.753	4.000	4.000	7.291
3'GL	G1	PRE-TERM	8	18	4.000	21.24	6.859	5.261	4.000	4.000	7.090
3'GL	G1	PRE-TERM	10	18	4.000	21.49	5.979	4.894	4.000	4.000	4.000
3'GL	G1	PRE-TERM	12	15	4.000	15.76	6.895	4.405	4.000	4.000	10.47
3'GL	G1	PRE-TERM	14	15	4.000	15.21	5.885	3.511	4.000	4.000	6.445
3'GL	G1	PRE-TERM	16	15	4.000	12.84	5.268	2.769	4.000	4.000	4.000
3'GL	G1	TERM	1	21	4.000	20.76	11.59	5.024	11.79	8.823	14.33
3'GL	G1	TERM	2	19	4.000	19.07	7.104	4.628	4.000	4.000	10.78
3'GL	G1	TERM	3	21	4.000	17.67	7.013	4.025	4.000	4.000	9.627
3'GL	G1	TERM	4	21	4.000	16.81	5.832	3.382	4.000	4.000	8.211
3'GL	G1	TERM	5	21	4.000	33.42	6.908	7.269	4.000	4.000	4.000
3'GL	G1	TERM	6	20	4.000	16.35	4.990	3.148	4.000	4.000	4.000
3'GL	G1	TERM	7	20	4.000	14.81	5.018	2.733	4.000	4.000	4.000
3'GL	G1	TERM	8	21	4.000	20.73	5.663	4.503	4.000	4.000	4.000
3'GL	G2	PRE-TERM	1	5	4.000	21.02	13.37	6.565	12.56	11.32	17.98
3'GL	G2	PRE-TERM	2	5	4.000	11.13	6.268	3.273	4.000	4.000	8.204
3'GL	G2	PRE-TERM	3	5	4.000	9.894	5.179	2.636	4.000	4.000	4.000
3'GL	G2	PRE-TERM	4	5	4.000	4.000	4.000	0.0000	4.000	4.000	4.000
3'GL	G2	PRE-TERM	5	5	4.000	16.72	6.544	5.688	4.000	4.000	4.000
3'GL	G2	PRE-TERM	6	5	4.000	12.18	6.816	3.940	4.000	4.000	9.895
3'GL	G2	PRE-TERM	7	5	4.000	4.000	4.000	0.0000	4.000	4.000	4.000
3'GL	G2	PRE-TERM	8	5	4.000	9.118	5.826	2.531	4.000	4.000	8.011
3'GL	G2	PRE-TERM	10	4	4.000	11.78	5.945	3.890	4.000	4.000	5.945
3'GL	G2	PRE-TERM	12	5	4.000	4.000	4.000	0.0000	4.000	4.000	4.000
3'GL	G2	PRE-TERM	14	5	4.000	12.65	6.983	4.171	4.000	4.000	10.27
3'GL	G2	PRE-TERM	16	3	4.000	4.000	4.000	0.0000	4.000	4.000	4.000
3'GL	G2	TERM	1	5	9.509	21.33	15.56	4.788	14.96	12.76	19.25
3'GL	G2	TERM	2	5	4.000	10.91	5.382	3.091	4.000	4.000	4.000

Table S2 Concentration of Human Milk Oligosaccharides in Term or Preterm Milk At Different Weeks Postpartum Separated By Milk Group

** When there are results below the method limit of quanfification (LoQ) the result has been assigned value of 0.5 × LoQ, hence the minimum value appears to be the same in many cases. When a large number of datapoints are below LoQ this can also have the effect that the median = minimum.*

					HMO Concentration (mg/L)						
HMO	Milk Group	Study Arm	Week Post Partum	N	min *	max	mean	sd	median *	Quartile 1 (25%)	Quartile 3 (75%)
3'GL	G2	TERM	3	5	4.000	8.782	4.956	2.139	4.000	4.000	4.000
3'GL	G2	TERM	4	5	4.000	4.000	4.000	0.0000	4.000	4.000	4.000
3'GL	G2	TERM	5	5	4.000	4.000	4.000	0.0000	4.000	4.000	4.000
3'GL	G2	TERM	6	5	4.000	4.000	4.000	0.0000	4.000	4.000	4.000
3'GL	G2	TERM	7	5	4.000	4.000	4.000	0.0000	4.000	4.000	4.000
3'GL	G2	TERM	8	5	4.000	4.000	4.000	0.0000	4.000	4.000	4.000
3'GL	G3	PRE-TERM	1	1	4.000	4.000	4.000	NA	4.000	4.000	4.000
3'GL	G3	PRE-TERM	2	1	4.000	4.000	4.000	NA	4.000	4.000	4.000
3'GL	G3	PRE-TERM	3	1	4.000	4.000	4.000	NA	4.000	4.000	4.000
3'GL	G3	PRE-TERM	4	1	4.000	4.000	4.000	NA	4.000	4.000	4.000
3'GL	G3	PRE-TERM	5	1	4.000	4.000	4.000	NA	4.000	4.000	4.000
3'GL	G3	PRE-TERM	6	1	4.000	4.000	4.000	NA	4.000	4.000	4.000
3'GL	G3	PRE-TERM	7	1	4.000	4.000	4.000	NA	4.000	4.000	4.000
3'GL	G3	PRE-TERM	8	1	4.000	4.000	4.000	NA	4.000	4.000	4.000
3'GL	G3	PRE-TERM	10	1	4.000	4.000	4.000	NA	4.000	4.000	4.000
3'GL	G3	PRE-TERM	12	1	4.000	4.000	4.000	NA	4.000	4.000	4.000
3'GL	G3	PRE-TERM	14	1	4.000	4.000	4.000	NA	4.000	4.000	4.000
3'GL	G3	PRE-TERM	16	1	4.000	4.000	4.000	NA	4.000	4.000	4.000
3'GL	G3	TERM	1	1	8.864	8.864	8.864	NA	8.864	8.864	8.864
3'GL	G3	TERM	2	1	4.000	4.000	4.000	NA	4.000	4.000	4.000
3'GL	G3	TERM	3	1	4.000	4.000	4.000	NA	4.000	4.000	4.000
3'GL	G3	TERM	4	1	4.000	4.000	4.000	NA	4.000	4.000	4.000
3'GL	G3	TERM	5	1	4.000	4.000	4.000	NA	4.000	4.000	4.000
3'GL	G3	TERM	6	1	4.000	4.000	4.000	NA	4.000	4.000	4.000
3'GL	G3	TERM	7	1	4.000	4.000	4.000	NA	4.000	4.000	4.000
3'GL	G3	TERM	8	1	4.000	4.000	4.000	NA	4.000	4.000	4.000
3'GL	G4	TERM	1	1	32.97	32.97	32.97	NA	32.97	32.97	32.97
3'GL	G4	TERM	2	1	19.72	19.72	19.72	NA	19.72	19.72	19.72
3'GL	G4	TERM	3	1	10.00	10.00	10.00	NA	10.00	10.00	10.00
3'GL	G4	TERM	4	1	8.499	8.499	8.499	NA	8.499	8.499	8.499
3'GL	G4	TERM	5	1	4.000	4.000	4.000	NA	4.000	4.000	4.000
3'GL	G4	TERM	6	1	8.446	8.446	8.446	NA	8.446	8.446	8.446
3'GL	G4	TERM	7	1	4.000	4.000	4.000	NA	4.000	4.000	4.000
3'GL	G4	TERM	8	1	4.000	4.000	4.000	NA	4.000	4.000	4.000

Table S2 Concentration of Human Milk Oligosaccharides in Term or Preterm Milk At Different Weeks Postpartum Separated By Milk Group

** When there are results below the method limit of quanfification (LoQ) the result has been assigned value of 0.5 × LoQ, hence the minimum value appears to be the same in many cases. When a large number of datapoints are below LoQ this can also have the effect that the median = minimum.*

					HMO Concentration (mg/L)						
HMO	Milk Group	Study Arm	Week Post Partum	N	min *	max	mean	sd	median *	Quartile 1 (25%)	Quartile 3 (75%)
3'SL	G1	PRE-TERM	1	19	154.6	513.3	230.8	82.50	217.5	189.7	233.7
3'SL	G1	PRE-TERM	2	19	117.4	329.8	192.1	59.45	180.4	150.4	221.5
3'SL	G1	PRE-TERM	3	19	107.7	430.3	195.7	74.66	175.1	163.0	239.9
3'SL	G1	PRE-TERM	4	18	98.75	439.9	196.4	78.56	186.8	155.0	216.4
3'SL	G1	PRE-TERM	5	19	103.5	455.8	198.9	81.49	188.2	159.1	226.9
3'SL	G1	PRE-TERM	6	18	85.96	344.4	190.9	74.26	181.7	126.0	239.1
3'SL	G1	PRE-TERM	7	18	91.34	333.8	186.4	73.17	173.7	117.0	231.1
3'SL	G1	PRE-TERM	8	18	91.08	438.1	194.7	98.93	173.5	133.2	212.8
3'SL	G1	PRE-TERM	10	18	88.38	403.2	185.3	79.70	159.8	145.6	210.0
3'SL	G1	PRE-TERM	12	15	93.94	382.6	200.6	82.28	177.2	149.8	247.5
3'SL	G1	PRE-TERM	14	15	82.71	291.3	172.9	68.42	160.6	120.6	236.9
3'SL	G1	PRE-TERM	16	15	93.80	325.0	186.6	78.65	165.2	120.5	253.0
3'SL	G1	TERM	1	21	119.6	354.4	226.7	70.78	230.8	181.9	279.2
3'SL	G1	TERM	2	19	94.16	280.3	150.0	41.99	146.2	122.2	169.1
3'SL	G1	TERM	3	21	78.49	282.3	138.5	43.69	138.8	114.0	157.0
3'SL	G1	TERM	4	21	77.94	272.4	136.4	42.94	134.0	105.6	153.8
3'SL	G1	TERM	5	21	82.07	274.9	131.9	44.68	121.8	105.7	149.2
3'SL	G1	TERM	6	20	72.18	303.0	135.2	50.07	123.0	104.1	156.7
3'SL	G1	TERM	7	20	74.58	240.1	129.8	41.48	127.5	99.13	153.8
3'SL	G1	TERM	8	21	50.25	236.8	122.8	41.97	115.8	93.96	143.8
3'SL	G2	PRE-TERM	1	5	215.7	419.1	282.7	80.34	272.6	232.2	274.1
3'SL	G2	PRE-TERM	2	5	161.1	257.5	217.3	42.73	228.2	185.3	254.5
3'SL	G2	PRE-TERM	3	5	151.7	223.3	196.8	29.02	196.6	191.1	221.3
3'SL	G2	PRE-TERM	4	5	129.5	284.0	190.2	66.29	178.0	131.1	228.3
3'SL	G2	PRE-TERM	5	5	129.1	311.3	180.8	75.02	164.7	132.6	166.0
3'SL	G2	PRE-TERM	6	5	120.4	240.4	170.6	49.78	154.2	135.4	202.4
3'SL	G2	PRE-TERM	7	5	115.6	362.9	181.4	105.6	122.5	119.6	186.3
3'SL	G2	PRE-TERM	8	5	92.00	342.8	160.8	103.5	119.4	105.8	143.9
3'SL	G2	PRE-TERM	10	4	97.27	162.9	124.7	28.07	119.3	108.7	135.3
3'SL	G2	PRE-TERM	12	5	92.61	212.0	138.4	46.15	132.8	108.4	146.4
3'SL	G2	PRE-TERM	14	5	83.76	239.2	143.7	63.81	146.0	86.95	162.4
3'SL	G2	PRE-TERM	16	3	86.41	179.1	137.2	46.98	145.9	116.2	162.5
3'SL	G2	TERM	1	5	105.2	380.6	215.0	103.4	197.0	162.7	229.6
3'SL	G2	TERM	2	5	89.66	160.1	122.4	28.19	125.3	100.4	136.3

Table S2 Concentration of Human Milk Oligosaccharides in Term or Preterm Milk At Different Weeks Postpartum Separated By Milk Group

** When there are results below the method limit of quanfification (LoQ) the result has been assigned value of 0.5 × LoQ, hence the minimum value appears to be the same in many cases. When a large number of datapoints are below LoQ this can also have the effect that the median = minimum.*

					HMO Concentration (mg/L)						
HMO	Milk Group	Study Arm	Week Post Partum	N	min *	max	mean	sd	median *	Quartile 1 (25%)	Quartile 3 (75%)
3'SL	G2	TERM	3	5	94.04	136.4	114.3	18.84	118.5	95.72	126.8
3'SL	G2	TERM	4	5	96.47	136.6	116.2	18.31	116.6	99.32	132.1
3'SL	G2	TERM	5	5	92.27	140.4	112.8	19.78	103.5	101.7	126.0
3'SL	G2	TERM	6	5	35.33	163.0	107.3	48.00	111.0	93.14	134.1
3'SL	G2	TERM	7	5	74.78	122.0	98.92	18.14	102.7	87.95	107.1
3'SL	G2	TERM	8	5	77.15	131.7	103.5	22.91	97.29	88.86	122.5
3'SL	G3	PRE-TERM	1	1	174.7	174.7	174.7	NA	174.7	174.7	174.7
3'SL	G3	PRE-TERM	2	1	218.9	218.9	218.9	NA	218.9	218.9	218.9
3'SL	G3	PRE-TERM	3	1	209.5	209.5	209.5	NA	209.5	209.5	209.5
3'SL	G3	PRE-TERM	4	1	155.9	155.9	155.9	NA	155.9	155.9	155.9
3'SL	G3	PRE-TERM	5	1	177.6	177.6	177.6	NA	177.6	177.6	177.6
3'SL	G3	PRE-TERM	6	1	176.5	176.5	176.5	NA	176.5	176.5	176.5
3'SL	G3	PRE-TERM	7	1	124.4	124.4	124.4	NA	124.4	124.4	124.4
3'SL	G3	PRE-TERM	8	1	132.0	132.0	132.0	NA	132.0	132.0	132.0
3'SL	G3	PRE-TERM	10	1	117.7	117.7	117.7	NA	117.7	117.7	117.7
3'SL	G3	PRE-TERM	12	1	123.5	123.5	123.5	NA	123.5	123.5	123.5
3'SL	G3	PRE-TERM	14	1	128.8	128.8	128.8	NA	128.8	128.8	128.8
3'SL	G3	PRE-TERM	16	1	131.0	131.0	131.0	NA	131.0	131.0	131.0
3'SL	G3	TERM	1	1	154.8	154.8	154.8	NA	154.8	154.8	154.8
3'SL	G3	TERM	2	1	152.1	152.1	152.1	NA	152.1	152.1	152.1
3'SL	G3	TERM	3	1	149.3	149.3	149.3	NA	149.3	149.3	149.3
3'SL	G3	TERM	4	1	153.6	153.6	153.6	NA	153.6	153.6	153.6
3'SL	G3	TERM	5	1	140.2	140.2	140.2	NA	140.2	140.2	140.2
3'SL	G3	TERM	6	1	135.1	135.1	135.1	NA	135.1	135.1	135.1
3'SL	G3	TERM	7	1	138.1	138.1	138.1	NA	138.1	138.1	138.1
3'SL	G3	TERM	8	1	140.2	140.2	140.2	NA	140.2	140.2	140.2
3'SL	G4	TERM	1	1	241.3	241.3	241.3	NA	241.3	241.3	241.3
3'SL	G4	TERM	2	1	206.5	206.5	206.5	NA	206.5	206.5	206.5
3'SL	G4	TERM	3	1	162.2	162.2	162.2	NA	162.2	162.2	162.2
3'SL	G4	TERM	4	1	155.5	155.5	155.5	NA	155.5	155.5	155.5
3'SL	G4	TERM	5	1	140.1	140.1	140.1	NA	140.1	140.1	140.1
3'SL	G4	TERM	6	1	152.2	152.2	152.2	NA	152.2	152.2	152.2
3'SL	G4	TERM	7	1	119.8	119.8	119.8	NA	119.8	119.8	119.8
3'SL	G4	TERM	8	1	111.7	111.7	111.7	NA	111.7	111.7	111.7

Table S2 Concentration of Human Milk Oligosaccharides in Term or Preterm Milk At Different Weeks Postpartum Separated By Milk Group

** When there are results below the method limit of quanfification (LoQ) the result has been assigned value of 0.5 × LoQ, hence the minimum value appears to be the same in many cases. When a large number of datapoints are below LoQ this can also have the effect that the median = minimum.*

					HMO Concentration (mg/L)						
HMO	Milk Group	Study Arm	Week Post Partum	N	min *	max	mean	sd	median *	Quartile 1 (25%)	Quartile 3 (75%)
3FL	G1	PRE-TERM	1	19	112.2	844.2	293.9	172.1	239.0	184.9	348.7
3FL	G1	PRE-TERM	2	19	154.5	978.0	330.4	186.5	271.8	238.3	351.9
3FL	G1	PRE-TERM	3	19	182.1	1087	419.7	206.8	392.0	301.6	501.6
3FL	G1	PRE-TERM	4	18	205.2	1262	491.0	236.3	460.3	366.9	556.6
3FL	G1	PRE-TERM	5	19	220.2	1264	569.2	253.5	504.9	418.9	683.4
3FL	G1	PRE-TERM	6	18	75.94	1201	564.9	271.1	512.7	459.0	654.3
3FL	G1	PRE-TERM	7	18	232.4	1242	622.0	239.9	558.6	503.4	757.6
3FL	G1	PRE-TERM	8	18	270.0	1356	665.8	246.7	640.1	543.6	750.1
3FL	G1	PRE-TERM	10	18	356.0	1550	702.2	287.6	678.7	501.8	864.7
3FL	G1	PRE-TERM	12	15	492.5	1504	853.5	271.7	790.3	673.8	1009
3FL	G1	PRE-TERM	14	15	488.0	1914	889.4	376.6	740.6	643.2	1132
3FL	G1	PRE-TERM	16	15	435.5	2188	954.0	435.0	941.1	650.1	1114
3FL	G1	TERM	1	21	119.4	447.8	212.4	85.81	180.2	159.4	253.9
3FL	G1	TERM	2	19	145.3	662.5	300.1	129.6	273.5	199.0	383.4
3FL	G1	TERM	3	21	178.3	706.0	351.4	139.5	342.3	233.7	428.1
3FL	G1	TERM	4	21	200.0	690.4	401.5	161.3	380.1	263.0	528.0
3FL	G1	TERM	5	21	236.2	849.6	474.9	167.3	431.5	363.2	564.4
3FL	G1	TERM	6	20	287.4	1114	561.1	210.7	492.0	433.2	667.9
3FL	G1	TERM	7	20	242.1	1222	587.3	274.6	525.9	451.7	579.5
3FL	G1	TERM	8	21	151.0	1231	582.7	276.4	527.3	435.6	688.7
3FL	G2	PRE-TERM	1	5	908.2	1472	1176	208.3	1176	1078	1246
3FL	G2	PRE-TERM	2	5	991.9	1367	1111	151.9	1050	1019	1124
3FL	G2	PRE-TERM	3	5	1041	1359	1198	125.9	1165	1137	1287
3FL	G2	PRE-TERM	4	5	667.5	1649	1220	437.2	1457	836.1	1488
3FL	G2	PRE-TERM	5	5	829.2	2284	1442	612.4	1549	856.8	1690
3FL	G2	PRE-TERM	6	5	841.4	1663	1234	396.9	1034	969.7	1661
3FL	G2	PRE-TERM	7	5	829.5	1825	1347	453.4	1307	985.7	1787
3FL	G2	PRE-TERM	8	5	894.6	2465	1547	744.3	1186	956.4	2231
3FL	G2	PRE-TERM	10	4	958.9	2821	1775	860.6	1659	1134	2300
3FL	G2	PRE-TERM	12	5	1015	3101	1896	835.0	2011	1202	2151
3FL	G2	PRE-TERM	14	5	1158	3398	2335	847.5	2185	2077	2855
3FL	G2	PRE-TERM	16	3	1213	2505	1687	711.3	1342	1278	1923
3FL	G2	TERM	1	5	768.8	1240	995.1	207.2	1025	800.0	1142
3FL	G2	TERM	2	5	632.0	1361	1144	292.0	1245	1207	1277

Table S2 Concentration of Human Milk Oligosaccharides in Term or Preterm Milk At Different Weeks Postpartum Separated By Milk Group

** When there are results below the method limit of quanfification (LoQ) the result has been assigned value of 0.5 × LoQ, hence the minimum value appears to be the same in many cases. When a large number of datapoints are below LoQ this can also have the effect that the median = minimum.*

					HMO Concentration (mg/L)						
HMO	Milk Group	Study Arm	Week Post Partum	N	min *	max	mean	sd	median *	Quartile 1 (25%)	Quartile 3 (75%)
3FL	G2	TERM	3	5	780.7	1577	1202	293.3	1258	1089	1304
3FL	G2	TERM	4	5	782.1	1861	1345	428.6	1381	1076	1625
3FL	G2	TERM	5	5	799.4	1720	1390	430.0	1675	1062	1696
3FL	G2	TERM	6	5	624.3	1983	1316	595.5	1265	858.8	1848
3FL	G2	TERM	7	5	686.5	1978	1431	517.5	1460	1198	1834
3FL	G2	TERM	8	5	727.3	2013	1493	537.7	1609	1186	1931
3FL	G3	PRE-TERM	1	1	64.12	64.12	64.12	NA	64.12	64.12	64.12
3FL	G3	PRE-TERM	2	1	148.3	148.3	148.3	NA	148.3	148.3	148.3
3FL	G3	PRE-TERM	3	1	181.0	181.0	181.0	NA	181.0	181.0	181.0
3FL	G3	PRE-TERM	4	1	158.0	158.0	158.0	NA	158.0	158.0	158.0
3FL	G3	PRE-TERM	5	1	215.6	215.6	215.6	NA	215.6	215.6	215.6
3FL	G3	PRE-TERM	6	1	223.4	223.4	223.4	NA	223.4	223.4	223.4
3FL	G3	PRE-TERM	7	1	254.0	254.0	254.0	NA	254.0	254.0	254.0
3FL	G3	PRE-TERM	8	1	224.9	224.9	224.9	NA	224.9	224.9	224.9
3FL	G3	PRE-TERM	10	1	241.8	241.8	241.8	NA	241.8	241.8	241.8
3FL	G3	PRE-TERM	12	1	278.6	278.6	278.6	NA	278.6	278.6	278.6
3FL	G3	PRE-TERM	14	1	298.6	298.6	298.6	NA	298.6	298.6	298.6
3FL	G3	PRE-TERM	16	1	325.1	325.1	325.1	NA	325.1	325.1	325.1
3FL	G3	TERM	1	1	39.33	39.33	39.33	NA	39.33	39.33	39.33
3FL	G3	TERM	2	1	66.65	66.65	66.65	NA	66.65	66.65	66.65
3FL	G3	TERM	3	1	82.63	82.63	82.63	NA	82.63	82.63	82.63
3FL	G3	TERM	4	1	99.24	99.24	99.24	NA	99.24	99.24	99.24
3FL	G3	TERM	5	1	95.34	95.34	95.34	NA	95.34	95.34	95.34
3FL	G3	TERM	6	1	102.9	102.9	102.9	NA	102.9	102.9	102.9
3FL	G3	TERM	7	1	118.2	118.2	118.2	NA	118.2	118.2	118.2
3FL	G3	TERM	8	1	131.6	131.6	131.6	NA	131.6	131.6	131.6
3FL	G4	TERM	1	1	224.5	224.5	224.5	NA	224.5	224.5	224.5
3FL	G4	TERM	2	1	358.0	358.0	358.0	NA	358.0	358.0	358.0
3FL	G4	TERM	3	1	350.3	350.3	350.3	NA	350.3	350.3	350.3
3FL	G4	TERM	4	1	361.8	361.8	361.8	NA	361.8	361.8	361.8
3FL	G4	TERM	5	1	390.6	390.6	390.6	NA	390.6	390.6	390.6
3FL	G4	TERM	6	1	538.7	538.7	538.7	NA	538.7	538.7	538.7
3FL	G4	TERM	7	1	439.5	439.5	439.5	NA	439.5	439.5	439.5
3FL	G4	TERM	8	1	485.5	485.5	485.5	NA	485.5	485.5	485.5

Table S2 Concentration of Human Milk Oligosaccharides in Term or Preterm Milk At Different Weeks Postpartum Separated By Milk Group

** When there are results below the method limit of quanfification (LoQ) the result has been assigned value of 0.5 × LoQ, hence the minimum value appears to be the same in many cases. When a large number of datapoints are below LoQ this can also have the effect that the median = minimum.*

					HMO Concentration (mg/L)						
HMO	Milk Group	Study Arm	Week Post Partum	N	min *	max	mean	sd	median *	Quartile 1 (25%)	Quartile 3 (75%)
6'GL	G1	PRE-TERM	1	19	40.18	156.4	91.62	30.21	92.10	76.37	105.7
6'GL	G1	PRE-TERM	2	19	25.16	80.30	45.71	16.10	41.32	35.50	51.31
6'GL	G1	PRE-TERM	3	19	16.66	61.99	35.28	12.68	33.17	25.69	43.26
6'GL	G1	PRE-TERM	4	18	18.78	57.48	32.05	11.80	29.92	22.39	41.33
6'GL	G1	PRE-TERM	5	19	16.16	96.94	30.58	18.51	27.70	20.08	30.26
6'GL	G1	PRE-TERM	6	18	11.89	179.6	35.03	37.45	25.25	20.64	33.47
6'GL	G1	PRE-TERM	7	18	11.33	43.71	24.22	10.07	23.07	15.36	30.60
6'GL	G1	PRE-TERM	8	18	7.746	59.26	23.64	12.27	19.45	15.34	29.17
6'GL	G1	PRE-TERM	10	18	9.255	48.40	20.48	9.770	18.33	13.65	22.95
6'GL	G1	PRE-TERM	12	15	6.997	44.84	21.02	10.28	16.48	13.94	27.13
6'GL	G1	PRE-TERM	14	15	8.465	52.92	18.13	10.94	14.18	12.57	20.85
6'GL	G1	PRE-TERM	16	15	3.150	46.05	16.74	10.32	12.52	11.41	21.14
6'GL	G1	TERM	1	21	53.98	234.0	122.4	50.11	108.2	92.85	136.6
6'GL	G1	TERM	2	19	12.64	94.87	51.33	20.64	46.00	37.87	66.80
6'GL	G1	TERM	3	21	8.541	59.24	36.10	13.28	33.61	29.14	44.54
6'GL	G1	TERM	4	21	7.969	59.96	29.09	12.14	26.92	20.71	37.78
6'GL	G1	TERM	5	21	3.150	68.27	24.59	14.08	20.87	17.35	29.23
6'GL	G1	TERM	6	20	3.150	39.70	20.62	8.062	19.16	15.38	26.30
6'GL	G1	TERM	7	20	3.150	38.09	19.52	9.131	19.71	14.24	25.38
6'GL	G1	TERM	8	21	3.150	47.47	18.60	11.93	14.54	10.52	23.25
6'GL	G2	PRE-TERM	1	5	39.10	94.25	65.73	21.03	64.95	54.12	76.24
6'GL	G2	PRE-TERM	2	5	17.55	38.87	30.39	8.054	31.35	29.26	34.93
6'GL	G2	PRE-TERM	3	5	16.82	39.49	28.42	8.448	27.06	25.85	32.89
6'GL	G2	PRE-TERM	4	5	18.30	26.36	21.88	3.388	21.92	18.91	23.91
6'GL	G2	PRE-TERM	5	5	21.56	38.77	26.96	6.792	25.00	23.70	25.75
6'GL	G2	PRE-TERM	6	5	3.150	29.68	19.97	10.47	23.56	17.12	26.32
6'GL	G2	PRE-TERM	7	5	12.88	25.95	17.58	5.015	16.62	14.82	17.65
6'GL	G2	PRE-TERM	8	5	9.818	25.22	17.96	6.612	18.51	12.86	23.40
6'GL	G2	PRE-TERM	10	4	13.41	28.95	19.32	7.110	17.47	14.26	22.53
6'GL	G2	PRE-TERM	12	5	10.43	24.69	17.87	5.120	17.62	17.17	19.42
6'GL	G2	PRE-TERM	14	5	13.05	32.99	22.95	8.405	25.56	15.52	27.62
6'GL	G2	PRE-TERM	16	3	14.07	19.16	16.37	2.581	15.88	14.98	17.52
6'GL	G2	TERM	1	5	122.7	175.0	146.7	22.98	134.4	134.2	167.4
6'GL	G2	TERM	2	5	28.62	76.66	53.16	17.48	52.93	48.00	59.61

Table S2 Concentration of Human Milk Oligosaccharides in Term or Preterm Milk At Different Weeks Postpartum Separated By Milk Group

** When there are results below the method limit of quanfification (LoQ) the result has been assigned value of 0.5 × LoQ, hence the minimum value appears to be the same in many cases. When a large number of datapoints are below LoQ this can also have the effect that the median = minimum.*

					HMO Concentration (mg/L)						
HMO	Milk Group	Study Arm	Week Post Partum	N	min *	max	mean	sd	median *	Quartile 1 (25%)	Quartile 3 (75%)
6'GL	G2	TERM	3	5	15.76	52.37	34.13	12.99	34.39	32.59	35.52
6'GL	G2	TERM	4	5	3.150	27.99	14.69	12.29	12.07	3.150	27.10
6'GL	G2	TERM	5	5	3.150	39.74	17.64	15.30	19.70	3.150	22.47
6'GL	G2	TERM	6	5	6.478	20.80	13.16	6.216	12.30	8.078	18.16
6'GL	G2	TERM	7	5	6.983	22.00	15.27	5.498	16.67	13.79	16.93
6'GL	G2	TERM	8	5	6.629	20.67	14.34	5.059	14.56	13.89	15.93
6'GL	G3	PRE-TERM	1	1	99.02	99.02	99.02	NA	99.02	99.02	99.02
6'GL	G3	PRE-TERM	2	1	51.62	51.62	51.62	NA	51.62	51.62	51.62
6'GL	G3	PRE-TERM	3	1	34.12	34.12	34.12	NA	34.12	34.12	34.12
6'GL	G3	PRE-TERM	4	1	28.35	28.35	28.35	NA	28.35	28.35	28.35
6'GL	G3	PRE-TERM	5	1	30.35	30.35	30.35	NA	30.35	30.35	30.35
6'GL	G3	PRE-TERM	6	1	30.43	30.43	30.43	NA	30.43	30.43	30.43
6'GL	G3	PRE-TERM	7	1	16.02	16.02	16.02	NA	16.02	16.02	16.02
6'GL	G3	PRE-TERM	8	1	18.65	18.65	18.65	NA	18.65	18.65	18.65
6'GL	G3	PRE-TERM	10	1	14.55	14.55	14.55	NA	14.55	14.55	14.55
6'GL	G3	PRE-TERM	12	1	13.66	13.66	13.66	NA	13.66	13.66	13.66
6'GL	G3	PRE-TERM	14	1	14.69	14.69	14.69	NA	14.69	14.69	14.69
6'GL	G3	PRE-TERM	16	1	12.31	12.31	12.31	NA	12.31	12.31	12.31
6'GL	G3	TERM	1	1	109.9	109.9	109.9	NA	109.9	109.9	109.9
6'GL	G3	TERM	2	1	50.71	50.71	50.71	NA	50.71	50.71	50.71
6'GL	G3	TERM	3	1	38.43	38.43	38.43	NA	38.43	38.43	38.43
6'GL	G3	TERM	4	1	31.73	31.73	31.73	NA	31.73	31.73	31.73
6'GL	G3	TERM	5	1	21.24	21.24	21.24	NA	21.24	21.24	21.24
6'GL	G3	TERM	6	1	16.32	16.32	16.32	NA	16.32	16.32	16.32
6'GL	G3	TERM	7	1	15.03	15.03	15.03	NA	15.03	15.03	15.03
6'GL	G3	TERM	8	1	12.89	12.89	12.89	NA	12.89	12.89	12.89
6'GL	G4	TERM	1	1	176.0	176.0	176.0	NA	176.0	176.0	176.0
6'GL	G4	TERM	2	1	92.67	92.67	92.67	NA	92.67	92.67	92.67
6'GL	G4	TERM	3	1	51.40	51.40	51.40	NA	51.40	51.40	51.40
6'GL	G4	TERM	4	1	39.76	39.76	39.76	NA	39.76	39.76	39.76
6'GL	G4	TERM	5	1	30.64	30.64	30.64	NA	30.64	30.64	30.64
6'GL	G4	TERM	6	1	39.81	39.81	39.81	NA	39.81	39.81	39.81
6'GL	G4	TERM	7	1	24.24	24.24	24.24	NA	24.24	24.24	24.24
6'GL	G4	TERM	8	1	20.55	20.55	20.55	NA	20.55	20.55	20.55

Table S2 Concentration of Human Milk Oligosaccharides in Term or Preterm Milk At Different Weeks Postpartum Separated By Milk Group

** When there are results below the method limit of quanfification (LoQ) the result has been assigned value of 0.5 × LoQ, hence the minimum value appears to be the same in many cases. When a large number of datapoints are below LoQ this can also have the effect that the median = minimum.*

					HMO Concentration (mg/L)						
HMO	Milk Group	Study Arm	Week Post Partum	N	min *	max	mean	sd	median *	Quartile 1 (25%)	Quartile 3 (75%)
6'SL	G1	PRE-TERM	1	19	103.1	1132	524.0	269.7	555.8	310.6	656.5
6'SL	G1	PRE-TERM	2	19	63.98	1135	548.8	289.4	582.9	323.2	751.2
6'SL	G1	PRE-TERM	3	19	74.88	926.1	493.7	234.8	517.0	347.5	653.4
6'SL	G1	PRE-TERM	4	18	61.36	705.7	437.0	169.0	471.4	344.0	540.3
6'SL	G1	PRE-TERM	5	19	74.89	569.3	344.5	144.0	382.5	223.1	442.7
6'SL	G1	PRE-TERM	6	18	10.00	534.3	309.5	145.0	340.7	190.1	406.5
6'SL	G1	PRE-TERM	7	18	135.1	524.7	264.3	118.3	276.6	151.6	328.6
6'SL	G1	PRE-TERM	8	18	116.1	476.6	245.6	105.8	235.2	154.6	293.9
6'SL	G1	PRE-TERM	10	18	100.6	309.4	178.8	66.34	161.4	130.4	192.1
6'SL	G1	PRE-TERM	12	15	84.36	256.2	152.0	47.93	150.6	117.4	185.1
6'SL	G1	PRE-TERM	14	15	61.11	212.2	117.2	43.77	101.1	86.36	146.5
6'SL	G1	PRE-TERM	16	15	46.43	196.6	98.58	47.51	85.22	60.33	129.2
6'SL	G1	TERM	1	21	222.0	794.7	501.0	144.5	497.2	428.5	567.1
6'SL	G1	TERM	2	19	363.7	1084	658.6	188.6	626.3	534.1	781.7
6'SL	G1	TERM	3	21	325.3	985.4	585.9	182.9	531.3	441.9	754.7
6'SL	G1	TERM	4	21	216.1	797.3	493.4	162.0	443.0	383.7	568.0
6'SL	G1	TERM	5	21	24.60	693.3	363.1	160.9	341.6	309.8	428.5
6'SL	G1	TERM	6	20	153.7	700.9	332.5	142.1	315.8	234.5	400.8
6'SL	G1	TERM	7	20	101.5	662.3	281.1	145.5	250.9	180.3	352.0
6'SL	G1	TERM	8	21	53.06	671.2	238.3	149.1	195.5	145.8	301.0
6'SL	G2	PRE-TERM	1	5	145.3	501.0	334.9	146.2	303.3	263.9	461.0
6'SL	G2	PRE-TERM	2	5	86.13	559.2	311.1	175.2	318.4	224.7	367.0
6'SL	G2	PRE-TERM	3	5	61.39	555.9	311.0	175.2	309.8	299.8	328.2
6'SL	G2	PRE-TERM	4	5	53.89	467.7	260.4	148.6	249.1	230.5	301.0
6'SL	G2	PRE-TERM	5	5	74.89	331.6	238.4	103.2	264.0	208.2	313.4
6'SL	G2	PRE-TERM	6	5	74.47	353.9	238.4	112.0	247.0	191.0	325.7
6'SL	G2	PRE-TERM	7	5	55.02	354.5	210.4	117.5	177.8	168.0	296.6
6'SL	G2	PRE-TERM	8	5	57.52	315.4	173.9	95.55	146.4	142.1	208.3
6'SL	G2	PRE-TERM	10	4	100.6	249.2	173.1	60.68	171.2	153.5	190.8
6'SL	G2	PRE-TERM	12	5	65.69	202.3	132.1	50.86	122.1	114.2	156.3
6'SL	G2	PRE-TERM	14	5	69.80	158.9	105.2	43.22	77.58	74.24	145.4
6'SL	G2	PRE-TERM	16	3	109.5	135.2	123.6	13.05	126.1	117.8	130.7
6'SL	G2	TERM	1	5	295.7	634.7	465.0	152.7	401.4	374.6	618.4
6'SL	G2	TERM	2	5	311.3	786.1	561.1	207.8	524.7	423.8	759.7

Table S2 Concentration of Human Milk Oligosaccharides in Term or Preterm Milk At Different Weeks Postpartum Separated By Milk Group

** When there are results below the method limit of quanfification (LoQ) the result has been assigned value of 0.5 × LoQ, hence the minimum value appears to be the same in many cases. When a large number of datapoints are below LoQ this can also have the effect that the median = minimum.*

					HMO Concentration (mg/L)						
HMO	Milk Group	Study Arm	Week Post Partum	N	min *	max	mean	sd	median *	Quartile 1 (25%)	Quartile 3 (75%)
6'SL	G2	TERM	3	5	262.0	718.6	478.3	178.3	491.0	355.6	564.6
6'SL	G2	TERM	4	5	202.8	623.8	417.7	157.9	457.8	334.8	469.3
6'SL	G2	TERM	5	5	129.4	549.1	336.2	157.3	365.6	249.8	387.3
6'SL	G2	TERM	6	5	112.5	318.7	204.6	89.82	179.5	134.9	277.4
6'SL	G2	TERM	7	5	89.66	234.6	173.6	72.82	220.1	98.67	225.2
6'SL	G2	TERM	8	5	86.83	211.6	161.8	52.93	176.2	129.6	205.0
6'SL	G3	PRE-TERM	1	1	673.9	673.9	673.9	NA	673.9	673.9	673.9
6'SL	G3	PRE-TERM	2	1	654.3	654.3	654.3	NA	654.3	654.3	654.3
6'SL	G3	PRE-TERM	3	1	441.3	441.3	441.3	NA	441.3	441.3	441.3
6'SL	G3	PRE-TERM	4	1	324.0	324.0	324.0	NA	324.0	324.0	324.0
6'SL	G3	PRE-TERM	5	1	270.7	270.7	270.7	NA	270.7	270.7	270.7
6'SL	G3	PRE-TERM	6	1	229.1	229.1	229.1	NA	229.1	229.1	229.1
6'SL	G3	PRE-TERM	7	1	128.6	128.6	128.6	NA	128.6	128.6	128.6
6'SL	G3	PRE-TERM	8	1	138.2	138.2	138.2	NA	138.2	138.2	138.2
6'SL	G3	PRE-TERM	10	1	91.24	91.24	91.24	NA	91.24	91.24	91.24
6'SL	G3	PRE-TERM	12	1	72.75	72.75	72.75	NA	72.75	72.75	72.75
6'SL	G3	PRE-TERM	14	1	60.09	60.09	60.09	NA	60.09	60.09	60.09
6'SL	G3	PRE-TERM	16	1	48.01	48.01	48.01	NA	48.01	48.01	48.01
6'SL	G3	TERM	1	1	648.7	648.7	648.7	NA	648.7	648.7	648.7
6'SL	G3	TERM	2	1	598.9	598.9	598.9	NA	598.9	598.9	598.9
6'SL	G3	TERM	3	1	511.9	511.9	511.9	NA	511.9	511.9	511.9
6'SL	G3	TERM	4	1	419.6	419.6	419.6	NA	419.6	419.6	419.6
6'SL	G3	TERM	5	1	355.0	355.0	355.0	NA	355.0	355.0	355.0
6'SL	G3	TERM	6	1	294.2	294.2	294.2	NA	294.2	294.2	294.2
6'SL	G3	TERM	7	1	273.7	273.7	273.7	NA	273.7	273.7	273.7
6'SL	G3	TERM	8	1	192.3	192.3	192.3	NA	192.3	192.3	192.3
6'SL	G4	TERM	1	1	436.4	436.4	436.4	NA	436.4	436.4	436.4
6'SL	G4	TERM	2	1	888.0	888.0	888.0	NA	888.0	888.0	888.0
6'SL	G4	TERM	3	1	670.5	670.5	670.5	NA	670.5	670.5	670.5
6'SL	G4	TERM	4	1	489.7	489.7	489.7	NA	489.7	489.7	489.7
6'SL	G4	TERM	5	1	357.8	357.8	357.8	NA	357.8	357.8	357.8
6'SL	G4	TERM	6	1	305.6	305.6	305.6	NA	305.6	305.6	305.6
6'SL	G4	TERM	7	1	193.1	193.1	193.1	NA	193.1	193.1	193.1
6'SL	G4	TERM	8	1	150.4	150.4	150.4	NA	150.4	150.4	150.4

Table S2 Concentration of Human Milk Oligosaccharides in Term or Preterm Milk At Different Weeks Postpartum Separated By Milk Group

** When there are results below the method limit of quanfification (LoQ) the result has been assigned value of 0.5 × LoQ, hence the minimum value appears to be the same in many cases. When a large number of datapoints are below LoQ this can also have the effect that the median = minimum.*

					HMO Concentration (mg/L)						
HMO	Milk Group	Study Arm	Week Post Partum	N	min *	max	mean	sd	median *	Quartile 1 (25%)	Quartile 3 (75%)
A-Tetrasaccharide	G1	PRE-TERM	1	19	7.500	315.1	63.25	91.82	7.500	7.500	62.65
A-Tetrasaccharide	G1	PRE-TERM	2	19	7.500	218.2	44.59	63.96	7.500	7.500	55.77
A-Tetrasaccharide	G1	PRE-TERM	3	19	7.500	250.0	47.75	71.06	7.500	7.500	56.82
A-Tetrasaccharide	G1	PRE-TERM	4	18	7.500	224.7	50.08	68.81	7.500	7.500	62.59
A-Tetrasaccharide	G1	PRE-TERM	5	19	7.500	226.0	47.29	64.82	7.500	7.500	65.24
A-Tetrasaccharide	G1	PRE-TERM	6	18	7.500	232.6	51.49	69.77	11.34	7.500	80.93
A-Tetrasaccharide	G1	PRE-TERM	7	18	7.500	266.6	51.89	76.09	7.500	7.500	83.86
A-Tetrasaccharide	G1	PRE-TERM	8	18	7.500	262.8	56.15	84.73	7.500	7.500	84.89
A-Tetrasaccharide	G1	PRE-TERM	10	18	7.500	322.7	53.88	85.50	7.500	7.500	82.15
A-Tetrasaccharide	G1	PRE-TERM	12	15	7.500	334.5	53.68	93.23	7.500	7.500	55.86
A-Tetrasaccharide	G1	PRE-TERM	14	15	7.500	300.7	53.93	81.65	7.500	7.500	81.50
A-Tetrasaccharide	G1	PRE-TERM	16	15	7.500	329.8	60.03	89.45	7.500	7.500	90.98
A-Tetrasaccharide	G1	TERM	1	21	7.500	188.2	46.66	57.67	7.500	7.500	62.07
A-Tetrasaccharide	G1	TERM	2	19	7.500	149.0	33.93	39.65	7.500	7.500	40.79
A-Tetrasaccharide	G1	TERM	3	21	7.500	153.9	29.67	39.53	7.500	7.500	33.43
A-Tetrasaccharide	G1	TERM	4	21	7.500	162.3	31.90	41.06	7.500	7.500	39.78
A-Tetrasaccharide	G1	TERM	5	21	7.500	138.9	29.05	36.05	7.500	7.500	32.57
A-Tetrasaccharide	G1	TERM	6	20	7.500	183.8	31.68	46.45	7.500	7.500	29.48
A-Tetrasaccharide	G1	TERM	7	20	7.500	165.4	30.38	43.10	7.500	7.500	32.30
A-Tetrasaccharide	G1	TERM	8	21	7.500	181.0	30.42	45.42	7.500	7.500	26.08
A-Tetrasaccharide	G2	PRE-TERM	1	5	7.500	7.500	7.500	0.0000	7.500	7.500	7.500
A-Tetrasaccharide	G2	PRE-TERM	2	5	7.500	7.500	7.500	0.0000	7.500	7.500	7.500
A-Tetrasaccharide	G2	PRE-TERM	3	5	7.500	7.500	7.500	0.0000	7.500	7.500	7.500
A-Tetrasaccharide	G2	PRE-TERM	4	5	7.500	7.500	7.500	0.0000	7.500	7.500	7.500
A-Tetrasaccharide	G2	PRE-TERM	5	5	7.500	7.500	7.500	0.0000	7.500	7.500	7.500
A-Tetrasaccharide	G2	PRE-TERM	6	5	7.500	16.81	9.361	4.162	7.500	7.500	7.500
A-Tetrasaccharide	G2	PRE-TERM	7	5	7.500	7.500	7.500	0.0000	7.500	7.500	7.500
A-Tetrasaccharide	G2	PRE-TERM	8	5	7.500	7.500	7.500	0.0000	7.500	7.500	7.500
A-Tetrasaccharide	G2	PRE-TERM	10	4	7.500	7.500	7.500	0.0000	7.500	7.500	7.500
A-Tetrasaccharide	G2	PRE-TERM	12	5	7.500	7.500	7.500	0.0000	7.500	7.500	7.500
A-Tetrasaccharide	G2	PRE-TERM	14	5	7.500	7.500	7.500	0.0000	7.500	7.500	7.500
A-Tetrasaccharide	G2	PRE-TERM	16	3	7.500	7.500	7.500	0.0000	7.500	7.500	7.500
A-Tetrasaccharide	G2	TERM	1	5	7.500	7.500	7.500	0.0000	7.500	7.500	7.500
A-Tetrasaccharide	G2	TERM	2	5	7.500	7.500	7.500	0.0000	7.500	7.500	7.500

Table S2 Concentration of Human Milk Oligosaccharides in Term or Preterm Milk At Different Weeks Postpartum Separated By Milk Group

** When there are results below the method limit of quanfification (LoQ) the result has been assigned value of 0.5 × LoQ, hence the minimum value appears to be the same in many cases. When a large number of datapoints are below LoQ this can also have the effect that the median = minimum.*

HMO	Milk Group	Study Arm	Week Post Partum	N	HMO Concentration (mg/L) min *	max	mean	sd	median *	Quartile 1 (25%)	Quartile 3 (75%)
۹-Tetrasaccharide	G2	TERM	3	5	7.500	7.500	7.500	0.0000	7.500	7.500	7.500
۹-Tetrasaccharide	G2	TERM	4	5	7.500	28.98	11.80	9.605	7.500	7.500	7.500
۹-Tetrasaccharide	G2	TERM	5	5	7.500	27.37	11.47	8.886	7.500	7.500	7.500
۹-Tetrasaccharide	G2	TERM	6	5	7.500	7.500	7.500	0.0000	7.500	7.500	7.500
۹-Tetrasaccharide	G2	TERM	7	5	7.500	7.500	7.500	0.0000	7.500	7.500	7.500
۹-Tetrasaccharide	G2	TERM	8	5	7.500	7.500	7.500	0.0000	7.500	7.500	7.500
۹-Tetrasaccharide	G3	PRE-TERM	1	1	7.500	7.500	7.500	NA	7.500	7.500	7.500
۹-Tetrasaccharide	G3	PRE-TERM	2	1	7.500	7.500	7.500	NA	7.500	7.500	7.500
۹-Tetrasaccharide	G3	PRE-TERM	3	1	7.500	7.500	7.500	NA	7.500	7.500	7.500
۹-Tetrasaccharide	G3	PRE-TERM	4	1	7.500	7.500	7.500	NA	7.500	7.500	7.500
۹-Tetrasaccharide	G3	PRE-TERM	5	1	7.500	7.500	7.500	NA	7.500	7.500	7.500
۹-Tetrasaccharide	G3	PRE-TERM	6	1	7.500	7.500	7.500	NA	7.500	7.500	7.500
۹-Tetrasaccharide	G3	PRE-TERM	7	1	7.500	7.500	7.500	NA	7.500	7.500	7.500
۹-Tetrasaccharide	G3	PRE-TERM	8	1	7.500	7.500	7.500	NA	7.500	7.500	7.500
۹-Tetrasaccharide	G3	PRE-TERM	10	1	7.500	7.500	7.500	NA	7.500	7.500	7.500
۹-Tetrasaccharide	G3	PRE-TERM	12	1	7.500	7.500	7.500	NA	7.500	7.500	7.500
۹-Tetrasaccharide	G3	PRE-TERM	14	1	7.500	7.500	7.500	NA	7.500	7.500	7.500
۹-Tetrasaccharide	G3	PRE-TERM	16	1	7.500	7.500	7.500	NA	7.500	7.500	7.500
۹-Tetrasaccharide	G3	TERM	1	1	43.15	43.15	43.15	NA	43.15	43.15	43.15
۹-Tetrasaccharide	G3	TERM	2	1	47.28	47.28	47.28	NA	47.28	47.28	47.28
۹-Tetrasaccharide	G3	TERM	3	1	46.17	46.17	46.17	NA	46.17	46.17	46.17
۹-Tetrasaccharide	G3	TERM	4	1	41.87	41.87	41.87	NA	41.87	41.87	41.87
۹-Tetrasaccharide	G3	TERM	5	1	36.38	36.38	36.38	NA	36.38	36.38	36.38
۹-Tetrasaccharide	G3	TERM	6	1	37.04	37.04	37.04	NA	37.04	37.04	37.04
۹-Tetrasaccharide	G3	TERM	7	1	27.14	27.14	27.14	NA	27.14	27.14	27.14
۹-Tetrasaccharide	G3	TERM	8	1	33.98	33.98	33.98	NA	33.98	33.98	33.98
۹-Tetrasaccharide	G4	TERM	1	1	7.500	7.500	7.500	NA	7.500	7.500	7.500
۹-Tetrasaccharide	G4	TERM	2	1	7.500	7.500	7.500	NA	7.500	7.500	7.500
۹-Tetrasaccharide	G4	TERM	3	1	7.500	7.500	7.500	NA	7.500	7.500	7.500
۹-Tetrasaccharide	G4	TERM	4	1	7.500	7.500	7.500	NA	7.500	7.500	7.500
۹-Tetrasaccharide	G4	TERM	5	1	7.500	7.500	7.500	NA	7.500	7.500	7.500
۹-Tetrasaccharide	G4	TERM	6	1	7.500	7.500	7.500	NA	7.500	7.500	7.500
۹-Tetrasaccharide	G4	TERM	7	1	7.500	7.500	7.500	NA	7.500	7.500	7.500
۹-Tetrasaccharide	G4	TERM	8	1	7.500	7.500	7.500	NA	7.500	7.500	7.500

Table S2 Concentration of Human Milk Oligosaccharides in Term or Preterm Milk At Different Weeks Postpartum Separated By Milk Group

** When there are results below the method limit of quanfification (LoQ) the result has been assigned value of 0.5 × LoQ, hence the minimum value appears to be the same in many cases. When a large number of datapoints are below LoQ this can also have the effect that the median = minimum.*

					HMO Concentration (mg/L)						
HMO	Milk Group	Study Arm	Week Post Partum	N	min *	max	mean	sd	median *	Quartile 1 (25%)	Quartile 3 (75%)
DFLNHa	G1	PRE-TERM	1	19	16.50	420.0	187.6	114.4	151.7	116.4	248.4
DFLNHa	G1	PRE-TERM	2	19	16.50	448.7	215.6	129.2	197.9	109.0	317.7
DFLNHa	G1	PRE-TERM	3	19	16.50	364.2	189.7	103.8	201.5	107.4	266.0
DFLNHa	G1	PRE-TERM	4	18	16.50	323.6	167.2	88.40	179.0	94.93	228.2
DFLNHa	G1	PRE-TERM	5	19	16.50	260.8	127.4	63.05	138.9	68.79	166.0
DFLNHa	G1	PRE-TERM	6	18	16.50	685.1	152.8	146.4	136.0	94.85	151.3
DFLNHa	G1	PRE-TERM	7	18	16.50	285.3	100.9	65.17	84.94	63.73	135.1
DFLNHa	G1	PRE-TERM	8	18	16.50	252.0	103.0	65.85	96.74	58.65	135.5
DFLNHa	G1	PRE-TERM	10	18	16.50	103.6	50.74	32.58	51.50	16.50	79.64
DFLNHa	G1	PRE-TERM	12	15	16.50	83.70	40.26	21.61	38.58	16.50	54.49
DFLNHa	G1	PRE-TERM	14	15	16.50	77.74	40.22	19.83	43.98	16.50	52.75
DFLNHa	G1	PRE-TERM	16	15	16.50	60.97	32.50	16.46	37.84	16.50	44.38
DFLNHa	G1	TERM	1	21	16.50	306.3	158.0	79.74	158.2	90.49	184.8
DFLNHa	G1	TERM	2	19	97.87	582.8	272.2	129.7	250.9	185.1	320.1
DFLNHa	G1	TERM	3	21	89.36	472.0	256.4	112.1	240.4	190.4	341.2
DFLNHa	G1	TERM	4	21	66.82	473.0	237.9	113.7	206.3	170.4	342.7
DFLNHa	G1	TERM	5	21	53.69	383.9	192.5	108.0	163.0	120.9	283.7
DFLNHa	G1	TERM	6	20	16.50	343.0	149.2	90.54	133.6	103.6	194.9
DFLNHa	G1	TERM	7	20	16.50	247.1	113.1	71.31	115.1	62.16	130.5
DFLNHa	G1	TERM	8	21	16.50	311.2	105.5	83.70	81.61	48.27	129.0
DFLNHa	G2	PRE-TERM	1	5	16.50	16.50	16.50	0.0000	16.50	16.50	16.50
DFLNHa	G2	PRE-TERM	2	5	16.50	16.50	16.50	0.0000	16.50	16.50	16.50
DFLNHa	G2	PRE-TERM	3	5	16.50	16.50	16.50	0.0000	16.50	16.50	16.50
DFLNHa	G2	PRE-TERM	4	5	16.50	16.50	16.50	0.0000	16.50	16.50	16.50
DFLNHa	G2	PRE-TERM	5	5	16.50	16.50	16.50	0.0000	16.50	16.50	16.50
DFLNHa	G2	PRE-TERM	6	5	16.50	16.50	16.50	0.0000	16.50	16.50	16.50
DFLNHa	G2	PRE-TERM	7	5	16.50	42.44	21.69	11.60	16.50	16.50	16.50
DFLNHa	G2	PRE-TERM	8	5	16.50	42.01	21.60	11.41	16.50	16.50	16.50
DFLNHa	G2	PRE-TERM	10	4	16.50	16.50	16.50	0.0000	16.50	16.50	16.50
DFLNHa	G2	PRE-TERM	12	5	16.50	36.86	20.57	9.104	16.50	16.50	16.50
DFLNHa	G2	PRE-TERM	14	5	16.50	139.6	41.13	55.06	16.50	16.50	16.50
DFLNHa	G2	PRE-TERM	16	3	16.50	16.50	16.50	0.0000	16.50	16.50	16.50
DFLNHa	G2	TERM	1	5	16.50	16.50	16.50	0.0000	16.50	16.50	16.50
DFLNHa	G2	TERM	2	5	16.50	16.50	16.50	0.0000	16.50	16.50	16.50

Table S2 Concentration of Human Milk Oligosaccharides in Term or Preterm Milk At Different Weeks Postpartum Separated By Milk Group

** When there are results below the method limit of quanfification (LoQ) the result has been assigned value of 0.5 × LoQ, hence the minimum value appears to be the same in many cases. When a large number of datapoints are below LoQ this can also have the effect that the median = minimum.*

					HMO Concentration (mg/L)						
HMO	Milk Group	Study Arm	Week Post Partum	N	min *	max	mean	sd	median *	Quartile 1 (25%)	Quartile 3 (75%)
DFLNHa	G2	TERM	3	5	16.50	16.50	16.50	0.0000	16.50	16.50	16.50
DFLNHa	G2	TERM	4	5	16.50	16.50	16.50	0.0000	16.50	16.50	16.50
DFLNHa	G2	TERM	5	5	16.50	16.50	16.50	0.0000	16.50	16.50	16.50
DFLNHa	G2	TERM	6	5	16.50	16.50	16.50	0.0000	16.50	16.50	16.50
DFLNHa	G2	TERM	7	5	16.50	16.50	16.50	0.0000	16.50	16.50	16.50
DFLNHa	G2	TERM	8	5	16.50	16.50	16.50	0.0000	16.50	16.50	16.50
DFLNHa	G3	PRE-TERM	1	1	577.3	577.3	577.3	NA	577.3	577.3	577.3
DFLNHa	G3	PRE-TERM	2	1	495.2	495.2	495.2	NA	495.2	495.2	495.2
DFLNHa	G3	PRE-TERM	3	1	430.4	430.4	430.4	NA	430.4	430.4	430.4
DFLNHa	G3	PRE-TERM	4	1	583.6	583.6	583.6	NA	583.6	583.6	583.6
DFLNHa	G3	PRE-TERM	5	1	623.8	623.8	623.8	NA	623.8	623.8	623.8
DFLNHa	G3	PRE-TERM	6	1	542.4	542.4	542.4	NA	542.4	542.4	542.4
DFLNHa	G3	PRE-TERM	7	1	418.7	418.7	418.7	NA	418.7	418.7	418.7
DFLNHa	G3	PRE-TERM	8	1	351.9	351.9	351.9	NA	351.9	351.9	351.9
DFLNHa	G3	PRE-TERM	10	1	249.7	249.7	249.7	NA	249.7	249.7	249.7
DFLNHa	G3	PRE-TERM	12	1	179.2	179.2	179.2	NA	179.2	179.2	179.2
DFLNHa	G3	PRE-TERM	14	1	58.00	58.00	58.00	NA	58.00	58.00	58.00
DFLNHa	G3	PRE-TERM	16	1	54.78	54.78	54.78	NA	54.78	54.78	54.78
DFLNHa	G3	TERM	1	1	488.4	488.4	488.4	NA	488.4	488.4	488.4
DFLNHa	G3	TERM	2	1	664.0	664.0	664.0	NA	664.0	664.0	664.0
DFLNHa	G3	TERM	3	1	759.1	759.1	759.1	NA	759.1	759.1	759.1
DFLNHa	G3	TERM	4	1	709.4	709.4	709.4	NA	709.4	709.4	709.4
DFLNHa	G3	TERM	5	1	747.0	747.0	747.0	NA	747.0	747.0	747.0
DFLNHa	G3	TERM	6	1	563.0	563.0	563.0	NA	563.0	563.0	563.0
DFLNHa	G3	TERM	7	1	525.5	525.5	525.5	NA	525.5	525.5	525.5
DFLNHa	G3	TERM	8	1	463.1	463.1	463.1	NA	463.1	463.1	463.1
DFLNHa	G4	TERM	1	1	16.50	16.50	16.50	NA	16.50	16.50	16.50
DFLNHa	G4	TERM	2	1	52.78	52.78	52.78	NA	52.78	52.78	52.78
DFLNHa	G4	TERM	3	1	16.50	16.50	16.50	NA	16.50	16.50	16.50
DFLNHa	G4	TERM	4	1	16.50	16.50	16.50	NA	16.50	16.50	16.50
DFLNHa	G4	TERM	5	1	16.50	16.50	16.50	NA	16.50	16.50	16.50
DFLNHa	G4	TERM	6	1	16.50	16.50	16.50	NA	16.50	16.50	16.50
DFLNHa	G4	TERM	7	1	16.50	16.50	16.50	NA	16.50	16.50	16.50
DFLNHa	G4	TERM	8	1	16.50	16.50	16.50	NA	16.50	16.50	16.50

Table S2 Concentration of Human Milk Oligosaccharides in Term or Preterm Milk At Different Weeks Postpartum Separated By Milk Group

** When there are results below the method limit of quanfification (LoQ) the result has been assigned value of 0.5 × LoQ, hence the minimum value appears to be the same in many cases. When a large number of datapoints are below LoQ this can also have the effect that the median = minimum.*

					HMO Concentration (mg/L)						
HMO	Milk Group	Study Arm	Week Post Partum	N	min *	max	mean	sd	median *	Quartile 1 (25%)	Quartile 3 (75%)
DSLNT	G1	PRE-TERM	1	19	17.50	703.2	355.6	175.6	385.2	263.4	461.4
DSLNT	G1	PRE-TERM	2	19	17.50	822.5	428.1	185.4	401.0	336.8	526.7
DSLNT	G1	PRE-TERM	3	19	169.1	908.0	441.5	211.6	354.2	300.2	484.2
DSLNT	G1	PRE-TERM	4	18	128.4	796.0	395.2	193.6	325.1	276.1	471.3
DSLNT	G1	PRE-TERM	5	19	129.9	811.9	366.8	196.4	291.5	235.4	430.9
DSLNT	G1	PRE-TERM	6	18	105.5	628.2	300.1	150.9	260.3	203.3	336.3
DSLNT	G1	PRE-TERM	7	18	100.2	597.5	292.6	153.6	218.5	182.1	362.1
DSLNT	G1	PRE-TERM	8	18	97.12	530.6	278.8	132.9	233.6	183.7	398.9
DSLNT	G1	PRE-TERM	10	18	84.32	435.1	232.6	105.8	200.8	162.4	310.2
DSLNT	G1	PRE-TERM	12	15	72.56	336.6	212.2	75.11	203.4	161.8	273.7
DSLNT	G1	PRE-TERM	14	15	102.0	311.8	185.5	64.84	169.3	135.4	238.7
DSLNT	G1	PRE-TERM	16	15	88.23	253.6	175.6	49.76	173.3	149.4	212.5
DSLNT	G1	TERM	1	21	212.2	649.5	353.0	104.7	340.2	273.5	385.7
DSLNT	G1	TERM	2	19	17.50	880.1	367.6	179.1	363.2	255.8	439.6
DSLNT	G1	TERM	3	21	117.1	836.5	380.3	143.7	396.2	293.3	421.1
DSLNT	G1	TERM	4	21	94.18	630.2	318.8	114.4	328.2	248.5	383.5
DSLNT	G1	TERM	5	21	75.20	555.7	259.1	103.4	274.8	205.7	294.1
DSLNT	G1	TERM	6	20	69.19	426.1	239.6	89.63	220.7	184.2	307.0
DSLNT	G1	TERM	7	20	48.04	437.5	219.8	92.25	193.7	158.0	286.2
DSLNT	G1	TERM	8	21	60.14	422.7	179.3	80.01	169.8	121.5	213.7
DSLNT	G2	PRE-TERM	1	5	340.2	946.4	710.0	229.5	719.0	701.2	843.4
DSLNT	G2	PRE-TERM	2	5	460.2	843.2	601.8	155.8	557.9	486.4	661.2
DSLNT	G2	PRE-TERM	3	5	433.2	786.4	567.4	132.6	555.3	503.0	559.2
DSLNT	G2	PRE-TERM	4	5	380.6	586.5	511.3	78.71	522.6	511.9	554.9
DSLNT	G2	PRE-TERM	5	5	383.5	590.1	447.7	82.57	420.6	401.6	443.0
DSLNT	G2	PRE-TERM	6	5	344.4	579.5	430.7	89.21	411.8	387.2	430.6
DSLNT	G2	PRE-TERM	7	5	264.8	677.8	383.9	168.1	312.6	300.0	364.4
DSLNT	G2	PRE-TERM	8	5	226.1	364.8	275.5	53.26	259.6	249.6	277.2
DSLNT	G2	PRE-TERM	10	4	158.8	256.3	207.4	44.63	207.3	176.6	238.2
DSLNT	G2	PRE-TERM	12	5	131.9	452.7	224.4	134.7	160.9	136.9	239.5
DSLNT	G2	PRE-TERM	14	5	131.9	321.5	201.0	77.40	176.5	144.4	230.5
DSLNT	G2	PRE-TERM	16	3	118.9	459.9	263.6	176.2	212.0	165.5	335.9
DSLNT	G2	TERM	1	5	262.5	540.0	368.0	119.3	304.3	288.7	444.5
DSLNT	G2	TERM	2	5	146.3	490.1	317.1	124.5	326.6	274.5	347.8

Table S2 Concentration of Human Milk Oligosaccharides in Term or Preterm Milk At Different Weeks Postpartum Separated By Milk Group

** When there are results below the method limit of quanfification (LoQ) the result has been assigned value of 0.5 × LoQ, hence the minimum value appears to be the same in many cases. When a large number of datapoints are below LoQ this can also have the effect that the median = minimum.*

					HMO Concentration (mg/L)						
HMO	Milk Group	Study Arm	Week Post Partum	N	min *	max	mean	sd	median *	Quartile 1 (25%)	Quartile 3 (75%)
DSLNT	G2	TERM	3	5	135.3	480.2	294.4	127.1	265.9	250.9	340.0
DSLNT	G2	TERM	4	5	129.5	332.1	237.6	90.24	224.7	175.1	326.4
DSLNT	G2	TERM	5	5	105.1	318.1	212.3	98.73	161.6	159.5	316.9
DSLNT	G2	TERM	6	5	48.35	312.0	180.7	121.0	150.1	90.80	302.1
DSLNT	G2	TERM	7	5	74.88	203.7	132.2	53.18	127.3	89.74	165.1
DSLNT	G2	TERM	8	5	69.77	224.3	134.3	67.70	115.3	77.83	184.6
DSLNT	G3	PRE-TERM	1	1	259.3	259.3	259.3	NA	259.3	259.3	259.3
DSLNT	G3	PRE-TERM	2	1	495.6	495.6	495.6	NA	495.6	495.6	495.6
DSLNT	G3	PRE-TERM	3	1	470.6	470.6	470.6	NA	470.6	470.6	470.6
DSLNT	G3	PRE-TERM	4	1	304.9	304.9	304.9	NA	304.9	304.9	304.9
DSLNT	G3	PRE-TERM	5	1	244.0	244.0	244.0	NA	244.0	244.0	244.0
DSLNT	G3	PRE-TERM	6	1	196.7	196.7	196.7	NA	196.7	196.7	196.7
DSLNT	G3	PRE-TERM	7	1	159.2	159.2	159.2	NA	159.2	159.2	159.2
DSLNT	G3	PRE-TERM	8	1	129.6	129.6	129.6	NA	129.6	129.6	129.6
DSLNT	G3	PRE-TERM	10	1	102.6	102.6	102.6	NA	102.6	102.6	102.6
DSLNT	G3	PRE-TERM	12	1	96.95	96.95	96.95	NA	96.95	96.95	96.95
DSLNT	G3	PRE-TERM	14	1	97.17	97.17	97.17	NA	97.17	97.17	97.17
DSLNT	G3	PRE-TERM	16	1	97.08	97.08	97.08	NA	97.08	97.08	97.08
DSLNT	G3	TERM	1	1	163.6	163.6	163.6	NA	163.6	163.6	163.6
DSLNT	G3	TERM	2	1	190.7	190.7	190.7	NA	190.7	190.7	190.7
DSLNT	G3	TERM	3	1	218.6	218.6	218.6	NA	218.6	218.6	218.6
DSLNT	G3	TERM	4	1	199.4	199.4	199.4	NA	199.4	199.4	199.4
DSLNT	G3	TERM	5	1	171.3	171.3	171.3	NA	171.3	171.3	171.3
DSLNT	G3	TERM	6	1	156.0	156.0	156.0	NA	156.0	156.0	156.0
DSLNT	G3	TERM	7	1	129.3	129.3	129.3	NA	129.3	129.3	129.3
DSLNT	G3	TERM	8	1	140.2	140.2	140.2	NA	140.2	140.2	140.2
DSLNT	G4	TERM	1	1	697.9	697.9	697.9	NA	697.9	697.9	697.9
DSLNT	G4	TERM	2	1	608.1	608.1	608.1	NA	608.1	608.1	608.1
DSLNT	G4	TERM	3	1	488.2	488.2	488.2	NA	488.2	488.2	488.2
DSLNT	G4	TERM	4	1	477.6	477.6	477.6	NA	477.6	477.6	477.6
DSLNT	G4	TERM	5	1	360.7	360.7	360.7	NA	360.7	360.7	360.7
DSLNT	G4	TERM	6	1	401.1	401.1	401.1	NA	401.1	401.1	401.1
DSLNT	G4	TERM	7	1	293.1	293.1	293.1	NA	293.1	293.1	293.1
DSLNT	G4	TERM	8	1	264.6	264.6	264.6	NA	264.6	264.6	264.6

Table S2 Concentration of Human Milk Oligosaccharides in Term or Preterm Milk At Different Weeks Postpartum Separated By Milk Group

** When there are results below the method limit of quanfification (LoQ) the result has been assigned value of 0.5 × LoQ, hence the minimum value appears to be the same in many cases. When a large number of datapoints are below LoQ this can also have the effect that the median = minimum.*

HMO	Milk Group	Study Arm	Week Post Partum	N	HMO Concentration (mg/L) min *	max	mean	sd	median *	Quartile 1 (25%)	Quartile 3 (75%)
LDFT	G1	PRE-TERM	1	19	22.50	1174	445.8	285.2	456.4	213.4	584.0
LDFT	G1	PRE-TERM	2	19	22.50	759.0	245.8	190.6	171.6	115.6	301.3
LDFT	G1	PRE-TERM	3	19	22.50	1926	340.4	460.1	197.0	152.0	261.6
LDFT	G1	PRE-TERM	4	18	22.50	2513	394.0	555.4	252.2	153.2	416.8
LDFT	G1	PRE-TERM	5	19	22.50	2072	376.0	453.4	286.5	187.8	356.9
LDFT	G1	PRE-TERM	6	18	22.50	987.2	370.1	269.9	239.6	183.1	562.7
LDFT	G1	PRE-TERM	7	18	22.50	2758	378.2	606.1	236.4	166.8	286.9
LDFT	G1	PRE-TERM	8	18	22.50	2333	461.3	631.6	204.2	176.8	331.9
LDFT	G1	PRE-TERM	10	18	22.50	1827	426.9	436.2	283.5	164.6	507.1
LDFT	G1	PRE-TERM	12	15	22.50	3003	624.6	808.7	366.3	241.0	497.7
LDFT	G1	PRE-TERM	14	15	22.50	1797	401.2	409.5	289.4	241.7	390.1
LDFT	G1	PRE-TERM	16	15	22.50	1825	519.7	464.6	417.4	269.6	545.9
LDFT	G1	TERM	1	21	111.6	983.9	444.1	256.5	390.4	287.5	617.8
LDFT	G1	TERM	2	19	76.98	732.1	327.6	176.5	281.0	223.5	414.1
LDFT	G1	TERM	3	21	89.15	965.0	331.2	224.1	315.4	207.6	337.2
LDFT	G1	TERM	4	21	105.9	549.3	257.8	115.5	230.6	190.0	302.0
LDFT	G1	TERM	5	21	130.3	1270	331.4	295.3	242.6	185.3	322.6
LDFT	G1	TERM	6	20	119.9	2907	390.6	596.8	285.2	194.7	325.8
LDFT	G1	TERM	7	20	126.0	1713	357.1	337.0	286.1	213.8	394.7
LDFT	G1	TERM	8	21	93.06	818.6	317.8	170.9	301.4	203.2	381.3
LDFT	G2	PRE-TERM	1	5	22.50	22.50	22.50	0.0000	22.50	22.50	22.50
LDFT	G2	PRE-TERM	2	5	22.50	22.50	22.50	0.0000	22.50	22.50	22.50
LDFT	G2	PRE-TERM	3	5	22.50	22.50	22.50	0.0000	22.50	22.50	22.50
LDFT	G2	PRE-TERM	4	5	22.50	22.50	22.50	0.0000	22.50	22.50	22.50
LDFT	G2	PRE-TERM	5	5	22.50	22.50	22.50	0.0000	22.50	22.50	22.50
LDFT	G2	PRE-TERM	6	5	22.50	22.50	22.50	0.0000	22.50	22.50	22.50
LDFT	G2	PRE-TERM	7	5	22.50	22.50	22.50	0.0000	22.50	22.50	22.50
LDFT	G2	PRE-TERM	8	5	22.50	22.50	22.50	0.0000	22.50	22.50	22.50
LDFT	G2	PRE-TERM	10	4	22.50	22.50	22.50	0.0000	22.50	22.50	22.50
LDFT	G2	PRE-TERM	12	5	22.50	22.50	22.50	0.0000	22.50	22.50	22.50
LDFT	G2	PRE-TERM	14	5	22.50	22.50	22.50	0.0000	22.50	22.50	22.50
LDFT	G2	PRE-TERM	16	3	22.50	22.50	22.50	0.0000	22.50	22.50	22.50
LDFT	G2	TERM	1	5	22.50	22.50	22.50	0.0000	22.50	22.50	22.50
LDFT	G2	TERM	2	5	22.50	22.50	22.50	0.0000	22.50	22.50	22.50

Table S2 Concentration of Human Milk Oligosaccharides in Term or Preterm Milk At Different Weeks Postpartum Separated By Milk Group

** When there are results below the method limit of quanfification (LoQ) the result has been assigned value of 0.5 × LoQ, hence the minimum value appears to be the same in many cases. When a large number of datapoints are below LoQ this can also have the effect that the median = minimum.*

					HMO Concentration (mg/L)						
HMO	Milk Group	Study Arm	Week Post Partum	N	min *	max	mean	sd	median *	Quartile 1 (25%)	Quartile 3 (75%)
LDFT	G2	TERM	3	5	22.50	22.50	22.50	0.0000	22.50	22.50	22.50
LDFT	G2	TERM	4	5	22.50	22.50	22.50	0.0000	22.50	22.50	22.50
LDFT	G2	TERM	5	5	22.50	22.50	22.50	0.0000	22.50	22.50	22.50
LDFT	G2	TERM	6	5	22.50	22.50	22.50	0.0000	22.50	22.50	22.50
LDFT	G2	TERM	7	5	22.50	22.50	22.50	0.0000	22.50	22.50	22.50
LDFT	G2	TERM	8	5	22.50	22.50	22.50	0.0000	22.50	22.50	22.50
LDFT	G3	PRE-TERM	1	1	56.66	56.66	56.66	NA	56.66	56.66	56.66
LDFT	G3	PRE-TERM	2	1	265.3	265.3	265.3	NA	265.3	265.3	265.3
LDFT	G3	PRE-TERM	3	1	135.2	135.2	135.2	NA	135.2	135.2	135.2
LDFT	G3	PRE-TERM	4	1	63.09	63.09	63.09	NA	63.09	63.09	63.09
LDFT	G3	PRE-TERM	5	1	91.93	91.93	91.93	NA	91.93	91.93	91.93
LDFT	G3	PRE-TERM	6	1	95.15	95.15	95.15	NA	95.15	95.15	95.15
LDFT	G3	PRE-TERM	7	1	77.50	77.50	77.50	NA	77.50	77.50	77.50
LDFT	G3	PRE-TERM	8	1	77.28	77.28	77.28	NA	77.28	77.28	77.28
LDFT	G3	PRE-TERM	10	1	76.44	76.44	76.44	NA	76.44	76.44	76.44
LDFT	G3	PRE-TERM	12	1	76.41	76.41	76.41	NA	76.41	76.41	76.41
LDFT	G3	PRE-TERM	14	1	91.69	91.69	91.69	NA	91.69	91.69	91.69
LDFT	G3	PRE-TERM	16	1	103.7	103.7	103.7	NA	103.7	103.7	103.7
LDFT	G3	TERM	1	1	71.16	71.16	71.16	NA	71.16	71.16	71.16
LDFT	G3	TERM	2	1	273.5	273.5	273.5	NA	273.5	273.5	273.5
LDFT	G3	TERM	3	1	136.6	136.6	136.6	NA	136.6	136.6	136.6
LDFT	G3	TERM	4	1	119.3	119.3	119.3	NA	119.3	119.3	119.3
LDFT	G3	TERM	5	1	61.93	61.93	61.93	NA	61.93	61.93	61.93
LDFT	G3	TERM	6	1	111.5	111.5	111.5	NA	111.5	111.5	111.5
LDFT	G3	TERM	7	1	68.95	68.95	68.95	NA	68.95	68.95	68.95
LDFT	G3	TERM	8	1	143.8	143.8	143.8	NA	143.8	143.8	143.8
LDFT	G4	TERM	1	1	22.50	22.50	22.50	NA	22.50	22.50	22.50
LDFT	G4	TERM	2	1	22.50	22.50	22.50	NA	22.50	22.50	22.50
LDFT	G4	TERM	3	1	22.50	22.50	22.50	NA	22.50	22.50	22.50
LDFT	G4	TERM	4	1	22.50	22.50	22.50	NA	22.50	22.50	22.50
LDFT	G4	TERM	5	1	22.50	22.50	22.50	NA	22.50	22.50	22.50
LDFT	G4	TERM	6	1	22.50	22.50	22.50	NA	22.50	22.50	22.50
LDFT	G4	TERM	7	1	22.50	22.50	22.50	NA	22.50	22.50	22.50
LDFT	G4	TERM	8	1	22.50	22.50	22.50	NA	22.50	22.50	22.50

Table S2 Concentration of Human Milk Oligosaccharides in Term or Preterm Milk At Different Weeks Postpartum Separated By Milk Group

** When there are results below the method limit of quanfification (LoQ) the result has been assigned value of 0.5 × LoQ, hence the minimum value appears to be the same in many cases. When a large number of datapoints are below LoQ this can also have the effect that the median = minimum.*

					HMO Concentration (mg/L)						
HMO	Milk Group	Study Arm	Week Post Partum	N	min *	max	mean	sd	median *	Quartile 1 (25%)	Quartile 3 (75%)
LNDFH-I	G1	PRE-TERM	1	19	81.36	1781	1276	427.3	1263	1063	1611
LNDFH-I	G1	PRE-TERM	2	19	75.44	1629	1058	375.6	1145	816.6	1299
LNDFH-I	G1	PRE-TERM	3	19	49.54	2269	1140	502.0	1047	865.0	1535
LNDFH-I	G1	PRE-TERM	4	18	40.53	3123	1159	643.6	961.2	870.4	1370
LNDFH-I	G1	PRE-TERM	5	19	41.75	3161	1192	651.0	1025	898.2	1512
LNDFH-I	G1	PRE-TERM	6	18	39.25	1624	1046	426.8	1051	816.4	1447
LNDFH-I	G1	PRE-TERM	7	18	36.61	1500	1006	364.2	1032	823.3	1260
LNDFH-I	G1	PRE-TERM	8	18	31.86	1910	1061	440.1	993.8	847.6	1226
LNDFH-I	G1	PRE-TERM	10	18	22.31	1663	970.8	378.4	927.2	777.2	1134
LNDFH-I	G1	PRE-TERM	12	15	18.66	1606	976.5	424.9	910.9	736.8	1272
LNDFH-I	G1	PRE-TERM	14	15	33.59	1768	888.4	393.7	887.0	629.6	1091
LNDFH-I	G1	PRE-TERM	16	15	34.15	1431	818.0	343.6	845.7	598.1	987.1
LNDFH-I	G1	TERM	1	21	825.9	2122	1256	405.9	1106	947.1	1565
LNDFH-I	G1	TERM	2	19	816.4	2264	1349	420.1	1220	1016	1638
LNDFH-I	G1	TERM	3	21	699.4	2313	1365	419.9	1380	1063	1649
LNDFH-I	G1	TERM	4	21	542.0	1937	1215	366.3	1246	1040	1367
LNDFH-I	G1	TERM	5	21	464.2	1838	1120	391.9	1146	827.0	1334
LNDFH-I	G1	TERM	6	20	458.7	2024	1113	430.6	1106	786.6	1313
LNDFH-I	G1	TERM	7	20	384.5	2167	1068	433.3	1036	773.2	1314
LNDFH-I	G1	TERM	8	21	5.000	1935	890.6	448.2	851.2	649.6	1064
LNDFH-I	G2	PRE-TERM	1	5	5.000	115.7	27.14	49.51	5.000	5.000	5.000
LNDFH-I	G2	PRE-TERM	2	5	5.000	65.99	17.20	27.27	5.000	5.000	5.000
LNDFH-I	G2	PRE-TERM	3	5	5.000	56.94	15.39	23.23	5.000	5.000	5.000
LNDFH-I	G2	PRE-TERM	4	5	5.000	22.95	8.589	8.025	5.000	5.000	5.000
LNDFH-I	G2	PRE-TERM	5	5	5.000	24.70	8.940	8.810	5.000	5.000	5.000
LNDFH-I	G2	PRE-TERM	6	5	5.000	22.52	8.505	7.836	5.000	5.000	5.000
LNDFH-I	G2	PRE-TERM	7	5	5.000	22.32	8.464	7.746	5.000	5.000	5.000
LNDFH-I	G2	PRE-TERM	8	5	5.000	21.68	8.336	7.460	5.000	5.000	5.000
LNDFH-I	G2	PRE-TERM	10	4	5.000	15.92	7.731	5.462	5.000	5.000	7.731
LNDFH-I	G2	PRE-TERM	12	5	5.000	14.75	6.949	4.359	5.000	5.000	5.000
LNDFH-I	G2	PRE-TERM	14	5	5.000	14.03	6.806	4.039	5.000	5.000	5.000
LNDFH-I	G2	PRE-TERM	16	3	5.000	11.93	7.311	4.002	5.000	5.000	8.466
LNDFH-I	G2	TERM	1	5	5.000	5.000	5.000	0.0000	5.000	5.000	5.000
LNDFH-I	G2	TERM	2	5	5.000	5.000	5.000	0.0000	5.000	5.000	5.000

Table S2 Concentration of Human Milk Oligosaccharides in Term or Preterm Milk At Different Weeks Postpartum Separated By Milk Group

** When there are results below the method limit of quanfification (LoQ) the result has been assigned value of 0.5 × LoQ, hence the minimum value appears to be the same in many cases. When a large number of datapoints are below LoQ this can also have the effect that the median = minimum.*

					HMO Concentration (mg/L)						
HMO	Milk Group	Study Arm	Week Post Partum	N	min *	max	mean	sd	median *	Quartile 1 (25%)	Quartile 3 (75%)
LNDFH-I	G2	TERM	3	5	5.000	5.000	5.000	0.0000	5.000	5.000	5.000
LNDFH-I	G2	TERM	4	5	5.000	15.85	7.171	4.853	5.000	5.000	5.000
LNDFH-I	G2	TERM	5	5	5.000	11.89	6.378	3.082	5.000	5.000	5.000
LNDFH-I	G2	TERM	6	5	5.000	5.000	5.000	0.0000	5.000	5.000	5.000
LNDFH-I	G2	TERM	7	5	5.000	5.000	5.000	0.0000	5.000	5.000	5.000
LNDFH-I	G2	TERM	8	5	5.000	5.000	5.000	0.0000	5.000	5.000	5.000
LNDFH-I	G3	PRE-TERM	1	1	5.000	5.000	5.000	NA	5.000	5.000	5.000
LNDFH-I	G3	PRE-TERM	2	1	5.000	5.000	5.000	NA	5.000	5.000	5.000
LNDFH-I	G3	PRE-TERM	3	1	5.000	5.000	5.000	NA	5.000	5.000	5.000
LNDFH-I	G3	PRE-TERM	4	1	5.000	5.000	5.000	NA	5.000	5.000	5.000
LNDFH-I	G3	PRE-TERM	5	1	5.000	5.000	5.000	NA	5.000	5.000	5.000
LNDFH-I	G3	PRE-TERM	6	1	5.000	5.000	5.000	NA	5.000	5.000	5.000
LNDFH-I	G3	PRE-TERM	7	1	5.000	5.000	5.000	NA	5.000	5.000	5.000
LNDFH-I	G3	PRE-TERM	8	1	5.000	5.000	5.000	NA	5.000	5.000	5.000
LNDFH-I	G3	PRE-TERM	10	1	5.000	5.000	5.000	NA	5.000	5.000	5.000
LNDFH-I	G3	PRE-TERM	12	1	5.000	5.000	5.000	NA	5.000	5.000	5.000
LNDFH-I	G3	PRE-TERM	14	1	5.000	5.000	5.000	NA	5.000	5.000	5.000
LNDFH-I	G3	PRE-TERM	16	1	5.000	5.000	5.000	NA	5.000	5.000	5.000
LNDFH-I	G3	TERM	1	1	5.000	5.000	5.000	NA	5.000	5.000	5.000
LNDFH-I	G3	TERM	2	1	5.000	5.000	5.000	NA	5.000	5.000	5.000
LNDFH-I	G3	TERM	3	1	5.000	5.000	5.000	NA	5.000	5.000	5.000
LNDFH-I	G3	TERM	4	1	5.000	5.000	5.000	NA	5.000	5.000	5.000
LNDFH-I	G3	TERM	5	1	5.000	5.000	5.000	NA	5.000	5.000	5.000
LNDFH-I	G3	TERM	6	1	5.000	5.000	5.000	NA	5.000	5.000	5.000
LNDFH-I	G3	TERM	7	1	5.000	5.000	5.000	NA	5.000	5.000	5.000
LNDFH-I	G3	TERM	8	1	5.000	5.000	5.000	NA	5.000	5.000	5.000
LNDFH-I	G4	TERM	1	1	5.000	5.000	5.000	NA	5.000	5.000	5.000
LNDFH-I	G4	TERM	2	1	5.000	5.000	5.000	NA	5.000	5.000	5.000
LNDFH-I	G4	TERM	3	1	5.000	5.000	5.000	NA	5.000	5.000	5.000
LNDFH-I	G4	TERM	4	1	5.000	5.000	5.000	NA	5.000	5.000	5.000
LNDFH-I	G4	TERM	5	1	5.000	5.000	5.000	NA	5.000	5.000	5.000
LNDFH-I	G4	TERM	6	1	5.000	5.000	5.000	NA	5.000	5.000	5.000
LNDFH-I	G4	TERM	7	1	5.000	5.000	5.000	NA	5.000	5.000	5.000
LNDFH-I	G4	TERM	8	1	5.000	5.000	5.000	NA	5.000	5.000	5.000

Table S2 Concentration of Human Milk Oligosaccharides in Term or Preterm Milk At Different Weeks Postpartum Separated By Milk Group

** When there are results below the method limit of quanfification (LoQ) the result has been assigned value of 0.5 × LoQ, hence the minimum value appears to be the same in many cases. When a large number of datapoints are below LoQ this can also have the effect that the median = minimum.*

					HMO Concentration (mg/L)						
HMO	Milk Group	Study Arm	Week Post Partum	N	min *	max	mean	sd	median *	Quartile 1 (25%)	Quartile 3 (75%)
LNFP-I	G1	PRE-TERM	1	19	13.50	2806	1617	672.0	1661	1282	2016
LNFP-I	G1	PRE-TERM	2	19	13.50	1949	1172	451.3	1203	937.4	1449
LNFP-I	G1	PRE-TERM	3	19	13.50	1634	1034	404.5	1085	727.2	1358
LNFP-I	G1	PRE-TERM	4	18	13.50	1549	897.8	347.8	937.5	673.4	1063
LNFP-I	G1	PRE-TERM	5	19	13.50	1443	837.2	374.3	780.4	604.2	1140
LNFP-I	G1	PRE-TERM	6	18	13.50	1840	755.9	403.8	693.0	576.3	953.7
LNFP-I	G1	PRE-TERM	7	18	13.50	1773	701.0	382.7	619.3	496.0	862.9
LNFP-I	G1	PRE-TERM	8	18	13.50	1123	648.6	279.0	596.5	463.7	832.2
LNFP-I	G1	PRE-TERM	10	18	13.50	1436	547.2	296.7	484.2	385.0	660.6
LNFP-I	G1	PRE-TERM	12	15	13.50	966.6	441.9	263.5	404.8	259.1	540.9
LNFP-I	G1	PRE-TERM	14	15	13.50	1014	447.6	272.8	393.4	287.2	622.3
LNFP-I	G1	PRE-TERM	16	15	13.50	933.6	368.2	256.2	299.3	181.3	540.5
LNFP-I	G1	TERM	1	21	685.4	3597	2207	750.6	2218	1768	2618
LNFP-I	G1	TERM	2	19	505.8	3073	1704	667.2	1613	1291	1926
LNFP-I	G1	TERM	3	21	492.7	3018	1518	649.8	1369	1055	1841
LNFP-I	G1	TERM	4	21	418.7	3156	1325	686.4	1198	831.7	1715
LNFP-I	G1	TERM	5	21	290.2	2655	1003	532.7	942.2	710.6	1138
LNFP-I	G1	TERM	6	20	231.4	2045	841.2	400.2	760.5	682.3	942.5
LNFP-I	G1	TERM	7	20	168.8	2617	796.7	542.2	702.6	547.6	805.5
LNFP-I	G1	TERM	8	21	192.0	1956	688.9	456.6	530.6	416.9	834.1
LNFP-I	G2	PRE-TERM	1	5	13.50	13.50	13.50	0.0000	13.50	13.50	13.50
LNFP-I	G2	PRE-TERM	2	5	13.50	13.50	13.50	0.0000	13.50	13.50	13.50
LNFP-I	G2	PRE-TERM	3	5	13.50	13.50	13.50	0.0000	13.50	13.50	13.50
LNFP-I	G2	PRE-TERM	4	5	13.50	13.50	13.50	0.0000	13.50	13.50	13.50
LNFP-I	G2	PRE-TERM	5	5	13.50	13.50	13.50	0.0000	13.50	13.50	13.50
LNFP-I	G2	PRE-TERM	6	5	13.50	13.50	13.50	0.0000	13.50	13.50	13.50
LNFP-I	G2	PRE-TERM	7	5	13.50	13.50	13.50	0.0000	13.50	13.50	13.50
LNFP-I	G2	PRE-TERM	8	5	13.50	13.50	13.50	0.0000	13.50	13.50	13.50
LNFP-I	G2	PRE-TERM	10	4	13.50	13.50	13.50	0.0000	13.50	13.50	13.50
LNFP-I	G2	PRE-TERM	12	5	13.50	13.50	13.50	0.0000	13.50	13.50	13.50
LNFP-I	G2	PRE-TERM	14	5	13.50	13.50	13.50	0.0000	13.50	13.50	13.50
LNFP-I	G2	PRE-TERM	16	3	13.50	13.50	13.50	0.0000	13.50	13.50	13.50
LNFP-I	G2	TERM	1	5	13.50	13.50	13.50	0.0000	13.50	13.50	13.50
LNFP-I	G2	TERM	2	5	13.50	13.50	13.50	0.0000	13.50	13.50	13.50

Table S2 Concentration of Human Milk Oligosaccharides in Term or Preterm Milk At Different Weeks Postpartum Separated By Milk Group

** When there are results below the method limit of quanfification (LoQ) the result has been assigned value of 0.5 × LoQ, hence the minimum value appears to be the same in many cases. When a large number of datapoints are below LoQ this can also have the effect that the median = minimum.*

					HMO Concentration (mg/L)						
HMO	Milk Group	Study Arm	Week Post Partum	N	min *	max	mean	sd	median *	Quartile 1 (25%)	Quartile 3 (75%)
LNFP-I	G2	TERM	3	5	13.50	13.50	13.50	0.0000	13.50	13.50	13.50
LNFP-I	G2	TERM	4	5	13.50	13.50	13.50	0.0000	13.50	13.50	13.50
LNFP-I	G2	TERM	5	5	13.50	13.50	13.50	0.0000	13.50	13.50	13.50
LNFP-I	G2	TERM	6	5	13.50	13.50	13.50	0.0000	13.50	13.50	13.50
LNFP-I	G2	TERM	7	5	13.50	13.50	13.50	0.0000	13.50	13.50	13.50
LNFP-I	G2	TERM	8	5	13.50	13.50	13.50	0.0000	13.50	13.50	13.50
LNFP-I	G3	PRE-TERM	1	1	2639	2639	2639	NA	2639	2639	2639
LNFP-I	G3	PRE-TERM	2	1	2195	2195	2195	NA	2195	2195	2195
LNFP-I	G3	PRE-TERM	3	1	2178	2178	2178	NA	2178	2178	2178
LNFP-I	G3	PRE-TERM	4	1	2062	2062	2062	NA	2062	2062	2062
LNFP-I	G3	PRE-TERM	5	1	1624	1624	1624	NA	1624	1624	1624
LNFP-I	G3	PRE-TERM	6	1	1332	1332	1332	NA	1332	1332	1332
LNFP-I	G3	PRE-TERM	7	1	949.2	949.2	949.2	NA	949.2	949.2	949.2
LNFP-I	G3	PRE-TERM	8	1	960.9	960.9	960.9	NA	960.9	960.9	960.9
LNFP-I	G3	PRE-TERM	10	1	807.3	807.3	807.3	NA	807.3	807.3	807.3
LNFP-I	G3	PRE-TERM	12	1	829.3	829.3	829.3	NA	829.3	829.3	829.3
LNFP-I	G3	PRE-TERM	14	1	829.0	829.0	829.0	NA	829.0	829.0	829.0
LNFP-I	G3	PRE-TERM	16	1	784.9	784.9	784.9	NA	784.9	784.9	784.9
LNFP-I	G3	TERM	1	1	2392	2392	2392	NA	2392	2392	2392
LNFP-I	G3	TERM	2	1	1768	1768	1768	NA	1768	1768	1768
LNFP-I	G3	TERM	3	1	2027	2027	2027	NA	2027	2027	2027
LNFP-I	G3	TERM	4	1	1812	1812	1812	NA	1812	1812	1812
LNFP-I	G3	TERM	5	1	1850	1850	1850	NA	1850	1850	1850
LNFP-I	G3	TERM	6	1	1480	1480	1480	NA	1480	1480	1480
LNFP-I	G3	TERM	7	1	1320	1320	1320	NA	1320	1320	1320
LNFP-I	G3	TERM	8	1	1180	1180	1180	NA	1180	1180	1180
LNFP-I	G4	TERM	1	1	13.50	13.50	13.50	NA	13.50	13.50	13.50
LNFP-I	G4	TERM	2	1	13.50	13.50	13.50	NA	13.50	13.50	13.50
LNFP-I	G4	TERM	3	1	13.50	13.50	13.50	NA	13.50	13.50	13.50
LNFP-I	G4	TERM	4	1	13.50	13.50	13.50	NA	13.50	13.50	13.50
LNFP-I	G4	TERM	5	1	13.50	13.50	13.50	NA	13.50	13.50	13.50
LNFP-I	G4	TERM	6	1	13.50	13.50	13.50	NA	13.50	13.50	13.50
LNFP-I	G4	TERM	7	1	13.50	13.50	13.50	NA	13.50	13.50	13.50
LNFP-I	G4	TERM	8	1	13.50	13.50	13.50	NA	13.50	13.50	13.50

Table S2 Concentration of Human Milk Oligosaccharides in Term or Preterm Milk At Different Weeks Postpartum Separated By Milk Group

** When there are results below the method limit of quanfification (LoQ) the result has been assigned value of 0.5 × LoQ, hence the minimum value appears to be the same in many cases. When a large number of datapoints are below LoQ this can also have the effect that the median = minimum.*

					HMO Concentration (mg/L)						
HMO	Milk Group	Study Arm	Week Post Partum	N	min *	max	mean	sd	median *	Quartile 1 (25%)	Quartile 3 (75%)
LNFP-II	G1	PRE-TERM	1	19	95.46	1108	293.7	248.1	189.9	135.4	383.2
LNFP-II	G1	PRE-TERM	2	19	136.4	1108	379.6	261.9	293.7	188.3	487.4
LNFP-II	G1	PRE-TERM	3	19	135.6	975.6	407.0	246.7	327.7	226.2	559.4
LNFP-II	G1	PRE-TERM	4	18	122.1	918.9	405.9	240.6	325.1	225.1	545.1
LNFP-II	G1	PRE-TERM	5	19	138.0	865.6	410.7	227.8	389.4	211.6	556.6
LNFP-II	G1	PRE-TERM	6	18	68.91	946.4	378.0	254.2	330.9	171.4	521.7
LNFP-II	G1	PRE-TERM	7	18	128.1	982.3	432.1	270.2	367.5	175.7	545.8
LNFP-II	G1	PRE-TERM	8	18	146.0	911.4	414.8	252.8	353.4	210.9	516.4
LNFP-II	G1	PRE-TERM	10	18	92.35	760.4	382.7	219.2	307.0	213.2	533.1
LNFP-II	G1	PRE-TERM	12	15	132.9	688.8	396.3	185.6	416.9	224.7	548.1
LNFP-II	G1	PRE-TERM	14	15	121.9	754.0	388.4	198.0	369.5	232.4	503.6
LNFP-II	G1	PRE-TERM	16	15	118.0	1014	362.4	230.6	302.2	218.0	398.9
LNFP-II	G1	TERM	1	21	55.63	1204	245.0	235.3	222.1	122.8	266.2
LNFP-II	G1	TERM	2	19	137.2	640.6	333.2	156.0	311.2	217.0	416.4
LNFP-II	G1	TERM	3	21	147.7	686.4	341.6	168.9	308.5	221.8	394.7
LNFP-II	G1	TERM	4	21	127.0	1531	400.8	303.5	327.6	244.3	458.3
LNFP-II	G1	TERM	5	21	137.6	718.3	325.7	166.2	289.6	186.1	431.9
LNFP-II	G1	TERM	6	20	109.8	824.7	344.6	190.6	304.1	199.9	422.2
LNFP-II	G1	TERM	7	20	126.2	846.5	348.6	201.2	290.9	203.2	429.4
LNFP-II	G1	TERM	8	21	96.47	854.5	311.1	200.1	272.1	173.9	382.2
LNFP-II	G2	PRE-TERM	1	5	1328	2094	1593	314.1	1435	1402	1703
LNFP-II	G2	PRE-TERM	2	5	1006	1950	1634	370.7	1740	1628	1845
LNFP-II	G2	PRE-TERM	3	5	947.4	1922	1579	382.0	1736	1514	1775
LNFP-II	G2	PRE-TERM	4	5	930.8	2220	1512	528.0	1667	1044	1696
LNFP-II	G2	PRE-TERM	5	5	897.4	1775	1366	383.2	1537	1023	1597
LNFP-II	G2	PRE-TERM	6	5	791.2	2138	1528	584.9	1607	1079	2024
LNFP-II	G2	PRE-TERM	7	5	774.2	2116	1409	586.6	1163	1050	1942
LNFP-II	G2	PRE-TERM	8	5	730.6	1804	1205	427.2	1039	983.3	1468
LNFP-II	G2	PRE-TERM	10	4	656.5	1432	1004	365.3	964.1	725.1	1243
LNFP-II	G2	PRE-TERM	12	5	630.5	2373	1177	779.3	668.0	643.6	1571
LNFP-II	G2	PRE-TERM	14	5	483.6	1476	942.4	451.9	784.2	601.3	1367
LNFP-II	G2	PRE-TERM	16	3	561.9	2593	1452	1039	1201	881.4	1897
LNFP-II	G2	TERM	1	5	1069	1616	1290	206.7	1238	1186	1339
LNFP-II	G2	TERM	2	5	901.1	1884	1447	377.0	1468	1296	1684

Table S2 Concentration of Human Milk Oligosaccharides in Term or Preterm Milk At Different Weeks Postpartum Separated By Milk Group

** When there are results below the method limit of quanfification (LoQ) the result has been assigned value of 0.5 × LoQ, hence the minimum value appears to be the same in many cases. When a large number of datapoints are below LoQ this can also have the effect that the median = minimum.*

					HMO Concentration (mg/L)						
HMO	Milk Group	Study Arm	Week Post Partum	N	min *	max	mean	sd	median *	Quartile 1 (25%)	Quartile 3 (75%)
LNFP-II	G2	TERM	3	5	884.6	1672	1399	334.8	1577	1238	1626
LNFP-II	G2	TERM	4	5	947.7	1565	1285	221.0	1310	1268	1332
LNFP-II	G2	TERM	5	5	964.1	1411	1180	165.8	1134	1133	1257
LNFP-II	G2	TERM	6	5	376.0	1476	981.4	414.9	982.8	847.4	1225
LNFP-II	G2	TERM	7	5	530.4	1171	881.4	266.6	923.5	697.1	1085
LNFP-II	G2	TERM	8	5	597.7	1272	940.4	305.3	1038	640.1	1155
LNFP-II	G3	PRE-TERM	1	1	17.50	17.50	17.50	NA	17.50	17.50	17.50
LNFP-II	G3	PRE-TERM	2	1	17.50	17.50	17.50	NA	17.50	17.50	17.50
LNFP-II	G3	PRE-TERM	3	1	17.50	17.50	17.50	NA	17.50	17.50	17.50
LNFP-II	G3	PRE-TERM	4	1	17.50	17.50	17.50	NA	17.50	17.50	17.50
LNFP-II	G3	PRE-TERM	5	1	17.50	17.50	17.50	NA	17.50	17.50	17.50
LNFP-II	G3	PRE-TERM	6	1	17.50	17.50	17.50	NA	17.50	17.50	17.50
LNFP-II	G3	PRE-TERM	7	1	17.50	17.50	17.50	NA	17.50	17.50	17.50
LNFP-II	G3	PRE-TERM	8	1	17.50	17.50	17.50	NA	17.50	17.50	17.50
LNFP-II	G3	PRE-TERM	10	1	17.50	17.50	17.50	NA	17.50	17.50	17.50
LNFP-II	G3	PRE-TERM	12	1	17.50	17.50	17.50	NA	17.50	17.50	17.50
LNFP-II	G3	PRE-TERM	14	1	17.50	17.50	17.50	NA	17.50	17.50	17.50
LNFP-II	G3	PRE-TERM	16	1	17.50	17.50	17.50	NA	17.50	17.50	17.50
LNFP-II	G3	TERM	1	1	17.50	17.50	17.50	NA	17.50	17.50	17.50
LNFP-II	G3	TERM	2	1	17.50	17.50	17.50	NA	17.50	17.50	17.50
LNFP-II	G3	TERM	3	1	17.50	17.50	17.50	NA	17.50	17.50	17.50
LNFP-II	G3	TERM	4	1	17.50	17.50	17.50	NA	17.50	17.50	17.50
LNFP-II	G3	TERM	5	1	17.50	17.50	17.50	NA	17.50	17.50	17.50
LNFP-II	G3	TERM	6	1	17.50	17.50	17.50	NA	17.50	17.50	17.50
LNFP-II	G3	TERM	7	1	17.50	17.50	17.50	NA	17.50	17.50	17.50
LNFP-II	G3	TERM	8	1	17.50	17.50	17.50	NA	17.50	17.50	17.50
LNFP-II	G4	TERM	1	1	17.50	17.50	17.50	NA	17.50	17.50	17.50
LNFP-II	G4	TERM	2	1	17.50	17.50	17.50	NA	17.50	17.50	17.50
LNFP-II	G4	TERM	3	1	17.50	17.50	17.50	NA	17.50	17.50	17.50
LNFP-II	G4	TERM	4	1	17.50	17.50	17.50	NA	17.50	17.50	17.50
LNFP-II	G4	TERM	5	1	17.50	17.50	17.50	NA	17.50	17.50	17.50
LNFP-II	G4	TERM	6	1	17.50	17.50	17.50	NA	17.50	17.50	17.50
LNFP-II	G4	TERM	7	1	17.50	17.50	17.50	NA	17.50	17.50	17.50
LNFP-II	G4	TERM	8	1	17.50	17.50	17.50	NA	17.50	17.50	17.50

Table S2 Concentration of Human Milk Oligosaccharides in Term or Preterm Milk At Different Weeks Postpartum Separated By Milk Group

** When there are results below the method limit of quanfification (LoQ) the result has been assigned value of 0.5 × LoQ, hence the minimum value appears to be the same in many cases. When a large number of datapoints are below LoQ this can also have the effect that the median = minimum.*

					HMO Concentration (mg/L)						
HMO	Milk Group	Study Arm	Week Post Partum	N	min *	max	mean	sd	median *	Quartile 1 (25%)	Quartile 3 (75%)
LNFP-III	G1	PRE-TERM	1	19	55.72	407.8	285.7	108.9	314.2	203.7	386.6
LNFP-III	G1	PRE-TERM	2	19	35.70	464.9	246.6	107.7	276.7	170.0	316.8
LNFP-III	G1	PRE-TERM	3	19	40.94	480.4	248.2	107.9	254.7	201.2	283.9
LNFP-III	G1	PRE-TERM	4	18	49.93	504.7	266.6	116.7	277.1	186.4	331.8
LNFP-III	G1	PRE-TERM	5	19	52.14	506.1	282.8	115.7	287.8	217.0	362.3
LNFP-III	G1	PRE-TERM	6	18	49.25	491.8	297.5	132.4	308.2	206.3	398.6
LNFP-III	G1	PRE-TERM	7	18	17.50	615.4	317.8	157.1	312.6	220.6	380.4
LNFP-III	G1	PRE-TERM	8	18	52.97	847.4	346.2	175.6	308.5	249.0	404.4
LNFP-III	G1	PRE-TERM	10	18	60.20	707.5	349.6	159.4	332.1	260.2	430.9
LNFP-III	G1	PRE-TERM	12	15	50.94	653.0	380.2	172.9	385.3	261.5	450.0
LNFP-III	G1	PRE-TERM	14	15	74.13	599.0	345.2	154.7	352.5	218.6	445.6
LNFP-III	G1	PRE-TERM	16	15	89.27	774.0	346.7	185.3	309.2	226.2	448.0
LNFP-III	G1	TERM	1	21	178.7	490.3	357.3	99.16	366.7	309.8	444.4
LNFP-III	G1	TERM	2	19	163.3	459.1	305.0	84.84	307.3	237.5	351.5
LNFP-III	G1	TERM	3	21	153.7	394.7	262.0	63.97	247.7	216.6	298.0
LNFP-III	G1	TERM	4	21	153.3	485.4	262.3	90.11	235.1	202.4	313.8
LNFP-III	G1	TERM	5	21	165.5	438.5	274.5	84.92	269.2	222.4	294.9
LNFP-III	G1	TERM	6	20	17.50	575.0	293.0	117.3	293.5	213.0	328.7
LNFP-III	G1	TERM	7	20	191.4	722.3	316.5	125.4	270.2	236.1	361.7
LNFP-III	G1	TERM	8	21	108.0	526.2	298.4	103.0	280.7	229.6	347.0
LNFP-III	G2	PRE-TERM	1	5	416.8	713.1	580.6	119.9	558.9	532.2	682.1
LNFP-III	G2	PRE-TERM	2	5	331.9	779.2	565.0	165.5	569.3	504.4	640.0
LNFP-III	G2	PRE-TERM	3	5	365.1	733.6	525.5	160.4	465.2	409.1	654.7
LNFP-III	G2	PRE-TERM	4	5	251.7	727.3	436.3	181.4	426.4	324.2	451.6
LNFP-III	G2	PRE-TERM	5	5	230.6	742.0	465.7	185.5	463.0	394.8	498.3
LNFP-III	G2	PRE-TERM	6	5	209.6	543.6	417.4	134.0	445.5	371.8	516.6
LNFP-III	G2	PRE-TERM	7	5	255.7	711.8	404.9	183.6	328.4	296.2	432.6
LNFP-III	G2	PRE-TERM	8	5	237.8	767.7	400.0	226.1	273.4	251.4	469.7
LNFP-III	G2	PRE-TERM	10	4	243.5	526.8	357.7	121.5	330.3	290.5	397.5
LNFP-III	G2	PRE-TERM	12	5	238.6	616.5	421.9	166.2	472.4	259.4	522.3
LNFP-III	G2	PRE-TERM	14	5	228.7	750.3	431.2	217.5	352.7	271.7	552.8
LNFP-III	G2	PRE-TERM	16	3	214.5	354.6	304.9	78.36	345.5	280.0	350.0
LNFP-III	G2	TERM	1	5	76.48	826.9	535.2	286.3	611.8	474.8	685.9
LNFP-III	G2	TERM	2	5	388.9	590.7	482.8	71.78	479.1	471.4	484.1

Table S2 Concentration of Human Milk Oligosaccharides in Term or Preterm Milk At Different Weeks Postpartum Separated By Milk Group

** When there are results below the method limit of quanfification (LoQ) the result has been assigned value of 0.5 × LoQ, hence the minimum value appears to be the same in many cases. When a large number of datapoints are below LoQ this can also have the effect that the median = minimum.*

					HMO Concentration (mg/L)						
HMO	Milk Group	Study Arm	Week Post Partum	N	min *	max	mean	sd	median *	Quartile 1 (25%)	Quartile 3 (75%)
LNFP-III	G2	TERM	3	5	318.0	515.2	381.8	80.55	339.0	337.6	399.3
LNFP-III	G2	TERM	4	5	273.1	525.0	347.9	103.9	294.0	290.7	356.7
LNFP-III	G2	TERM	5	5	247.7	494.3	351.8	92.86	336.1	302.7	378.4
LNFP-III	G2	TERM	6	5	147.3	499.8	373.0	133.3	395.6	394.5	428.0
LNFP-III	G2	TERM	7	5	280.5	461.6	345.7	69.91	319.1	312.8	354.7
LNFP-III	G2	TERM	8	5	272.6	493.6	364.3	83.62	368.3	312.1	375.0
LNFP-III	G3	PRE-TERM	1	1	322.8	322.8	322.8	NA	322.8	322.8	322.8
LNFP-III	G3	PRE-TERM	2	1	418.0	418.0	418.0	NA	418.0	418.0	418.0
LNFP-III	G3	PRE-TERM	3	1	352.3	352.3	352.3	NA	352.3	352.3	352.3
LNFP-III	G3	PRE-TERM	4	1	290.1	290.1	290.1	NA	290.1	290.1	290.1
LNFP-III	G3	PRE-TERM	5	1	324.2	324.2	324.2	NA	324.2	324.2	324.2
LNFP-III	G3	PRE-TERM	6	1	430.5	430.5	430.5	NA	430.5	430.5	430.5
LNFP-III	G3	PRE-TERM	7	1	334.1	334.1	334.1	NA	334.1	334.1	334.1
LNFP-III	G3	PRE-TERM	8	1	340.3	340.3	340.3	NA	340.3	340.3	340.3
LNFP-III	G3	PRE-TERM	10	1	329.7	329.7	329.7	NA	329.7	329.7	329.7
LNFP-III	G3	PRE-TERM	12	1	357.3	357.3	357.3	NA	357.3	357.3	357.3
LNFP-III	G3	PRE-TERM	14	1	404.7	404.7	404.7	NA	404.7	404.7	404.7
LNFP-III	G3	PRE-TERM	16	1	407.3	407.3	407.3	NA	407.3	407.3	407.3
LNFP-III	G3	TERM	1	1	329.2	329.2	329.2	NA	329.2	329.2	329.2
LNFP-III	G3	TERM	2	1	262.9	262.9	262.9	NA	262.9	262.9	262.9
LNFP-III	G3	TERM	3	1	219.7	219.7	219.7	NA	219.7	219.7	219.7
LNFP-III	G3	TERM	4	1	238.9	238.9	238.9	NA	238.9	238.9	238.9
LNFP-III	G3	TERM	5	1	202.2	202.2	202.2	NA	202.2	202.2	202.2
LNFP-III	G3	TERM	6	1	196.3	196.3	196.3	NA	196.3	196.3	196.3
LNFP-III	G3	TERM	7	1	190.9	190.9	190.9	NA	190.9	190.9	190.9
LNFP-III	G3	TERM	8	1	262.5	262.5	262.5	NA	262.5	262.5	262.5
LNFP-III	G4	TERM	1	1	1268	1268	1268	NA	1268	1268	1268
LNFP-III	G4	TERM	2	1	1169	1169	1169	NA	1169	1169	1169
LNFP-III	G4	TERM	3	1	911.0	911.0	911.0	NA	911.0	911.0	911.0
LNFP-III	G4	TERM	4	1	823.9	823.9	823.9	NA	823.9	823.9	823.9
LNFP-III	G4	TERM	5	1	714.0	714.0	714.0	NA	714.0	714.0	714.0
LNFP-III	G4	TERM	6	1	786.1	786.1	786.1	NA	786.1	786.1	786.1
LNFP-III	G4	TERM	7	1	703.6	703.6	703.6	NA	703.6	703.6	703.6
LNFP-III	G4	TERM	8	1	569.7	569.7	569.7	NA	569.7	569.7	569.7

Table S2 Concentration of Human Milk Oligosaccharides in Term or Preterm Milk At Different Weeks Postpartum Separated By Milk Group

** When there are results below the method limit of quanfification (LoQ) the result has been assigned value of 0.5 × LoQ, hence the minimum value appears to be the same in many cases. When a large number of datapoints are below LoQ this can also have the effect that the median = minimum.*

					HMO Concentration (mg/L)						
HMO	Milk Group	Study Arm	Week Post Partum	N	min *	max	mean	sd	median *	Quartile 1 (25%)	Quartile 3 (75%)
LNFP-V	G1	PRE-TERM	1	19	12.00	140.4	32.14	31.53	12.00	12.00	42.53
LNFP-V	G1	PRE-TERM	2	19	12.00	140.5	42.15	35.84	29.30	12.00	53.09
LNFP-V	G1	PRE-TERM	3	19	12.00	123.0	48.87	32.34	40.14	25.52	63.99
LNFP-V	G1	PRE-TERM	4	18	12.00	112.6	47.00	32.65	37.61	16.38	64.85
LNFP-V	G1	PRE-TERM	5	19	12.00	108.5	50.49	31.95	43.41	18.59	72.87
LNFP-V	G1	PRE-TERM	6	18	12.00	123.1	50.17	34.79	47.72	16.07	73.04
LNFP-V	G1	PRE-TERM	7	18	12.00	121.4	52.68	36.62	46.71	15.27	83.60
LNFP-V	G1	PRE-TERM	8	18	12.00	116.0	51.24	33.27	42.41	25.94	71.18
LNFP-V	G1	PRE-TERM	10	18	12.00	98.71	43.85	31.74	32.45	12.00	77.27
LNFP-V	G1	PRE-TERM	12	15	12.00	83.16	43.61	26.57	50.05	12.00	65.23
LNFP-V	G1	PRE-TERM	14	15	12.00	85.62	44.40	24.05	42.09	27.28	62.68
LNFP-V	G1	PRE-TERM	16	15	12.00	122.5	39.88	28.86	36.51	19.79	49.86
LNFP-V	G1	TERM	1	21	12.00	72.60	27.76	20.15	12.00	12.00	41.54
LNFP-V	G1	TERM	2	19	12.00	73.68	38.91	19.24	39.71	24.86	53.26
LNFP-V	G1	TERM	3	21	12.00	349.1	56.05	70.23	43.48	28.92	59.92
LNFP-V	G1	TERM	4	21	12.00	83.09	39.89	19.90	38.63	25.59	54.57
LNFP-V	G1	TERM	5	21	12.00	84.50	41.00	21.80	35.67	25.64	55.00
LNFP-V	G1	TERM	6	20	12.00	94.30	44.36	25.10	41.17	27.62	58.07
LNFP-V	G1	TERM	7	20	12.00	105.3	42.55	26.20	35.82	23.88	61.93
LNFP-V	G1	TERM	8	21	12.00	101.5	34.60	25.52	32.68	12.00	54.87
LNFP-V	G2	PRE-TERM	1	5	191.2	320.9	254.7	54.02	233.8	227.2	300.2
LNFP-V	G2	PRE-TERM	2	5	163.8	317.8	262.9	60.19	289.0	252.0	292.2
LNFP-V	G2	PRE-TERM	3	5	154.2	309.0	261.9	62.06	286.3	266.9	292.9
LNFP-V	G2	PRE-TERM	4	5	179.9	363.8	260.5	74.28	261.9	200.4	296.5
LNFP-V	G2	PRE-TERM	5	5	172.5	301.0	227.1	55.71	211.9	181.9	268.3
LNFP-V	G2	PRE-TERM	6	5	149.2	359.5	260.7	84.80	285.4	200.5	309.0
LNFP-V	G2	PRE-TERM	7	5	142.7	321.2	233.9	74.55	212.6	193.9	299.1
LNFP-V	G2	PRE-TERM	8	5	134.9	260.0	194.2	47.18	203.6	165.7	207.0
LNFP-V	G2	PRE-TERM	10	4	123.3	182.5	151.4	27.19	150.0	132.0	169.4
LNFP-V	G2	PRE-TERM	12	5	80.43	328.8	167.9	99.08	118.5	117.2	194.4
LNFP-V	G2	PRE-TERM	14	5	87.30	202.6	144.1	53.78	118.6	111.5	200.7
LNFP-V	G2	PRE-TERM	16	3	102.8	408.7	226.9	160.9	169.4	136.1	289.0
LNFP-V	G2	TERM	1	5	128.7	191.8	165.3	29.38	181.0	138.6	186.6
LNFP-V	G2	TERM	2	5	143.0	226.3	187.0	37.67	190.8	154.5	220.4

Table S2 Concentration of Human Milk Oligosaccharides in Term or Preterm Milk At Different Weeks Postpartum Separated By Milk Group

** When there are results below the method limit of quanfification (LoQ) the result has been assigned value of 0.5 × LoQ, hence the minimum value appears to be the same in many cases. When a large number of datapoints are below LoQ this can also have the effect that the median = minimum.*

					HMO Concentration (mg/L)						
HMO	Milk Group	Study Arm	Week Post Partum	N	min *	max	mean	sd	median *	Quartile 1 (25%)	Quartile 3 (75%)
LNFP-V	G2	TERM	3	5	139.7	247.0	190.8	45.88	209.6	147.2	210.6
LNFP-V	G2	TERM	4	5	163.2	202.6	180.3	18.50	173.2	165.2	197.5
LNFP-V	G2	TERM	5	5	139.2	228.2	168.3	37.26	154.3	140.1	179.4
LNFP-V	G2	TERM	6	5	46.89	226.4	136.8	64.33	142.8	119.8	148.0
LNFP-V	G2	TERM	7	5	87.21	162.9	118.7	33.13	111.1	89.82	142.2
LNFP-V	G2	TERM	8	5	76.47	196.7	127.1	44.19	124.8	107.4	130.3
LNFP-V	G3	PRE-TERM	1	1	12.00	12.00	12.00	NA	12.00	12.00	12.00
LNFP-V	G3	PRE-TERM	2	1	12.00	12.00	12.00	NA	12.00	12.00	12.00
LNFP-V	G3	PRE-TERM	3	1	24.37	24.37	24.37	NA	24.37	24.37	24.37
LNFP-V	G3	PRE-TERM	4	1	12.00	12.00	12.00	NA	12.00	12.00	12.00
LNFP-V	G3	PRE-TERM	5	1	12.00	12.00	12.00	NA	12.00	12.00	12.00
LNFP-V	G3	PRE-TERM	6	1	12.00	12.00	12.00	NA	12.00	12.00	12.00
LNFP-V	G3	PRE-TERM	7	1	12.00	12.00	12.00	NA	12.00	12.00	12.00
LNFP-V	G3	PRE-TERM	8	1	12.00	12.00	12.00	NA	12.00	12.00	12.00
LNFP-V	G3	PRE-TERM	10	1	12.00	12.00	12.00	NA	12.00	12.00	12.00
LNFP-V	G3	PRE-TERM	12	1	26.89	26.89	26.89	NA	26.89	26.89	26.89
LNFP-V	G3	PRE-TERM	14	1	25.61	25.61	25.61	NA	25.61	25.61	25.61
LNFP-V	G3	PRE-TERM	16	1	28.52	28.52	28.52	NA	28.52	28.52	28.52
LNFP-V	G3	TERM	1	1	25.88	25.88	25.88	NA	25.88	25.88	25.88
LNFP-V	G3	TERM	2	1	12.00	12.00	12.00	NA	12.00	12.00	12.00
LNFP-V	G3	TERM	3	1	12.00	12.00	12.00	NA	12.00	12.00	12.00
LNFP-V	G3	TERM	4	1	12.00	12.00	12.00	NA	12.00	12.00	12.00
LNFP-V	G3	TERM	5	1	12.00	12.00	12.00	NA	12.00	12.00	12.00
LNFP-V	G3	TERM	6	1	12.00	12.00	12.00	NA	12.00	12.00	12.00
LNFP-V	G3	TERM	7	1	12.00	12.00	12.00	NA	12.00	12.00	12.00
LNFP-V	G3	TERM	8	1	12.00	12.00	12.00	NA	12.00	12.00	12.00
LNFP-V	G4	TERM	1	1	203.7	203.7	203.7	NA	203.7	203.7	203.7
LNFP-V	G4	TERM	2	1	268.1	268.1	268.1	NA	268.1	268.1	268.1
LNFP-V	G4	TERM	3	1	127.3	127.3	127.3	NA	127.3	127.3	127.3
LNFP-V	G4	TERM	4	1	140.0	140.0	140.0	NA	140.0	140.0	140.0
LNFP-V	G4	TERM	5	1	103.6	103.6	103.6	NA	103.6	103.6	103.6
LNFP-V	G4	TERM	6	1	139.8	139.8	139.8	NA	139.8	139.8	139.8
LNFP-V	G4	TERM	7	1	102.4	102.4	102.4	NA	102.4	102.4	102.4
LNFP-V	G4	TERM	8	1	119.7	119.7	119.7	NA	119.7	119.7	119.7

Table S2 Concentration of Human Milk Oligosaccharides in Term or Preterm Milk At Different Weeks Postpartum Separated By Milk Group

** When there are results below the method limit of quanfification (LoQ) the result has been assigned value of 0.5 × LoQ, hence the minimum value appears to be the same in many cases. When a large number of datapoints are below LoQ this can also have the effect that the median = minimum.*

					HMO Concentration (mg/L)						
HMO	Milk Group	Study Arm	Week Post Partum	N	min *	max	mean	sd	median *	Quartile 1 (25%)	Quartile 3 (75%)
LNnDFH	G1	PRE-TERM	1	19	14.00	110.4	38.22	29.59	29.06	14.00	58.24
LNnDFH	G1	PRE-TERM	2	19	14.00	74.76	24.70	16.92	14.00	14.00	35.11
LNnDFH	G1	PRE-TERM	3	19	14.00	58.17	19.63	13.69	14.00	14.00	14.00
LNnDFH	G1	PRE-TERM	4	18	14.00	120.6	21.87	25.99	14.00	14.00	14.00
LNnDFH	G1	PRE-TERM	5	19	14.00	183.2	31.03	39.92	14.00	14.00	34.18
LNnDFH	G1	PRE-TERM	6	18	14.00	84.51	24.23	18.58	14.00	14.00	32.33
LNnDFH	G1	PRE-TERM	7	18	14.00	61.83	19.30	13.12	14.00	14.00	14.00
LNnDFH	G1	PRE-TERM	8	18	14.00	107.7	27.44	28.24	14.00	14.00	28.62
LNnDFH	G1	PRE-TERM	10	18	14.00	72.87	28.89	18.19	29.46	14.00	34.48
LNnDFH	G1	PRE-TERM	12	15	14.00	85.19	38.91	27.21	38.76	14.00	61.35
LNnDFH	G1	PRE-TERM	14	15	14.00	67.12	29.37	19.22	14.00	14.00	38.90
LNnDFH	G1	PRE-TERM	16	15	14.00	105.1	35.45	25.89	30.74	14.00	49.01
LNnDFH	G1	TERM	1	21	14.00	181.8	60.38	50.76	46.26	14.00	91.68
LNnDFH	G1	TERM	2	19	14.00	107.6	34.32	27.76	14.00	14.00	45.98
LNnDFH	G1	TERM	3	21	14.00	77.46	25.12	17.44	14.00	14.00	32.72
LNnDFH	G1	TERM	4	21	14.00	42.02	19.49	10.27	14.00	14.00	14.00
LNnDFH	G1	TERM	5	21	14.00	132.3	25.48	26.93	14.00	14.00	28.78
LNnDFH	G1	TERM	6	20	14.00	98.17	33.84	26.39	14.00	14.00	47.97
LNnDFH	G1	TERM	7	20	14.00	94.89	28.10	22.22	14.00	14.00	33.94
LNnDFH	G1	TERM	8	21	14.00	85.75	25.25	22.01	14.00	14.00	30.07
LNnDFH	G2	PRE-TERM	1	5	14.00	543.1	232.1	243.1	159.1	14.00	430.0
LNnDFH	G2	PRE-TERM	2	5	14.00	299.7	117.3	126.5	62.05	14.00	196.9
LNnDFH	G2	PRE-TERM	3	5	14.00	333.0	135.3	126.8	74.52	69.53	185.4
LNnDFH	G2	PRE-TERM	4	5	14.00	195.3	80.38	69.12	72.38	42.68	77.51
LNnDFH	G2	PRE-TERM	5	5	87.06	186.3	130.2	40.53	138.4	94.49	145.0
LNnDFH	G2	PRE-TERM	6	5	14.00	167.1	90.21	55.71	89.18	71.78	109.0
LNnDFH	G2	PRE-TERM	7	5	14.00	184.6	65.41	70.24	49.49	14.00	65.02
LNnDFH	G2	PRE-TERM	8	5	14.00	156.3	66.08	59.76	55.32	14.00	90.81
LNnDFH	G2	PRE-TERM	10	4	14.00	119.8	58.38	53.10	49.85	14.00	94.23
LNnDFH	G2	PRE-TERM	12	5	14.00	115.8	46.84	47.08	14.00	14.00	76.39
LNnDFH	G2	PRE-TERM	14	5	14.00	733.1	193.1	305.5	78.60	14.00	125.7
LNnDFH	G2	PRE-TERM	16	3	14.00	109.1	72.66	51.30	94.85	54.42	102.0
LNnDFH	G2	TERM	1	5	14.00	48.50	20.90	15.43	14.00	14.00	14.00
LNnDFH	G2	TERM	2	5	14.00	46.71	20.54	14.63	14.00	14.00	14.00

Table S2 Concentration of Human Milk Oligosaccharides in Term or Preterm Milk At Different Weeks Postpartum Separated By Milk Group

** When there are results below the method limit of quanfification (LoQ) the result has been assigned value of 0.5 × LoQ, hence the minimum value appears to be the same in many cases. When a large number of datapoints are below LoQ this can also have the effect that the median = minimum.*

					HMO Concentration (mg/L)						
HMO	Milk Group	Study Arm	Week Post Partum	N	min *	max	mean	sd	median *	Quartile 1 (25%)	Quartile 3 (75%)
LNnDFH	G2	TERM	3	5	14.00	40.38	19.28	11.80	14.00	14.00	14.00
LNnDFH	G2	TERM	4	5	14.00	34.25	18.05	9.055	14.00	14.00	14.00
LNnDFH	G2	TERM	5	5	14.00	31.98	17.60	8.039	14.00	14.00	14.00
LNnDFH	G2	TERM	6	5	14.00	97.13	30.63	37.18	14.00	14.00	14.00
LNnDFH	G2	TERM	7	5	14.00	77.39	26.68	28.35	14.00	14.00	14.00
LNnDFH	G2	TERM	8	5	14.00	94.07	30.01	35.81	14.00	14.00	14.00
LNnDFH	G3	PRE-TERM	1	1	14.00	14.00	14.00	NA	14.00	14.00	14.00
LNnDFH	G3	PRE-TERM	2	1	28.06	28.06	28.06	NA	28.06	28.06	28.06
LNnDFH	G3	PRE-TERM	3	1	14.00	14.00	14.00	NA	14.00	14.00	14.00
LNnDFH	G3	PRE-TERM	4	1	14.00	14.00	14.00	NA	14.00	14.00	14.00
LNnDFH	G3	PRE-TERM	5	1	14.00	14.00	14.00	NA	14.00	14.00	14.00
LNnDFH	G3	PRE-TERM	6	1	14.00	14.00	14.00	NA	14.00	14.00	14.00
LNnDFH	G3	PRE-TERM	7	1	14.00	14.00	14.00	NA	14.00	14.00	14.00
LNnDFH	G3	PRE-TERM	8	1	14.00	14.00	14.00	NA	14.00	14.00	14.00
LNnDFH	G3	PRE-TERM	10	1	14.00	14.00	14.00	NA	14.00	14.00	14.00
LNnDFH	G3	PRE-TERM	12	1	14.00	14.00	14.00	NA	14.00	14.00	14.00
LNnDFH	G3	PRE-TERM	14	1	14.00	14.00	14.00	NA	14.00	14.00	14.00
LNnDFH	G3	PRE-TERM	16	1	14.00	14.00	14.00	NA	14.00	14.00	14.00
LNnDFH	G3	TERM	1	1	14.00	14.00	14.00	NA	14.00	14.00	14.00
LNnDFH	G3	TERM	2	1	14.00	14.00	14.00	NA	14.00	14.00	14.00
LNnDFH	G3	TERM	3	1	28.07	28.07	28.07	NA	28.07	28.07	28.07
LNnDFH	G3	TERM	4	1	29.18	29.18	29.18	NA	29.18	29.18	29.18
LNnDFH	G3	TERM	5	1	14.00	14.00	14.00	NA	14.00	14.00	14.00
LNnDFH	G3	TERM	6	1	14.00	14.00	14.00	NA	14.00	14.00	14.00
LNnDFH	G3	TERM	7	1	14.00	14.00	14.00	NA	14.00	14.00	14.00
LNnDFH	G3	TERM	8	1	33.19	33.19	33.19	NA	33.19	33.19	33.19
LNnDFH	G4	TERM	1	1	60.72	60.72	60.72	NA	60.72	60.72	60.72
LNnDFH	G4	TERM	2	1	55.10	55.10	55.10	NA	55.10	55.10	55.10
LNnDFH	G4	TERM	3	1	14.00	14.00	14.00	NA	14.00	14.00	14.00
LNnDFH	G4	TERM	4	1	14.00	14.00	14.00	NA	14.00	14.00	14.00
LNnDFH	G4	TERM	5	1	14.00	14.00	14.00	NA	14.00	14.00	14.00
LNnDFH	G4	TERM	6	1	14.00	14.00	14.00	NA	14.00	14.00	14.00
LNnDFH	G4	TERM	7	1	14.00	14.00	14.00	NA	14.00	14.00	14.00
LNnDFH	G4	TERM	8	1	14.00	14.00	14.00	NA	14.00	14.00	14.00

Table S2 Concentration of Human Milk Oligosaccharides in Term or Preterm Milk At Different Weeks Postpartum Separated By Milk Group

** When there are results below the method limit of quanfification (LoQ) the result has been assigned value of 0.5 × LoQ, hence the minimum value appears to be the same in many cases. When a large number of datapoints are below LoQ this can also have the effect that the median = minimum.*

					HMO Concentration (mg/L)						
HMO	Milk Group	Study Arm	Week Post Partum	N	min *	max	mean	sd	median *	Quartile 1 (25%)	Quartile 3 (75%)
LNnFP	G1	PRE-TERM	1	19	9.500	89.76	17.38	19.33	9.500	9.500	14.70
LNnFP	G1	PRE-TERM	2	19	9.500	50.62	14.38	10.30	9.500	9.500	15.30
LNnFP	G1	PRE-TERM	3	19	9.500	50.34	13.13	10.07	9.500	9.500	9.500
LNnFP	G1	PRE-TERM	4	18	9.500	59.82	13.47	12.06	9.500	9.500	9.500
LNnFP	G1	PRE-TERM	5	19	9.500	61.67	14.38	12.58	9.500	9.500	9.500
LNnFP	G1	PRE-TERM	6	18	9.500	60.47	16.39	13.14	9.500	9.500	20.06
LNnFP	G1	PRE-TERM	7	18	9.500	55.24	14.91	12.16	9.500	9.500	9.500
LNnFP	G1	PRE-TERM	8	18	9.500	57.65	16.41	15.17	9.500	9.500	16.65
LNnFP	G1	PRE-TERM	10	18	9.500	60.19	17.53	14.96	9.500	9.500	18.69
LNnFP	G1	PRE-TERM	12	15	9.500	53.23	17.15	13.91	9.500	9.500	20.97
LNnFP	G1	PRE-TERM	14	15	9.500	70.04	16.99	18.20	9.500	9.500	9.500
LNnFP	G1	PRE-TERM	16	15	9.500	88.05	18.80	22.76	9.500	9.500	9.500
LNnFP	G1	TERM	1	21	9.500	38.23	12.07	7.120	9.500	9.500	9.500
LNnFP	G1	TERM	2	19	9.500	38.38	12.85	8.443	9.500	9.500	9.500
LNnFP	G1	TERM	3	21	9.500	27.38	10.90	4.542	9.500	9.500	9.500
LNnFP	G1	TERM	4	21	9.500	66.17	14.15	12.89	9.500	9.500	9.500
LNnFP	G1	TERM	5	21	9.500	29.19	12.21	6.051	9.500	9.500	9.500
LNnFP	G1	TERM	6	20	9.500	24.69	12.44	5.313	9.500	9.500	11.92
LNnFP	G1	TERM	7	20	9.500	22.33	12.68	5.024	9.500	9.500	19.18
LNnFP	G1	TERM	8	21	9.500	22.09	12.24	5.034	9.500	9.500	9.500
LNnFP	G2	PRE-TERM	1	5	9.500	35.34	25.36	9.621	28.16	25.09	28.70
LNnFP	G2	PRE-TERM	2	5	9.500	27.64	21.92	7.700	25.44	19.47	27.56
LNnFP	G2	PRE-TERM	3	5	9.500	40.83	22.95	11.60	19.24	19.18	26.02
LNnFP	G2	PRE-TERM	4	5	9.500	44.75	22.72	18.17	9.500	9.500	40.34
LNnFP	G2	PRE-TERM	5	5	9.500	45.15	19.60	15.66	9.500	9.500	24.35
LNnFP	G2	PRE-TERM	6	5	9.500	9.500	9.50	0.0000	9.500	9.500	9.500
LNnFP	G2	PRE-TERM	7	5	9.500	23.82	12.36	6.405	9.500	9.500	9.500
LNnFP	G2	PRE-TERM	8	5	9.500	22.87	12.17	5.978	9.500	9.500	9.500
LNnFP	G2	PRE-TERM	10	4	9.500	9.500	9.500	0.0000	9.500	9.500	9.500
LNnFP	G2	PRE-TERM	12	5	9.500	9.500	9.500	0.0000	9.500	9.500	9.500
LNnFP	G2	PRE-TERM	14	5	9.500	22.67	12.13	5.891	9.500	9.500	9.500
LNnFP	G2	PRE-TERM	16	3	9.500	9.500	9.500	0.0000	9.500	9.500	9.500
LNnFP	G2	TERM	1	5	21.93	56.56	41.13	15.45	49.07	27.31	50.79
LNnFP	G2	TERM	2	5	9.500	32.89	22.72	12.16	29.02	9.500	32.67

Table S2 Concentration of Human Milk Oligosaccharides in Term or Preterm Milk At Different Weeks Postpartum Separated By Milk Group

** When there are results below the method limit of quanfification (LoQ) the result has been assigned value of 0.5 × LoQ, hence the minimum value appears to be the same in many cases. When a large number of datapoints are below LoQ this can also have the effect that the median = minimum.*

HMO	Milk Group	Study Arm	Week Post Partum	N	HMO Concentration (mg/L) min *	max	mean	sd	median *	Quartile 1 (25%)	Quartile 3 (75%)
LNnFP	G2	TERM	3	5	9.500	23.56	18.34	5.240	19.53	19.37	19.72
LNnFP	G2	TERM	4	5	9.500	19.79	11.56	4.601	9.500	9.500	9.500
LNnFP	G2	TERM	5	5	9.500	20.88	11.78	5.091	9.500	9.500	9.500
LNnFP	G2	TERM	6	5	9.500	23.84	17.09	7.154	19.24	9.500	23.36
LNnFP	G2	TERM	7	5	9.500	23.61	14.74	7.212	9.500	9.500	21.60
LNnFP	G2	TERM	8	5	9.500	28.50	16.63	9.804	9.500	9.500	26.17
LNnFP	G3	PRE-TERM	1	1	9.500	9.500	9.500	NA	9.500	9.500	9.500
LNnFP	G3	PRE-TERM	2	1	9.500	9.500	9.500	NA	9.500	9.500	9.500
LNnFP	G3	PRE-TERM	3	1	9.500	9.500	9.500	NA	9.500	9.500	9.500
LNnFP	G3	PRE-TERM	4	1	9.500	9.500	9.500	NA	9.500	9.500	9.500
LNnFP	G3	PRE-TERM	5	1	9.500	9.500	9.500	NA	9.500	9.500	9.500
LNnFP	G3	PRE-TERM	6	1	9.500	9.500	9.500	NA	9.500	9.500	9.500
LNnFP	G3	PRE-TERM	7	1	9.500	9.500	9.500	NA	9.500	9.500	9.500
LNnFP	G3	PRE-TERM	8	1	9.500	9.500	9.500	NA	9.500	9.500	9.500
LNnFP	G3	PRE-TERM	10	1	9.500	9.500	9.500	NA	9.500	9.500	9.500
LNnFP	G3	PRE-TERM	12	1	9.500	9.500	9.500	NA	9.500	9.500	9.500
LNnFP	G3	PRE-TERM	14	1	9.500	9.500	9.500	NA	9.500	9.500	9.500
LNnFP	G3	PRE-TERM	16	1	9.500	9.500	9.500	NA	9.500	9.500	9.500
LNnFP	G3	TERM	1	1	9.500	9.500	9.500	NA	9.500	9.500	9.500
LNnFP	G3	TERM	2	1	9.500	9.500	9.500	NA	9.500	9.500	9.500
LNnFP	G3	TERM	3	1	9.500	9.500	9.500	NA	9.500	9.500	9.500
LNnFP	G3	TERM	4	1	9.500	9.500	9.500	NA	9.500	9.500	9.500
LNnFP	G3	TERM	5	1	9.500	9.500	9.500	NA	9.500	9.500	9.500
LNnFP	G3	TERM	6	1	9.500	9.500	9.500	NA	9.500	9.500	9.500
LNnFP	G3	TERM	7	1	9.500	9.500	9.500	NA	9.500	9.500	9.500
LNnFP	G3	TERM	8	1	9.500	9.500	9.500	NA	9.500	9.500	9.500
LNnFP	G4	TERM	1	1	9.500	9.500	9.500	NA	9.500	9.500	9.500
LNnFP	G4	TERM	2	1	9.500	9.500	9.500	NA	9.500	9.500	9.500
LNnFP	G4	TERM	3	1	49.35	49.35	49.35	NA	49.35	49.35	49.35
LNnFP	G4	TERM	4	1	40.04	40.04	40.04	NA	40.04	40.04	40.04
LNnFP	G4	TERM	5	1	52.21	52.21	52.21	NA	52.21	52.21	52.21
LNnFP	G4	TERM	6	1	56.13	56.13	56.13	NA	56.13	56.13	56.13
LNnFP	G4	TERM	7	1	37.85	37.85	37.85	NA	37.85	37.85	37.85
LNnFP	G4	TERM	8	1	23.06	23.06	23.06	NA	23.06	23.06	23.06

Table S2 Concentration of Human Milk Oligosaccharides in Term or Preterm Milk At Different Weeks Postpartum Separated By Milk Group

** When there are results below the method limit of quanfification (LoQ) the result has been assigned value of 0.5 × LoQ, hence the minimum value appears to be the same in many cases. When a large number of datapoints are below LoQ this can also have the effect that the median = minimum.*

					HMO Concentration (mg/L)						
HMO	Milk Group	Study Arm	Week Post Partum	N	min *	max	mean	sd	median *	Quartile 1 (25%)	Quartile 3 (75%)
LNnT	G1	PRE-TERM	1	19	145.7	546.3	309.5	115.0	317.3	208.6	408.9
LNnT	G1	PRE-TERM	2	19	115.9	380.7	249.4	85.34	254.2	182.4	310.4
LNnT	G1	PRE-TERM	3	19	84.09	353.8	211.2	74.32	207.3	165.7	258.7
LNnT	G1	PRE-TERM	4	18	80.52	349.0	203.3	80.83	202.0	156.9	244.5
LNnT	G1	PRE-TERM	5	19	74.63	383.2	184.3	89.05	183.1	108.9	236.6
LNnT	G1	PRE-TERM	6	18	12.00	397.8	196.4	99.75	205.5	138.2	252.3
LNnT	G1	PRE-TERM	7	18	69.59	314.7	193.1	74.36	191.2	160.5	243.0
LNnT	G1	PRE-TERM	8	18	77.62	440.8	189.0	86.08	184.5	156.2	203.4
LNnT	G1	PRE-TERM	10	18	61.39	464.0	183.4	102.3	164.2	114.1	217.4
LNnT	G1	PRE-TERM	12	15	51.86	379.4	152.2	84.47	140.1	97.18	164.9
LNnT	G1	PRE-TERM	14	15	38.26	444.3	156.6	102.0	129.1	86.51	180.1
LNnT	G1	PRE-TERM	16	15	34.77	437.3	148.8	105.4	119.5	80.88	194.5
LNnT	G1	TERM	1	21	190.1	565.3	348.6	93.32	349.8	287.9	402.6
LNnT	G1	TERM	2	19	129.4	475.4	256.3	93.62	252.6	201.2	272.8
LNnT	G1	TERM	3	21	78.28	385.9	202.1	82.98	198.7	140.4	230.4
LNnT	G1	TERM	4	21	74.62	350.3	187.5	74.72	180.4	139.2	249.7
LNnT	G1	TERM	5	21	79.18	369.5	176.3	80.25	146.4	116.8	225.1
LNnT	G1	TERM	6	20	71.86	311.2	164.4	70.87	143.2	115.1	215.2
LNnT	G1	TERM	7	20	52.58	270.0	157.1	61.32	147.5	126.4	198.7
LNnT	G1	TERM	8	21	64.27	284.9	145.1	66.27	131.4	88.27	186.8
LNnT	G2	PRE-TERM	1	5	90.25	257.5	151.3	66.04	137.3	106.1	165.4
LNnT	G2	PRE-TERM	2	5	96.36	190.4	141.8	36.87	150.1	115.1	156.9
LNnT	G2	PRE-TERM	3	5	66.51	192.5	128.2	48.31	136.5	97.50	147.7
LNnT	G2	PRE-TERM	4	5	63.43	179.1	102.4	49.03	79.69	66.71	123.0
LNnT	G2	PRE-TERM	5	5	43.09	112.2	74.18	30.57	67.88	48.51	99.24
LNnT	G2	PRE-TERM	6	5	48.61	118.6	80.14	27.25	68.95	68.78	95.82
LNnT	G2	PRE-TERM	7	5	35.89	112.8	73.13	31.10	60.11	59.95	96.86
LNnT	G2	PRE-TERM	8	5	33.45	103.7	64.04	32.24	54.40	36.42	92.24
LNnT	G2	PRE-TERM	10	4	24.44	77.16	45.58	24.73	40.36	26.66	59.28
LNnT	G2	PRE-TERM	12	5	12.00	66.88	42.47	20.12	47.80	37.04	48.63
LNnT	G2	PRE-TERM	14	5	12.00	70.50	39.54	20.88	38.00	35.29	41.91
LNnT	G2	PRE-TERM	16	3	25.55	39.37	32.43	6.911	32.38	28.97	35.88
LNnT	G2	TERM	1	5	187.1	338.7	244.0	57.85	229.1	214.0	251.3
LNnT	G2	TERM	2	5	113.9	136.8	126.7	9.685	126.2	121.2	135.6

Table S2 Concentration of Human Milk Oligosaccharides in Term or Preterm Milk At Different Weeks Postpartum Separated By Milk Group

** When there are results below the method limit of quanfification (LoQ) the result has been assigned value of 0.5 × LoQ, hence the minimum value appears to be the same in many cases. When a large number of datapoints are below LoQ this can also have the effect that the median = minimum.*

					HMO Concentration (mg/L)						
**HMO**	**Milk Group**	**Study Arm**	**Week Post Partum**	**N**	**min ***	**max**	**mean**	**sd**	**median ***	**Quartile 1 (25%)**	**Quartile 3 (75%)**
LNnT	G2	TERM	3	5	67.58	131.0	103.8	24.40	113.1	92.55	114.6
LNnT	G2	TERM	4	5	52.63	129.1	90.43	31.61	82.76	71.58	116.1
LNnT	G2	TERM	5	5	51.21	147.0	86.26	43.95	59.76	54.25	119.1
LNnT	G2	TERM	6	5	12.00	175.5	91.75	69.90	79.17	41.39	150.6
LNnT	G2	TERM	7	5	33.31	141.0	84.84	52.63	76.38	35.80	137.7
LNnT	G2	TERM	8	5	27.23	158.7	93.23	59.99	101.7	36.11	142.4
LNnT	G3	PRE-TERM	1	1	215.7	215.7	215.7	NA	215.7	215.7	215.7
LNnT	G3	PRE-TERM	2	1	118.3	118.3	118.3	NA	118.3	118.3	118.3
LNnT	G3	PRE-TERM	3	1	92.48	92.48	92.48	NA	92.48	92.48	92.48
LNnT	G3	PRE-TERM	4	1	108.9	108.9	108.9	NA	108.9	108.9	108.9
LNnT	G3	PRE-TERM	5	1	88.14	88.14	88.14	NA	88.14	88.14	88.14
LNnT	G3	PRE-TERM	6	1	118.9	118.9	118.9	NA	118.9	118.9	118.9
LNnT	G3	PRE-TERM	7	1	71.24	71.24	71.24	NA	71.24	71.24	71.24
LNnT	G3	PRE-TERM	8	1	93.06	93.06	93.06	NA	93.06	93.06	93.06
LNnT	G3	PRE-TERM	10	1	73.63	73.63	73.63	NA	73.63	73.63	73.63
LNnT	G3	PRE-TERM	12	1	74.39	74.39	74.39	NA	74.39	74.39	74.39
LNnT	G3	PRE-TERM	14	1	85.57	85.57	85.57	NA	85.57	85.57	85.57
LNnT	G3	PRE-TERM	16	1	67.99	67.99	67.99	NA	67.99	67.99	67.99
LNnT	G3	TERM	1	1	394.6	394.6	394.6	NA	394.6	394.6	394.6
LNnT	G3	TERM	2	1	162.7	162.7	162.7	NA	162.7	162.7	162.7
LNnT	G3	TERM	3	1	159.5	159.5	159.5	NA	159.5	159.5	159.5
LNnT	G3	TERM	4	1	135.3	135.3	135.3	NA	135.3	135.3	135.3
LNnT	G3	TERM	5	1	129.1	129.1	129.1	NA	129.1	129.1	129.1
LNnT	G3	TERM	6	1	77.12	77.12	77.12	NA	77.12	77.12	77.12
LNnT	G3	TERM	7	1	77.74	77.74	77.74	NA	77.74	77.74	77.74
LNnT	G3	TERM	8	1	101.1	101.1	101.1	NA	101.1	101.1	101.1
LNnT	G4	TERM	1	1	399.6	399.6	399.6	NA	399.6	399.6	399.6
LNnT	G4	TERM	2	1	247.5	247.5	247.5	NA	247.5	247.5	247.5
LNnT	G4	TERM	3	1	183.6	183.6	183.6	NA	183.6	183.6	183.6
LNnT	G4	TERM	4	1	118.1	118.1	118.1	NA	118.1	118.1	118.1
LNnT	G4	TERM	5	1	12.00	12.00	12.00	NA	12.00	12.00	12.00
LNnT	G4	TERM	6	1	76.25	76.25	76.25	NA	76.25	76.25	76.25
LNnT	G4	TERM	7	1	84.14	84.14	84.14	NA	84.14	84.14	84.14
LNnT	G4	TERM	8	1	104.1	104.1	104.1	NA	104.1	104.1	104.1

Table S2 Concentration of Human Milk Oligosaccharides in Term or Preterm Milk At Different Weeks Postpartum Separated By Milk Group

** When there are results below the method limit of quanfification (LoQ) the result has been assigned value of 0.5 × LoQ, hence the minimum value appears to be the same in many cases. When a large number of datapoints are below LoQ this can also have the effect that the median = minimum.*

					HMO Concentration (mg/L)						
HMO	Milk Group	Study Arm	Week Post Partum	N	min *	max	mean	sd	median *	Quartile 1 (25%)	Quartile 3 (75%)
LNT	G1	PRE-TERM	1	19	298.8	2002	936.7	454.9	819.4	675.1	1086
LNT	G1	PRE-TERM	2	19	644.9	2851	1306	544.9	1105	957.4	1650
LNT	G1	PRE-TERM	3	19	510.5	2277	1252	487.7	1167	931.7	1581
LNT	G1	PRE-TERM	4	18	469.1	2737	1114	553.5	1005	856.4	1320
LNT	G1	PRE-TERM	5	19	356.6	2468	1050	536.4	930.7	691.2	1222
LNT	G1	PRE-TERM	6	18	144.1	2428	971.2	591.2	887.5	538.8	1344
LNT	G1	PRE-TERM	7	18	312.8	2198	991.0	541.7	909.3	553.7	1437
LNT	G1	PRE-TERM	8	18	294.8	1960	912.6	478.9	839.9	506.7	1194
LNT	G1	PRE-TERM	10	18	223.6	1829	760.5	413.2	639.8	451.7	972.7
LNT	G1	PRE-TERM	12	15	175.3	1120	676.6	316.9	649.9	446.7	922.3
LNT	G1	PRE-TERM	14	15	232.8	1191	651.3	297.2	675.4	439.1	822.2
LNT	G1	PRE-TERM	16	15	145.2	1057	545.3	280.6	506.5	304.1	771.4
LNT	G1	TERM	1	21	199.8	1434	716.8	349.1	659.2	549.4	923.2
LNT	G1	TERM	2	19	637.9	1760	1156	302.5	1186	942.1	1347
LNT	G1	TERM	3	21	447.8	1924	1141	392.9	1233	854.1	1376
LNT	G1	TERM	4	21	384.6	1862	1075	382.2	1154	766.6	1282
LNT	G1	TERM	5	21	307.2	1894	918.3	436.8	766.7	632.9	1188
LNT	G1	TERM	6	20	280.7	1392	856.1	375.2	850.9	586.9	1255
LNT	G1	TERM	7	20	217.7	1549	818.0	384.7	794.2	482.2	1129
LNT	G1	TERM	8	21	213.7	1567	725.2	395.1	702.4	362.2	903.7
LNT	G2	PRE-TERM	1	5	1428	3029	2142	587.8	2061	1901	2290
LNT	G2	PRE-TERM	2	5	1613	3296	2457	657.7	2537	2029	2810
LNT	G2	PRE-TERM	3	5	1519	2903	2259	512.0	2283	2091	2498
LNT	G2	PRE-TERM	4	5	1854	2397	2084	278.8	1902	1887	2381
LNT	G2	PRE-TERM	5	5	1119	1867	1641	316.2	1825	1563	1831
LNT	G2	PRE-TERM	6	5	1628	2361	1910	284.0	1908	1712	1942
LNT	G2	PRE-TERM	7	5	1110	2376	1690	471.0	1762	1428	1775
LNT	G2	PRE-TERM	8	5	918.0	2184	1363	502.9	1333	992.2	1389
LNT	G2	PRE-TERM	10	4	704.7	1249	1018	262.8	1060	850.2	1228
LNT	G2	PRE-TERM	12	5	312.5	1554	1008	445.5	1051	1013	1109
LNT	G2	PRE-TERM	14	5	401.3	1162	739.3	319.5	775.7	447.8	909.4
LNT	G2	PRE-TERM	16	3	816.9	2113	1254	744.0	831.5	824.2	1472
LNT	G2	TERM	1	5	1130	2478	1801	584.6	1994	1253	2148
LNT	G2	TERM	2	5	1074	2056	1702	420.4	1844	1488	2050

Table S2 Concentration of Human Milk Oligosaccharides in Term or Preterm Milk At Different Weeks Postpartum Separated By Milk Group

** When there are results below the method limit of quanfification (LoQ) the result has been assigned value of 0.5 × LoQ, hence the minimum value appears to be the same in many cases. When a large number of datapoints are below LoQ this can also have the effect that the median = minimum.*

HMO	Milk Group	Study Arm	Week Post Partum	N	HMO Concentration (mg/L) min *	max	mean	sd	median *	Quartile 1 (25%)	Quartile 3 (75%)
LNT	G2	TERM	3	5	904.3	2236	1673	484.7	1766	1639	1819
LNT	G2	TERM	4	5	985.8	2354	1578	547.1	1346	1294	1909
LNT	G2	TERM	5	5	905.7	2342	1447	713.8	942.4	939.1	2104
LNT	G2	TERM	6	5	342.4	1986	1143	737.4	792.0	726.0	1869
LNT	G2	TERM	7	5	576.2	1405	931.7	358.0	813.3	659.9	1204
LNT	G2	TERM	8	5	454.4	1637	1024	518.1	764.7	756.8	1509
LNT	G3	PRE-TERM	1	1	975.2	975.2	975.2	NA	975.2	975.2	975.2
LNT	G3	PRE-TERM	2	1	965.1	965.1	965.1	NA	965.1	965.1	965.1
LNT	G3	PRE-TERM	3	1	1112	1112	1112	NA	1112	1112	1112
LNT	G3	PRE-TERM	4	1	1195	1195	1195	NA	1195	1195	1195
LNT	G3	PRE-TERM	5	1	880.4	880.4	880.4	NA	880.4	880.4	880.4
LNT	G3	PRE-TERM	6	1	760.5	760.5	760.5	NA	760.5	760.5	760.5
LNT	G3	PRE-TERM	7	1	592.7	592.7	592.7	NA	592.7	592.7	592.7
LNT	G3	PRE-TERM	8	1	659.1	659.1	659.1	NA	659.1	659.1	659.1
LNT	G3	PRE-TERM	10	1	561.7	561.7	561.7	NA	561.7	561.7	561.7
LNT	G3	PRE-TERM	12	1	660.0	660.0	660.0	NA	660.0	660.0	660.0
LNT	G3	PRE-TERM	14	1	586.0	586.0	586.0	NA	586.0	586.0	586.0
LNT	G3	PRE-TERM	16	1	604.4	604.4	604.4	NA	604.4	604.4	604.4
LNT	G3	TERM	1	1	359.0	359.0	359.0	NA	359.0	359.0	359.0
LNT	G3	TERM	2	1	358.7	358.7	358.7	NA	358.7	358.7	358.7
LNT	G3	TERM	3	1	588.9	588.9	588.9	NA	588.9	588.9	588.9
LNT	G3	TERM	4	1	484.2	484.2	484.2	NA	484.2	484.2	484.2
LNT	G3	TERM	5	1	572.1	572.1	572.1	NA	572.1	572.1	572.1
LNT	G3	TERM	6	1	394.1	394.1	394.1	NA	394.1	394.1	394.1
LNT	G3	TERM	7	1	417.8	417.8	417.8	NA	417.8	417.8	417.8
LNT	G3	TERM	8	1	307.1	307.1	307.1	NA	307.1	307.1	307.1
LNT	G4	TERM	1	1	3400	3400	3400	NA	3400	3400	3400
LNT	G4	TERM	2	1	3848	3848	3848	NA	3848	3848	3848
LNT	G4	TERM	3	1	3092	3092	3092	NA	3092	3092	3092
LNT	G4	TERM	4	1	3087	3087	3087	NA	3087	3087	3087
LNT	G4	TERM	5	1	2480	2480	2480	NA	2480	2480	2480
LNT	G4	TERM	6	1	2109	2109	2109	NA	2109	2109	2109
LNT	G4	TERM	7	1	2251	2251	2251	NA	2251	2251	2251
LNT	G4	TERM	8	1	1852	1852	1852	NA	1852	1852	1852

Table S2 Concentration of Human Milk Oligosaccharides in Term or Preterm Milk At Different Weeks Postpartum Separated By Milk Group

** When there are results below the method limit of quanfification (LoQ) the result has been assigned value of 0.5 × LoQ, hence the minimum value appears to be the same in many cases. When a large number of datapoints are below LoQ this can also have the effect that the median = minimum.*

					HMO Concentration (mg/L)						
HMO	Milk Group	Study Arm	Week Post Partum	N	min *	max	mean	sd	median *	Quartile 1 (25%)	Quartile 3 (75%)
LSTb	G1	PRE-TERM	1	19	44.25	147.8	81.29	28.49	74.27	60.42	90.22
LSTb	G1	PRE-TERM	2	19	41.49	211.3	97.72	42.38	93.18	72.78	109.6
LSTb	G1	PRE-TERM	3	19	45.44	205.4	105.4	44.97	105.5	71.08	129.6
LSTb	G1	PRE-TERM	4	18	47.34	164.8	99.56	39.42	101.0	64.86	130.3
LSTb	G1	PRE-TERM	5	19	42.09	240.8	104.3	50.36	101.0	53.33	133.3
LSTb	G1	PRE-TERM	6	18	37.86	161.8	97.13	40.93	106.8	55.17	131.7
LSTb	G1	PRE-TERM	7	18	36.96	144.9	91.64	37.36	100.7	56.31	124.0
LSTb	G1	PRE-TERM	8	18	7.000	191.9	88.63	46.85	87.16	51.68	119.6
LSTb	G1	PRE-TERM	10	18	34.87	195.0	86.45	43.84	76.68	48.87	115.1
LSTb	G1	PRE-TERM	12	15	31.54	152.7	80.35	39.36	85.94	44.70	101.0
LSTb	G1	PRE-TERM	14	15	25.18	113.3	75.75	25.70	78.78	64.49	90.99
LSTb	G1	PRE-TERM	16	15	23.33	102.6	69.22	22.79	75.40	52.93	87.97
LSTb	G1	TERM	1	21	33.70	141.7	72.05	27.95	68.78	50.04	89.73
LSTb	G1	TERM	2	19	28.89	149.1	71.39	27.42	64.19	53.50	83.47
LSTb	G1	TERM	3	21	36.75	163.7	82.00	31.97	81.00	56.56	106.0
LSTb	G1	TERM	4	21	20.15	136.8	80.38	32.02	77.58	57.77	106.5
LSTb	G1	TERM	5	21	21.68	166.6	75.44	34.27	69.84	54.18	95.80
LSTb	G1	TERM	6	20	7.000	147.4	70.38	36.83	71.33	43.33	94.00
LSTb	G1	TERM	7	20	17.27	177.0	80.42	41.44	72.37	54.23	107.0
LSTb	G1	TERM	8	21	7.000	152.6	66.20	37.54	59.12	43.19	92.50
LSTb	G2	PRE-TERM	1	5	125.0	219.5	173.9	36.04	183.1	153.2	188.4
LSTb	G2	PRE-TERM	2	5	117.2	182.1	151.0	23.49	151.4	145.4	159.2
LSTb	G2	PRE-TERM	3	5	110.3	217.4	158.2	38.68	151.3	148.0	163.7
LSTb	G2	PRE-TERM	4	5	92.87	256.1	160.4	68.06	156.2	100.9	195.8
LSTb	G2	PRE-TERM	5	5	106.0	334.7	165.4	95.89	133.0	110.4	142.8
LSTb	G2	PRE-TERM	6	5	96.85	314.7	168.0	85.29	139.1	127.5	161.7
LSTb	G2	PRE-TERM	7	5	91.92	423.9	173.0	142.8	100.0	92.79	156.5
LSTb	G2	PRE-TERM	8	5	74.62	400.7	159.9	136.9	109.8	78.90	135.2
LSTb	G2	PRE-TERM	10	4	66.06	143.0	92.98	34.29	81.43	74.37	100.0
LSTb	G2	PRE-TERM	12	5	54.05	405.6	147.2	148.6	80.04	55.66	140.5
LSTb	G2	PRE-TERM	14	5	51.40	243.3	114.5	75.43	85.64	77.72	114.5
LSTb	G2	PRE-TERM	16	3	43.51	247.3	132.6	104.3	106.9	75.22	177.1
LSTb	G2	TERM	1	5	65.94	196.6	117.3	48.75	102.3	99.12	122.7
LSTb	G2	TERM	2	5	51.20	108.2	82.02	24.09	73.74	72.16	104.9

Table S2 Concentration of Human Milk Oligosaccharides in Term or Preterm Milk At Different Weeks Postpartum Separated By Milk Group

** When there are results below the method limit of quanfification (LoQ) the result has been assigned value of 0.5 × LoQ, hence the minimum value appears to be the same in many cases. When a large number of datapoints are below LoQ this can also have the effect that the median = minimum.*

					HMO Concentration (mg/L)						
HMO	Milk Group	Study Arm	Week Post Partum	N	min *	max	mean	sd	median *	Quartile 1 (25%)	Quartile 3 (75%)
LSTb	G2	TERM	3	5	52.81	124.3	86.29	30.46	83.32	61.70	109.3
LSTb	G2	TERM	4	5	42.44	106.8	77.16	26.43	85.80	57.67	93.06
LSTb	G2	TERM	5	5	46.78	120.7	82.04	32.55	70.91	59.96	111.8
LSTb	G2	TERM	6	5	15.28	141.7	81.25	47.92	83.89	58.51	106.9
LSTb	G2	TERM	7	5	27.92	104.2	63.27	28.55	65.64	47.10	71.49
LSTb	G2	TERM	8	5	20.65	119.5	64.38	36.15	64.17	48.44	69.13
LSTb	G3	PRE-TERM	1	1	97.28	97.28	97.28	NA	97.28	97.28	97.28
LSTb	G3	PRE-TERM	2	1	161.6	161.6	161.6	NA	161.6	161.6	161.6
LSTb	G3	PRE-TERM	3	1	151.5	151.5	151.5	NA	151.5	151.5	151.5
LSTb	G3	PRE-TERM	4	1	107.7	107.7	107.7	NA	107.7	107.7	107.7
LSTb	G3	PRE-TERM	5	1	86.22	86.22	86.22	NA	86.22	86.22	86.22
LSTb	G3	PRE-TERM	6	1	67.69	67.69	67.69	NA	67.69	67.69	67.69
LSTb	G3	PRE-TERM	7	1	59.00	59.00	59.00	NA	59.00	59.00	59.00
LSTb	G3	PRE-TERM	8	1	54.27	54.27	54.27	NA	54.27	54.27	54.27
LSTb	G3	PRE-TERM	10	1	42.77	42.77	42.77	NA	42.77	42.77	42.77
LSTb	G3	PRE-TERM	12	1	52.23	52.23	52.23	NA	52.23	52.23	52.23
LSTb	G3	PRE-TERM	14	1	53.04	53.04	53.04	NA	53.04	53.04	53.04
LSTb	G3	PRE-TERM	16	1	53.45	53.45	53.45	NA	53.45	53.45	53.45
LSTb	G3	TERM	1	1	15.52	15.52	15.52	NA	15.52	15.52	15.52
LSTb	G3	TERM	2	1	40.88	40.88	40.88	NA	40.88	40.88	40.88
LSTb	G3	TERM	3	1	46.68	46.68	46.68	NA	46.68	46.68	46.68
LSTb	G3	TERM	4	1	54.30	54.30	54.30	NA	54.30	54.30	54.30
LSTb	G3	TERM	5	1	50.89	50.89	50.89	NA	50.89	50.89	50.89
LSTb	G3	TERM	6	1	46.48	46.48	46.48	NA	46.48	46.48	46.48
LSTb	G3	TERM	7	1	40.09	40.09	40.09	NA	40.09	40.09	40.09
LSTb	G3	TERM	8	1	45.28	45.28	45.28	NA	45.28	45.28	45.28
LSTb	G4	TERM	1	1	168.8	168.8	168.8	NA	168.8	168.8	168.8
LSTb	G4	TERM	2	1	160.5	160.5	160.5	NA	160.5	160.5	160.5
LSTb	G4	TERM	3	1	115.0	115.0	115.0	NA	115.0	115.0	115.0
LSTb	G4	TERM	4	1	124.3	124.3	124.3	NA	124.3	124.3	124.3
LSTb	G4	TERM	5	1	107.1	107.1	107.1	NA	107.1	107.1	107.1
LSTb	G4	TERM	6	1	136.3	136.3	136.3	NA	136.3	136.3	136.3
LSTb	G4	TERM	7	1	100.9	100.9	100.9	NA	100.9	100.9	100.9
LSTb	G4	TERM	8	1	97.38	97.38	97.38	NA	97.38	97.38	97.38

Table S2 Concentration of Human Milk Oligosaccharides in Term or Preterm Milk At Different Weeks Postpartum Separated By Milk Group

** When there are results below the method limit of quanfification (LoQ) the result has been assigned value of 0.5 × LoQ, hence the minimum value appears to be the same in many cases. When a large number of datapoints are below LoQ this can also have the effect that the median = minimum.*

					HMO Concentration (mg/L)						
HMO	Milk Group	Study Arm	Week Post Partum	N	min *	max	mean	sd	median *	Quartile 1 (25%)	Quartile 3 (75%)
LSTc	G1	PRE-TERM	1	19	95.01	1019	464.0	259.5	505.4	220.9	616.5
LSTc	G1	PRE-TERM	2	19	37.98	629.5	300.7	161.8	281.6	190.7	402.7
LSTc	G1	PRE-TERM	3	19	31.79	534.2	229.8	132.9	197.8	126.0	297.7
LSTc	G1	PRE-TERM	4	18	28.75	622.9	208.6	141.2	179.1	107.6	276.7
LSTc	G1	PRE-TERM	5	19	27.29	305.6	145.7	76.75	127.8	96.79	200.7
LSTc	G1	PRE-TERM	6	18	20.21	345.5	144.0	84.65	114.4	81.71	197.1
LSTc	G1	PRE-TERM	7	18	45.27	371.1	123.9	79.86	103.1	61.85	172.8
LSTc	G1	PRE-TERM	8	18	42.87	355.6	113.6	74.76	95.02	62.02	132.1
LSTc	G1	PRE-TERM	10	18	26.24	209.1	86.09	45.87	79.03	54.54	101.8
LSTc	G1	PRE-TERM	12	15	21.32	125.3	64.16	27.81	55.68	45.33	82.43
LSTc	G1	PRE-TERM	14	15	10.91	102.5	46.98	28.13	43.57	26.64	63.39
LSTc	G1	PRE-TERM	16	15	9.258	96.78	41.10	28.59	25.54	17.91	65.12
LSTc	G1	TERM	1	21	36.80	1199	566.7	274.0	526.0	404.9	740.6
LSTc	G1	TERM	2	19	215.4	1169	501.9	248.2	450.7	365.0	514.0
LSTc	G1	TERM	3	21	128.1	755.6	327.4	157.2	297.0	221.9	403.2
LSTc	G1	TERM	4	21	93.09	524.8	237.1	108.2	217.0	153.8	301.1
LSTc	G1	TERM	5	21	82.02	367.7	175.9	76.20	156.4	120.9	213.3
LSTc	G1	TERM	6	20	71.57	267.6	147.0	56.06	139.9	106.6	180.2
LSTc	G1	TERM	7	20	36.24	271.5	124.2	65.74	105.9	77.05	138.2
LSTc	G1	TERM	8	21	30.28	254.8	101.9	60.84	86.97	56.35	141.6
LSTc	G2	PRE-TERM	1	5	95.07	693.6	294.9	236.8	184.0	182.8	318.9
LSTc	G2	PRE-TERM	2	5	55.00	248.2	169.8	79.53	176.2	132.4	237.1
LSTc	G2	PRE-TERM	3	5	41.80	233.7	139.4	73.08	142.3	101.3	177.8
LSTc	G2	PRE-TERM	4	5	30.60	129.5	87.41	36.64	97.02	78.40	101.6
LSTc	G2	PRE-TERM	5	5	24.49	119.0	74.08	35.73	76.65	57.53	92.70
LSTc	G2	PRE-TERM	6	5	23.78	110.0	72.49	32.75	78.48	59.87	90.38
LSTc	G2	PRE-TERM	7	5	17.24	90.27	52.65	28.07	51.20	36.77	67.76
LSTc	G2	PRE-TERM	8	5	17.82	75.30	42.03	24.48	36.31	22.11	58.60
LSTc	G2	PRE-TERM	10	4	28.57	51.70	41.82	10.16	43.50	36.84	48.48
LSTc	G2	PRE-TERM	12	5	28.41	43.54	34.29	5.755	33.53	30.95	35.01
LSTc	G2	PRE-TERM	14	5	11.60	35.59	23.48	9.823	25.88	15.48	28.83
LSTc	G2	PRE-TERM	16	3	18.59	22.43	21.03	2.117	22.05	20.32	22.24
LSTc	G2	TERM	1	5	302.5	761.3	490.8	181.2	517.1	347.0	526.1
LSTc	G2	TERM	2	5	204.1	398.1	303.3	74.22	316.4	260.3	337.8

Table S2 Concentration of Human Milk Oligosaccharides in Term or Preterm Milk At Different Weeks Postpartum Separated By Milk Group

** When there are results below the method limit of quanfification (LoQ) the result has been assigned value of 0.5 × LoQ, hence the minimum value appears to be the same in many cases. When a large number of datapoints are below LoQ this can also have the effect that the median = minimum.*

HMO	Milk Group	Study Arm	Week Post Partum	N	HMO Concentration (mg/L) min *	max	mean	sd	median *	Quartile 1 (25%)	Quartile 3 (75%)
LSTc	G2	TERM	3	5	103.3	232.6	176.0	52.82	165.2	156.2	222.6
LSTc	G2	TERM	4	5	64.34	151.8	114.6	32.76	114.3	109.1	133.4
LSTc	G2	TERM	5	5	32.73	129.5	86.37	37.93	86.73	69.49	113.4
LSTc	G2	TERM	6	5	30.19	135.6	77.95	44.31	91.63	36.34	95.98
LSTc	G2	TERM	7	5	20.07	91.63	52.31	26.42	52.74	39.15	57.98
LSTc	G2	TERM	8	5	16.92	86.93	47.80	25.66	43.60	37.96	53.62
LSTc	G3	PRE-TERM	1	1	526.1	526.1	526.1	NA	526.1	526.1	526.1
LSTc	G3	PRE-TERM	2	1	303.7	303.7	303.7	NA	303.7	303.7	303.7
LSTc	G3	PRE-TERM	3	1	136.2	136.2	136.2	NA	136.2	136.2	136.2
LSTc	G3	PRE-TERM	4	1	87.33	87.33	87.33	NA	87.33	87.33	87.33
LSTc	G3	PRE-TERM	5	1	62.73	62.73	62.73	NA	62.73	62.73	62.73
LSTc	G3	PRE-TERM	6	1	71.54	71.54	71.54	NA	71.54	71.54	71.54
LSTc	G3	PRE-TERM	7	1	42.20	42.20	42.20	NA	42.20	42.20	42.20
LSTc	G3	PRE-TERM	8	1	38.50	38.50	38.50	NA	38.50	38.50	38.50
LSTc	G3	PRE-TERM	10	1	24.55	24.55	24.55	NA	24.55	24.55	24.55
LSTc	G3	PRE-TERM	12	1	20.28	20.28	20.28	NA	20.28	20.28	20.28
LSTc	G3	PRE-TERM	14	1	24.47	24.47	24.47	NA	24.47	24.47	24.47
LSTc	G3	PRE-TERM	16	1	17.33	17.33	17.33	NA	17.33	17.33	17.33
LSTc	G3	TERM	1	1	853.8	853.8	853.8	NA	853.8	853.8	853.8
LSTc	G3	TERM	2	1	298.2	298.2	298.2	NA	298.2	298.2	298.2
LSTc	G3	TERM	3	1	193.2	193.2	193.2	NA	193.2	193.2	193.2
LSTc	G3	TERM	4	1	139.1	139.1	139.1	NA	139.1	139.1	139.1
LSTc	G3	TERM	5	1	97.32	97.32	97.32	NA	97.32	97.32	97.32
LSTc	G3	TERM	6	1	73.72	73.72	73.72	NA	73.72	73.72	73.72
LSTc	G3	TERM	7	1	62.70	62.70	62.70	NA	62.70	62.70	62.70
LSTc	G3	TERM	8	1	76.90	76.90	76.90	NA	76.90	76.90	76.90
LSTc	G4	TERM	1	1	979.8	979.8	979.8	NA	979.8	979.8	979.8
LSTc	G4	TERM	2	1	1156	1156	1156	NA	1156	1156	1156
LSTc	G4	TERM	3	1	620.3	620.3	620.3	NA	620.3	620.3	620.3
LSTc	G4	TERM	4	1	346.2	346.2	346.2	NA	346.2	346.2	346.2
LSTc	G4	TERM	5	1	188.7	188.7	188.7	NA	188.7	188.7	188.7
LSTc	G4	TERM	6	1	160.6	160.6	160.6	NA	160.6	160.6	160.6
LSTc	G4	TERM	7	1	96.53	96.53	96.53	NA	96.53	96.53	96.53
LSTc	G4	TERM	8	1	57.77	57.77	57.77	NA	57.77	57.77	57.77

Table S2 Concentration of Human Milk Oligosaccharides in Term or Preterm Milk At Different Weeks Postpartum Separated By Milk Group

** When there are results below the method limit of quanfification (LoQ) the result has been assigned value of 0.5 × LoQ, hence the minimum value appears to be the same in many cases. When a large number of datapoints are below LoQ this can also have the effect that the median = minimum.*

					HMO Concentration (mg/L)						
HMO	Milk Group	Study Arm	Week Post Partum	N	min *	max	mean	sd	median *	Quartile 1 (25%)	Quartile 3 (75%)
MFLNH-III	G1	PRE-TERM	1	19	70.38	548.6	248.6	139.4	221.4	141.2	317.6
MFLNH-III	G1	PRE-TERM	2	19	58.75	649.8	357.7	153.4	336.7	260.5	458.2
MFLNH-III	G1	PRE-TERM	3	19	64.26	663.0	347.8	149.2	355.2	262.4	415.4
MFLNH-III	G1	PRE-TERM	4	18	65.81	604.0	313.6	143.3	331.7	228.6	392.0
MFLNH-III	G1	PRE-TERM	5	19	57.17	445.5	248.8	108.8	253.2	185.9	317.8
MFLNH-III	G1	PRE-TERM	6	18	87.89	422.1	230.9	91.09	221.8	187.1	275.3
MFLNH-III	G1	PRE-TERM	7	18	79.18	400.7	221.3	92.75	206.7	157.7	285.2
MFLNH-III	G1	PRE-TERM	8	18	64.97	420.5	210.2	106.1	192.8	134.1	269.7
MFLNH-III	G1	PRE-TERM	10	18	53.68	321.8	146.2	80.43	135.8	85.01	162.6
MFLNH-III	G1	PRE-TERM	12	15	17.50	323.4	115.2	74.22	109.8	66.07	141.9
MFLNH-III	G1	PRE-TERM	14	15	17.50	215.4	101.4	51.18	96.61	70.10	116.2
MFLNH-III	G1	PRE-TERM	16	15	17.50	193.4	80.91	51.04	79.62	48.33	88.04
MFLNH-III	G1	TERM	1	21	17.50	469.8	186.9	118.6	152.2	121.3	211.7
MFLNH-III	G1	TERM	2	19	180.0	613.8	358.8	115.7	337.0	292.2	419.0
MFLNH-III	G1	TERM	3	21	166.7	578.2	356.5	108.0	340.7	302.1	399.4
MFLNH-III	G1	TERM	4	21	121.1	505.6	335.8	101.5	332.1	289.1	408.7
MFLNH-III	G1	TERM	5	21	81.47	532.2	282.5	112.9	295.6	202.5	362.1
MFLNH-III	G1	TERM	6	20	17.50	425.6	257.5	91.58	279.3	218.5	291.9
MFLNH-III	G1	TERM	7	20	17.50	417.6	209.8	87.48	200.0	150.6	266.6
MFLNH-III	G1	TERM	8	21	17.50	443.9	192.5	99.88	173.3	139.7	233.4
MFLNH-III	G2	PRE-TERM	1	5	166.0	327.0	237.4	76.50	194.1	186.2	313.5
MFLNH-III	G2	PRE-TERM	2	5	147.0	714.1	441.3	208.0	437.6	377.6	530.3
MFLNH-III	G2	PRE-TERM	3	5	165.4	790.5	510.6	258.6	517.0	355.5	724.8
MFLNH-III	G2	PRE-TERM	4	5	141.6	743.2	487.0	229.5	454.7	454.4	640.9
MFLNH-III	G2	PRE-TERM	5	5	101.4	820.2	411.6	258.5	385.5	354.1	397.0
MFLNH-III	G2	PRE-TERM	6	5	116.0	856.2	456.5	272.1	438.0	337.9	534.3
MFLNH-III	G2	PRE-TERM	7	5	77.34	745.1	419.2	247.3	476.4	304.9	492.1
MFLNH-III	G2	PRE-TERM	8	5	66.95	753.0	355.4	253.9	286.7	261.1	409.3
MFLNH-III	G2	PRE-TERM	10	4	221.7	707.1	363.4	230.3	262.4	239.6	386.2
MFLNH-III	G2	PRE-TERM	12	5	58.13	551.6	224.3	204.0	144.9	78.50	288.3
MFLNH-III	G2	PRE-TERM	14	5	50.63	386.3	173.5	167.4	53.20	53.16	324.5
MFLNH-III	G2	PRE-TERM	16	3	123.9	337.9	231.2	107.0	231.9	177.9	284.9
MFLNH-III	G2	TERM	1	5	17.50	385.1	200.1	139.4	169.0	148.4	280.6
MFLNH-III	G2	TERM	2	5	326.4	772.4	610.4	174.6	685.0	569.1	699.1

Table S2 Concentration of Human Milk Oligosaccharides in Term or Preterm Milk At Different Weeks Postpartum Separated By Milk Group

** When there are results below the method limit of quanfification (LoQ) the result has been assigned value of 0.5 × LoQ, hence the minimum value appears to be the same in many cases. When a large number of datapoints are below LoQ this can also have the effect that the median = minimum.*

					HMO Concentration (mg/L)						
HMO	Milk Group	Study Arm	Week Post Partum	N	min *	max	mean	sd	median *	Quartile 1 (25%)	Quartile 3 (75%)
MFLNH-III	G2	TERM	3	5	312.8	861.7	678.7	212.3	751.9	706.1	761.1
MFLNH-III	G2	TERM	4	5	339.0	976.4	734.2	239.2	806.8	722.0	826.8
MFLNH-III	G2	TERM	5	5	222.9	886.4	598.6	238.2	620.9	617.0	645.6
MFLNH-III	G2	TERM	6	5	194.8	758.8	416.2	217.5	393.8	273.3	460.5
MFLNH-III	G2	TERM	7	5	17.50	600.2	320.7	236.4	409.7	140.6	435.4
MFLNH-III	G2	TERM	8	5	163.9	875.6	428.6	267.2	382.4	314.1	407.1
MFLNH-III	G3	PRE-TERM	1	1	452.0	452.0	452.0	NA	452.0	452.0	452.0
MFLNH-III	G3	PRE-TERM	2	1	330.7	330.7	330.7	NA	330.7	330.7	330.7
MFLNH-III	G3	PRE-TERM	3	1	314.2	314.2	314.2	NA	314.2	314.2	314.2
MFLNH-III	G3	PRE-TERM	4	1	467.1	467.1	467.1	NA	467.1	467.1	467.1
MFLNH-III	G3	PRE-TERM	5	1	454.7	454.7	454.7	NA	454.7	454.7	454.7
MFLNH-III	G3	PRE-TERM	6	1	443.4	443.4	443.4	NA	443.4	443.4	443.4
MFLNH-III	G3	PRE-TERM	7	1	370.4	370.4	370.4	NA	370.4	370.4	370.4
MFLNH-III	G3	PRE-TERM	8	1	334.9	334.9	334.9	NA	334.9	334.9	334.9
MFLNH-III	G3	PRE-TERM	10	1	246.6	246.6	246.6	NA	246.6	246.6	246.6
MFLNH-III	G3	PRE-TERM	12	1	180.9	180.9	180.9	NA	180.9	180.9	180.9
MFLNH-III	G3	PRE-TERM	14	1	140.0	140.0	140.0	NA	140.0	140.0	140.0
MFLNH-III	G3	PRE-TERM	16	1	102.0	102.0	102.0	NA	102.0	102.0	102.0
MFLNH-III	G3	TERM	1	1	524.0	524.0	524.0	NA	524.0	524.0	524.0
MFLNH-III	G3	TERM	2	1	493.2	493.2	493.2	NA	493.2	493.2	493.2
MFLNH-III	G3	TERM	3	1	593.9	593.9	593.9	NA	593.9	593.9	593.9
MFLNH-III	G3	TERM	4	1	496.9	496.9	496.9	NA	496.9	496.9	496.9
MFLNH-III	G3	TERM	5	1	574.6	574.6	574.6	NA	574.6	574.6	574.6
MFLNH-III	G3	TERM	6	1	355.1	355.1	355.1	NA	355.1	355.1	355.1
MFLNH-III	G3	TERM	7	1	369.9	369.9	369.9	NA	369.9	369.9	369.9
MFLNH-III	G3	TERM	8	1	299.2	299.2	299.2	NA	299.2	299.2	299.2
MFLNH-III	G4	TERM	1	1	189.9	189.9	189.9	NA	189.9	189.9	189.9
MFLNH-III	G4	TERM	2	1	695.9	695.9	695.9	NA	695.9	695.9	695.9
MFLNH-III	G4	TERM	3	1	934.4	934.4	934.4	NA	934.4	934.4	934.4
MFLNH-III	G4	TERM	4	1	895.2	895.2	895.2	NA	895.2	895.2	895.2
MFLNH-III	G4	TERM	5	1	872.4	872.4	872.4	NA	872.4	872.4	872.4
MFLNH-III	G4	TERM	6	1	513.6	513.6	513.6	NA	513.6	513.6	513.6
MFLNH-III	G4	TERM	7	1	571.8	571.8	571.8	NA	571.8	571.8	571.8
MFLNH-III	G4	TERM	8	1	427.0	427.0	427.0	NA	427.0	427.0	427.0
