# Supplementary material for: Human Milk Oligosaccharides in the Milk of Mothers Delivering Term versus Preterm Infants
Source: Nutrients. 2019 Jun 5;11(6):1282. doi: 10.3390/nu11061282 (PMC6627155; doi:10.3390/nu11061282)
Supplement: Supplementary file 1 [file nutrients-11-01282-s001.zip › nutrients-512673-supplementary/Supp_Table_S3_HMO_PM_All.pdf]

Table S3 Concentration of Human Milk Oligosaccharides in Term or Preterm Milk At Specified Postmenstrual Age

** When there are results below the method limit of quanfification (LoQ) the result has been assigned value of 0.5 × LoQ, hence the minimum value appears to be the same in many cases. When a large number of datapoints are below LoQ this can also have the effect that the median = minimum.*

				HMO Concentration (mg/L)						
HMO	Study Arm	Postmenstrual Age (weeks)	N	min *	max	mean	sd	median *	Quartile 1 (25%)	Quartile 3 (75%)
2'FL	PRE-TERM	29	1	35.03	35.03	35.03	NA	35.03	35.03	35.03
2'FL	PRE-TERM	30	10	21.88	3958	2347	1643	3327	634.6	3462
2'FL	PRE-TERM	31	12	6.500	3798	1860	1284	2213	935.7	2438
2'FL	PRE-TERM	32	17	6.500	4196	1933	1375	1975	1070	2900
2'FL	PRE-TERM	33	24	6.500	5478	1890	1407	2194	819.7	2660
2'FL	PRE-TERM	34	25	6.500	3592	1627	1125	1705	832.5	2721
2'FL	PRE-TERM	35	24	6.500	3071	1515	1036	1643	626.4	2310
2'FL	PRE-TERM	36	25	6.500	3088	1573	1040	1819	788.8	2373
2'FL	PRE-TERM	37	23	6.500	3335	1585	1008	1735	1051	2264
2'FL	PRE-TERM	38	15	6.500	3006	1569	1190	1958	135.4	2650
2'FL	PRE-TERM	39	20	6.500	3151	1585	1012	1540	1195	2466
2'FL	PRE-TERM	40	10	6.500	3353	1466	1221	1576	252.0	2454
2'FL	PRE-TERM	41	15	6.500	3574	1713	1101	1895	1233	2293
2'FL	PRE-TERM	42	9	6.500	2926	1231	1129	1427	16.58	1552
2'FL	PRE-TERM	43	13	6.500	2847	1421	940.5	1464	1079	2077
2'FL	PRE-TERM	44	8	6.500	2687	1036	1035	961.5	13.04	1677
2'FL	PRE-TERM	45	12	6.500	2198	1208	862.5	1195	689.0	2042
2'FL	PRE-TERM	46	8	6.500	2946	1381	1120	1350	538.9	2158
2'FL	PRE-TERM	47	3	956.7	2203	1753	691.7	2099	1528	2151
2'FL	PRE-TERM	48	5	6.500	3455	1424	1303	1461	625.6	1569
2'FL	TERM	38	2	3186	4230	3708	738.3	3708	3447	3969
2'FL	TERM	39	10	6.500	5570	3463	1766	3171	2560	4969
2'FL	TERM	40	19	6.500	4305	2033	1530	2302	26.25	3260
2'FL	TERM	41	27	6.500	6076	2494	1601	2469	2027	3055
2'FL	TERM	42	29	6.500	5572	2043	1412	2043	1387	3065
2'FL	TERM	43	28	6.500	4390	2006	1311	2079	1509	2841
2'FL	TERM	44	27	6.500	4556	1845	1264	2043	1092	2935
2'FL	TERM	45	28	6.500	4090	1830	1281	1770	967.5	2994
2'FL	TERM	46	24	6.500	3666	1616	1200	1720	182.9	2581
2'FL	TERM	47	18	6.500	3530	1654	1269	1693	183.6	2885

Table S3 Concentration of Human Milk Oligosaccharides in Term or Preterm Milk At Specified Postmenstrual Age

** When there are results below the method limit of quanfification (LoQ) the result has been assigned value of 0.5 × LoQ, hence the minimum value appears to be the same in many cases. When a large number of datapoints are below LoQ this can also have the effect that the median = minimum.*

				HMO Concentration (mg/L)						
HMO	Study Arm	Postmenstrual Age (weeks)	N	min *	max	mean	sd	median *	Quartile 1 (25%)	Quartile 3 (75%)
2'FL	TERM	48	7	1399	4133	2782	854.0	2868	2428	3110
3'GL	PRE-TERM	29	1	11.32	11.32	11.32	NA	11.32	11.32	11.32
3'GL	PRE-TERM	30	10	4.000	10.44	5.944	2.587	4.000	4.000	8.174
3'GL	PRE-TERM	31	12	4.000	14.88	5.782	3.519	4.000	4.000	5.154
3'GL	PRE-TERM	32	17	4.000	28.16	7.583	6.324	4.000	4.000	10.38
3'GL	PRE-TERM	33	24	4.000	28.82	8.182	7.592	4.000	4.000	9.552
3'GL	PRE-TERM	34	25	4.000	30.06	7.369	6.804	4.000	4.000	9.744
3'GL	PRE-TERM	35	24	4.000	21.56	5.706	4.371	4.000	4.000	4.000
3'GL	PRE-TERM	36	25	4.000	10.04	4.775	1.846	4.000	4.000	4.000
3'GL	PRE-TERM	37	23	4.000	16.72	5.539	3.318	4.000	4.000	4.000
3'GL	PRE-TERM	38	15	4.000	70.00	11.52	17.05	4.000	4.000	10.99
3'GL	PRE-TERM	39	20	4.000	23.04	6.149	5.050	4.000	4.000	4.000
3'GL	PRE-TERM	40	10	4.000	21.24	8.792	6.732	4.000	4.000	12.78
3'GL	PRE-TERM	41	15	4.000	15.76	6.140	4.453	4.000	4.000	4.000
3'GL	PRE-TERM	42	9	4.000	21.49	8.540	6.193	4.000	4.000	11.78
3'GL	PRE-TERM	43	13	4.000	15.01	5.395	3.497	4.000	4.000	4.000
3'GL	PRE-TERM	44	8	4.000	10.64	5.441	2.708	4.000	4.000	5.222
3'GL	PRE-TERM	45	12	4.000	10.27	4.874	2.087	4.000	4.000	4.000
3'GL	PRE-TERM	46	8	4.000	15.21	7.858	4.511	6.523	4.000	10.63
3'GL	PRE-TERM	47	3	4.000	4.000	4.000	0.0000	4.000	4.000	4.000
3'GL	PRE-TERM	48	5	4.000	12.84	5.769	3.955	4.000	4.000	4.000
3'GL	TERM	38	2	8.823	9.726	9.274	0.6384	9.274	9.049	9.500
3'GL	TERM	39	10	4.000	19.87	10.76	5.880	9.735	5.105	15.77
3'GL	TERM	40	19	4.000	32.97	12.18	7.800	12.76	4.000	15.42
3'GL	TERM	41	27	4.000	19.72	8.324	4.870	8.173	4.000	11.70
3'GL	TERM	42	29	4.000	17.67	5.552	3.208	4.000	4.000	4.000
3'GL	TERM	43	28	4.000	16.81	5.967	3.465	4.000	4.000	8.365
3'GL	TERM	44	27	4.000	18.94	5.874	4.258	4.000	4.000	4.000
3'GL	TERM	45	28	4.000	33.42	5.596	5.872	4.000	4.000	4.000
3'GL	TERM	46	24	4.000	10.07	4.463	1.576	4.000	4.000	4.000

Table S3 Concentration of Human Milk Oligosaccharides in Term or Preterm Milk At Specified Postmenstrual Age

** When there are results below the method limit of quanfification (LoQ) the result has been assigned value of 0.5 × LoQ, hence the minimum value appears to be the same in many cases. When a large number of datapoints are below LoQ this can also have the effect that the median = minimum.*

HMO	Study Arm	Postmenstrual Age (weeks)	N	HMO Concentration (mg/L) min *	max	mean	sd	median *	Quartile 1 (25%)	Quartile 3 (75%)
3'GL	TERM	47	18	4.000	16.11	4.924	2.988	4.000	4.000	4.000
3'GL	TERM	48	7	4.000	20.73	6.391	6.325	4.000	4.000	4.000
3'SL	PRE-TERM	29	1	419.1	419.1	419.1	NA	419.1	419.1	419.1
3'SL	PRE-TERM	30	10	174.7	365.6	230.5	55.48	221.0	196.3	247.8
3'SL	PRE-TERM	31	12	126.6	329.8	215.7	48.28	215.2	193.8	238.5
3'SL	PRE-TERM	32	17	107.7	272.6	203.5	48.64	209.5	184.5	241.6
3'SL	PRE-TERM	33	24	113.1	513.3	204.6	85.71	192.4	149.1	237.8
3'SL	PRE-TERM	34	25	107.6	439.9	199.1	77.81	173.1	151.4	234.3
3'SL	PRE-TERM	35	24	98.75	430.3	191.0	76.44	173.8	157.4	207.2
3'SL	PRE-TERM	36	25	103.5	362.9	176.6	61.49	168.9	129.5	199.7
3'SL	PRE-TERM	37	23	85.96	342.8	178.3	62.12	166.0	143.1	208.9
3'SL	PRE-TERM	38	15	91.34	438.1	185.1	92.53	169.3	108.7	229.4
3'SL	PRE-TERM	39	20	91.08	329.8	168.4	64.07	159.4	121.3	192.1
3'SL	PRE-TERM	40	10	92.61	333.8	174.9	81.31	147.5	119.8	208.4
3'SL	PRE-TERM	41	15	99.07	393.0	209.4	92.31	173.0	147.3	258.6
3'SL	PRE-TERM	42	9	83.76	274.9	148.7	65.02	146.8	93.94	162.9
3'SL	PRE-TERM	43	13	109.4	403.2	201.6	88.32	163.7	132.8	250.0
3'SL	PRE-TERM	44	8	86.41	248.9	147.8	57.48	131.8	108.3	164.8
3'SL	PRE-TERM	45	12	86.95	325.0	177.4	66.44	163.1	128.2	212.2
3'SL	PRE-TERM	46	8	82.71	282.3	162.9	71.35	153.3	114.6	185.1
3'SL	PRE-TERM	47	3	116.2	264.4	184.9	74.69	174.1	145.1	219.3
3'SL	PRE-TERM	48	5	100.5	319.4	163.5	88.68	130.3	121.3	145.9
3'SL	TERM	38	2	181.9	351.2	266.5	119.7	266.5	224.2	308.9
3'SL	TERM	39	10	119.6	287.2	213.4	65.63	220.1	150.4	276.3
3'SL	TERM	40	19	94.16	380.6	187.4	84.03	153.1	130.4	235.4
3'SL	TERM	41	27	78.49	270.7	149.2	47.01	139.2	119.7	173.0
3'SL	TERM	42	29	86.80	280.3	135.6	38.89	134.0	105.6	153.2
3'SL	TERM	43	28	77.94	282.3	132.9	40.79	126.9	105.2	154.2
3'SL	TERM	44	27	72.18	272.4	128.6	42.45	126.0	94.14	150.2
3'SL	TERM	45	28	35.33	274.9	128.9	44.43	125.5	105.5	152.0

Table S3 Concentration of Human Milk Oligosaccharides in Term or Preterm Milk At Specified Postmenstrual Age

** When there are results below the method limit of quanfification (LoQ) the result has been assigned value of 0.5 × LoQ, hence the minimum value appears to be the same in many cases. When a large number of datapoints are below LoQ this can also have the effect that the median = minimum.*

				HMO Concentration (mg/L)						
HMO	Study Arm	Postmenstrual Age (weeks)	N	min *	max	mean	sd	median *	Quartile 1 (25%)	Quartile 3 (75%)
3'SL	TERM	46	24	74.78	303.0	125.4	45.56	112.4	102.0	136.7
3'SL	TERM	47	18	50.25	240.1	125.0	45.82	127.1	89.94	150.9
3'SL	TERM	48	7	94.50	236.8	144.4	47.99	140.2	113.8	155.7
3FL	PRE-TERM	29	1	1472	1472	1472	NA	1472	1472	1472
3FL	PRE-TERM	30	10	64.12	1176	474.3	388.5	300.0	199.2	748.7
3FL	PRE-TERM	31	12	148.3	1124	468.8	366.5	292.5	206.9	655.0
3FL	PRE-TERM	32	17	112.2	1137	467.4	352.8	315.1	186.2	575.6
3FL	PRE-TERM	33	24	154.5	1457	523.7	386.3	374.3	264.6	673.2
3FL	PRE-TERM	34	25	179.6	1549	578.9	400.2	409.6	308.8	770.1
3FL	PRE-TERM	35	24	205.2	1488	637.2	376.5	494.0	443.2	755.3
3FL	PRE-TERM	36	25	228.4	1787	669.2	450.5	508.2	391.5	730.7
3FL	PRE-TERM	37	23	224.9	2465	798.1	601.9	601.0	433.8	905.7
3FL	PRE-TERM	38	15	75.94	1661	788.9	398.6	752.4	591.5	974.2
3FL	PRE-TERM	39	20	241.8	1825	747.9	396.5	694.8	525.3	841.1
3FL	PRE-TERM	40	10	487.8	2231	874.2	506.3	727.7	586.6	919.6
3FL	PRE-TERM	41	15	278.6	2126	917.6	546.1	777.4	611.7	947.7
3FL	PRE-TERM	42	9	492.5	2821	1057	705.9	899.8	638.4	1158
3FL	PRE-TERM	43	13	298.6	3101	1128	911.1	790.3	579.4	1045
3FL	PRE-TERM	44	8	546.0	2151	1111	497.1	1129	753.3	1223
3FL	PRE-TERM	45	12	325.1	2188	1021	621.9	853.8	606.4	1195
3FL	PRE-TERM	46	8	486.6	3398	1391	942.2	1185	831.9	1457
3FL	PRE-TERM	47	3	745.8	1094	927.1	174.7	941.1	843.5	1018
3FL	PRE-TERM	48	5	688.4	2505	1343	695.0	1133	1013	1378
3FL	TERM	38	2	260.1	341.2	300.7	57.39	300.7	280.4	321.0
3FL	TERM	39	10	137.2	1142	351.0	303.2	205.9	175.0	429.0
3FL	TERM	40	19	145.3	1245	484.2	366.3	302.7	207.4	715.6
3FL	TERM	41	27	39.33	1577	420.6	386.7	273.5	182.7	484.5
3FL	TERM	42	29	66.65	1861	524.3	433.9	350.3	228.3	608.7
3FL	TERM	43	28	82.63	1675	577.1	420.8	417.0	309.5	726.8
3FL	TERM	44	27	99.24	1848	628.7	459.1	456.7	376.0	736.4

Table S3 Concentration of Human Milk Oligosaccharides in Term or Preterm Milk At Specified Postmenstrual Age

** When there are results below the method limit of quanfification (LoQ) the result has been assigned value of 0.5 × LoQ, hence the minimum value appears to be the same in many cases. When a large number of datapoints are below LoQ this can also have the effect that the median = minimum.*

				HMO Concentration (mg/L)						
HMO	Study Arm	Postmenstrual Age (weeks)	N	min *	max	mean	sd	median *	Quartile 1 (25%)	Quartile 3 (75%)
3FL	TERM	45	28	95.34	1983	664.0	434.1	531.8	429.7	685.8
3FL	TERM	46	24	102.9	1978	742.7	506.4	551.3	445.3	975.5
3FL	TERM	47	18	118.2	2013	712.5	500.8	545.8	448.1	913.2
3FL	TERM	48	7	131.6	721.9	465.8	203.4	468.0	368.5	601.1
6'GL	PRE-TERM	29	1	54.12	54.12	54.12	NA	54.12	54.12	54.12
6'GL	PRE-TERM	30	10	29.26	156.4	83.51	42.55	87.28	44.35	103.7
6'GL	PRE-TERM	31	12	17.55	109.6	53.08	27.16	47.65	36.18	74.65
6'GL	PRE-TERM	32	17	16.66	106.1	52.56	29.84	49.64	24.67	71.55
6'GL	PRE-TERM	33	24	18.91	137.9	50.01	29.30	42.29	28.06	61.93
6'GL	PRE-TERM	34	25	16.16	75.81	34.65	13.63	30.35	26.46	41.32
6'GL	PRE-TERM	35	24	3.150	55.98	28.62	11.66	26.15	23.45	33.86
6'GL	PRE-TERM	36	25	11.33	48.53	24.94	8.857	23.40	18.30	30.48
6'GL	PRE-TERM	37	23	7.746	43.71	24.69	9.233	23.56	19.17	30.21
6'GL	PRE-TERM	38	15	12.88	179.6	35.97	41.48	23.88	17.82	30.21
6'GL	PRE-TERM	39	20	9.818	33.74	17.97	6.454	16.45	13.99	20.02
6'GL	PRE-TERM	40	10	12.86	48.40	23.05	11.29	18.91	17.84	22.28
6'GL	PRE-TERM	41	15	10.40	35.95	20.74	8.054	17.89	14.30	25.90
6'GL	PRE-TERM	42	9	9.255	44.84	21.53	10.54	18.77	15.52	24.56
6'GL	PRE-TERM	43	13	8.465	32.86	19.07	6.895	16.48	14.18	22.66
6'GL	PRE-TERM	44	8	6.997	52.92	20.83	14.35	16.52	13.80	22.68
6'GL	PRE-TERM	45	12	8.310	28.68	16.06	6.662	12.31	11.15	20.65
6'GL	PRE-TERM	46	8	8.740	46.05	20.91	12.68	15.69	12.93	24.95
6'GL	PRE-TERM	47	3	11.73	21.16	15.14	5.229	12.52	12.12	16.84
6'GL	PRE-TERM	48	5	3.150	15.94	11.94	5.153	14.07	11.72	14.83
6'GL	TERM	38	2	108.2	128.5	118.3	14.31	118.3	113.3	123.4
6'GL	TERM	39	10	38.11	232.6	107.0	64.81	106.8	55.43	119.5
6'GL	TERM	40	19	12.64	234.0	102.2	60.96	94.87	59.89	135.5
6'GL	TERM	41	27	8.541	170.4	63.30	40.29	53.13	34.08	82.75
6'GL	TERM	42	29	7.969	69.86	37.13	15.17	36.24	28.52	50.18
6'GL	TERM	43	28	3.150	59.96	27.45	13.89	28.80	18.18	36.82

Table S3 Concentration of Human Milk Oligosaccharides in Term or Preterm Milk At Specified Postmenstrual Age

** When there are results below the method limit of quanfification (LoQ) the result has been assigned value of 0.5 × LoQ, hence the minimum value appears to be the same in many cases. When a large number of datapoints are below LoQ this can also have the effect that the median = minimum.*

			HMO Concentration (mg/L)							
HMO	Study Arm	Postmenstrual Age (weeks)	N	min *	max	mean	sd	median *	Quartile 1 (25%)	Quartile 3 (75%)
6'GL	TERM	44	27	3.150	47.44	23.52	11.20	23.93	16.51	30.27
6'GL	TERM	45	28	3.150	68.27	20.69	13.32	18.92	12.87	26.07
6'GL	TERM	46	24	3.150	40.84	18.80	8.489	16.33	15.11	22.56
6'GL	TERM	47	18	8.807	47.47	18.28	8.932	14.95	14.05	20.64
6'GL	TERM	48	7	9.963	40.02	17.74	10.67	12.89	12.12	18.55
6'SL	PRE-TERM	29	1	303.3	303.3	303.3	NA	303.3	303.3	303.3
6'SL	PRE-TERM	30	10	145.3	673.9	430.9	219.6	494.9	222.2	635.9
6'SL	PRE-TERM	31	12	63.98	879.6	509.3	273.5	590.5	289.3	667.4
6'SL	PRE-TERM	32	17	61.39	1132	509.2	285.8	598.5	263.9	659.3
6'SL	PRE-TERM	33	24	53.89	1135	471.9	263.5	477.3	327.3	576.6
6'SL	PRE-TERM	34	25	74.89	926.1	407.2	223.0	387.5	241.1	541.4
6'SL	PRE-TERM	35	24	74.47	695.8	370.6	164.5	401.3	257.2	491.4
6'SL	PRE-TERM	36	25	55.02	565.1	323.6	149.2	315.4	167.5	449.2
6'SL	PRE-TERM	37	23	57.52	476.6	276.0	113.7	294.3	183.9	365.6
6'SL	PRE-TERM	38	15	10.00	394.0	252.2	97.01	249.2	204.8	309.4
6'SL	PRE-TERM	39	20	91.24	383.8	199.2	78.82	184.4	147.9	231.5
6'SL	PRE-TERM	40	10	135.1	401.4	218.2	95.04	180.6	148.7	279.0
6'SL	PRE-TERM	41	15	72.75	199.9	147.1	37.92	154.2	129.1	171.7
6'SL	PRE-TERM	42	9	100.6	271.0	172.2	73.21	158.9	105.5	256.2
6'SL	PRE-TERM	43	13	60.09	185.6	112.0	38.36	101.1	88.74	129.4
6'SL	PRE-TERM	44	8	65.69	212.2	146.5	50.68	156.0	122.5	173.3
6'SL	PRE-TERM	45	12	46.43	176.7	92.85	40.21	84.59	59.48	113.7
6'SL	PRE-TERM	46	8	61.11	196.6	121.2	50.68	115.8	78.43	154.3
6'SL	PRE-TERM	47	3	79.24	109.6	95.55	15.29	97.87	88.55	103.7
6'SL	PRE-TERM	48	5	48.01	142.2	88.10	42.89	65.21	58.93	126.1
6'SL	TERM	38	2	313.5	442.1	377.8	90.97	377.8	345.7	410.0
6'SL	TERM	39	10	222.0	706.2	428.5	130.1	402.1	364.9	494.0
6'SL	TERM	40	19	295.7	1084	556.0	213.8	508.6	387.9	662.5
6'SL	TERM	41	27	216.1	985.4	594.9	210.8	552.4	439.9	780.7
6'SL	TERM	42	29	198.9	821.3	555.2	179.9	572.4	463.3	684.6

Table S3 Concentration of Human Milk Oligosaccharides in Term or Preterm Milk At Specified Postmenstrual Age

** When there are results below the method limit of quanfification (LoQ) the result has been assigned value of 0.5 × LoQ, hence the minimum value appears to be the same in many cases. When a large number of datapoints are below LoQ this can also have the effect that the median = minimum.*

				HMO Concentration (mg/L)						
HMO	Study Arm	Postmenstrual Age (weeks)	N	min *	max	mean	sd	median *	Quartile 1 (25%)	Quartile 3 (75%)
6'SL	TERM	43	28	129.4	792.3	469.6	176.4	486.7	341.0	573.2
6'SL	TERM	44	27	24.60	574.6	353.5	149.3	387.3	255.9	435.7
6'SL	TERM	45	28	89.66	700.9	302.8	142.6	321.2	185.4	360.9
6'SL	TERM	46	24	53.06	662.3	263.5	130.4	241.1	199.3	322.4
6'SL	TERM	47	18	99.59	671.2	252.5	135.0	209.9	170.5	285.1
6'SL	TERM	48	7	145.8	301.0	204.9	52.97	192.3	176.3	221.2
4-Tetrasaccharide	PRE-TERM	29	1	7.500	7.500	7.500	NA	7.500	7.500	7.500
4-Tetrasaccharide	PRE-TERM	30	10	7.500	166.6	26.86	50.27	7.500	7.500	7.500
4-Tetrasaccharide	PRE-TERM	31	12	7.500	70.30	19.02	22.95	7.500	7.500	12.61
4-Tetrasaccharide	PRE-TERM	32	17	7.500	315.1	47.39	83.02	7.500	7.500	54.83
4-Tetrasaccharide	PRE-TERM	33	24	7.500	232.5	49.50	70.29	7.500	7.500	65.43
4-Tetrasaccharide	PRE-TERM	34	25	7.500	250.0	33.91	61.74	7.500	7.500	19.00
4-Tetrasaccharide	PRE-TERM	35	24	7.500	224.7	42.79	67.38	7.500	7.500	25.74
4-Tetrasaccharide	PRE-TERM	36	25	7.500	226.0	41.18	66.59	7.500	7.500	27.62
4-Tetrasaccharide	PRE-TERM	37	23	7.500	232.6	40.60	63.02	7.500	7.500	38.05
4-Tetrasaccharide	PRE-TERM	38	15	7.500	266.6	60.21	89.13	7.500	7.500	70.09
4-Tetrasaccharide	PRE-TERM	39	20	7.500	322.7	48.35	89.70	7.500	7.500	23.05
4-Tetrasaccharide	PRE-TERM	40	10	7.500	113.0	29.44	42.36	7.500	7.500	19.50
4-Tetrasaccharide	PRE-TERM	41	15	7.500	334.5	49.07	94.25	7.500	7.500	15.20
4-Tetrasaccharide	PRE-TERM	42	9	7.500	101.9	29.85	40.38	7.500	7.500	22.64
4-Tetrasaccharide	PRE-TERM	43	13	7.500	300.7	49.96	91.55	7.500	7.500	7.500
4-Tetrasaccharide	PRE-TERM	44	8	7.500	86.79	19.30	27.78	7.500	7.500	11.28
4-Tetrasaccharide	PRE-TERM	45	12	7.500	329.8	52.36	97.22	7.500	7.500	30.59
4-Tetrasaccharide	PRE-TERM	46	8	7.500	132.3	37.58	50.15	7.500	7.500	47.51
4-Tetrasaccharide	PRE-TERM	47	3	7.500	144.8	77.28	68.69	79.52	43.51	112.2
4-Tetrasaccharide	PRE-TERM	48	5	7.500	102.4	32.23	41.17	7.500	7.500	36.19
4-Tetrasaccharide	TERM	38	2	7.500	7.500	7.500	0.0000	7.500	7.500	7.500
4-Tetrasaccharide	TERM	39	10	7.500	188.2	36.21	57.81	7.500	7.500	46.38
4-Tetrasaccharide	TERM	40	19	7.500	154.4	30.39	40.33	7.500	7.500	40.12
4-Tetrasaccharide	TERM	41	27	7.500	150.2	26.46	34.19	7.500	7.500	37.45

Table S3 Concentration of Human Milk Oligosaccharides in Term or Preterm Milk At Specified Postmenstrual Age

** When there are results below the method limit of quanfification (LoQ) the result has been assigned value of 0.5 × LoQ, hence the minimum value appears to be the same in many cases. When a large number of datapoints are below LoQ this can also have the effect that the median = minimum.*

				HMO Concentration (mg/L)						
**HMO**	**Study Arm**	**Postmenstrual Age (weeks)**	**N**	**min ***	**max**	**mean**	**sd**	**median ***	**Quartile 1 (25%)**	**Quartile 3 (75%)**
A-Tetrasaccharide	TERM	42	29	7.500	149.0	24.92	34.39	7.500	7.500	33.43
A-Tetrasaccharide	TERM	43	28	7.500	153.9	26.56	34.47	7.500	7.500	33.52
A-Tetrasaccharide	TERM	44	27	7.500	162.3	27.03	36.66	7.500	7.500	28.20
A-Tetrasaccharide	TERM	45	28	7.500	138.9	23.50	33.48	7.500	7.500	25.12
A-Tetrasaccharide	TERM	46	24	7.500	183.8	29.69	43.75	7.500	7.500	28.82
A-Tetrasaccharide	TERM	47	18	7.500	165.4	29.38	45.03	7.500	7.500	22.23
A-Tetrasaccharide	TERM	48	7	7.500	181.0	50.04	67.95	7.500	7.500	69.63
DFLNHa	PRE-TERM	29	1	16.50	16.50	16.50	NA	16.50	16.50	16.50
DFLNHa	PRE-TERM	30	10	16.50	577.3	159.4	172.7	121.5	24.24	217.8
DFLNHa	PRE-TERM	31	12	16.50	495.2	212.9	185.3	184.5	16.50	389.3
DFLNHa	PRE-TERM	32	17	16.50	430.4	189.5	148.6	184.4	33.32	334.0
DFLNHa	PRE-TERM	33	24	16.50	583.6	184.5	153.7	180.9	16.50	297.9
DFLNHa	PRE-TERM	34	25	16.50	623.8	145.4	137.1	122.4	52.33	210.8
DFLNHa	PRE-TERM	35	24	16.50	542.4	125.1	113.1	141.3	16.50	165.8
DFLNHa	PRE-TERM	36	25	16.50	418.7	137.2	105.2	138.9	45.30	192.4
DFLNHa	PRE-TERM	37	23	16.50	351.9	111.5	86.69	101.2	40.79	152.7
DFLNHa	PRE-TERM	38	15	16.50	685.1	113.8	165.8	71.73	16.50	125.1
DFLNHa	PRE-TERM	39	20	16.50	249.7	71.43	59.80	63.68	16.50	97.26
DFLNHa	PRE-TERM	40	10	16.50	230.0	77.00	72.97	61.51	16.50	108.2
DFLNHa	PRE-TERM	41	15	16.50	179.2	47.95	42.05	36.86	16.50	60.39
DFLNHa	PRE-TERM	42	9	16.50	103.6	48.14	39.62	16.50	16.50	85.30
DFLNHa	PRE-TERM	43	13	16.50	83.70	43.28	21.41	47.27	16.50	51.95
DFLNHa	PRE-TERM	44	8	16.50	77.74	36.72	24.24	27.57	16.50	52.73
DFLNHa	PRE-TERM	45	12	16.50	139.6	37.86	36.24	16.50	16.50	45.64
DFLNHa	PRE-TERM	46	8	16.50	60.97	37.92	19.34	40.06	16.50	54.97
DFLNHa	PRE-TERM	47	3	16.50	52.20	35.51	17.96	37.84	27.17	45.02
DFLNHa	PRE-TERM	48	5	16.50	51.52	29.43	17.81	16.50	16.50	46.15
DFLNHa	TERM	38	2	130.3	159.2	144.7	20.46	144.7	137.5	152.0
DFLNHa	TERM	39	10	16.50	306.3	127.1	104.4	79.40	62.19	198.0
DFLNHa	TERM	40	19	16.50	399.0	130.9	108.2	131.6	16.50	178.7

Table S3 Concentration of Human Milk Oligosaccharides in Term or Preterm Milk At Specified Postmenstrual Age

** When there are results below the method limit of quanfification (LoQ) the result has been assigned value of 0.5 × LoQ, hence the minimum value appears to be the same in many cases. When a large number of datapoints are below LoQ this can also have the effect that the median = minimum.*

				HMO Concentration (mg/L)						
HMO	Study Arm	Postmenstrual Age (weeks)	N	min *	max	mean	sd	median *	Quartile 1 (25%)	Quartile 3 (75%)
DFLNHa	TERM	41	27	16.50	488.4	194.6	125.3	181.4	103.9	259.1
DFLNHa	TERM	42	29	16.50	664.0	215.1	177.2	196.2	79.80	300.9
DFLNHa	TERM	43	28	16.50	759.1	199.7	180.7	162.2	67.58	322.2
DFLNHa	TERM	44	27	16.50	709.4	170.6	169.5	125.2	35.25	269.3
DFLNHa	TERM	45	28	16.50	747.0	145.7	160.8	114.5	16.50	227.8
DFLNHa	TERM	46	24	16.50	563.0	114.4	126.3	77.45	16.50	163.5
DFLNHa	TERM	47	18	16.50	525.5	123.0	133.2	101.2	16.50	137.8
DFLNHa	TERM	48	7	81.61	463.1	182.7	137.8	121.4	101.3	205.0
DSLNT	PRE-TERM	29	1	946.4	946.4	946.4	NA	946.4	946.4	946.4
DSLNT	PRE-TERM	30	10	17.50	843.2	355.5	208.3	333.9	263.9	392.5
DSLNT	PRE-TERM	31	12	17.50	786.4	398.7	209.6	419.4	386.4	488.7
DSLNT	PRE-TERM	32	17	193.6	719.0	412.4	142.1	430.8	295.4	511.9
DSLNT	PRE-TERM	33	24	197.2	843.4	443.6	179.7	389.3	303.4	588.7
DSLNT	PRE-TERM	34	25	169.1	822.5	427.0	184.2	398.1	258.9	559.2
DSLNT	PRE-TERM	35	24	128.4	908.0	419.8	216.2	364.8	265.3	536.9
DSLNT	PRE-TERM	36	25	129.9	796.0	345.5	182.8	291.5	213.2	426.4
DSLNT	PRE-TERM	37	23	105.5	725.2	305.0	155.8	282.1	182.9	381.7
DSLNT	PRE-TERM	38	15	100.2	628.2	286.8	150.4	289.8	175.4	374.0
DSLNT	PRE-TERM	39	20	97.12	597.5	263.9	146.3	214.3	174.3	316.9
DSLNT	PRE-TERM	40	10	105.8	530.6	314.5	141.2	292.2	227.9	423.5
DSLNT	PRE-TERM	41	15	84.32	452.7	218.9	104.1	191.0	164.9	255.5
DSLNT	PRE-TERM	42	9	72.56	422.5	238.5	111.5	256.2	158.8	328.2
DSLNT	PRE-TERM	43	13	97.17	435.1	208.0	92.41	169.3	148.8	241.7
DSLNT	PRE-TERM	44	8	102.0	318.0	203.0	78.25	216.4	132.4	249.2
DSLNT	PRE-TERM	45	12	97.08	459.9	190.6	96.20	165.0	136.4	212.0
DSLNT	PRE-TERM	46	8	88.23	311.8	204.4	76.04	209.0	162.4	254.4
DSLNT	PRE-TERM	47	3	103.0	158.2	133.9	28.20	140.5	121.8	149.4
DSLNT	PRE-TERM	48	5	124.7	219.3	183.0	37.51	184.3	174.5	212.0
DSLNT	TERM	38	2	412.7	433.7	423.2	14.79	423.2	418.0	428.4
DSLNT	TERM	39	10	248.3	489.2	359.2	79.12	353.3	308.5	401.1

Table S3 Concentration of Human Milk Oligosaccharides in Term or Preterm Milk At Specified Postmenstrual Age

** When there are results below the method limit of quanfification (LoQ) the result has been assigned value of 0.5 × LoQ, hence the minimum value appears to be the same in many cases. When a large number of datapoints are below LoQ this can also have the effect that the median = minimum.*

				HMO Concentration (mg/L)						
HMO	Study Arm	Postmenstrual Age (weeks)	N	min *	max	mean	sd	median *	Quartile 1 (25%)	Quartile 3 (75%)
DSLNT	TERM	40	19	146.3	697.9	389.5	149.4	348.3	277.4	475.5
DSLNT	TERM	41	27	135.3	880.1	367.0	145.9	357.8	279.9	403.8
DSLNT	TERM	42	29	17.50	836.5	332.2	152.6	309.1	249.9	406.8
DSLNT	TERM	43	28	105.1	630.2	293.9	116.1	286.9	222.1	336.6
DSLNT	TERM	44	27	90.80	555.7	247.5	105.2	225.4	166.7	317.5
DSLNT	TERM	45	28	48.35	426.1	221.4	95.81	196.5	159.7	295.5
DSLNT	TERM	46	24	69.19	437.5	194.0	90.51	172.4	125.9	247.8
DSLNT	TERM	47	18	48.04	422.7	185.0	93.01	177.2	118.8	250.6
DSLNT	TERM	48	7	60.14	223.1	148.2	63.00	140.2	103.6	203.2
LDFT	PRE-TERM	29	1	22.50	22.50	22.50	NA	22.50	22.50	22.50
LDFT	PRE-TERM	30	10	22.50	546.1	258.0	230.9	200.6	31.04	506.4
LDFT	PRE-TERM	31	12	22.50	759.0	234.4	230.8	159.1	58.08	307.6
LDFT	PRE-TERM	32	17	22.50	1243	259.7	307.2	148.2	89.90	310.7
LDFT	PRE-TERM	33	24	22.50	864.6	236.6	242.9	168.2	52.94	253.9
LDFT	PRE-TERM	34	25	22.50	2513	299.3	518.2	131.8	90.33	262.6
LDFT	PRE-TERM	35	24	22.50	1926	284.1	388.4	204.4	76.99	302.9
LDFT	PRE-TERM	36	25	22.50	604.5	182.6	148.8	163.8	70.57	248.0
LDFT	PRE-TERM	37	23	22.50	910.9	231.3	204.2	178.0	74.68	331.7
LDFT	PRE-TERM	38	15	22.50	2333	453.2	589.8	292.4	99.55	469.1
LDFT	PRE-TERM	39	20	22.50	813.5	266.7	243.3	203.6	102.8	339.9
LDFT	PRE-TERM	40	10	22.50	2758	466.0	825.2	206.8	47.81	384.0
LDFT	PRE-TERM	41	15	22.50	3003	725.4	901.8	430.0	115.2	837.2
LDFT	PRE-TERM	42	9	22.50	677.0	210.4	207.9	210.5	22.50	231.9
LDFT	PRE-TERM	43	13	22.50	1827	464.0	618.7	251.7	91.69	394.0
LDFT	PRE-TERM	44	8	22.50	574.3	216.6	208.7	169.1	22.50	371.3
LDFT	PRE-TERM	45	12	22.50	1825	430.6	552.1	279.6	83.40	407.5
LDFT	PRE-TERM	46	8	22.50	791.9	288.6	243.8	266.8	179.3	321.4
LDFT	PRE-TERM	47	3	142.6	499.2	327.3	178.6	340.0	241.3	419.6
LDFT	PRE-TERM	48	5	22.50	516.9	284.5	204.4	284.9	150.8	447.7
LDFT	TERM	38	2	390.4	617.8	504.1	160.8	504.1	447.3	561.0

Table S3 Concentration of Human Milk Oligosaccharides in Term or Preterm Milk At Specified Postmenstrual Age

** When there are results below the method limit of quanfification (LoQ) the result has been assigned value of 0.5 × LoQ, hence the minimum value appears to be the same in many cases. When a large number of datapoints are below LoQ this can also have the effect that the median = minimum.*

				HMO Concentration (mg/L)						
HMO	Study Arm	Postmenstrual Age (weeks)	N	min *	max	mean	sd	median *	Quartile 1 (25%)	Quartile 3 (75%)
LDFT	TERM	39	10	22.50	983.9	491.3	325.2	473.2	271.8	743.3
LDFT	TERM	40	19	22.50	810.2	299.3	263.6	325.0	22.50	456.9
LDFT	TERM	41	27	22.50	965.0	247.5	196.9	235.2	100.4	335.1
LDFT	TERM	42	29	22.50	549.3	198.1	133.4	222.2	109.1	281.0
LDFT	TERM	43	28	22.50	1270	250.4	272.1	193.3	133.0	296.4
LDFT	TERM	44	27	22.50	2907	286.4	536.7	196.4	112.6	297.8
LDFT	TERM	45	28	22.50	1713	278.5	349.8	222.5	110.0	301.5
LDFT	TERM	46	24	22.50	566.9	219.9	159.6	216.7	89.24	336.0
LDFT	TERM	47	18	22.50	429.7	198.2	148.5	214.3	34.11	324.2
LDFT	TERM	48	7	143.8	818.6	383.8	235.2	334.0	228.2	466.9
LNDFH-I	PRE-TERM	29	1	115.7	115.7	115.7	NA	115.7	115.7	115.7
LNDFH-I	PRE-TERM	30	10	5.000	1679	741.0	647.0	963.9	69.83	1076
LNDFH-I	PRE-TERM	31	12	5.000	1761	779.0	630.1	816.6	70.81	1232
LNDFH-I	PRE-TERM	32	17	5.000	1781	851.4	649.8	960.8	49.54	1468
LNDFH-I	PRE-TERM	33	24	5.000	1860	876.0	647.8	994.8	36.57	1336
LNDFH-I	PRE-TERM	34	25	5.000	3123	911.0	758.7	1004	41.75	1373
LNDFH-I	PRE-TERM	35	24	5.000	2269	870.6	646.6	975.8	35.02	1287
LNDFH-I	PRE-TERM	36	25	5.000	1707	782.9	564.8	905.6	36.61	1157
LNDFH-I	PRE-TERM	37	23	5.000	1585	776.9	526.2	939.9	302.3	1167
LNDFH-I	PRE-TERM	38	15	5.000	1910	808.8	620.2	832.1	289.7	1272
LNDFH-I	PRE-TERM	39	20	5.000	1661	783.2	519.0	918.0	510.7	1088
LNDFH-I	PRE-TERM	40	10	5.000	1575	801.0	616.5	839.2	199.5	1221
LNDFH-I	PRE-TERM	41	15	5.000	1551	827.6	561.0	910.9	385.0	1261
LNDFH-I	PRE-TERM	42	9	5.000	1606	668.2	584.3	613.2	14.03	1090
LNDFH-I	PRE-TERM	43	13	5.000	1768	804.6	645.3	775.3	33.59	1147
LNDFH-I	PRE-TERM	44	8	5.000	1182	577.2	498.1	741.2	10.20	924.3
LNDFH-I	PRE-TERM	45	12	5.000	1249	600.3	469.5	707.0	26.86	980.0
LNDFH-I	PRE-TERM	46	8	5.000	1431	687.7	501.0	737.0	432.2	946.5
LNDFH-I	PRE-TERM	47	3	578.1	1327	833.6	427.2	595.8	587.0	961.3
LNDFH-I	PRE-TERM	48	5	5.000	967.5	555.8	350.0	600.4	530.1	676.3

Table S3 Concentration of Human Milk Oligosaccharides in Term or Preterm Milk At Specified Postmenstrual Age

** When there are results below the method limit of quanfification (LoQ) the result has been assigned value of 0.5 × LoQ, hence the minimum value appears to be the same in many cases. When a large number of datapoints are below LoQ this can also have the effect that the median = minimum.*

				HMO Concentration (mg/L)						
HMO	Study Arm	Postmenstrual Age (weeks)	N	min *	max	mean	sd	median *	Quartile 1 (25%)	Quartile 3 (75%)
LNDFH-I	TERM	38	2	1301	1689	1495	274.3	1495	1398	1592
LNDFH-I	TERM	39	10	5.000	1755	1106	506.9	1101	871.5	1417
LNDFH-I	TERM	40	19	5.000	2122	981.7	764.9	1015	5.000	1578
LNDFH-I	TERM	41	27	5.000	2264	1024	653.6	1093	853.8	1420
LNDFH-I	TERM	42	29	5.000	2313	948.8	684.0	1069	5.000	1334
LNDFH-I	TERM	43	28	5.000	1937	897.2	612.1	982.8	523.4	1293
LNDFH-I	TERM	44	27	5.000	1983	813.5	611.2	804.7	238.0	1175
LNDFH-I	TERM	45	28	5.000	2024	788.6	565.8	825.6	412.5	1171
LNDFH-I	TERM	46	24	5.000	2167	711.1	598.5	681.2	5.000	1024
LNDFH-I	TERM	47	18	5.000	1935	640.9	661.4	440.0	5.000	1174
LNDFH-I	TERM	48	7	5.000	1304	757.7	464.7	849.3	464.5	1108
LNFP-I	PRE-TERM	29	1	13.50	13.50	13.50	NA	13.50	13.50	13.50
LNFP-I	PRE-TERM	30	10	13.50	2639	1254	969.2	1686	203.5	1909
LNFP-I	PRE-TERM	31	12	13.50	2630	1176	902.1	1220	494.2	1669
LNFP-I	PRE-TERM	32	17	13.50	2806	1118	825.5	1197	723.1	1448
LNFP-I	PRE-TERM	33	24	13.50	2062	968.0	665.5	1058	579.8	1429
LNFP-I	PRE-TERM	34	25	13.50	1634	830.4	563.7	917.9	578.9	1351
LNFP-I	PRE-TERM	35	24	13.50	1840	734.9	519.2	725.3	439.5	993.7
LNFP-I	PRE-TERM	36	25	13.50	1773	693.8	506.4	660.3	558.8	949.2
LNFP-I	PRE-TERM	37	23	13.50	1188	604.7	399.6	628.0	401.9	952.1
LNFP-I	PRE-TERM	38	15	13.50	1126	459.2	374.5	393.9	157.7	617.6
LNFP-I	PRE-TERM	39	20	13.50	1436	537.3	373.8	508.7	370.2	759.1
LNFP-I	PRE-TERM	40	10	13.50	1075	451.4	379.4	429.0	97.57	753.4
LNFP-I	PRE-TERM	41	15	13.50	829.3	438.8	291.5	494.9	222.1	659.3
LNFP-I	PRE-TERM	42	9	13.50	966.6	365.5	339.1	301.7	13.50	649.5
LNFP-I	PRE-TERM	43	13	13.50	1014	486.6	352.6	417.3	251.8	812.1
LNFP-I	PRE-TERM	44	8	13.50	549.4	266.0	224.5	327.9	13.50	423.1
LNFP-I	PRE-TERM	45	12	13.50	933.6	357.3	326.4	264.0	114.8	636.2
LNFP-I	PRE-TERM	46	8	13.50	717.8	304.0	246.1	324.6	102.0	442.0
LNFP-I	PRE-TERM	47	3	195.2	635.0	376.5	229.8	299.3	247.3	467.2

Table S3 Concentration of Human Milk Oligosaccharides in Term or Preterm Milk At Specified Postmenstrual Age

** When there are results below the method limit of quanfification (LoQ) the result has been assigned value of 0.5 × LoQ, hence the minimum value appears to be the same in many cases. When a large number of datapoints are below LoQ this can also have the effect that the median = minimum.*

				HMO Concentration (mg/L)						
HMO	Study Arm	Postmenstrual Age (weeks)	N	min *	max	mean	sd	median *	Quartile 1 (25%)	Quartile 3 (75%)
LNFP-I	PRE-TERM	48	5	13.50	377.1	194.7	137.6	167.3	142.5	273.0
LNFP-I	TERM	38	2	1760	1768	1764	6.024	1764	1762	1766
LNFP-I	TERM	39	10	13.50	2547	1518	868.9	1530	986.7	2270
LNFP-I	TERM	40	19	13.50	3381	1253	1103	1228	13.50	1998
LNFP-I	TERM	41	27	13.50	3597	1576	1058	1613	917.8	2292
LNFP-I	TERM	42	29	13.50	3156	1194	895.3	1264	418.7	1723
LNFP-I	TERM	43	28	13.50	2655	1000	796.7	882.0	461.8	1284
LNFP-I	TERM	44	27	13.50	2647	819.8	707.1	735.2	272.0	1226
LNFP-I	TERM	45	28	13.50	1899	687.6	544.1	696.7	291.8	937.8
LNFP-I	TERM	46	24	13.50	1956	582.3	525.3	489.0	147.3	788.7
LNFP-I	TERM	47	18	13.50	2617	644.3	646.1	700.4	52.33	836.7
LNFP-I	TERM	48	7	192.3	1715	851.2	509.9	750.0	525.2	1125
LNFP-II	PRE-TERM	29	1	2094	2094	2094	NA	2094	2094	2094
LNFP-II	PRE-TERM	30	10	17.50	1740	553.6	609.0	269.7	125.2	935.4
LNFP-II	PRE-TERM	31	12	17.50	1628	556.6	547.1	307.0	213.7	649.2
LNFP-II	PRE-TERM	32	17	17.50	1775	513.3	500.4	327.7	164.5	678.9
LNFP-II	PRE-TERM	33	24	17.50	1845	576.3	547.8	341.6	193.5	683.5
LNFP-II	PRE-TERM	34	25	17.50	1950	596.8	532.1	417.8	193.2	791.2
LNFP-II	PRE-TERM	35	24	17.50	2138	623.8	564.5	406.9	264.7	783.7
LNFP-II	PRE-TERM	36	25	17.50	2220	622.4	584.2	483.2	222.8	779.9
LNFP-II	PRE-TERM	37	23	17.50	1804	577.0	495.6	452.5	240.0	742.9
LNFP-II	PRE-TERM	38	15	68.91	2024	563.5	536.7	337.4	165.7	753.0
LNFP-II	PRE-TERM	39	20	17.50	1942	532.3	454.1	417.3	205.1	765.8
LNFP-II	PRE-TERM	40	10	149.3	1468	629.9	434.4	669.5	253.9	864.6
LNFP-II	PRE-TERM	41	15	17.50	2373	502.9	591.2	323.9	198.3	550.0
LNFP-II	PRE-TERM	42	9	141.6	1432	601.2	393.6	624.3	249.7	748.0
LNFP-II	PRE-TERM	43	13	17.50	1476	463.5	363.4	427.7	207.8	528.8
LNFP-II	PRE-TERM	44	8	160.6	1571	626.4	423.4	565.8	452.8	673.2
LNFP-II	PRE-TERM	45	12	17.50	2593	571.4	692.4	373.6	266.7	497.5
LNFP-II	PRE-TERM	46	8	121.9	1367	536.2	400.2	511.3	247.1	627.5

Table S3 Concentration of Human Milk Oligosaccharides in Term or Preterm Milk At Specified Postmenstrual Age

** When there are results below the method limit of quanfification (LoQ) the result has been assigned value of 0.5 × LoQ, hence the minimum value appears to be the same in many cases. When a large number of datapoints are below LoQ this can also have the effect that the median = minimum.*

				HMO Concentration (mg/L)						
**HMO**	**Study Arm**	**Postmenstrual Age (weeks)**	**N**	**min ***	**max**	**mean**	**sd**	**median ***	**Quartile 1 (25%)**	**Quartile 3 (75%)**
LNFP-II	PRE-TERM	47	3	168.9	386.9	311.6	123.6	378.9	273.9	382.9
LNFP-II	PRE-TERM	48	5	118.0	1201	544.8	419.9	483.9	267.1	654.3
LNFP-II	TERM	38	2	222.1	251.5	236.8	20.83	236.8	229.4	244.2
LNFP-II	TERM	39	10	85.02	1238	317.3	342.7	196.2	129.8	351.4
LNFP-II	TERM	40	19	17.50	1616	554.5	486.6	344.2	245.8	832.7
LNFP-II	TERM	41	27	17.50	1884	492.9	468.4	323.3	183.4	635.6
LNFP-II	TERM	42	29	17.50	1684	548.6	516.9	366.6	203.2	649.6
LNFP-II	TERM	43	28	17.50	1565	521.4	452.8	354.1	199.5	673.9
LNFP-II	TERM	44	27	17.50	1411	446.0	383.6	280.9	201.8	572.3
LNFP-II	TERM	45	28	17.50	1476	421.4	354.0	322.8	184.8	456.3
LNFP-II	TERM	46	24	17.50	1171	430.0	340.3	319.5	171.8	589.4
LNFP-II	TERM	47	18	17.50	1272	425.7	377.5	304.2	140.1	629.5
LNFP-II	TERM	48	7	17.50	382.2	186.3	111.2	180.5	145.5	216.5
LNFP-III	PRE-TERM	29	1	682.1	682.1	682.1	NA	682.1	682.1	682.1
LNFP-III	PRE-TERM	30	10	55.72	569.3	378.2	141.8	386.6	335.5	404.2
LNFP-III	PRE-TERM	31	12	35.70	640.0	341.1	153.9	333.7	288.7	429.7
LNFP-III	PRE-TERM	32	17	40.94	654.7	298.6	152.0	275.6	236.0	352.3
LNFP-III	PRE-TERM	33	24	49.93	727.3	306.2	172.4	285.6	173.0	399.6
LNFP-III	PRE-TERM	34	25	52.14	779.2	320.9	173.2	316.4	232.4	347.4
LNFP-III	PRE-TERM	35	24	49.25	733.6	317.2	145.5	306.6	250.1	399.9
LNFP-III	PRE-TERM	36	25	17.50	711.8	305.3	140.5	300.2	214.3	365.7
LNFP-III	PRE-TERM	37	23	52.97	767.7	349.0	154.1	352.3	263.4	412.5
LNFP-III	PRE-TERM	38	15	55.78	615.4	330.9	158.8	316.3	223.0	420.8
LNFP-III	PRE-TERM	39	20	60.20	847.4	350.0	181.4	335.2	249.6	411.4
LNFP-III	PRE-TERM	40	10	175.8	534.0	314.1	126.6	268.2	233.8	418.8
LNFP-III	PRE-TERM	41	15	50.94	653.0	422.7	168.8	434.5	355.8	563.5
LNFP-III	PRE-TERM	42	9	177.7	526.8	307.9	111.3	298.4	228.7	314.2
LNFP-III	PRE-TERM	43	13	74.13	635.2	403.4	157.0	404.7	336.1	522.3
LNFP-III	PRE-TERM	44	8	204.0	528.1	329.0	127.8	271.7	232.8	441.3
LNFP-III	PRE-TERM	45	12	89.27	599.0	347.5	166.0	356.4	226.1	441.3

Table S3 Concentration of Human Milk Oligosaccharides in Term or Preterm Milk At Specified Postmenstrual Age

** When there are results below the method limit of quanfification (LoQ) the result has been assigned value of 0.5 × LoQ, hence the minimum value appears to be the same in many cases. When a large number of datapoints are below LoQ this can also have the effect that the median = minimum.*

				HMO Concentration (mg/L)						
HMO	Study Arm	Postmenstrual Age (weeks)	N	min *	max	mean	sd	median *	Quartile 1 (25%)	Quartile 3 (75%)
LNFP-III	PRE-TERM	46	8	187.7	750.3	379.7	183.1	352.6	268.0	422.8
LNFP-III	PRE-TERM	47	3	140.7	774.0	387.5	339.0	247.7	194.2	510.9
LNFP-III	PRE-TERM	48	5	266.6	345.5	305.7	37.30	309.2	268.8	338.5
LNFP-III	TERM	38	2	444.4	477.8	461.1	23.62	461.1	452.8	469.5
LNFP-III	TERM	39	10	192.5	826.9	414.9	174.3	413.4	317.8	465.4
LNFP-III	TERM	40	19	76.48	1268	423.9	254.3	390.8	321.4	454.5
LNFP-III	TERM	41	27	169.8	1169	361.6	190.9	327.1	236.8	420.8
LNFP-III	TERM	42	29	153.7	911.0	319.0	145.9	288.8	233.0	339.0
LNFP-III	TERM	43	28	165.5	823.9	306.6	141.6	264.8	221.7	338.2
LNFP-III	TERM	44	27	153.3	714.0	290.3	111.8	269.2	223.3	322.4
LNFP-III	TERM	45	28	17.50	786.1	310.5	140.9	291.6	229.8	361.9
LNFP-III	TERM	46	24	108.0	703.6	322.4	126.2	303.7	235.1	377.2
LNFP-III	TERM	47	18	164.2	569.7	312.3	116.4	269.7	234.1	357.3
LNFP-III	TERM	48	7	209.5	398.2	279.2	63.68	262.5	239.4	302.8
LNFP-V	PRE-TERM	29	1	320.9	320.9	320.9	NA	320.9	320.9	320.9
LNFP-V	PRE-TERM	30	10	12.00	289.0	76.92	97.54	21.27	12.00	119.8
LNFP-V	PRE-TERM	31	12	12.00	266.9	81.16	90.54	44.14	22.49	92.26
LNFP-V	PRE-TERM	32	17	12.00	286.3	74.16	84.38	40.14	12.00	88.93
LNFP-V	PRE-TERM	33	24	12.00	300.2	81.13	94.73	39.11	12.00	84.76
LNFP-V	PRE-TERM	34	25	12.00	317.8	85.63	92.32	57.47	25.18	92.28
LNFP-V	PRE-TERM	35	24	12.00	359.5	92.63	97.67	61.18	32.02	102.6
LNFP-V	PRE-TERM	36	25	12.00	363.8	89.74	98.44	53.67	12.00	106.3
LNFP-V	PRE-TERM	37	23	12.00	285.4	79.91	79.86	51.51	30.57	91.35
LNFP-V	PRE-TERM	38	15	12.00	309.0	83.19	89.48	36.45	12.00	116.1
LNFP-V	PRE-TERM	39	20	12.00	299.1	75.75	74.97	48.92	25.20	98.53
LNFP-V	PRE-TERM	40	10	12.00	207.0	89.15	72.85	90.29	25.92	114.3
LNFP-V	PRE-TERM	41	15	12.00	328.8	66.61	84.84	36.69	19.45	69.63
LNFP-V	PRE-TERM	42	9	12.00	165.1	76.96	56.06	85.68	12.00	111.5
LNFP-V	PRE-TERM	43	13	12.00	200.7	57.88	48.98	46.12	28.01	68.72
LNFP-V	PRE-TERM	44	8	12.00	194.4	82.49	57.29	71.97	52.95	106.7

Table S3 Concentration of Human Milk Oligosaccharides in Term or Preterm Milk At Specified Postmenstrual Age

** When there are results below the method limit of quanfification (LoQ) the result has been assigned value of 0.5 × LoQ, hence the minimum value appears to be the same in many cases. When a large number of datapoints are below LoQ this can also have the effect that the median = minimum.*

				HMO Concentration (mg/L)						
HMO	Study Arm	Postmenstrual Age (weeks)	N	min *	max	mean	sd	median *	Quartile 1 (25%)	Quartile 3 (75%)
LNFP-V	PRE-TERM	45	12	12.00	408.7	79.84	109.6	40.67	28.29	71.91
LNFP-V	PRE-TERM	46	8	12.00	202.6	68.99	61.58	64.62	24.09	82.31
LNFP-V	PRE-TERM	47	3	12.00	47.17	31.89	18.03	36.51	24.26	41.84
LNFP-V	PRE-TERM	48	5	12.00	169.4	66.40	61.49	52.56	29.49	68.59
LNFP-V	TERM	38	2	12.00	30.70	21.35	13.23	21.35	16.68	26.03
LNFP-V	TERM	39	10	12.00	138.6	38.49	40.92	18.65	12.00	48.24
LNFP-V	TERM	40	19	12.00	203.7	79.83	68.39	54.21	24.86	135.8
LNFP-V	TERM	41	27	12.00	349.1	82.07	87.14	49.87	33.34	70.70
LNFP-V	TERM	42	29	12.00	247.0	76.53	67.41	52.36	35.88	75.41
LNFP-V	TERM	43	28	12.00	202.6	65.25	60.47	45.53	12.00	76.82
LNFP-V	TERM	44	27	12.00	228.2	63.66	56.31	41.53	26.81	74.83
LNFP-V	TERM	45	28	12.00	226.4	59.85	52.18	45.12	26.25	68.26
LNFP-V	TERM	46	24	12.00	162.9	59.97	43.87	54.98	27.51	87.87
LNFP-V	TERM	47	18	12.00	196.7	62.33	52.74	55.57	12.00	95.88
LNFP-V	TERM	48	7	12.00	38.23	17.47	10.19	12.00	12.00	18.02
LNnDFH	PRE-TERM	29	1	543.1	543.1	543.1	NA	543.1	543.1	543.1
LNnDFH	PRE-TERM	30	10	14.00	299.7	65.58	87.74	36.61	14.00	58.54
LNnDFH	PRE-TERM	31	12	14.00	333.0	58.57	89.41	30.92	14.00	56.50
LNnDFH	PRE-TERM	32	17	14.00	195.3	33.14	44.88	14.00	14.00	39.02
LNnDFH	PRE-TERM	33	24	14.00	430.0	49.09	93.24	14.00	14.00	17.77
LNnDFH	PRE-TERM	34	25	14.00	196.9	44.37	53.81	14.00	14.00	40.63
LNnDFH	PRE-TERM	35	24	14.00	185.4	39.71	49.06	14.00	14.00	48.78
LNnDFH	PRE-TERM	36	25	14.00	156.3	29.22	33.00	14.00	14.00	35.39
LNnDFH	PRE-TERM	37	23	14.00	183.2	37.05	45.59	14.00	14.00	33.87
LNnDFH	PRE-TERM	38	15	14.00	119.8	44.69	41.10	14.00	14.00	78.15
LNnDFH	PRE-TERM	39	20	14.00	107.7	29.96	25.58	14.00	14.00	36.04
LNnDFH	PRE-TERM	40	10	14.00	115.8	39.15	36.74	21.33	14.00	49.21
LNnDFH	PRE-TERM	41	15	14.00	85.19	34.09	26.33	14.00	14.00	48.12
LNnDFH	PRE-TERM	42	9	14.00	125.7	42.22	39.14	29.34	14.00	46.53
LNnDFH	PRE-TERM	43	13	14.00	75.44	27.19	20.62	14.00	14.00	35.14

Table S3 Concentration of Human Milk Oligosaccharides in Term or Preterm Milk At Specified Postmenstrual Age

** When there are results below the method limit of quanfification (LoQ) the result has been assigned value of 0.5 × LoQ, hence the minimum value appears to be the same in many cases. When a large number of datapoints are below LoQ this can also have the effect that the median = minimum.*

				HMO Concentration (mg/L)						
HMO	Study Arm	Postmenstrual Age (weeks)	N	min *	max	mean	sd	median *	Quartile 1 (25%)	Quartile 3 (75%)
LNnDFH	PRE-TERM	44	8	14.00	109.1	45.82	35.13	35.98	14.00	69.41
LNnDFH	PRE-TERM	45	12	14.00	67.12	28.88	19.99	14.00	14.00	48.57
LNnDFH	PRE-TERM	46	8	14.00	733.1	123.6	247.3	40.52	14.00	60.41
LNnDFH	PRE-TERM	47	3	14.00	105.1	44.36	52.59	14.00	14.00	59.54
LNnDFH	PRE-TERM	48	5	14.00	94.85	45.30	33.64	48.19	14.00	55.46
LNnDFH	TERM	38	2	50.53	106.3	78.39	39.40	78.39	64.46	92.32
LNnDFH	TERM	39	10	14.00	181.8	48.42	54.80	21.27	14.00	66.86
LNnDFH	TERM	40	19	14.00	172.8	47.32	42.27	31.75	14.00	61.65
LNnDFH	TERM	41	27	14.00	110.0	28.38	24.84	14.00	14.00	38.58
LNnDFH	TERM	42	29	14.00	52.71	22.80	13.03	14.00	14.00	32.72
LNnDFH	TERM	43	28	14.00	77.46	24.52	16.48	14.00	14.00	33.95
LNnDFH	TERM	44	27	14.00	85.76	21.69	16.36	14.00	14.00	21.39
LNnDFH	TERM	45	28	14.00	132.3	29.27	31.72	14.00	14.00	18.78
LNnDFH	TERM	46	24	14.00	94.89	25.57	22.95	14.00	14.00	19.52
LNnDFH	TERM	47	18	14.00	94.07	29.09	26.13	14.00	14.00	29.94
LNnDFH	TERM	48	7	14.00	70.18	27.23	20.80	14.00	14.00	32.22
LNnFP	PRE-TERM	29	1	28.70	28.70	28.70	NA	28.70	28.70	28.70
LNnFP	PRE-TERM	30	10	9.500	89.76	23.87	24.54	14.98	9.500	26.85
LNnFP	PRE-TERM	31	12	9.500	50.62	17.40	12.43	9.500	9.500	22.03
LNnFP	PRE-TERM	32	17	9.500	50.34	13.75	12.10	9.500	9.500	9.500
LNnFP	PRE-TERM	33	24	9.500	59.82	17.12	13.54	9.500	9.500	19.57
LNnFP	PRE-TERM	34	25	9.500	61.67	14.36	11.41	9.500	9.500	9.500
LNnFP	PRE-TERM	35	24	9.500	60.47	16.64	13.03	9.500	9.500	20.39
LNnFP	PRE-TERM	36	25	9.500	55.24	15.30	11.82	9.500	9.500	20.00
LNnFP	PRE-TERM	37	23	9.500	57.65	14.28	10.89	9.500	9.500	14.26
LNnFP	PRE-TERM	38	15	9.500	32.68	13.15	8.015	9.500	9.500	9.500
LNnFP	PRE-TERM	39	20	9.500	60.19	16.76	15.42	9.500	9.500	12.56
LNnFP	PRE-TERM	40	10	9.500	9.500	9.500	0.0000	9.500	9.500	9.500
LNnFP	PRE-TERM	41	15	9.500	53.23	17.04	14.37	9.500	9.500	14.94
LNnFP	PRE-TERM	42	9	9.500	21.55	10.84	4.017	9.500	9.500	9.500

Table S3 Concentration of Human Milk Oligosaccharides in Term or Preterm Milk At Specified Postmenstrual Age

** When there are results below the method limit of quanfification (LoQ) the result has been assigned value of 0.5 × LoQ, hence the minimum value appears to be the same in many cases. When a large number of datapoints are below LoQ this can also have the effect that the median = minimum.*

				HMO Concentration (mg/L)						
HMO	Study Arm	Postmenstrual Age (weeks)	N	min *	max	mean	sd	median *	Quartile 1 (25%)	Quartile 3 (75%)
LNnFP	PRE-TERM	43	13	9.500	70.04	16.87	18.72	9.500	9.500	9.500
LNnFP	PRE-TERM	44	8	9.500	22.23	12.40	5.409	9.500	9.500	12.12
LNnFP	PRE-TERM	45	12	9.500	88.05	19.49	24.64	9.500	9.500	9.500
LNnFP	PRE-TERM	46	8	9.500	22.67	11.15	4.657	9.500	9.500	9.500
LNnFP	PRE-TERM	47	3	9.500	56.14	25.05	26.93	9.500	9.500	32.82
LNnFP	PRE-TERM	48	5	9.500	23.76	12.35	6.376	9.500	9.500	9.500
LNnFP	TERM	38	2	9.500	19.80	14.65	7.285	14.65	12.08	17.23
LNnFP	TERM	39	10	9.500	49.07	19.22	15.92	9.500	9.500	31.04
LNnFP	TERM	40	19	9.500	56.56	19.88	14.79	9.500	9.500	27.35
LNnFP	TERM	41	27	9.500	29.02	11.98	5.501	9.500	9.500	9.500
LNnFP	TERM	42	29	9.500	49.35	13.97	9.452	9.500	9.500	9.500
LNnFP	TERM	43	28	9.500	66.17	15.38	12.51	9.500	9.500	19.35
LNnFP	TERM	44	27	9.500	52.21	12.74	8.869	9.500	9.500	9.500
LNnFP	TERM	45	28	9.500	56.13	13.96	9.658	9.500	9.500	19.18
LNnFP	TERM	46	24	9.500	37.85	14.11	7.439	9.500	9.500	20.27
LNnFP	TERM	47	18	9.500	28.50	13.45	6.782	9.500	9.500	17.67
LNnFP	TERM	48	7	9.500	9.500	9.500	0.0000	9.500	9.500	9.500
LNnT	PRE-TERM	29	1	165.4	165.4	165.4	NA	165.4	165.4	165.4
LNnT	PRE-TERM	30	10	137.3	546.3	311.4	128.0	290.4	222.9	394.7
LNnT	PRE-TERM	31	12	118.3	435.5	253.3	97.79	272.6	149.0	301.6
LNnT	PRE-TERM	32	17	79.69	413.1	214.0	89.45	216.4	154.6	258.6
LNnT	PRE-TERM	33	24	67.88	404.7	196.7	87.06	197.6	126.1	239.0
LNnT	PRE-TERM	34	25	66.51	383.2	193.1	99.31	182.2	106.3	240.1
LNnT	PRE-TERM	35	24	60.11	397.8	186.6	102.0	182.2	97.08	248.7
LNnT	PRE-TERM	36	25	43.09	341.2	169.5	85.23	167.2	96.86	244.2
LNnT	PRE-TERM	37	23	48.51	372.0	167.9	81.05	163.5	102.6	206.5
LNnT	PRE-TERM	38	15	12.00	314.7	132.5	91.65	118.6	70.68	162.1
LNnT	PRE-TERM	39	20	33.45	440.8	168.9	96.22	166.3	96.12	215.4
LNnT	PRE-TERM	40	10	36.42	211.5	134.9	68.75	131.3	85.50	202.4
LNnT	PRE-TERM	41	15	27.39	464.0	151.6	115.6	140.1	71.45	166.0

Table S3 Concentration of Human Milk Oligosaccharides in Term or Preterm Milk At Specified Postmenstrual Age

** When there are results below the method limit of quanfification (LoQ) the result has been assigned value of 0.5 × LoQ, hence the minimum value appears to be the same in many cases. When a large number of datapoints are below LoQ this can also have the effect that the median = minimum.*

HMO	Study Arm	Postmenstrual Age (weeks)	N	HMO Concentration (mg/L) min *	max	mean	sd	median *	Quartile 1 (25%)	Quartile 3 (75%)
LNnT	PRE-TERM	42	9	24.44	279.5	117.4	80.66	95.54	77.16	144.7
LNnT	PRE-TERM	43	13	12.00	379.4	134.9	101.5	110.0	81.67	174.6
LNnT	PRE-TERM	44	8	32.38	229.8	110.2	67.42	109.4	59.42	140.0
LNnT	PRE-TERM	45	12	12.00	444.3	130.1	126.4	79.71	39.86	166.9
LNnT	PRE-TERM	46	8	38.00	180.1	106.2	47.79	88.84	82.30	148.1
LNnT	PRE-TERM	47	3	34.77	437.3	184.3	220.3	80.91	57.84	259.1
LNnT	PRE-TERM	48	5	25.55	208.8	108.4	71.92	80.85	75.22	151.8
LNnT	TERM	38	2	377.9	400.4	389.2	15.95	389.2	383.5	394.8
LNnT	TERM	39	10	162.4	565.3	356.0	136.6	372.1	253.5	457.5
LNnT	TERM	40	19	113.9	452.2	279.2	93.31	275.6	220.7	341.0
LNnT	TERM	41	27	67.58	435.1	253.5	94.96	258.9	181.4	313.8
LNnT	TERM	42	29	52.63	350.3	182.9	71.69	179.2	129.4	222.7
LNnT	TERM	43	28	54.25	369.5	165.2	77.77	151.5	112.9	218.0
LNnT	TERM	44	27	12.00	311.2	141.5	73.49	135.3	92.80	174.9
LNnT	TERM	45	28	12.00	281.9	144.7	67.45	138.6	103.3	183.5
LNnT	TERM	46	24	35.80	284.9	131.2	66.18	133.8	76.93	150.8
LNnT	TERM	47	18	27.23	245.5	123.0	54.53	124.3	79.41	159.2
LNnT	TERM	48	7	64.38	195.3	108.0	43.21	101.1	82.14	115.5
LNT	PRE-TERM	29	1	3029	3029	3029	NA	3029	3029	3029
LNT	PRE-TERM	30	10	630.4	3296	1224	841.8	869.2	720.9	1315
LNT	PRE-TERM	31	12	965.1	2903	1489	567.3	1194	1099	1718
LNT	PRE-TERM	32	17	298.8	2283	1309	564.6	1167	916.9	1816
LNT	PRE-TERM	33	24	341.5	2537	1278	576.4	1168	892.1	1758
LNT	PRE-TERM	34	25	653.0	2851	1333	642.7	1059	880.4	1628
LNT	PRE-TERM	35	24	479.3	2498	1332	625.0	1167	877.4	1723
LNT	PRE-TERM	36	25	469.1	2737	1252	668.7	1127	609.9	1563
LNT	PRE-TERM	37	23	356.6	2468	1066	529.5	1116	626.0	1323
LNT	PRE-TERM	38	15	144.1	1942	921.6	593.8	789.0	400.3	1362
LNT	PRE-TERM	39	20	332.2	2376	978.1	586.6	765.9	557.5	1386
LNT	PRE-TERM	40	10	317.3	2184	1059	655.9	1051	468.0	1378

Table S3 Concentration of Human Milk Oligosaccharides in Term or Preterm Milk At Specified Postmenstrual Age

** When there are results below the method limit of quanfification (LoQ) the result has been assigned value of 0.5 × LoQ, hence the minimum value appears to be the same in many cases. When a large number of datapoints are below LoQ this can also have the effect that the median = minimum.*

				HMO Concentration (mg/L)						
HMO	Study Arm	Postmenstrual Age (weeks)	N	min *	max	mean	sd	median *	Quartile 1 (25%)	Quartile 3 (75%)
LNT	PRE-TERM	41	15	175.3	1554	715.2	392.1	658.7	478.3	932.0
LNT	PRE-TERM	42	9	287.6	1271	821.3	354.9	909.4	614.5	1043
LNT	PRE-TERM	43	13	304.6	1191	712.2	315.8	675.4	502.5	1056
LNT	PRE-TERM	44	8	301.3	1051	777.6	252.6	847.9	701.9	912.8
LNT	PRE-TERM	45	12	145.2	2113	728.1	514.0	686.9	417.4	830.5
LNT	PRE-TERM	46	8	232.8	1162	623.6	323.0	543.0	423.7	771.6
LNT	PRE-TERM	47	3	248.2	773.5	569.6	281.7	687.1	467.6	730.3
LNT	PRE-TERM	48	5	205.0	879.6	548.4	314.6	597.9	242.5	816.9
LNT	TERM	38	2	598.9	923.2	761.1	229.3	761.1	680.0	842.1
LNT	TERM	39	10	199.8	1253	694.0	410.7	568.7	370.0	1092
LNT	TERM	40	19	214.3	3400	1351	731.6	1205	855.9	1507
LNT	TERM	41	27	359.0	3848	1275	706.2	1181	730.9	1467
LNT	TERM	42	29	358.7	3092	1289	554.9	1206	985.8	1639
LNT	TERM	43	28	359.8	3087	1176	607.2	1180	697.1	1354
LNT	TERM	44	27	313.1	2480	1026	581.5	846.7	602.5	1318
LNT	TERM	45	28	307.2	2109	902.6	495.5	762.8	571.2	1158
LNT	TERM	46	24	213.7	2251	877.0	498.1	789.0	520.3	1179
LNT	TERM	47	18	217.7	1852	892.8	503.1	830.2	480.1	1263
LNT	TERM	48	7	269.1	856.9	526.3	243.8	462.5	314.6	733.1
LSTb	PRE-TERM	29	1	183.1	183.1	183.1	NA	183.1	183.1	183.1
LSTb	PRE-TERM	30	10	54.76	159.2	88.48	32.50	81.22	65.26	95.82
LSTb	PRE-TERM	31	12	71.27	182.1	114.7	37.18	109.5	83.08	147.9
LSTb	PRE-TERM	32	17	44.25	217.4	108.8	54.55	100.9	62.51	151.5
LSTb	PRE-TERM	33	24	41.49	256.1	111.8	58.27	91.29	68.50	146.2
LSTb	PRE-TERM	34	25	49.85	334.7	113.6	59.09	100.3	86.22	133.5
LSTb	PRE-TERM	35	24	47.34	314.7	118.8	58.95	115.3	80.11	142.6
LSTb	PRE-TERM	36	25	42.09	423.9	108.6	76.33	99.07	59.00	129.6
LSTb	PRE-TERM	37	23	37.86	400.7	107.7	73.27	95.27	57.96	131.8
LSTb	PRE-TERM	38	15	7.000	161.7	88.58	45.97	92.79	50.74	127.6
LSTb	PRE-TERM	39	20	38.99	156.5	89.38	39.22	87.17	50.34	122.2

Table S3 Concentration of Human Milk Oligosaccharides in Term or Preterm Milk At Specified Postmenstrual Age

** When there are results below the method limit of quanfification (LoQ) the result has been assigned value of 0.5 × LoQ, hence the minimum value appears to be the same in many cases. When a large number of datapoints are below LoQ this can also have the effect that the median = minimum.*

				HMO Concentration (mg/L)						
HMO	Study Arm	Postmenstrual Age (weeks)	N	min *	max	mean	sd	median *	Quartile 1 (25%)	Quartile 3 (75%)
LSTb	PRE-TERM	40	10	48.94	146.3	99.10	35.48	105.0	66.00	129.3
LSTb	PRE-TERM	41	15	34.87	405.6	106.5	94.87	77.14	48.20	120.6
LSTb	PRE-TERM	42	9	39.45	143.0	86.36	40.30	85.72	51.40	105.5
LSTb	PRE-TERM	43	13	31.54	243.3	101.3	60.09	91.07	54.05	113.3
LSTb	PRE-TERM	44	8	43.51	140.5	78.68	30.83	77.98	61.18	87.59
LSTb	PRE-TERM	45	12	33.70	247.3	90.12	53.23	83.33	70.10	91.55
LSTb	PRE-TERM	46	8	25.18	114.5	77.32	27.06	82.46	63.98	93.05
LSTb	PRE-TERM	47	3	36.39	84.41	61.71	24.12	64.34	50.36	74.38
LSTb	PRE-TERM	48	5	23.33	106.9	61.31	31.49	51.67	49.11	75.54
LSTb	TERM	38	2	56.67	94.71	75.69	26.90	75.69	66.18	85.20
LSTb	TERM	39	10	33.70	102.3	68.80	20.81	69.85	54.57	81.62
LSTb	TERM	40	19	28.89	196.6	87.92	44.00	81.00	57.45	98.49
LSTb	TERM	41	27	15.52	160.5	79.79	34.79	72.16	53.55	102.7
LSTb	TERM	42	29	32.58	163.7	81.61	30.88	77.06	57.48	106.5
LSTb	TERM	43	28	36.28	127.3	78.93	28.02	81.08	55.97	98.34
LSTb	TERM	44	27	20.15	166.6	77.71	34.58	70.91	56.40	108.1
LSTb	TERM	45	28	7.000	147.4	71.07	36.37	63.39	46.25	95.69
LSTb	TERM	46	24	18.33	177.0	73.74	40.61	66.97	45.66	100.7
LSTb	TERM	47	18	7.000	152.6	69.85	44.19	62.95	33.26	108.2
LSTb	TERM	48	7	22.20	119.2	64.07	35.39	55.09	38.07	87.93
LSTc	PRE-TERM	29	1	184.0	184.0	184.0	NA	184.0	184.0	184.0
LSTc	PRE-TERM	30	10	95.07	747.9	402.2	245.4	465.3	148.5	595.8
LSTc	PRE-TERM	31	12	37.98	629.5	321.4	209.4	341.3	137.2	466.4
LSTc	PRE-TERM	32	17	31.79	1019	312.5	245.4	284.3	136.2	439.1
LSTc	PRE-TERM	33	24	28.75	767.6	267.0	209.7	207.4	114.8	304.7
LSTc	PRE-TERM	34	25	24.49	622.9	204.5	140.1	199.2	101.3	244.8
LSTc	PRE-TERM	35	24	23.78	402.8	166.3	94.56	140.5	88.29	228.6
LSTc	PRE-TERM	36	25	17.24	371.1	133.9	88.26	102.6	69.44	180.2
LSTc	PRE-TERM	37	23	17.82	355.6	121.2	78.50	111.0	62.29	139.3
LSTc	PRE-TERM	38	15	20.21	211.2	98.81	56.26	95.39	55.35	110.1

Table S3 Concentration of Human Milk Oligosaccharides in Term or Preterm Milk At Specified Postmenstrual Age

** When there are results below the method limit of quanfification (LoQ) the result has been assigned value of 0.5 × LoQ, hence the minimum value appears to be the same in many cases. When a large number of datapoints are below LoQ this can also have the effect that the median = minimum.*

				HMO Concentration (mg/L)						
HMO	Study Arm	Postmenstrual Age (weeks)	N	min *	max	mean	sd	median *	Quartile 1 (25%)	Quartile 3 (75%)
LSTc	PRE-TERM	39	20	22.11	211.9	89.33	50.87	79.03	59.97	114.4
LSTc	PRE-TERM	40	10	36.31	151.3	79.19	36.93	61.32	59.22	100.1
LSTc	PRE-TERM	41	15	20.28	209.1	68.29	46.60	55.68	38.33	82.43
LSTc	PRE-TERM	42	9	28.83	125.3	60.84	29.79	48.35	46.32	71.72
LSTc	PRE-TERM	43	13	10.91	91.88	39.25	21.77	35.01	24.47	49.47
LSTc	PRE-TERM	44	8	22.05	100.6	51.84	30.13	38.40	30.32	71.35
LSTc	PRE-TERM	45	12	9.258	102.5	34.12	30.92	21.42	15.13	34.81
LSTc	PRE-TERM	46	8	22.75	68.68	42.58	17.38	41.04	27.74	52.78
LSTc	PRE-TERM	47	3	12.11	96.78	44.70	45.57	25.21	18.66	61.00
LSTc	PRE-TERM	48	5	14.03	67.61	33.39	22.72	21.13	18.59	45.59
LSTc	TERM	38	2	329.6	575.3	452.4	173.7	452.4	391.0	513.9
LSTc	TERM	39	10	215.4	1199	565.8	303.8	492.9	370.4	624.9
LSTc	TERM	40	19	36.80	1169	544.5	338.4	461.0	302.2	752.5
LSTc	TERM	41	27	103.3	1156	477.6	239.7	496.0	340.9	555.2
LSTc	TERM	42	29	64.34	620.3	298.8	128.5	297.0	217.0	388.5
LSTc	TERM	43	28	32.73	367.7	208.3	91.49	203.2	132.4	283.1
LSTc	TERM	44	27	30.19	254.9	148.8	54.82	153.7	113.7	191.8
LSTc	TERM	45	28	20.07	282.9	130.8	69.18	107.5	95.05	165.3
LSTc	TERM	46	24	16.92	261.1	104.5	58.28	95.62	74.55	120.4
LSTc	TERM	47	18	36.24	221.6	91.31	53.74	72.93	57.85	110.3
LSTc	TERM	48	7	37.33	141.6	69.39	33.98	61.01	53.88	69.03
MFLNH-III	PRE-TERM	29	1	166.0	166.0	166.0	NA	166.0	166.0	166.0
MFLNH-III	PRE-TERM	30	10	70.38	452.0	236.0	126.4	220.9	156.0	269.1
MFLNH-III	PRE-TERM	31	12	58.75	724.8	370.6	206.1	333.7	229.2	518.2
MFLNH-III	PRE-TERM	32	17	98.01	743.2	346.9	185.6	314.2	200.7	423.2
MFLNH-III	PRE-TERM	33	24	71.20	820.2	350.6	184.3	338.0	206.5	445.0
MFLNH-III	PRE-TERM	34	25	65.81	856.2	339.6	199.4	358.5	170.6	454.7
MFLNH-III	PRE-TERM	35	24	64.26	790.5	320.4	173.3	283.2	232.1	364.6
MFLNH-III	PRE-TERM	36	25	77.34	753.0	332.7	152.2	329.7	234.5	397.0
MFLNH-III	PRE-TERM	37	23	66.95	438.0	265.9	105.8	259.1	191.0	352.6

Table S3 Concentration of Human Milk Oligosaccharides in Term or Preterm Milk At Specified Postmenstrual Age

** When there are results below the method limit of quanfification (LoQ) the result has been assigned value of 0.5 × LoQ, hence the minimum value appears to be the same in many cases. When a large number of datapoints are below LoQ this can also have the effect that the median = minimum.*

				HMO Concentration (mg/L)						
**HMO**	**Study Arm**	**Postmenstrual Age (weeks)**	**N**	**min ***	**max**	**mean**	**sd**	**median ***	**Quartile 1 (25%)**	**Quartile 3 (75%)**
MFLNH-III	PRE-TERM	38	15	64.97	707.1	260.8	172.6	236.9	146.6	321.4
MFLNH-III	PRE-TERM	39	20	58.71	492.1	213.7	120.4	188.1	132.5	269.0
MFLNH-III	PRE-TERM	40	10	79.18	551.6	263.6	159.7	267.9	131.9	378.6
MFLNH-III	PRE-TERM	41	15	17.50	279.2	112.0	76.63	95.92	67.56	116.0
MFLNH-III	PRE-TERM	42	9	50.65	386.3	212.6	101.7	221.7	150.6	245.5
MFLNH-III	PRE-TERM	43	13	17.50	323.4	101.5	76.09	86.39	58.13	125.0
MFLNH-III	PRE-TERM	44	8	81.42	337.9	178.5	89.47	155.2	121.3	204.7
MFLNH-III	PRE-TERM	45	12	17.50	199.1	84.10	48.60	77.52	51.54	107.5
MFLNH-III	PRE-TERM	46	8	53.20	324.5	131.5	92.16	101.1	76.76	135.6
MFLNH-III	PRE-TERM	47	3	49.98	185.3	107.4	69.94	86.82	68.40	136.0
MFLNH-III	PRE-TERM	48	5	17.50	231.9	117.7	91.35	89.25	56.39	193.4
MFLNH-III	TERM	38	2	137.0	188.4	162.7	36.40	162.7	149.8	175.6
MFLNH-III	TERM	39	10	17.50	469.8	193.5	151.5	156.7	70.76	311.2
MFLNH-III	TERM	40	19	17.50	613.8	253.5	139.0	236.2	151.3	329.4
MFLNH-III	TERM	41	27	121.3	772.4	376.8	178.1	337.0	290.2	430.9
MFLNH-III	TERM	42	29	194.2	934.4	443.2	196.3	380.0	321.3	505.6
MFLNH-III	TERM	43	28	81.47	976.4	417.8	240.3	345.0	259.6	541.9
MFLNH-III	TERM	44	27	17.50	886.4	366.5	219.1	324.0	223.7	462.2
MFLNH-III	TERM	45	28	17.50	758.8	294.6	160.6	272.2	154.0	397.6
MFLNH-III	TERM	46	24	17.50	600.2	263.8	151.0	255.6	160.8	328.4
MFLNH-III	TERM	47	18	57.71	875.6	300.8	177.1	284.7	193.7	379.3
MFLNH-III	TERM	48	7	108.5	299.2	211.8	67.79	194.4	179.1	261.3
