# Supplementary material for: Human Milk Oligosaccharides in the Milk of Mothers Delivering Term versus Preterm Infants
Source: Nutrients. 2019 Jun 5;11(6):1282. doi: 10.3390/nu11061282 (PMC6627155; doi:10.3390/nu11061282)
Supplement: Supplementary file 1 [file nutrients-11-01282-s001.zip › nutrients-512673-supplementary/Supp_Table_S4_HMO_PM_Milk_Group.pdf]

Table S4 Concentration of Human Milk Oligosaccharides in Term or Preterm Milk At Specified Postmenstrual Age Separated By Milk Group

** When there are results below the method limit of quanfification (LoQ) the result has been assigned value of 0.5 × LoQ, hence the minimum value appears to be the same in many cases. When a large number of datapoints are below LoQ this can also have the effect that the median = minimum.*

					HMO Concentration (mg/L)						
HMO	Milk Group	Study Arm	Postmenstrual Age (weeks)	N	min *	max	mean	sd	median *	Quartile 1 (25%)	Quartile 3 (75%)
2'FL	G1	PRE-TERM	30	7	53.00	3958	2873	1332	3370	2859	3506
2'FL	G1	PRE-TERM	31	9	68.21	3798	2092	1016	2333	1959	2382
2'FL	G1	PRE-TERM	32	13	40.96	4196	2294	1187	2089	1723	3095
2'FL	G1	PRE-TERM	33	18	35.03	5478	2367	1175	2372	1637	2728
2'FL	G1	PRE-TERM	34	19	37.54	3592	1979	853.8	1834	1527	2758
2'FL	G1	PRE-TERM	35	18	32.13	2996	1847	726.6	1844	1573	2348
2'FL	G1	PRE-TERM	36	19	30.17	3088	1931	755.9	1846	1665	2418
2'FL	G1	PRE-TERM	37	18	27.93	3335	1895	770.8	1797	1539	2317
2'FL	G1	PRE-TERM	38	11	250.1	3006	2136	810.5	2199	1801	2723
2'FL	G1	PRE-TERM	39	16	22.31	3151	1846	840.2	1656	1332	2509
2'FL	G1	PRE-TERM	40	7	951.5	3353	2090	849.6	1740	1576	2717
2'FL	G1	PRE-TERM	41	12	19.65	3574	1966	965.0	1908	1430	2460
2'FL	G1	PRE-TERM	42	6	861.6	2926	1841	835.4	1507	1436	2501
2'FL	G1	PRE-TERM	43	10	32.90	2847	1627	788.9	1658	1224	2066
2'FL	G1	PRE-TERM	44	5	671.4	2687	1652	779.5	1529	1252	2124
2'FL	G1	PRE-TERM	45	9	30.28	2198	1385	737.2	1421	924.1	2096
2'FL	G1	PRE-TERM	46	6	716.4	2946	1840	865.4	1720	1294	2517
2'FL	G1	PRE-TERM	47	3	956.7	2203	1753	691.7	2099	1528	2151
2'FL	G1	PRE-TERM	48	4	625.6	3455	1778	1195	1515	1252	2040
2'FL	G1	TERM	38	2	3186	4230	3708	738.3	3708	3447	3969
2'FL	G1	TERM	39	9	2269	5570	3847	1360	3491	2571	4999
2'FL	G1	TERM	40	13	1676	4305	2966	726.5	3113	2302	3420
2'FL	G1	TERM	41	21	1518	4950	2915	980.5	2760	2140	3199
2'FL	G1	TERM	42	21	1387	3876	2553	666.4	2461	1977	3082
2'FL	G1	TERM	43	21	1325	4225	2463	787.2	2482	1682	2870
2'FL	G1	TERM	44	20	1028	3370	2262	741.9	2221	1580	3003
2'FL	G1	TERM	45	21	622.0	3962	2243	885.2	2242	1739	3007
2'FL	G1	TERM	46	17	241.7	3099	2063	789.4	2151	1617	2719
2'FL	G1	TERM	47	12	715.0	3067	2184	792.9	2271	1529	2941
2'FL	G1	TERM	48	6	1399	3201	2557	670.5	2785	2292	2981
2'FL	G2	PRE-TERM	29	1	35.03	35.03	35.03	NA	35.03	35.03	35.03
2'FL	G2	PRE-TERM	30	2	21.88	26.98	24.43	3.606	24.43	23.16	25.71
2'FL	G2	PRE-TERM	31	2	6.500	18.12	12.31	8.215	12.31	9.405	15.21
2'FL	G2	PRE-TERM	32	3	6.500	116.4	45.55	61.46	13.76	10.13	65.07
2'FL	G2	PRE-TERM	33	5	6.500	48.64	23.04	22.66	6.500	6.500	47.07
2'FL	G2	PRE-TERM	34	5	6.500	30.75	12.69	10.50	6.500	6.500	13.19

Table S4 Concentration of Human Milk Oligosaccharides in Term or Preterm Milk At Specified Postmenstrual Age Separated By Milk Group

** When there are results below the method limit of quanfification (LoQ) the result has been assigned value of 0.5 × LoQ, hence the minimum value appears to be the same in many cases. When a large number of datapoints are below LoQ this can also have the effect that the median = minimum.*

					HMO Concentration (mg/L)						
HMO	Milk Group	Study Arm	Postmenstrual Age (weeks)	N	min *	max	mean	sd	median *	Quartile 1 (25%)	Quartile 3 (75%)
2'FL	G2	PRE-TERM	35	5	6.500	26.23	10.45	8.824	6.500	6.500	6.500
2'FL	G2	PRE-TERM	36	5	6.500	22.44	9.689	7.131	6.500	6.500	6.500
2'FL	G2	PRE-TERM	37	4	6.500	6.500	6.500	0.0000	6.500	6.500	6.500
2'FL	G2	PRE-TERM	38	4	6.500	20.67	10.04	7.083	6.500	6.500	10.04
2'FL	G2	PRE-TERM	39	3	6.500	6.500	6.500	0.0000	6.500	6.500	6.500
2'FL	G2	PRE-TERM	40	3	6.500	18.86	10.62	7.137	6.500	6.500	12.68
2'FL	G2	PRE-TERM	41	2	6.500	6.500	6.500	0.0000	6.500	6.500	6.500
2'FL	G2	PRE-TERM	42	3	6.500	16.58	9.861	5.821	6.500	6.500	11.54
2'FL	G2	PRE-TERM	43	2	6.500	6.500	6.500	0.0000	6.500	6.500	6.500
2'FL	G2	PRE-TERM	44	3	6.500	15.22	9.406	5.034	6.500	6.500	10.86
2'FL	G2	PRE-TERM	45	2	6.500	6.500	6.500	0.0000	6.500	6.500	6.500
2'FL	G2	PRE-TERM	46	2	6.500	6.500	6.500	0.0000	6.500	6.500	6.500
2'FL	G2	PRE-TERM	48	1	6.500	6.500	6.500	NA	6.500	6.500	6.500
2'FL	G2	TERM	39	1	6.500	6.500	6.500	NA	6.500	6.500	6.500
2'FL	G2	TERM	40	5	6.500	25.68	10.34	8.578	6.500	6.500	6.500
2'FL	G2	TERM	41	4	6.500	6.500	6.500	0.0000	6.500	6.500	6.500
2'FL	G2	TERM	42	6	6.500	6.500	6.500	0.0000	6.500	6.500	6.500
2'FL	G2	TERM	43	5	6.500	14.11	8.023	3.405	6.500	6.500	6.500
2'FL	G2	TERM	44	5	6.500	6.500	6.500	0.0000	6.500	6.500	6.500
2'FL	G2	TERM	45	5	6.500	6.500	6.500	0.0000	6.500	6.500	6.500
2'FL	G2	TERM	46	5	6.500	6.500	6.500	0.0000	6.500	6.500	6.500
2'FL	G2	TERM	47	4	6.500	6.500	6.500	0.0000	6.500	6.500	6.500
2'FL	G3	PRE-TERM	30	1	3314	3314	3314	NA	3314	3314	3314
2'FL	G3	PRE-TERM	31	1	3465	3465	3465	NA	3465	3465	3465
2'FL	G3	PRE-TERM	32	1	2900	2900	2900	NA	2900	2900	2900
2'FL	G3	PRE-TERM	33	1	2645	2645	2645	NA	2645	2645	2645
2'FL	G3	PRE-TERM	34	1	3001	3001	3001	NA	3001	3001	3001
2'FL	G3	PRE-TERM	35	1	3071	3071	3071	NA	3071	3071	3071
2'FL	G3	PRE-TERM	36	1	2571	2571	2571	NA	2571	2571	2571
2'FL	G3	PRE-TERM	37	1	2316	2316	2316	NA	2316	2316	2316
2'FL	G3	PRE-TERM	39	1	2139	2139	2139	NA	2139	2139	2139
2'FL	G3	PRE-TERM	41	1	2079	2079	2079	NA	2079	2079	2079
2'FL	G3	PRE-TERM	43	1	2189	2189	2189	NA	2189	2189	2189
2'FL	G3	PRE-TERM	45	1	2024	2024	2024	NA	2024	2024	2024
2'FL	G3	TERM	41	1	6076	6076	6076	NA	6076	6076	6076
2'FL	G3	TERM	42	1	5572	5572	5572	NA	5572	5572	5572

Table S4 Concentration of Human Milk Oligosaccharides in Term or Preterm Milk At Specified Postmenstrual Age Separated By Milk Group

** When there are results below the method limit of quanfification (LoQ) the result has been assigned value of 0.5 × LoQ, hence the minimum value appears to be the same in many cases. When a large number of datapoints are below LoQ this can also have the effect that the median = minimum.*

					HMO Concentration (mg/L)						
HMO	Milk Group	Study Arm	Postmenstrual Age (weeks)	N	min *	max	mean	sd	median *	Quartile 1 (25%)	Quartile 3 (75%)
2'FL	G3	TERM	43	1	4390	4390	4390	NA	4390	4390	4390
2'FL	G3	TERM	44	1	4556	4556	4556	NA	4556	4556	4556
2'FL	G3	TERM	45	1	4090	4090	4090	NA	4090	4090	4090
2'FL	G3	TERM	46	1	3666	3666	3666	NA	3666	3666	3666
2'FL	G3	TERM	47	1	3530	3530	3530	NA	3530	3530	3530
2'FL	G3	TERM	48	1	4133	4133	4133	NA	4133	4133	4133
2'FL	G4	TERM	40	1	26.81	26.81	26.81	NA	26.81	26.81	26.81
2'FL	G4	TERM	41	1	16.16	16.16	16.16	NA	16.16	16.16	16.16
2'FL	G4	TERM	42	1	6.500	6.500	6.500	NA	6.500	6.500	6.500
2'FL	G4	TERM	43	1	6.500	6.500	6.500	NA	6.500	6.500	6.500
2'FL	G4	TERM	44	1	6.500	6.500	6.500	NA	6.500	6.500	6.500
2'FL	G4	TERM	45	1	6.500	6.500	6.500	NA	6.500	6.500	6.500
2'FL	G4	TERM	46	1	6.500	6.500	6.500	NA	6.500	6.500	6.500
2'FL	G4	TERM	47	1	6.500	6.500	6.500	NA	6.500	6.500	6.500
3'GL	G1	PRE-TERM	30	7	4.000	10.44	6.177	2.804	4.000	4.000	8.401
3'GL	G1	PRE-TERM	31	9	4.000	14.88	5.722	3.758	4.000	4.000	4.000
3'GL	G1	PRE-TERM	32	13	4.000	28.16	8.027	6.917	4.000	4.000	10.38
3'GL	G1	PRE-TERM	33	18	4.000	28.82	7.853	7.644	4.000	4.000	7.820
3'GL	G1	PRE-TERM	34	19	4.000	30.06	7.747	7.610	4.000	4.000	6.872
3'GL	G1	PRE-TERM	35	18	4.000	21.56	6.275	4.948	4.000	4.000	4.000
3'GL	G1	PRE-TERM	36	19	4.000	10.04	4.808	1.948	4.000	4.000	4.000
3'GL	G1	PRE-TERM	37	18	4.000	11.85	4.975	2.322	4.000	4.000	4.000
3'GL	G1	PRE-TERM	38	11	4.000	70.00	13.51	19.64	4.000	4.000	11.84
3'GL	G1	PRE-TERM	39	16	4.000	23.04	6.686	5.546	4.000	4.000	5.030
3'GL	G1	PRE-TERM	40	7	4.000	21.24	10.85	7.181	11.12	4.000	15.78
3'GL	G1	PRE-TERM	41	12	4.000	15.76	6.675	4.865	4.000	4.000	6.359
3'GL	G1	PRE-TERM	42	6	4.000	21.49	9.513	7.063	7.152	4.000	12.54
3'GL	G1	PRE-TERM	43	10	4.000	15.01	5.814	3.932	4.000	4.000	4.000
3'GL	G1	PRE-TERM	44	5	4.000	10.64	6.305	3.216	4.000	4.000	8.889
3'GL	G1	PRE-TERM	45	9	4.000	8.221	4.469	1.407	4.000	4.000	4.000
3'GL	G1	PRE-TERM	46	6	4.000	15.21	7.703	4.570	6.523	4.000	9.728
3'GL	G1	PRE-TERM	47	3	4.000	4.000	4.000	0.0000	4.000	4.000	4.000
3'GL	G1	PRE-TERM	48	4	4.000	12.84	6.211	4.422	4.000	4.000	6.211
3'GL	G1	TERM	38	2	8.823	9.726	9.274	0.6384	9.274	9.049	9.500
3'GL	G1	TERM	39	9	4.000	19.87	10.29	6.037	9.017	4.000	16.04
3'GL	G1	TERM	40	13	4.000	20.76	10.12	5.773	11.16	4.000	13.96

Table S4 Concentration of Human Milk Oligosaccharides in Term or Preterm Milk At Specified Postmenstrual Age Separated By Milk Group

** When there are results below the method limit of quanfification (LoQ) the result has been assigned value of 0.5 × LoQ, hence the minimum value appears to be the same in many cases. When a large number of datapoints are below LoQ this can also have the effect that the median = minimum.*

					HMO Concentration (mg/L)						
HMO	Milk Group	Study Arm	Postmenstrual Age (weeks)	N	min *	max	mean	sd	median *	Quartile 1 (25%)	Quartile 3 (75%)
3'GL	G1	TERM	41	21	4.000	19.07	8.251	4.602	8.173	4.000	11.70
3'GL	G1	TERM	42	21	4.000	17.67	5.629	3.488	4.000	4.000	4.000
3'GL	G1	TERM	43	21	4.000	16.81	6.408	3.811	4.000	4.000	9.229
3'GL	G1	TERM	44	20	4.000	18.94	6.530	4.803	4.000	4.000	5.443
3'GL	G1	TERM	45	21	4.000	33.42	5.916	6.729	4.000	4.000	4.000
3'GL	G1	TERM	46	17	4.000	10.07	4.654	1.854	4.000	4.000	4.000
3'GL	G1	TERM	47	12	4.000	16.11	5.386	3.619	4.000	4.000	4.000
3'GL	G1	TERM	48	6	4.000	20.73	6.789	6.832	4.000	4.000	4.000
3'GL	G2	PRE-TERM	29	1	11.32	11.32	11.32	NA	11.32	11.32	11.32
3'GL	G2	PRE-TERM	30	2	4.000	8.204	6.102	2.973	6.102	5.051	7.153
3'GL	G2	PRE-TERM	31	2	4.000	9.894	6.947	4.167	6.947	5.473	8.420
3'GL	G2	PRE-TERM	32	3	4.000	12.56	6.853	4.942	4.000	4.000	8.279
3'GL	G2	PRE-TERM	33	5	4.000	21.02	10.20	8.556	4.000	4.000	17.98
3'GL	G2	PRE-TERM	34	5	4.000	11.13	6.606	3.595	4.000	4.000	9.895
3'GL	G2	PRE-TERM	35	5	4.000	4.000	4.000	0.0000	4.000	4.000	4.000
3'GL	G2	PRE-TERM	36	5	4.000	8.011	4.802	1.794	4.000	4.000	4.000
3'GL	G2	PRE-TERM	37	4	4.000	16.72	8.459	6.012	6.559	4.000	11.02
3'GL	G2	PRE-TERM	38	4	4.000	12.18	6.046	4.092	4.000	4.000	6.046
3'GL	G2	PRE-TERM	39	3	4.000	4.000	4.000	0.0000	4.000	4.000	4.000
3'GL	G2	PRE-TERM	40	3	4.000	4.000	4.000	0.0000	4.000	4.000	4.000
3'GL	G2	PRE-TERM	41	2	4.000	4.000	4.000	0.0000	4.000	4.000	4.000
3'GL	G2	PRE-TERM	42	3	4.000	11.78	6.593	4.492	4.000	4.000	7.890
3'GL	G2	PRE-TERM	43	2	4.000	4.000	4.000	0.0000	4.000	4.000	4.000
3'GL	G2	PRE-TERM	44	3	4.000	4.000	4.000	0.0000	4.000	4.000	4.000
3'GL	G2	PRE-TERM	45	2	4.000	10.27	7.133	4.431	7.133	5.567	8.700
3'GL	G2	PRE-TERM	46	2	4.000	12.65	8.325	6.117	8.325	6.163	10.49
3'GL	G2	PRE-TERM	48	1	4.000	4.000	4.000	NA	4.000	4.000	4.000
3'GL	G2	TERM	39	1	14.96	14.96	14.96	NA	14.96	14.96	14.96
3'GL	G2	TERM	40	5	4.000	21.33	13.37	7.088	12.76	9.509	19.25
3'GL	G2	TERM	41	4	4.000	10.91	5.728	3.456	4.000	4.000	5.728
3'GL	G2	TERM	42	6	4.000	8.782	4.797	1.952	4.000	4.000	4.000
3'GL	G2	TERM	43	5	4.000	4.000	4.000	0.0000	4.000	4.000	4.000
3'GL	G2	TERM	44	5	4.000	4.000	4.000	0.0000	4.000	4.000	4.000
3'GL	G2	TERM	45	5	4.000	4.000	4.000	0.0000	4.000	4.000	4.000
3'GL	G2	TERM	46	5	4.000	4.000	4.000	0.0000	4.000	4.000	4.000
3'GL	G2	TERM	47	4	4.000	4.000	4.000	0.0000	4.000	4.000	4.000

Table S4 Concentration of Human Milk Oligosaccharides in Term or Preterm Milk At Specified Postmenstrual Age Separated By Milk Group

** When there are results below the method limit of quanfification (LoQ) the result has been assigned value of 0.5 × LoQ, hence the minimum value appears to be the same in many cases. When a large number of datapoints are below LoQ this can also have the effect that the median = minimum.*

HMO	Milk Group	Study Arm	Postmenstrual Age (weeks)	N	HMO Concentration (mg/L) min *	max	mean	sd	median *	Quartile 1 (25%)	Quartile 3 (75%)
3'GL	G3	PRE-TERM	30	1	4.000	4.000	4.000	NA	4.000	4.000	4.000
3'GL	G3	PRE-TERM	31	1	4.000	4.000	4.000	NA	4.000	4.000	4.000
3'GL	G3	PRE-TERM	32	1	4.000	4.000	4.000	NA	4.000	4.000	4.000
3'GL	G3	PRE-TERM	33	1	4.000	4.000	4.000	NA	4.000	4.000	4.000
3'GL	G3	PRE-TERM	34	1	4.000	4.000	4.000	NA	4.000	4.000	4.000
3'GL	G3	PRE-TERM	35	1	4.000	4.000	4.000	NA	4.000	4.000	4.000
3'GL	G3	PRE-TERM	36	1	4.000	4.000	4.000	NA	4.000	4.000	4.000
3'GL	G3	PRE-TERM	37	1	4.000	4.000	4.000	NA	4.000	4.000	4.000
3'GL	G3	PRE-TERM	39	1	4.000	4.000	4.000	NA	4.000	4.000	4.000
3'GL	G3	PRE-TERM	41	1	4.000	4.000	4.000	NA	4.000	4.000	4.000
3'GL	G3	PRE-TERM	43	1	4.000	4.000	4.000	NA	4.000	4.000	4.000
3'GL	G3	PRE-TERM	45	1	4.000	4.000	4.000	NA	4.000	4.000	4.000
3'GL	G3	TERM	41	1	8.864	8.864	8.864	NA	8.864	8.864	8.864
3'GL	G3	TERM	42	1	4.000	4.000	4.000	NA	4.000	4.000	4.000
3'GL	G3	TERM	43	1	4.000	4.000	4.000	NA	4.000	4.000	4.000
3'GL	G3	TERM	44	1	4.000	4.000	4.000	NA	4.000	4.000	4.000
3'GL	G3	TERM	45	1	4.000	4.000	4.000	NA	4.000	4.000	4.000
3'GL	G3	TERM	46	1	4.000	4.000	4.000	NA	4.000	4.000	4.000
3'GL	G3	TERM	47	1	4.000	4.000	4.000	NA	4.000	4.000	4.000
3'GL	G3	TERM	48	1	4.000	4.000	4.000	NA	4.000	4.000	4.000
3'GL	G4	TERM	40	1	32.97	32.97	32.97	NA	32.97	32.97	32.97
3'GL	G4	TERM	41	1	19.72	19.72	19.72	NA	19.72	19.72	19.72
3'GL	G4	TERM	42	1	10.00	10.00	10.00	NA	10.00	10.00	10.00
3'GL	G4	TERM	43	1	8.499	8.499	8.499	NA	8.499	8.499	8.499
3'GL	G4	TERM	44	1	4.000	4.000	4.000	NA	4.000	4.000	4.000
3'GL	G4	TERM	45	1	8.446	8.446	8.446	NA	8.446	8.446	8.446
3'GL	G4	TERM	46	1	4.000	4.000	4.000	NA	4.000	4.000	4.000
3'GL	G4	TERM	47	1	4.000	4.000	4.000	NA	4.000	4.000	4.000
3'SL	G1	PRE-TERM	30	7	175.9	365.6	236.7	62.41	224.6	203.6	241.7
3'SL	G1	PRE-TERM	31	9	126.6	329.8	220.9	54.89	220.0	198.8	241.0
3'SL	G1	PRE-TERM	32	13	107.7	260.5	201.7	47.75	202.4	184.5	241.6
3'SL	G1	PRE-TERM	33	18	113.1	513.3	198.7	92.80	178.5	132.0	222.8
3'SL	G1	PRE-TERM	34	19	107.6	439.9	199.3	82.89	169.5	150.4	236.1
3'SL	G1	PRE-TERM	35	18	98.75	430.3	194.3	85.34	171.8	160.5	198.4
3'SL	G1	PRE-TERM	36	19	103.5	286.8	173.7	48.94	177.0	144.6	199.0
3'SL	G1	PRE-TERM	37	18	85.96	303.5	176.5	55.56	173.5	156.3	212.2

Table S4 Concentration of Human Milk Oligosaccharides in Term or Preterm Milk At Specified Postmenstrual Age Separated By Milk Group

** When there are results below the method limit of quanfification (LoQ) the result has been assigned value of 0.5 × LoQ, hence the minimum value appears to be the same in many cases. When a large number of datapoints are below LoQ this can also have the effect that the median = minimum.*

					HMO Concentration (mg/L)						
**HMO**	**Milk Group**	**Study Arm**	**Postmenstrual Age (weeks)**	**N**	**min ***	**max**	**mean**	**sd**	**median ***	**Quartile 1 (25%)**	**Quartile 3 (75%)**
3'SL	G1	PRE-TERM	38	11	91.34	438.1	202.3	100.6	219.4	124.3	239.1
3'SL	G1	PRE-TERM	39	16	91.08	329.8	178.1	66.15	160.6	141.1	215.8
3'SL	G1	PRE-TERM	40	7	99.72	333.8	199.1	86.22	185.0	136.0	251.4
3'SL	G1	PRE-TERM	41	12	99.07	393.0	223.2	96.61	176.6	167.4	298.0
3'SL	G1	PRE-TERM	42	6	88.38	274.9	163.2	73.26	149.4	107.2	205.2
3'SL	G1	PRE-TERM	43	10	109.4	403.2	212.1	94.91	170.5	143.2	281.0
3'SL	G1	PRE-TERM	44	5	108.2	248.9	168.2	62.71	143.1	120.4	220.2
3'SL	G1	PRE-TERM	45	9	119.6	325.0	192.4	67.26	165.2	155.6	224.8
3'SL	G1	PRE-TERM	46	6	82.71	282.3	165.7	84.03	141.1	100.8	230.2
3'SL	G1	PRE-TERM	47	3	116.2	264.4	184.9	74.69	174.1	145.1	219.3
3'SL	G1	PRE-TERM	48	4	100.5	319.4	167.9	101.8	125.8	116.1	177.5
3'SL	G1	TERM	38	2	181.9	351.2	266.5	119.7	266.5	224.2	308.9
3'SL	G1	TERM	39	9	119.6	287.2	219.1	66.99	230.8	146.2	279.2
3'SL	G1	TERM	40	13	94.16	354.4	177.4	76.04	140.2	134.9	186.1
3'SL	G1	TERM	41	21	78.49	270.7	152.3	48.41	139.2	122.9	175.6
3'SL	G1	TERM	42	21	86.80	280.3	138.5	43.46	134.0	105.6	163.4
3'SL	G1	TERM	43	21	77.94	282.3	135.3	45.14	121.8	106.4	156.4
3'SL	G1	TERM	44	20	72.18	272.4	130.7	47.71	128.6	91.36	151.7
3'SL	G1	TERM	45	21	74.58	274.9	132.6	44.95	124.1	105.7	151.9
3'SL	G1	TERM	46	17	82.35	303.0	132.9	51.17	114.4	104.8	145.2
3'SL	G1	TERM	47	12	50.25	240.1	130.9	53.79	141.0	87.70	163.0
3'SL	G1	TERM	48	6	94.50	236.8	145.1	52.53	142.1	105.0	157.2
3'SL	G2	PRE-TERM	29	1	419.1	419.1	419.1	NA	419.1	419.1	419.1
3'SL	G2	PRE-TERM	30	2	215.7	257.5	236.6	29.53	236.6	226.2	247.1
3'SL	G2	PRE-TERM	31	2	185.3	196.6	190.9	7.999	190.9	188.1	193.8
3'SL	G2	PRE-TERM	32	3	131.1	272.6	209.0	71.83	223.3	177.2	247.9
3'SL	G2	PRE-TERM	33	5	132.6	284.0	235.5	60.83	254.5	232.2	274.1
3'SL	G2	PRE-TERM	34	5	120.4	311.3	202.4	72.62	191.1	161.1	228.2
3'SL	G2	PRE-TERM	35	5	119.6	240.4	182.2	49.47	178.0	151.7	221.3
3'SL	G2	PRE-TERM	36	5	105.8	362.9	198.3	103.0	164.7	129.5	228.3
3'SL	G2	PRE-TERM	37	4	129.1	342.8	198.0	97.72	160.1	148.0	210.2
3'SL	G2	PRE-TERM	38	4	97.27	202.4	137.7	45.88	125.5	111.0	152.2
3'SL	G2	PRE-TERM	39	3	92.00	186.3	133.6	48.14	122.5	107.2	154.4
3'SL	G2	PRE-TERM	40	3	92.61	143.9	118.6	25.65	119.4	106.0	131.7
3'SL	G2	PRE-TERM	41	2	126.1	212.0	169.1	60.80	169.1	147.6	190.6
3'SL	G2	PRE-TERM	42	3	83.76	162.9	119.7	40.07	112.4	98.11	137.7

Table S4 Concentration of Human Milk Oligosaccharides in Term or Preterm Milk At Specified Postmenstrual Age Separated By Milk Group

** When there are results below the method limit of quanfification (LoQ) the result has been assigned value of 0.5 × LoQ, hence the minimum value appears to be the same in many cases. When a large number of datapoints are below LoQ this can also have the effect that the median = minimum.*

					HMO Concentration (mg/L)						
HMO	Milk Group	Study Arm	Postmenstrual Age (weeks)	N	min *	max	mean	sd	median *	Quartile 1 (25%)	Quartile 3 (75%)
3'SL	G2	PRE-TERM	43	2	132.8	239.2	186.0	75.23	186.0	159.4	212.6
3'SL	G2	PRE-TERM	44	3	86.41	146.4	113.7	30.35	108.4	97.39	127.4
3'SL	G2	PRE-TERM	45	2	86.95	179.1	133.0	65.20	133.0	110.0	156.1
3'SL	G2	PRE-TERM	46	2	146.0	162.4	154.2	11.56	154.2	150.1	158.3
3'SL	G2	PRE-TERM	48	1	145.9	145.9	145.9	NA	145.9	145.9	145.9
3'SL	G2	TERM	39	1	162.7	162.7	162.7	NA	162.7	162.7	162.7
3'SL	G2	TERM	40	5	100.4	380.6	202.6	114.4	197.0	105.2	229.6
3'SL	G2	TERM	41	4	89.66	160.1	117.7	32.28	110.5	94.20	134.0
3'SL	G2	TERM	42	6	94.04	136.4	118.6	18.31	122.6	104.1	134.0
3'SL	G2	TERM	43	5	92.27	136.6	114.8	20.12	116.6	96.47	132.1
3'SL	G2	TERM	44	5	93.14	140.4	112.9	19.55	103.5	101.7	126.0
3'SL	G2	TERM	45	5	35.33	163.0	106.3	48.44	111.0	87.95	134.1
3'SL	G2	TERM	46	5	74.78	122.0	99.10	18.01	102.7	88.86	107.1
3'SL	G2	TERM	47	4	77.15	131.7	107.1	24.72	109.9	92.26	124.8
3'SL	G3	PRE-TERM	30	1	174.7	174.7	174.7	NA	174.7	174.7	174.7
3'SL	G3	PRE-TERM	31	1	218.9	218.9	218.9	NA	218.9	218.9	218.9
3'SL	G3	PRE-TERM	32	1	209.5	209.5	209.5	NA	209.5	209.5	209.5
3'SL	G3	PRE-TERM	33	1	155.9	155.9	155.9	NA	155.9	155.9	155.9
3'SL	G3	PRE-TERM	34	1	177.6	177.6	177.6	NA	177.6	177.6	177.6
3'SL	G3	PRE-TERM	35	1	176.5	176.5	176.5	NA	176.5	176.5	176.5
3'SL	G3	PRE-TERM	36	1	124.4	124.4	124.4	NA	124.4	124.4	124.4
3'SL	G3	PRE-TERM	37	1	132.0	132.0	132.0	NA	132.0	132.0	132.0
3'SL	G3	PRE-TERM	39	1	117.7	117.7	117.7	NA	117.7	117.7	117.7
3'SL	G3	PRE-TERM	41	1	123.5	123.5	123.5	NA	123.5	123.5	123.5
3'SL	G3	PRE-TERM	43	1	128.8	128.8	128.8	NA	128.8	128.8	128.8
3'SL	G3	PRE-TERM	45	1	131.0	131.0	131.0	NA	131.0	131.0	131.0
3'SL	G3	TERM	41	1	154.8	154.8	154.8	NA	154.8	154.8	154.8
3'SL	G3	TERM	42	1	152.1	152.1	152.1	NA	152.1	152.1	152.1
3'SL	G3	TERM	43	1	149.3	149.3	149.3	NA	149.3	149.3	149.3
3'SL	G3	TERM	44	1	153.6	153.6	153.6	NA	153.6	153.6	153.6
3'SL	G3	TERM	45	1	140.2	140.2	140.2	NA	140.2	140.2	140.2
3'SL	G3	TERM	46	1	135.1	135.1	135.1	NA	135.1	135.1	135.1
3'SL	G3	TERM	47	1	138.1	138.1	138.1	NA	138.1	138.1	138.1
3'SL	G3	TERM	48	1	140.2	140.2	140.2	NA	140.2	140.2	140.2
3'SL	G4	TERM	40	1	241.3	241.3	241.3	NA	241.3	241.3	241.3
3'SL	G4	TERM	41	1	206.5	206.5	206.5	NA	206.5	206.5	206.5

Table S4 Concentration of Human Milk Oligosaccharides in Term or Preterm Milk At Specified Postmenstrual Age Separated By Milk Group

** When there are results below the method limit of quanfification (LoQ) the result has been assigned value of 0.5 × LoQ, hence the minimum value appears to be the same in many cases. When a large number of datapoints are below LoQ this can also have the effect that the median = minimum.*

HMO	Milk Group	Study Arm	Postmenstrual Age (weeks)	N	HMO Concentration (mg/L) min *	max	mean	sd	median *	Quartile 1 (25%)	Quartile 3 (75%)
3'SL	G4	TERM	42	1	162.2	162.2	162.2	NA	162.2	162.2	162.2
3'SL	G4	TERM	43	1	155.5	155.5	155.5	NA	155.5	155.5	155.5
3'SL	G4	TERM	44	1	140.1	140.1	140.1	NA	140.1	140.1	140.1
3'SL	G4	TERM	45	1	152.2	152.2	152.2	NA	152.2	152.2	152.2
3'SL	G4	TERM	46	1	119.8	119.8	119.8	NA	119.8	119.8	119.8
3'SL	G4	TERM	47	1	111.7	111.7	111.7	NA	111.7	111.7	111.7
3FL	G1	PRE-TERM	30	7	183.6	844.2	358.7	236.7	239.7	210.5	411.2
3FL	G1	PRE-TERM	31	9	181.4	978.0	368.0	256.5	259.0	213.0	381.1
3FL	G1	PRE-TERM	32	13	112.2	1087	362.5	259.7	271.8	186.2	395.4
3FL	G1	PRE-TERM	33	18	154.5	1262	384.6	248.9	321.4	260.7	420.9
3FL	G1	PRE-TERM	34	19	179.6	1264	436.0	250.1	394.1	274.0	507.2
3FL	G1	PRE-TERM	35	18	205.2	1201	495.3	214.4	473.7	433.2	530.2
3FL	G1	PRE-TERM	36	19	228.4	1242	515.2	229.1	495.4	385.8	576.9
3FL	G1	PRE-TERM	37	18	266.2	1356	604.9	269.4	530.1	423.4	654.3
3FL	G1	PRE-TERM	38	11	75.94	1080	630.6	292.9	661.1	535.2	761.0
3FL	G1	PRE-TERM	39	16	270.0	1550	679.7	294.1	655.5	525.3	793.0
3FL	G1	PRE-TERM	40	7	487.8	809.4	648.6	118.5	682.9	552.3	727.7
3FL	G1	PRE-TERM	41	12	368.4	1504	779.0	282.7	761.5	634.8	840.5
3FL	G1	PRE-TERM	42	6	492.5	931.8	723.1	189.0	734.7	568.3	882.6
3FL	G1	PRE-TERM	43	10	446.0	1914	840.4	418.2	762.5	610.7	858.4
3FL	G1	PRE-TERM	44	5	546.0	1253	864.7	285.3	772.2	696.4	1056
3FL	G1	PRE-TERM	45	9	435.5	2188	933.6	529.4	740.6	611.9	1091
3FL	G1	PRE-TERM	46	6	486.6	1250	941.9	318.2	1032	713.2	1191
3FL	G1	PRE-TERM	47	3	745.8	1094	927.1	174.7	941.1	843.5	1018
3FL	G1	PRE-TERM	48	4	688.4	1378	1053	286.6	1073	932.0	1194
3FL	G1	TERM	38	2	260.1	341.2	300.7	57.39	300.7	280.4	321.0
3FL	G1	TERM	39	9	137.2	447.8	263.1	128.8	184.1	173.2	412.7
3FL	G1	TERM	40	13	145.3	662.5	299.7	152.9	255.6	190.3	359.4
3FL	G1	TERM	41	21	119.4	706.0	294.4	161.4	253.7	178.3	388.9
3FL	G1	TERM	42	21	159.7	672.5	343.7	152.2	314.9	223.3	414.1
3FL	G1	TERM	43	21	197.3	921.0	436.9	204.6	392.4	271.5	465.1
3FL	G1	TERM	44	20	213.6	863.1	468.0	176.9	446.8	363.6	534.5
3FL	G1	TERM	45	21	294.2	1114	542.5	215.4	496.2	424.4	572.6
3FL	G1	TERM	46	17	151.0	1231	589.9	288.9	527.8	447.2	608.8
3FL	G1	TERM	47	12	242.1	1182	557.2	277.9	525.9	403.3	595.5
3FL	G1	TERM	48	6	360.8	721.9	521.5	153.6	490.8	399.1	644.9

Table S4 Concentration of Human Milk Oligosaccharides in Term or Preterm Milk At Specified Postmenstrual Age Separated By Milk Group

** When there are results below the method limit of quanfification (LoQ) the result has been assigned value of 0.5 × LoQ, hence the minimum value appears to be the same in many cases. When a large number of datapoints are below LoQ this can also have the effect that the median = minimum.*

					HMO Concentration (mg/L)						
HMO	Milk Group	Study Arm	Postmenstrual Age (weeks)	N	min *	max	mean	sd	median *	Quartile 1 (25%)	Quartile 3 (75%)
3FL	G2	PRE-TERM	29	1	1472	1472	1472	NA	1472	1472	1472
3FL	G2	PRE-TERM	30	2	991.9	1176	1084	130.2	1084	1038	1130
3FL	G2	PRE-TERM	31	2	1041	1124	1083	59.20	1083	1062	1104
3FL	G2	PRE-TERM	32	3	836.1	1137	1017	159.5	1078	957.1	1108
3FL	G2	PRE-TERM	33	5	856.8	1457	1097	250.6	1019	908.2	1246
3FL	G2	PRE-TERM	34	5	841.4	1549	1195	274.6	1165	1050	1367
3FL	G2	PRE-TERM	35	5	985.7	1488	1231	214.7	1287	1034	1359
3FL	G2	PRE-TERM	36	5	667.5	1787	1338	516.8	1649	894.6	1690
3FL	G2	PRE-TERM	37	4	829.2	2465	1810	739.0	1974	1454	2330
3FL	G2	PRE-TERM	38	4	958.9	1661	1224	333.1	1138	967.0	1396
3FL	G2	PRE-TERM	39	3	829.5	1825	1280	504.6	1186	1008	1506
3FL	G2	PRE-TERM	40	3	956.4	2231	1401	719.5	1015	985.5	1623
3FL	G2	PRE-TERM	41	2	2011	2126	2069	81.13	2069	2040	2097
3FL	G2	PRE-TERM	42	3	1158	2821	1724	950.0	1193	1175	2007
3FL	G2	PRE-TERM	43	2	2855	3101	2978	174.1	2978	2916	3039
3FL	G2	PRE-TERM	44	3	1202	2151	1522	544.8	1213	1208	1682
3FL	G2	PRE-TERM	45	2	1342	2185	1764	596.2	1764	1553	1975
3FL	G2	PRE-TERM	46	2	2077	3398	2738	933.6	2738	2407	3068
3FL	G2	PRE-TERM	48	1	2505	2505	2505	NA	2505	2505	2505
3FL	G2	TERM	39	1	1142	1142	1142	NA	1142	1142	1142
3FL	G2	TERM	40	5	768.8	1245	1016	229.5	1025	800.0	1240
3FL	G2	TERM	41	4	632.0	1577	1194	404.3	1284	1063	1415
3FL	G2	TERM	42	6	780.7	1861	1262	352.8	1268	1131	1297
3FL	G2	TERM	43	5	782.1	1675	1308	377.7	1381	1076	1625
3FL	G2	TERM	44	5	799.4	1848	1425	464.2	1696	1062	1720
3FL	G2	TERM	45	5	624.3	1983	1313	592.4	1265	858.8	1834
3FL	G2	TERM	46	5	686.5	1978	1451	537.9	1460	1198	1931
3FL	G2	TERM	47	4	727.3	2013	1384	552.8	1398	1072	1710
3FL	G3	PRE-TERM	30	1	64.12	64.12	64.12	NA	64.12	64.12	64.12
3FL	G3	PRE-TERM	31	1	148.3	148.3	148.3	NA	148.3	148.3	148.3
3FL	G3	PRE-TERM	32	1	181.0	181.0	181.0	NA	181.0	181.0	181.0
3FL	G3	PRE-TERM	33	1	158.0	158.0	158.0	NA	158.0	158.0	158.0
3FL	G3	PRE-TERM	34	1	215.6	215.6	215.6	NA	215.6	215.6	215.6
3FL	G3	PRE-TERM	35	1	223.4	223.4	223.4	NA	223.4	223.4	223.4
3FL	G3	PRE-TERM	36	1	254.0	254.0	254.0	NA	254.0	254.0	254.0
3FL	G3	PRE-TERM	37	1	224.9	224.9	224.9	NA	224.9	224.9	224.9

Table S4 Concentration of Human Milk Oligosaccharides in Term or Preterm Milk At Specified Postmenstrual Age Separated By Milk Group

** When there are results below the method limit of quanfification (LoQ) the result has been assigned value of 0.5 × LoQ, hence the minimum value appears to be the same in many cases. When a large number of datapoints are below LoQ this can also have the effect that the median = minimum.*

					HMO Concentration (mg/L)						
HMO	Milk Group	Study Arm	Postmenstrual Age (weeks)	N	min *	max	mean	sd	median *	Quartile 1 (25%)	Quartile 3 (75%)
3FL	G3	PRE-TERM	39	1	241.8	241.8	241.8	NA	241.8	241.8	241.8
3FL	G3	PRE-TERM	41	1	278.6	278.6	278.6	NA	278.6	278.6	278.6
3FL	G3	PRE-TERM	43	1	298.6	298.6	298.6	NA	298.6	298.6	298.6
3FL	G3	PRE-TERM	45	1	325.1	325.1	325.1	NA	325.1	325.1	325.1
3FL	G3	TERM	41	1	39.33	39.33	39.33	NA	39.33	39.33	39.33
3FL	G3	TERM	42	1	66.65	66.65	66.65	NA	66.65	66.65	66.65
3FL	G3	TERM	43	1	82.63	82.63	82.63	NA	82.63	82.63	82.63
3FL	G3	TERM	44	1	99.24	99.24	99.24	NA	99.24	99.24	99.24
3FL	G3	TERM	45	1	95.34	95.34	95.34	NA	95.34	95.34	95.34
3FL	G3	TERM	46	1	102.9	102.9	102.9	NA	102.9	102.9	102.9
3FL	G3	TERM	47	1	118.2	118.2	118.2	NA	118.2	118.2	118.2
3FL	G3	TERM	48	1	131.6	131.6	131.6	NA	131.6	131.6	131.6
3FL	G4	TERM	40	1	224.5	224.5	224.5	NA	224.5	224.5	224.5
3FL	G4	TERM	41	1	358.0	358.0	358.0	NA	358.0	358.0	358.0
3FL	G4	TERM	42	1	350.3	350.3	350.3	NA	350.3	350.3	350.3
3FL	G4	TERM	43	1	361.8	361.8	361.8	NA	361.8	361.8	361.8
3FL	G4	TERM	44	1	390.6	390.6	390.6	NA	390.6	390.6	390.6
3FL	G4	TERM	45	1	538.7	538.7	538.7	NA	538.7	538.7	538.7
3FL	G4	TERM	46	1	439.5	439.5	439.5	NA	439.5	439.5	439.5
3FL	G4	TERM	47	1	485.5	485.5	485.5	NA	485.5	485.5	485.5
6'GL	G1	PRE-TERM	30	7	40.18	156.4	95.39	41.13	95.55	67.93	119.9
6'GL	G1	PRE-TERM	31	9	25.16	109.6	58.70	28.24	51.04	40.93	76.93
6'GL	G1	PRE-TERM	32	13	16.66	106.1	57.98	30.65	51.57	30.70	77.72
6'GL	G1	PRE-TERM	33	18	19.99	137.9	50.99	29.64	43.60	33.96	56.07
6'GL	G1	PRE-TERM	34	19	16.16	75.81	36.56	15.02	34.26	26.64	46.22
6'GL	G1	PRE-TERM	35	18	11.89	55.98	30.12	11.87	26.07	23.39	40.08
6'GL	G1	PRE-TERM	36	19	11.33	48.53	26.38	9.589	24.46	19.99	32.16
6'GL	G1	PRE-TERM	37	18	7.746	43.71	24.33	9.809	23.04	17.33	30.44
6'GL	G1	PRE-TERM	38	11	15.17	179.6	41.77	47.49	27.80	19.14	35.84
6'GL	G1	PRE-TERM	39	16	10.72	33.74	18.97	6.759	17.51	13.99	23.49
6'GL	G1	PRE-TERM	40	7	13.55	48.40	25.94	12.48	21.20	18.91	30.29
6'GL	G1	PRE-TERM	41	12	10.40	35.95	21.52	8.511	19.06	15.78	27.27
6'GL	G1	PRE-TERM	42	6	9.255	44.84	22.65	12.03	20.13	17.42	23.79
6'GL	G1	PRE-TERM	43	10	8.465	32.86	18.61	7.272	16.36	13.92	22.48
6'GL	G1	PRE-TERM	44	5	6.997	52.92	24.64	17.49	21.20	14.93	27.14
6'GL	G1	PRE-TERM	45	9	8.310	28.68	15.07	6.759	11.67	11.12	20.50

Table S4 Concentration of Human Milk Oligosaccharides in Term or Preterm Milk At Specified Postmenstrual Age Separated By Milk Group

** When there are results below the method limit of quanfification (LoQ) the result has been assigned value of 0.5 × LoQ, hence the minimum value appears to be the same in many cases. When a large number of datapoints are below LoQ this can also have the effect that the median = minimum.*

					HMO Concentration (mg/L)						
HMO	Milk Group	Study Arm	Postmenstrual Age (weeks)	N	min *	max	mean	sd	median *	Quartile 1 (25%)	Quartile 3 (75%)
6'GL	G1	PRE-TERM	46	6	8.740	46.05	20.20	13.52	15.65	12.87	21.29
6'GL	G1	PRE-TERM	47	3	11.73	21.16	15.14	5.229	12.52	12.12	16.84
6'GL	G1	PRE-TERM	48	4	3.150	15.94	11.41	5.789	13.28	9.581	15.11
6'GL	G1	TERM	38	2	108.2	128.5	118.3	14.31	118.3	113.3	123.4
6'GL	G1	TERM	39	9	38.11	232.6	105.3	68.50	105.7	53.98	110.1
6'GL	G1	TERM	40	13	12.64	234.0	86.63	58.64	77.51	46.00	98.12
6'GL	G1	TERM	41	21	8.541	170.4	62.53	42.65	53.13	33.23	81.07
6'GL	G1	TERM	42	21	7.969	69.86	35.62	15.18	36.24	26.92	42.90
6'GL	G1	TERM	43	21	6.639	59.96	29.80	12.34	31.70	20.87	36.44
6'GL	G1	TERM	44	20	3.150	47.44	23.98	10.78	24.05	16.53	29.59
6'GL	G1	TERM	45	21	3.150	68.27	21.60	14.03	19.23	13.72	27.35
6'GL	G1	TERM	46	17	3.150	40.84	19.69	9.410	16.25	15.18	25.94
6'GL	G1	TERM	47	12	8.807	47.47	19.04	10.83	14.76	12.37	23.35
6'GL	G1	TERM	48	6	9.963	40.02	18.55	11.45	13.20	12.08	20.74
6'GL	G2	PRE-TERM	29	1	54.12	54.12	54.12	NA	54.12	54.12	54.12
6'GL	G2	PRE-TERM	30	2	29.26	39.10	34.18	6.961	34.18	31.72	36.64
6'GL	G2	PRE-TERM	31	2	17.55	39.49	28.52	15.51	28.52	23.04	34.01
6'GL	G2	PRE-TERM	32	3	16.82	64.95	35.23	25.98	23.91	20.37	44.43
6'GL	G2	PRE-TERM	33	5	18.91	94.25	50.80	32.87	38.87	25.75	76.24
6'GL	G2	PRE-TERM	34	5	21.56	34.93	28.25	5.102	27.06	26.32	31.35
6'GL	G2	PRE-TERM	35	5	3.150	32.89	22.84	11.40	25.95	25.85	26.36
6'GL	G2	PRE-TERM	36	5	17.65	25.00	21.25	3.194	21.92	18.30	23.40
6'GL	G2	PRE-TERM	37	4	23.56	38.77	27.81	7.344	24.46	23.66	28.60
6'GL	G2	PRE-TERM	38	4	12.88	29.68	20.02	7.139	18.75	16.06	22.71
6'GL	G2	PRE-TERM	39	3	9.818	16.62	13.75	3.526	14.82	12.32	15.72
6'GL	G2	PRE-TERM	40	3	12.86	18.51	16.33	3.037	17.62	15.24	18.06
6'GL	G2	PRE-TERM	41	2	14.54	24.69	19.62	7.175	19.62	17.08	22.15
6'GL	G2	PRE-TERM	42	3	13.41	28.95	19.29	8.430	15.52	14.46	22.24
6'GL	G2	PRE-TERM	43	2	19.42	27.62	23.52	5.796	23.52	21.47	25.57
6'GL	G2	PRE-TERM	44	3	10.43	17.17	14.49	3.577	15.88	13.15	16.52
6'GL	G2	PRE-TERM	45	2	19.16	25.56	22.36	4.523	22.36	20.76	23.96
6'GL	G2	PRE-TERM	46	2	13.05	32.99	23.02	14.10	23.02	18.04	28.01
6'GL	G2	PRE-TERM	48	1	14.07	14.07	14.07	NA	14.07	14.07	14.07
6'GL	G2	TERM	39	1	122.7	122.7	122.7	NA	122.7	122.7	122.7
6'GL	G2	TERM	40	5	28.62	175.0	127.9	58.55	134.4	134.2	167.4
6'GL	G2	TERM	41	4	15.76	76.66	48.34	25.07	50.47	39.94	58.86

Table S4 Concentration of Human Milk Oligosaccharides in Term or Preterm Milk At Specified Postmenstrual Age Separated By Milk Group

** When there are results below the method limit of quanfification (LoQ) the result has been assigned value of 0.5 × LoQ, hence the minimum value appears to be the same in many cases. When a large number of datapoints are below LoQ this can also have the effect that the median = minimum.*

					HMO Concentration (mg/L)						
**HMO**	**Milk Group**	**Study Arm**	**Postmenstrual Age (weeks)**	**N**	**min ***	**max**	**mean**	**sd**	**median ***	**Quartile 1 (25%)**	**Quartile 3 (75%)**
6'GL	G2	TERM	42	6	12.07	59.61	37.76	16.70	34.95	33.04	48.16
6'GL	G2	TERM	43	5	3.150	27.99	12.91	13.37	3.150	3.150	27.10
6'GL	G2	TERM	44	5	3.150	39.74	18.63	14.25	19.70	8.078	22.47
6'GL	G2	TERM	45	5	6.478	20.80	12.94	6.455	12.30	6.983	18.16
6'GL	G2	TERM	46	5	6.629	22.00	15.20	5.632	16.67	13.79	16.93
6'GL	G2	TERM	47	4	13.89	20.67	16.26	3.061	15.24	14.39	17.12
6'GL	G3	PRE-TERM	30	1	99.02	99.02	99.02	NA	99.02	99.02	99.02
6'GL	G3	PRE-TERM	31	1	51.62	51.62	51.62	NA	51.62	51.62	51.62
6'GL	G3	PRE-TERM	32	1	34.12	34.12	34.12	NA	34.12	34.12	34.12
6'GL	G3	PRE-TERM	33	1	28.35	28.35	28.35	NA	28.35	28.35	28.35
6'GL	G3	PRE-TERM	34	1	30.35	30.35	30.35	NA	30.35	30.35	30.35
6'GL	G3	PRE-TERM	35	1	30.43	30.43	30.43	NA	30.43	30.43	30.43
6'GL	G3	PRE-TERM	36	1	16.02	16.02	16.02	NA	16.02	16.02	16.02
6'GL	G3	PRE-TERM	37	1	18.65	18.65	18.65	NA	18.65	18.65	18.65
6'GL	G3	PRE-TERM	39	1	14.55	14.55	14.55	NA	14.55	14.55	14.55
6'GL	G3	PRE-TERM	41	1	13.66	13.66	13.66	NA	13.66	13.66	13.66
6'GL	G3	PRE-TERM	43	1	14.69	14.69	14.69	NA	14.69	14.69	14.69
6'GL	G3	PRE-TERM	45	1	12.31	12.31	12.31	NA	12.31	12.31	12.31
6'GL	G3	TERM	41	1	109.9	109.9	109.9	NA	109.9	109.9	109.9
6'GL	G3	TERM	42	1	50.71	50.71	50.71	NA	50.71	50.71	50.71
6'GL	G3	TERM	43	1	38.43	38.43	38.43	NA	38.43	38.43	38.43
6'GL	G3	TERM	44	1	31.73	31.73	31.73	NA	31.73	31.73	31.73
6'GL	G3	TERM	45	1	21.24	21.24	21.24	NA	21.24	21.24	21.24
6'GL	G3	TERM	46	1	16.32	16.32	16.32	NA	16.32	16.32	16.32
6'GL	G3	TERM	47	1	15.03	15.03	15.03	NA	15.03	15.03	15.03
6'GL	G3	TERM	48	1	12.89	12.89	12.89	NA	12.89	12.89	12.89
6'GL	G4	TERM	40	1	176.0	176.0	176.0	NA	176.0	176.0	176.0
6'GL	G4	TERM	41	1	92.67	92.67	92.67	NA	92.67	92.67	92.67
6'GL	G4	TERM	42	1	51.40	51.40	51.40	NA	51.40	51.40	51.40
6'GL	G4	TERM	43	1	39.76	39.76	39.76	NA	39.76	39.76	39.76
6'GL	G4	TERM	44	1	30.64	30.64	30.64	NA	30.64	30.64	30.64
6'GL	G4	TERM	45	1	39.81	39.81	39.81	NA	39.81	39.81	39.81
6'GL	G4	TERM	46	1	24.24	24.24	24.24	NA	24.24	24.24	24.24
6'GL	G4	TERM	47	1	20.55	20.55	20.55	NA	20.55	20.55	20.55
6'SL	G1	PRE-TERM	30	7	166.1	656.8	466.5	200.8	550.1	330.6	615.5
6'SL	G1	PRE-TERM	31	9	63.98	879.6	562.3	263.9	598.1	562.3	706.5

Table S4 Concentration of Human Milk Oligosaccharides in Term or Preterm Milk At Specified Postmenstrual Age Separated By Milk Group

** When there are results below the method limit of quanfification (LoQ) the result has been assigned value of 0.5 × LoQ, hence the minimum value appears to be the same in many cases. When a large number of datapoints are below LoQ this can also have the effect that the median = minimum.*

HMO	Milk Group	Study Arm	Postmenstrual Age (weeks)	N	HMO Concentration (mg/L) min *	max	mean	sd	median *	Quartile 1 (25%)	Quartile 3 (75%)
6'SL	G1	PRE-TERM	32	13	74.88	1132	589.2	270.9	623.3	555.8	742.3
6'SL	G1	PRE-TERM	33	18	61.36	1135	516.0	280.1	522.5	329.0	671.9
6'SL	G1	PRE-TERM	34	19	77.87	926.1	435.5	236.8	424.7	234.3	555.4
6'SL	G1	PRE-TERM	35	18	106.1	695.8	393.3	164.7	432.2	282.1	496.1
6'SL	G1	PRE-TERM	36	19	139.1	565.1	347.9	145.9	376.6	218.1	459.4
6'SL	G1	PRE-TERM	37	18	116.1	476.6	299.1	108.8	314.3	212.0	378.5
6'SL	G1	PRE-TERM	38	11	10.00	394.0	258.2	108.0	284.6	228.5	309.4
6'SL	G1	PRE-TERM	39	16	107.3	383.8	205.4	78.33	188.1	152.5	231.5
6'SL	G1	PRE-TERM	40	7	135.1	401.4	232.2	111.4	158.9	145.5	319.3
6'SL	G1	PRE-TERM	41	12	89.80	199.9	153.3	34.29	156.1	139.1	175.4
6'SL	G1	PRE-TERM	42	6	105.0	271.0	186.4	85.27	186.0	108.1	262.9
6'SL	G1	PRE-TERM	43	10	77.47	185.6	121.2	37.02	106.2	94.83	150.4
6'SL	G1	PRE-TERM	44	5	84.36	212.2	163.0	49.65	165.2	155.7	197.7
6'SL	G1	PRE-TERM	45	9	46.43	176.7	97.68	43.38	85.22	61.73	126.5
6'SL	G1	PRE-TERM	46	6	61.11	196.6	125.1	54.95	115.8	84.79	168.9
6'SL	G1	PRE-TERM	47	3	79.24	109.6	95.55	15.29	97.87	88.55	103.7
6'SL	G1	PRE-TERM	48	4	48.01	142.2	78.59	43.01	62.07	56.20	84.46
6'SL	G1	TERM	38	2	313.5	442.1	377.8	90.97	377.8	345.7	410.0
6'SL	G1	TERM	39	9	222.0	706.2	434.5	136.5	429.6	363.7	515.2
6'SL	G1	TERM	40	13	325.3	1084	605.1	226.5	581.8	428.5	692.4
6'SL	G1	TERM	41	21	216.1	985.4	585.4	207.5	552.4	440.2	775.3
6'SL	G1	TERM	42	21	198.9	821.3	570.2	185.8	578.1	463.3	701.3
6'SL	G1	TERM	43	21	153.7	792.3	482.4	183.9	511.6	343.0	588.8
6'SL	G1	TERM	44	20	24.60	574.6	355.1	156.8	406.7	252.3	450.9
6'SL	G1	TERM	45	21	93.75	700.9	324.7	149.5	340.6	213.6	397.6
6'SL	G1	TERM	46	17	53.06	662.3	292.4	138.9	264.3	219.4	325.9
6'SL	G1	TERM	47	12	99.59	671.2	283.1	154.6	253.2	194.4	325.3
6'SL	G1	TERM	48	6	145.8	301.0	206.9	57.71	193.6	168.6	234.0
6'SL	G2	PRE-TERM	29	1	303.3	303.3	303.3	NA	303.3	303.3	303.3
6'SL	G2	PRE-TERM	30	2	145.3	224.7	185.0	56.15	185.0	165.2	204.9
6'SL	G2	PRE-TERM	31	2	86.13	309.8	198.0	158.1	198.0	142.0	253.9
6'SL	G2	PRE-TERM	32	3	61.39	263.9	185.3	108.6	230.5	145.9	247.2
6'SL	G2	PRE-TERM	33	5	53.89	501.0	342.9	175.5	367.0	331.6	461.0
6'SL	G2	PRE-TERM	34	5	74.89	559.2	326.9	172.0	328.2	318.4	353.9
6'SL	G2	PRE-TERM	35	5	74.47	555.9	317.1	171.7	301.0	299.8	354.5
6'SL	G2	PRE-TERM	36	5	55.02	467.7	270.2	148.2	264.0	249.1	315.4

Table S4 Concentration of Human Milk Oligosaccharides in Term or Preterm Milk At Specified Postmenstrual Age Separated By Milk Group

** When there are results below the method limit of quanfification (LoQ) the result has been assigned value of 0.5 × LoQ, hence the minimum value appears to be the same in many cases. When a large number of datapoints are below LoQ this can also have the effect that the median = minimum.*

					HMO Concentration (mg/L)						
HMO	Milk Group	Study Arm	Postmenstrual Age (weeks)	N	min *	max	mean	sd	median *	Quartile 1 (25%)	Quartile 3 (75%)
6'SL	G2	PRE-TERM	37	4	57.52	313.4	206.6	108.4	227.6	170.5	263.6
6'SL	G2	PRE-TERM	38	4	177.8	325.7	235.9	67.41	220.1	187.7	268.3
6'SL	G2	PRE-TERM	39	3	142.1	296.6	202.2	82.75	168.0	155.0	232.3
6'SL	G2	PRE-TERM	40	3	146.4	208.3	185.7	34.16	202.3	174.3	205.3
6'SL	G2	PRE-TERM	41	2	122.1	171.1	146.6	34.62	146.6	134.4	158.9
6'SL	G2	PRE-TERM	42	3	100.6	171.4	143.6	37.77	158.9	129.8	165.1
6'SL	G2	PRE-TERM	43	2	69.80	114.2	91.99	31.39	91.99	80.90	103.1
6'SL	G2	PRE-TERM	44	3	65.69	156.3	119.1	47.42	135.2	100.4	145.8
6'SL	G2	PRE-TERM	45	2	77.58	109.5	93.53	22.56	93.53	85.55	101.5
6'SL	G2	PRE-TERM	46	2	74.24	145.4	109.8	50.29	109.8	92.02	127.6
6'SL	G2	PRE-TERM	48	1	126.1	126.1	126.1	NA	126.1	126.1	126.1
6'SL	G2	TERM	39	1	374.6	374.6	374.6	NA	374.6	374.6	374.6
6'SL	G2	TERM	40	5	295.7	634.7	452.3	164.2	401.4	311.3	618.4
6'SL	G2	TERM	41	4	262.0	786.1	557.9	257.1	591.8	383.3	766.3
6'SL	G2	TERM	42	6	202.8	718.6	476.2	178.0	507.9	389.4	554.6
6'SL	G2	TERM	43	5	129.4	623.8	403.0	184.2	457.8	334.8	469.3
6'SL	G2	TERM	44	5	112.5	549.1	332.9	163.0	365.6	249.8	387.3
6'SL	G2	TERM	45	5	89.66	318.7	200.0	96.04	179.5	134.9	277.4
6'SL	G2	TERM	46	5	86.83	234.6	173.1	73.64	220.1	98.67	225.2
6'SL	G2	TERM	47	4	129.6	211.6	180.6	37.30	190.6	164.5	206.6
6'SL	G3	PRE-TERM	30	1	673.9	673.9	673.9	NA	673.9	673.9	673.9
6'SL	G3	PRE-TERM	31	1	654.3	654.3	654.3	NA	654.3	654.3	654.3
6'SL	G3	PRE-TERM	32	1	441.3	441.3	441.3	NA	441.3	441.3	441.3
6'SL	G3	PRE-TERM	33	1	324.0	324.0	324.0	NA	324.0	324.0	324.0
6'SL	G3	PRE-TERM	34	1	270.7	270.7	270.7	NA	270.7	270.7	270.7
6'SL	G3	PRE-TERM	35	1	229.1	229.1	229.1	NA	229.1	229.1	229.1
6'SL	G3	PRE-TERM	36	1	128.6	128.6	128.6	NA	128.6	128.6	128.6
6'SL	G3	PRE-TERM	37	1	138.2	138.2	138.2	NA	138.2	138.2	138.2
6'SL	G3	PRE-TERM	39	1	91.24	91.24	91.24	NA	91.24	91.24	91.24
6'SL	G3	PRE-TERM	41	1	72.75	72.75	72.75	NA	72.75	72.75	72.75
6'SL	G3	PRE-TERM	43	1	60.09	60.09	60.09	NA	60.09	60.09	60.09
6'SL	G3	PRE-TERM	45	1	48.01	48.01	48.01	NA	48.01	48.01	48.01
6'SL	G3	TERM	41	1	648.7	648.7	648.7	NA	648.7	648.7	648.7
6'SL	G3	TERM	42	1	598.9	598.9	598.9	NA	598.9	598.9	598.9
6'SL	G3	TERM	43	1	511.9	511.9	511.9	NA	511.9	511.9	511.9
6'SL	G3	TERM	44	1	419.6	419.6	419.6	NA	419.6	419.6	419.6

Table S4 Concentration of Human Milk Oligosaccharides in Term or Preterm Milk At Specified Postmenstrual Age Separated By Milk Group

** When there are results below the method limit of quanfification (LoQ) the result has been assigned value of 0.5 × LoQ, hence the minimum value appears to be the same in many cases. When a large number of datapoints are below LoQ this can also have the effect that the median = minimum.*

					HMO Concentration (mg/L)						
HMO	Milk Group	Study Arm	Postmenstrual Age (weeks)	N	min *	max	mean	sd	median *	Quartile 1 (25%)	Quartile 3 (75%)
6'SL	G3	TERM	45	1	355.0	355.0	355.0	NA	355.0	355.0	355.0
6'SL	G3	TERM	46	1	294.2	294.2	294.2	NA	294.2	294.2	294.2
6'SL	G3	TERM	47	1	273.7	273.7	273.7	NA	273.7	273.7	273.7
6'SL	G3	TERM	48	1	192.3	192.3	192.3	NA	192.3	192.3	192.3
6'SL	G4	TERM	40	1	436.4	436.4	436.4	NA	436.4	436.4	436.4
6'SL	G4	TERM	41	1	888.0	888.0	888.0	NA	888.0	888.0	888.0
6'SL	G4	TERM	42	1	670.5	670.5	670.5	NA	670.5	670.5	670.5
6'SL	G4	TERM	43	1	489.7	489.7	489.7	NA	489.7	489.7	489.7
6'SL	G4	TERM	44	1	357.8	357.8	357.8	NA	357.8	357.8	357.8
6'SL	G4	TERM	45	1	305.6	305.6	305.6	NA	305.6	305.6	305.6
6'SL	G4	TERM	46	1	193.1	193.1	193.1	NA	193.1	193.1	193.1
6'SL	G4	TERM	47	1	150.4	150.4	150.4	NA	150.4	150.4	150.4
A-Tetrasaccharide	G1	PRE-TERM	30	7	7.500	166.6	35.16	59.36	7.500	7.500	24.77
A-Tetrasaccharide	G1	PRE-TERM	31	9	7.500	70.30	22.86	25.65	7.500	7.500	27.92
A-Tetrasaccharide	G1	PRE-TERM	32	13	7.500	315.1	59.67	92.18	7.500	7.500	63.10
A-Tetrasaccharide	G1	PRE-TERM	33	18	7.500	232.5	63.50	76.51	16.56	7.500	111.3
A-Tetrasaccharide	G1	PRE-TERM	34	19	7.500	250.0	42.26	69.11	7.500	7.500	29.69
A-Tetrasaccharide	G1	PRE-TERM	35	18	7.500	224.7	54.04	74.85	7.500	7.500	75.70
A-Tetrasaccharide	G1	PRE-TERM	36	19	7.500	226.0	51.82	73.58	7.500	7.500	70.55
A-Tetrasaccharide	G1	PRE-TERM	37	18	7.500	232.6	49.80	68.75	7.500	7.500	80.93
A-Tetrasaccharide	G1	PRE-TERM	38	11	7.500	266.6	79.38	98.02	29.48	7.500	127.6
A-Tetrasaccharide	G1	PRE-TERM	39	16	7.500	322.7	58.57	98.16	7.500	7.500	46.20
A-Tetrasaccharide	G1	PRE-TERM	40	7	7.500	113.0	38.84	48.46	7.500	7.500	64.43
A-Tetrasaccharide	G1	PRE-TERM	41	12	7.500	334.5	59.46	103.5	7.500	7.500	43.59
A-Tetrasaccharide	G1	PRE-TERM	42	6	7.500	101.9	41.03	46.46	15.07	7.500	80.02
A-Tetrasaccharide	G1	PRE-TERM	43	10	7.500	300.7	62.70	102.0	7.500	7.500	68.70
A-Tetrasaccharide	G1	PRE-TERM	44	5	7.500	86.79	26.38	34.40	7.500	7.500	22.62
A-Tetrasaccharide	G1	PRE-TERM	45	9	7.500	329.8	67.31	109.5	7.500	7.500	76.21
A-Tetrasaccharide	G1	PRE-TERM	46	6	7.500	132.3	47.60	55.13	18.55	7.500	83.31
A-Tetrasaccharide	G1	PRE-TERM	47	3	7.500	144.8	77.28	68.69	79.52	43.51	112.2
A-Tetrasaccharide	G1	PRE-TERM	48	4	7.500	102.4	38.41	44.78	21.85	7.500	52.75
A-Tetrasaccharide	G1	TERM	38	2	7.500	7.500	7.500	0.0000	7.500	7.500	7.500
A-Tetrasaccharide	G1	TERM	39	9	7.500	188.2	39.40	60.37	7.500	7.500	59.33
A-Tetrasaccharide	G1	TERM	40	13	7.500	154.4	40.96	45.36	39.01	7.500	43.87
A-Tetrasaccharide	G1	TERM	41	21	7.500	150.2	30.18	37.43	7.500	7.500	40.31
A-Tetrasaccharide	G1	TERM	42	21	7.500	149.0	29.66	38.74	7.500	7.500	39.78

Table S4 Concentration of Human Milk Oligosaccharides in Term or Preterm Milk At Specified Postmenstrual Age Separated By Milk Group

** When there are results below the method limit of quanfification (LoQ) the result has been assigned value of 0.5 × LoQ, hence the minimum value appears to be the same in many cases. When a large number of datapoints are below LoQ this can also have the effect that the median = minimum.*

					HMO Concentration (mg/L)						
HMO	Milk Group	Study Arm	Postmenstrual Age (weeks)	N	min *	max	mean	sd	median *	Quartile 1 (25%)	Quartile 3 (75%)
A-Tetrasaccharide	G1	TERM	43	21	7.500	153.9	30.04	38.48	7.500	7.500	35.93
A-Tetrasaccharide	G1	TERM	44	20	7.500	162.3	31.15	41.34	11.26	7.500	29.04
A-Tetrasaccharide	G1	TERM	45	21	7.500	138.9	27.46	37.58	7.500	7.500	29.91
A-Tetrasaccharide	G1	TERM	46	17	7.500	183.8	37.09	50.05	7.500	7.500	39.48
A-Tetrasaccharide	G1	TERM	47	12	7.500	165.4	38.68	53.12	7.500	7.500	55.70
A-Tetrasaccharide	G1	TERM	48	6	7.500	181.0	52.72	74.03	7.500	7.500	80.83
A-Tetrasaccharide	G2	PRE-TERM	29	1	7.500	7.500	7.500	NA	7.500	7.500	7.500
A-Tetrasaccharide	G2	PRE-TERM	30	2	7.500	7.500	7.500	0.0000	7.500	7.500	7.500
A-Tetrasaccharide	G2	PRE-TERM	31	2	7.500	7.500	7.500	0.0000	7.500	7.500	7.500
A-Tetrasaccharide	G2	PRE-TERM	32	3	7.500	7.500	7.500	0.0000	7.500	7.500	7.500
A-Tetrasaccharide	G2	PRE-TERM	33	5	7.500	7.500	7.500	0.0000	7.500	7.500	7.500
A-Tetrasaccharide	G2	PRE-TERM	34	5	7.500	7.500	7.500	0.0000	7.500	7.500	7.500
A-Tetrasaccharide	G2	PRE-TERM	35	5	7.500	16.81	9.361	4.162	7.500	7.500	7.500
A-Tetrasaccharide	G2	PRE-TERM	36	5	7.500	7.500	7.500	0.0000	7.500	7.500	7.500
A-Tetrasaccharide	G2	PRE-TERM	37	4	7.500	7.500	7.500	0.0000	7.500	7.500	7.500
A-Tetrasaccharide	G2	PRE-TERM	38	4	7.500	7.500	7.500	0.0000	7.500	7.500	7.500
A-Tetrasaccharide	G2	PRE-TERM	39	3	7.500	7.500	7.500	0.0000	7.500	7.500	7.500
A-Tetrasaccharide	G2	PRE-TERM	40	3	7.500	7.500	7.500	0.0000	7.500	7.500	7.500
A-Tetrasaccharide	G2	PRE-TERM	41	2	7.500	7.500	7.500	0.0000	7.500	7.500	7.500
A-Tetrasaccharide	G2	PRE-TERM	42	3	7.500	7.500	7.500	0.0000	7.500	7.500	7.500
A-Tetrasaccharide	G2	PRE-TERM	43	2	7.500	7.500	7.500	0.0000	7.500	7.500	7.500
A-Tetrasaccharide	G2	PRE-TERM	44	3	7.500	7.500	7.500	0.0000	7.500	7.500	7.500
A-Tetrasaccharide	G2	PRE-TERM	45	2	7.500	7.500	7.500	0.0000	7.500	7.500	7.500
A-Tetrasaccharide	G2	PRE-TERM	46	2	7.500	7.500	7.500	0.0000	7.500	7.500	7.500
A-Tetrasaccharide	G2	PRE-TERM	48	1	7.500	7.500	7.500	NA	7.500	7.500	7.500
A-Tetrasaccharide	G2	TERM	39	1	7.500	7.500	7.500	NA	7.500	7.500	7.500
A-Tetrasaccharide	G2	TERM	40	5	7.500	7.500	7.500	0.0000	7.500	7.500	7.500
A-Tetrasaccharide	G2	TERM	41	4	7.500	7.500	7.500	0.0000	7.500	7.500	7.500
A-Tetrasaccharide	G2	TERM	42	6	7.500	7.500	7.500	0.0000	7.500	7.500	7.500
A-Tetrasaccharide	G2	TERM	43	5	7.500	28.98	11.80	9.605	7.500	7.500	7.500
A-Tetrasaccharide	G2	TERM	44	5	7.500	27.37	11.47	8.886	7.500	7.500	7.500
A-Tetrasaccharide	G2	TERM	45	5	7.500	7.500	7.500	0.0000	7.500	7.500	7.500
A-Tetrasaccharide	G2	TERM	46	5	7.500	7.500	7.500	0.0000	7.500	7.500	7.500
A-Tetrasaccharide	G2	TERM	47	4	7.500	7.500	7.500	0.0000	7.500	7.500	7.500
A-Tetrasaccharide	G3	PRE-TERM	30	1	7.500	7.500	7.500	NA	7.500	7.500	7.500
A-Tetrasaccharide	G3	PRE-TERM	31	1	7.500	7.500	7.500	NA	7.500	7.500	7.500

Table S4 Concentration of Human Milk Oligosaccharides in Term or Preterm Milk At Specified Postmenstrual Age Separated By Milk Group

** When there are results below the method limit of quanfification (LoQ) the result has been assigned value of 0.5 × LoQ, hence the minimum value appears to be the same in many cases. When a large number of datapoints are below LoQ this can also have the effect that the median = minimum.*

HMO	Milk Group	Study Arm	Postmenstrual Age (weeks)	N	HMO Concentration (mg/L) min *	max	mean	sd	median *	Quartile 1 (25%)	Quartile 3 (75%)
A-Tetrasaccharide	G3	PRE-TERM	32	1	7.500	7.500	7.500	NA	7.500	7.500	7.500
A-Tetrasaccharide	G3	PRE-TERM	33	1	7.500	7.500	7.500	NA	7.500	7.500	7.500
A-Tetrasaccharide	G3	PRE-TERM	34	1	7.500	7.500	7.500	NA	7.500	7.500	7.500
A-Tetrasaccharide	G3	PRE-TERM	35	1	7.500	7.500	7.500	NA	7.500	7.500	7.500
A-Tetrasaccharide	G3	PRE-TERM	36	1	7.500	7.500	7.500	NA	7.500	7.500	7.500
A-Tetrasaccharide	G3	PRE-TERM	37	1	7.500	7.500	7.500	NA	7.500	7.500	7.500
A-Tetrasaccharide	G3	PRE-TERM	39	1	7.500	7.500	7.500	NA	7.500	7.500	7.500
A-Tetrasaccharide	G3	PRE-TERM	41	1	7.500	7.500	7.500	NA	7.500	7.500	7.500
A-Tetrasaccharide	G3	PRE-TERM	43	1	7.500	7.500	7.500	NA	7.500	7.500	7.500
A-Tetrasaccharide	G3	PRE-TERM	45	1	7.500	7.500	7.500	NA	7.500	7.500	7.500
A-Tetrasaccharide	G3	TERM	41	1	43.15	43.15	43.15	NA	43.15	43.15	43.15
A-Tetrasaccharide	G3	TERM	42	1	47.28	47.28	47.28	NA	47.28	47.28	47.28
A-Tetrasaccharide	G3	TERM	43	1	46.17	46.17	46.17	NA	46.17	46.17	46.17
A-Tetrasaccharide	G3	TERM	44	1	41.87	41.87	41.87	NA	41.87	41.87	41.87
A-Tetrasaccharide	G3	TERM	45	1	36.38	36.38	36.38	NA	36.38	36.38	36.38
A-Tetrasaccharide	G3	TERM	46	1	37.04	37.04	37.04	NA	37.04	37.04	37.04
A-Tetrasaccharide	G3	TERM	47	1	27.14	27.14	27.14	NA	27.14	27.14	27.14
A-Tetrasaccharide	G3	TERM	48	1	33.98	33.98	33.98	NA	33.98	33.98	33.98
A-Tetrasaccharide	G4	TERM	40	1	7.500	7.500	7.500	NA	7.500	7.500	7.500
A-Tetrasaccharide	G4	TERM	41	1	7.500	7.500	7.500	NA	7.500	7.500	7.500
A-Tetrasaccharide	G4	TERM	42	1	7.500	7.500	7.500	NA	7.500	7.500	7.500
A-Tetrasaccharide	G4	TERM	43	1	7.500	7.500	7.500	NA	7.500	7.500	7.500
A-Tetrasaccharide	G4	TERM	44	1	7.500	7.500	7.500	NA	7.500	7.500	7.500
A-Tetrasaccharide	G4	TERM	45	1	7.500	7.500	7.500	NA	7.500	7.500	7.500
A-Tetrasaccharide	G4	TERM	46	1	7.500	7.500	7.500	NA	7.500	7.500	7.500
A-Tetrasaccharide	G4	TERM	47	1	7.500	7.500	7.500	NA	7.500	7.500	7.500
DFLNHa	G1	PRE-TERM	30	7	16.50	266.3	140.5	91.70	132.0	79.18	205.1
DFLNHa	G1	PRE-TERM	31	9	16.50	448.7	225.1	165.6	197.9	95.57	383.8
DFLNHa	G1	PRE-TERM	32	13	16.50	420.0	210.8	128.9	201.2	111.1	334.0
DFLNHa	G1	PRE-TERM	33	18	16.50	354.5	209.0	116.9	245.1	103.4	306.7
DFLNHa	G1	PRE-TERM	34	19	16.50	291.2	154.1	87.41	126.6	81.00	231.7
DFLNHa	G1	PRE-TERM	35	18	16.50	229.0	132.1	59.63	147.2	103.3	170.2
DFLNHa	G1	PRE-TERM	36	19	16.50	295.0	152.8	79.73	143.3	97.78	192.6
DFLNHa	G1	PRE-TERM	37	18	16.50	252.0	117.8	66.39	114.9	74.98	153.4
DFLNHa	G1	PRE-TERM	38	11	16.50	685.1	149.2	182.6	107.6	69.52	143.6
DFLNHa	G1	PRE-TERM	39	16	16.50	142.9	70.59	42.51	72.94	31.91	97.26

Table S4 Concentration of Human Milk Oligosaccharides in Term or Preterm Milk At Specified Postmenstrual Age Separated By Milk Group

** When there are results below the method limit of quanfification (LoQ) the result has been assigned value of 0.5 × LoQ, hence the minimum value appears to be the same in many cases. When a large number of datapoints are below LoQ this can also have the effect that the median = minimum.*

					HMO Concentration (mg/L)						
HMO	Milk Group	Study Arm	Postmenstrual Age (weeks)	N	min *	max	mean	sd	median *	Quartile 1 (25%)	Quartile 3 (75%)
DFLNHa	G1	PRE-TERM	40	7	16.50	230.0	102.9	73.30	67.08	61.51	142.0
DFLNHa	G1	PRE-TERM	41	12	16.50	83.83	40.55	22.89	37.02	16.50	58.06
DFLNHa	G1	PRE-TERM	42	6	16.50	103.6	63.96	40.13	72.26	27.18	98.29
DFLNHa	G1	PRE-TERM	43	10	16.50	83.70	47.16	20.27	48.30	44.09	51.48
DFLNHa	G1	PRE-TERM	44	5	16.50	77.74	48.86	23.18	49.76	38.65	61.64
DFLNHa	G1	PRE-TERM	45	9	16.50	61.96	27.05	16.93	16.50	16.50	39.86
DFLNHa	G1	PRE-TERM	46	6	16.50	60.97	45.07	16.70	47.22	39.64	57.83
DFLNHa	G1	PRE-TERM	47	3	16.50	52.20	35.51	17.96	37.84	27.17	45.02
DFLNHa	G1	PRE-TERM	48	4	16.50	51.52	32.67	18.80	31.33	16.50	47.49
DFLNHa	G1	TERM	38	2	130.3	159.2	144.7	20.46	144.7	137.5	152.0
DFLNHa	G1	TERM	39	9	16.50	306.3	139.4	102.8	81.69	66.19	208.4
DFLNHa	G1	TERM	40	13	90.49	399.0	183.7	89.40	153.7	131.6	219.9
DFLNHa	G1	TERM	41	21	89.36	427.0	221.3	89.35	203.4	158.2	267.7
DFLNHa	G1	TERM	42	21	79.80	582.8	260.0	134.0	224.3	193.0	326.9
DFLNHa	G1	TERM	43	21	66.82	473.0	225.4	132.9	190.4	123.5	342.6
DFLNHa	G1	TERM	44	20	34.26	405.6	189.9	127.1	157.5	86.28	284.4
DFLNHa	G1	TERM	45	21	16.50	355.9	153.9	108.4	125.4	54.75	240.4
DFLNHa	G1	TERM	46	17	16.50	293.0	122.6	81.81	115.7	68.25	190.0
DFLNHa	G1	TERM	47	12	16.50	311.2	133.8	86.07	125.0	77.98	158.5
DFLNHa	G1	TERM	48	6	81.61	264.3	136.0	66.76	115.9	96.87	139.6
DFLNHa	G2	PRE-TERM	29	1	16.50	16.50	16.50	NA	16.50	16.50	16.50
DFLNHa	G2	PRE-TERM	30	2	16.50	16.50	16.50	0.0000	16.50	16.50	16.50
DFLNHa	G2	PRE-TERM	31	2	16.50	16.50	16.50	0.0000	16.50	16.50	16.50
DFLNHa	G2	PRE-TERM	32	3	16.50	16.50	16.50	0.0000	16.50	16.50	16.50
DFLNHa	G2	PRE-TERM	33	5	16.50	16.50	16.50	0.0000	16.50	16.50	16.50
DFLNHa	G2	PRE-TERM	34	5	16.50	16.50	16.50	0.0000	16.50	16.50	16.50
DFLNHa	G2	PRE-TERM	35	5	16.50	16.50	16.50	0.0000	16.50	16.50	16.50
DFLNHa	G2	PRE-TERM	36	5	16.50	42.44	21.69	11.60	16.50	16.50	16.50
DFLNHa	G2	PRE-TERM	37	4	16.50	42.01	22.88	12.75	16.50	16.50	22.88
DFLNHa	G2	PRE-TERM	38	4	16.50	16.50	16.50	0.0000	16.50	16.50	16.50
DFLNHa	G2	PRE-TERM	39	3	16.50	16.50	16.50	0.0000	16.50	16.50	16.50
DFLNHa	G2	PRE-TERM	40	3	16.50	16.50	16.50	0.0000	16.50	16.50	16.50
DFLNHa	G2	PRE-TERM	41	2	16.50	36.86	26.68	14.39	26.68	21.59	31.77
DFLNHa	G2	PRE-TERM	42	3	16.50	16.50	16.50	0.0000	16.50	16.50	16.50
DFLNHa	G2	PRE-TERM	43	2	16.50	16.50	16.50	0.0000	16.50	16.50	16.50
DFLNHa	G2	PRE-TERM	44	3	16.50	16.50	16.50	0.0000	16.50	16.50	16.50

Table S4 Concentration of Human Milk Oligosaccharides in Term or Preterm Milk At Specified Postmenstrual Age Separated By Milk Group

** When there are results below the method limit of quanfification (LoQ) the result has been assigned value of 0.5 × LoQ, hence the minimum value appears to be the same in many cases. When a large number of datapoints are below LoQ this can also have the effect that the median = minimum.*

HMO	Milk Group	Study Arm	Postmenstrual Age (weeks)	N	HMO Concentration (mg/L) min *	max	mean	sd	median *	Quartile 1 (25%)	Quartile 3 (75%)
DFLNHa	G2	PRE-TERM	45	2	16.50	139.6	78.06	87.06	78.06	47.28	108.8
DFLNHa	G2	PRE-TERM	46	2	16.50	16.50	16.50	0.0000	16.50	16.50	16.50
DFLNHa	G2	PRE-TERM	48	1	16.50	16.50	16.50	NA	16.50	16.50	16.50
DFLNHa	G2	TERM	39	1	16.50	16.50	16.50	NA	16.50	16.50	16.50
DFLNHa	G2	TERM	40	5	16.50	16.50	16.50	0.0000	16.50	16.50	16.50
DFLNHa	G2	TERM	41	4	16.50	16.50	16.50	0.0000	16.50	16.50	16.50
DFLNHa	G2	TERM	42	6	16.50	16.50	16.50	0.0000	16.50	16.50	16.50
DFLNHa	G2	TERM	43	5	16.50	16.50	16.50	0.0000	16.50	16.50	16.50
DFLNHa	G2	TERM	44	5	16.50	16.50	16.50	0.0000	16.50	16.50	16.50
DFLNHa	G2	TERM	45	5	16.50	16.50	16.50	0.0000	16.50	16.50	16.50
DFLNHa	G2	TERM	46	5	16.50	16.50	16.50	0.0000	16.50	16.50	16.50
DFLNHa	G2	TERM	47	4	16.50	16.50	16.50	0.0000	16.50	16.50	16.50
DFLNHa	G3	PRE-TERM	30	1	577.3	577.3	577.3	NA	577.3	577.3	577.3
DFLNHa	G3	PRE-TERM	31	1	495.2	495.2	495.2	NA	495.2	495.2	495.2
DFLNHa	G3	PRE-TERM	32	1	430.4	430.4	430.4	NA	430.4	430.4	430.4
DFLNHa	G3	PRE-TERM	33	1	583.6	583.6	583.6	NA	583.6	583.6	583.6
DFLNHa	G3	PRE-TERM	34	1	623.8	623.8	623.8	NA	623.8	623.8	623.8
DFLNHa	G3	PRE-TERM	35	1	542.4	542.4	542.4	NA	542.4	542.4	542.4
DFLNHa	G3	PRE-TERM	36	1	418.7	418.7	418.7	NA	418.7	418.7	418.7
DFLNHa	G3	PRE-TERM	37	1	351.9	351.9	351.9	NA	351.9	351.9	351.9
DFLNHa	G3	PRE-TERM	39	1	249.7	249.7	249.7	NA	249.7	249.7	249.7
DFLNHa	G3	PRE-TERM	41	1	179.2	179.2	179.2	NA	179.2	179.2	179.2
DFLNHa	G3	PRE-TERM	43	1	58.00	58.00	58.00	NA	58.00	58.00	58.00
DFLNHa	G3	PRE-TERM	45	1	54.78	54.78	54.78	NA	54.78	54.78	54.78
DFLNHa	G3	TERM	41	1	488.4	488.4	488.4	NA	488.4	488.4	488.4
DFLNHa	G3	TERM	42	1	664.0	664.0	664.0	NA	664.0	664.0	664.0
DFLNHa	G3	TERM	43	1	759.1	759.1	759.1	NA	759.1	759.1	759.1
DFLNHa	G3	TERM	44	1	709.4	709.4	709.4	NA	709.4	709.4	709.4
DFLNHa	G3	TERM	45	1	747.0	747.0	747.0	NA	747.0	747.0	747.0
DFLNHa	G3	TERM	46	1	563.0	563.0	563.0	NA	563.0	563.0	563.0
DFLNHa	G3	TERM	47	1	525.5	525.5	525.5	NA	525.5	525.5	525.5
DFLNHa	G3	TERM	48	1	463.1	463.1	463.1	NA	463.1	463.1	463.1
DFLNHa	G4	TERM	40	1	16.50	16.50	16.50	NA	16.50	16.50	16.50
DFLNHa	G4	TERM	41	1	52.78	52.78	52.78	NA	52.78	52.78	52.78
DFLNHa	G4	TERM	42	1	16.50	16.50	16.50	NA	16.50	16.50	16.50
DFLNHa	G4	TERM	43	1	16.50	16.50	16.50	NA	16.50	16.50	16.50

Table S4 Concentration of Human Milk Oligosaccharides in Term or Preterm Milk At Specified Postmenstrual Age Separated By Milk Group

** When there are results below the method limit of quanfification (LoQ) the result has been assigned value of 0.5 × LoQ, hence the minimum value appears to be the same in many cases. When a large number of datapoints are below LoQ this can also have the effect that the median = minimum.*

					HMO Concentration (mg/L)						
HMO	Milk Group	Study Arm	Postmenstrual Age (weeks)	N	min *	max	mean	sd	median *	Quartile 1 (25%)	Quartile 3 (75%)
DFLNHa	G4	TERM	44	1	16.50	16.50	16.50	NA	16.50	16.50	16.50
DFLNHa	G4	TERM	45	1	16.50	16.50	16.50	NA	16.50	16.50	16.50
DFLNHa	G4	TERM	46	1	16.50	16.50	16.50	NA	16.50	16.50	16.50
DFLNHa	G4	TERM	47	1	16.50	16.50	16.50	NA	16.50	16.50	16.50
DSLNT	G1	PRE-TERM	30	7	17.50	454.1	301.8	143.2	327.6	266.6	390.0
DSLNT	G1	PRE-TERM	31	9	17.50	534.4	335.1	186.9	397.1	357.5	437.9
DSLNT	G1	PRE-TERM	32	13	193.6	605.8	365.8	117.9	345.9	282.9	443.0
DSLNT	G1	PRE-TERM	33	18	197.2	703.2	394.7	150.7	328.5	297.5	508.9
DSLNT	G1	PRE-TERM	34	19	169.1	822.5	416.9	198.5	381.5	253.5	554.3
DSLNT	G1	PRE-TERM	35	18	128.4	908.0	418.2	238.9	315.3	261.5	561.8
DSLNT	G1	PRE-TERM	36	19	129.9	796.0	326.0	181.1	223.8	205.5	400.4
DSLNT	G1	PRE-TERM	37	18	105.5	725.2	295.8	166.5	265.2	181.9	349.2
DSLNT	G1	PRE-TERM	38	11	100.2	628.2	272.8	165.2	212.8	160.6	328.5
DSLNT	G1	PRE-TERM	39	16	97.12	597.5	270.0	156.8	201.0	174.3	321.6
DSLNT	G1	PRE-TERM	40	7	105.8	530.6	349.6	154.3	340.1	262.3	472.9
DSLNT	G1	PRE-TERM	41	12	84.32	413.6	208.5	85.67	188.7	169.5	246.9
DSLNT	G1	PRE-TERM	42	6	72.56	422.5	264.4	126.4	292.2	192.1	334.5
DSLNT	G1	PRE-TERM	43	10	132.9	435.1	215.3	88.80	186.4	158.7	240.2
DSLNT	G1	PRE-TERM	44	5	102.0	318.0	225.7	82.93	240.7	193.2	274.7
DSLNT	G1	PRE-TERM	45	9	121.3	253.6	177.6	44.11	170.0	145.6	205.6
DSLNT	G1	PRE-TERM	46	6	88.23	311.8	204.7	88.33	218.0	136.8	266.3
DSLNT	G1	PRE-TERM	47	3	103.0	158.2	133.9	28.20	140.5	121.8	149.4
DSLNT	G1	PRE-TERM	48	4	124.7	219.3	175.7	39.05	179.4	162.1	193.0
DSLNT	G1	TERM	38	2	412.7	433.7	423.2	14.79	423.2	418.0	428.4
DSLNT	G1	TERM	39	9	248.3	489.2	369.9	75.78	366.5	337.5	408.4
DSLNT	G1	TERM	40	13	203.7	649.5	383.0	130.4	348.3	266.1	468.7
DSLNT	G1	TERM	41	21	212.2	880.1	373.3	138.2	374.1	286.2	401.3
DSLNT	G1	TERM	42	21	17.50	836.5	343.5	162.5	353.3	249.9	406.8
DSLNT	G1	TERM	43	21	117.1	630.2	303.3	115.3	293.3	223.6	342.1
DSLNT	G1	TERM	44	20	94.18	555.7	253.8	108.2	237.0	173.9	313.2
DSLNT	G1	TERM	45	21	75.20	426.1	225.7	84.09	202.7	163.1	286.9
DSLNT	G1	TERM	46	17	69.19	437.5	208.4	94.82	181.6	129.7	256.8
DSLNT	G1	TERM	47	12	48.04	422.7	195.2	103.0	183.4	133.7	262.7
DSLNT	G1	TERM	48	6	60.14	223.1	149.5	68.91	156.0	97.78	206.4
DSLNT	G2	PRE-TERM	29	1	946.4	946.4	946.4	NA	946.4	946.4	946.4
DSLNT	G2	PRE-TERM	30	2	340.2	843.2	591.7	355.7	591.7	465.9	717.4

Table S4 Concentration of Human Milk Oligosaccharides in Term or Preterm Milk At Specified Postmenstrual Age Separated By Milk Group

** When there are results below the method limit of quanfification (LoQ) the result has been assigned value of 0.5 × LoQ, hence the minimum value appears to be the same in many cases. When a large number of datapoints are below LoQ this can also have the effect that the median = minimum.*

					HMO Concentration (mg/L)						
HMO	Milk Group	Study Arm	Postmenstrual Age (weeks)	N	min *	max	mean	sd	median *	Quartile 1 (25%)	Quartile 3 (75%)
DSLNT	G2	PRE-TERM	31	2	486.4	786.4	636.4	212.1	636.4	561.4	711.4
DSLNT	G2	PRE-TERM	32	3	511.9	719.0	595.4	109.2	555.3	533.6	637.1
DSLNT	G2	PRE-TERM	33	5	443.0	843.4	647.1	147.5	661.2	586.5	701.2
DSLNT	G2	PRE-TERM	34	5	344.4	590.1	502.3	100.9	557.9	460.2	559.2
DSLNT	G2	PRE-TERM	35	5	312.6	579.5	470.2	102.4	503.0	433.2	522.6
DSLNT	G2	PRE-TERM	36	5	249.6	677.8	456.7	164.7	420.6	380.6	554.9
DSLNT	G2	PRE-TERM	37	4	364.8	411.8	390.4	20.70	392.6	378.8	404.2
DSLNT	G2	PRE-TERM	38	4	182.5	430.6	325.1	109.5	343.6	270.6	398.1
DSLNT	G2	PRE-TERM	39	3	226.1	364.4	285.1	71.36	264.8	245.4	314.6
DSLNT	G2	PRE-TERM	40	3	160.9	277.2	232.6	62.67	259.6	210.3	268.4
DSLNT	G2	PRE-TERM	41	2	232.1	452.7	342.4	156.0	342.4	287.3	397.6
DSLNT	G2	PRE-TERM	42	3	144.4	256.3	186.5	60.84	158.8	151.6	207.5
DSLNT	G2	PRE-TERM	43	2	131.9	321.5	226.7	134.1	226.7	179.3	274.1
DSLNT	G2	PRE-TERM	44	3	118.9	239.5	165.1	65.03	136.9	127.9	188.2
DSLNT	G2	PRE-TERM	45	2	131.9	459.9	295.9	231.9	295.9	213.9	377.9
DSLNT	G2	PRE-TERM	46	2	176.5	230.5	203.5	38.18	203.5	190.0	217.0
DSLNT	G2	PRE-TERM	48	1	212.0	212.0	212.0	NA	212.0	212.0	212.0
DSLNT	G2	TERM	39	1	262.5	262.5	262.5	NA	262.5	262.5	262.5
DSLNT	G2	TERM	40	5	146.3	540.0	344.8	151.9	304.3	288.7	444.5
DSLNT	G2	TERM	41	4	135.3	490.1	325.0	145.8	337.2	278.8	383.4
DSLNT	G2	TERM	42	6	129.5	480.2	290.2	115.6	270.2	254.6	323.6
DSLNT	G2	TERM	43	5	105.1	332.1	232.7	97.88	224.7	175.1	326.4
DSLNT	G2	TERM	44	5	90.80	318.1	209.4	102.7	161.6	159.5	316.9
DSLNT	G2	TERM	45	5	48.35	312.0	177.5	124.1	150.1	74.88	302.1
DSLNT	G2	TERM	46	5	77.83	203.7	132.7	52.39	127.3	89.74	165.1
DSLNT	G2	TERM	47	4	69.77	224.3	148.5	69.14	149.9	103.9	194.5
DSLNT	G3	PRE-TERM	30	1	259.3	259.3	259.3	NA	259.3	259.3	259.3
DSLNT	G3	PRE-TERM	31	1	495.6	495.6	495.6	NA	495.6	495.6	495.6
DSLNT	G3	PRE-TERM	32	1	470.6	470.6	470.6	NA	470.6	470.6	470.6
DSLNT	G3	PRE-TERM	33	1	304.9	304.9	304.9	NA	304.9	304.9	304.9
DSLNT	G3	PRE-TERM	34	1	244.0	244.0	244.0	NA	244.0	244.0	244.0
DSLNT	G3	PRE-TERM	35	1	196.7	196.7	196.7	NA	196.7	196.7	196.7
DSLNT	G3	PRE-TERM	36	1	159.2	159.2	159.2	NA	159.2	159.2	159.2
DSLNT	G3	PRE-TERM	37	1	129.6	129.6	129.6	NA	129.6	129.6	129.6
DSLNT	G3	PRE-TERM	39	1	102.6	102.6	102.6	NA	102.6	102.6	102.6
DSLNT	G3	PRE-TERM	41	1	96.95	96.95	96.95	NA	96.95	96.95	96.95

Table S4 Concentration of Human Milk Oligosaccharides in Term or Preterm Milk At Specified Postmenstrual Age Separated By Milk Group

** When there are results below the method limit of quanfification (LoQ) the result has been assigned value of 0.5 × LoQ, hence the minimum value appears to be the same in many cases. When a large number of datapoints are below LoQ this can also have the effect that the median = minimum.*

					HMO Concentration (mg/L)						
HMO	Milk Group	Study Arm	Postmenstrual Age (weeks)	N	min *	max	mean	sd	median *	Quartile 1 (25%)	Quartile 3 (75%)
DSLNT	G3	PRE-TERM	43	1	97.17	97.17	97.17	NA	97.17	97.17	97.17
DSLNT	G3	PRE-TERM	45	1	97.08	97.08	97.08	NA	97.08	97.08	97.08
DSLNT	G3	TERM	41	1	163.6	163.6	163.6	NA	163.6	163.6	163.6
DSLNT	G3	TERM	42	1	190.7	190.7	190.7	NA	190.7	190.7	190.7
DSLNT	G3	TERM	43	1	218.6	218.6	218.6	NA	218.6	218.6	218.6
DSLNT	G3	TERM	44	1	199.4	199.4	199.4	NA	199.4	199.4	199.4
DSLNT	G3	TERM	45	1	171.3	171.3	171.3	NA	171.3	171.3	171.3
DSLNT	G3	TERM	46	1	156.0	156.0	156.0	NA	156.0	156.0	156.0
DSLNT	G3	TERM	47	1	129.3	129.3	129.3	NA	129.3	129.3	129.3
DSLNT	G3	TERM	48	1	140.2	140.2	140.2	NA	140.2	140.2	140.2
DSLNT	G4	TERM	40	1	697.9	697.9	697.9	NA	697.9	697.9	697.9
DSLNT	G4	TERM	41	1	608.1	608.1	608.1	NA	608.1	608.1	608.1
DSLNT	G4	TERM	42	1	488.2	488.2	488.2	NA	488.2	488.2	488.2
DSLNT	G4	TERM	43	1	477.6	477.6	477.6	NA	477.6	477.6	477.6
DSLNT	G4	TERM	44	1	360.7	360.7	360.7	NA	360.7	360.7	360.7
DSLNT	G4	TERM	45	1	401.1	401.1	401.1	NA	401.1	401.1	401.1
DSLNT	G4	TERM	46	1	293.1	293.1	293.1	NA	293.1	293.1	293.1
DSLNT	G4	TERM	47	1	264.6	264.6	264.6	NA	264.6	264.6	264.6
LDFT	G1	PRE-TERM	30	7	22.50	546.1	354.1	209.6	456.4	200.6	526.2
LDFT	G1	PRE-TERM	31	9	22.50	759.0	278.0	244.4	162.6	145.7	434.6
LDFT	G1	PRE-TERM	32	13	22.50	1243	324.1	325.6	223.4	146.1	321.0
LDFT	G1	PRE-TERM	33	18	22.50	864.6	305.7	244.0	230.9	158.0	423.5
LDFT	G1	PRE-TERM	34	19	22.50	2513	383.0	571.8	234.9	120.3	326.5
LDFT	G1	PRE-TERM	35	18	22.50	1926	367.2	417.7	231.6	186.8	364.8
LDFT	G1	PRE-TERM	36	19	22.50	604.5	230.3	139.3	212.7	142.9	296.8
LDFT	G1	PRE-TERM	37	18	22.50	910.9	286.2	197.8	249.6	171.5	336.4
LDFT	G1	PRE-TERM	38	11	176.6	2333	609.8	621.1	427.8	261.0	623.9
LDFT	G1	PRE-TERM	39	16	22.50	813.5	324.4	239.0	238.5	181.5	443.4
LDFT	G1	PRE-TERM	40	7	123.7	2758	656.1	938.6	323.3	206.8	487.3
LDFT	G1	PRE-TERM	41	12	22.50	3003	896.7	935.4	469.3	313.7	1347
LDFT	G1	PRE-TERM	42	6	142.7	677.0	304.3	193.3	227.6	213.7	313.3
LDFT	G1	PRE-TERM	43	10	22.50	1827	589.5	658.9	367.9	225.3	479.3
LDFT	G1	PRE-TERM	44	5	105.5	574.3	333.0	176.1	366.3	232.7	386.2
LDFT	G1	PRE-TERM	45	9	22.50	1825	557.6	588.2	333.4	254.3	574.9
LDFT	G1	PRE-TERM	46	6	231.6	791.9	377.3	213.2	284.5	260.3	385.4
LDFT	G1	PRE-TERM	47	3	142.6	499.2	327.3	178.6	340.0	241.3	419.6

Table S4 Concentration of Human Milk Oligosaccharides in Term or Preterm Milk At Specified Postmenstrual Age Separated By Milk Group

** When there are results below the method limit of quanfification (LoQ) the result has been assigned value of 0.5 × LoQ, hence the minimum value appears to be the same in many cases. When a large number of datapoints are below LoQ this can also have the effect that the median = minimum.*

					HMO Concentration (mg/L)						
**HMO**	**Milk Group**	**Study Arm**	**Postmenstrual Age (weeks)**	**N**	**min ***	**max**	**mean**	**sd**	**median ***	**Quartile 1 (25%)**	**Quartile 3 (75%)**
LDFT	G1	PRE-TERM	48	4	150.8	516.9	350.0	164.6	366.3	251.3	465.0
LDFT	G1	TERM	38	2	390.4	617.8	504.1	160.8	504.1	447.3	561.0
LDFT	G1	TERM	39	9	111.8	983.9	543.3	297.4	521.9	287.5	774.1
LDFT	G1	TERM	40	13	76.98	810.2	427.1	219.6	403.0	325.0	499.6
LDFT	G1	TERM	41	21	89.15	965.0	309.5	179.2	318.3	222.1	347.9
LDFT	G1	TERM	42	21	109.1	549.3	253.0	103.3	238.7	205.1	315.4
LDFT	G1	TERM	43	21	130.3	1270	321.0	280.1	210.4	189.5	302.0
LDFT	G1	TERM	44	20	105.9	2907	374.0	602.2	260.7	182.1	321.8
LDFT	G1	TERM	45	21	126.0	1713	362.0	368.6	250.2	189.9	305.4
LDFT	G1	TERM	46	17	118.3	566.9	295.9	123.0	322.8	203.2	341.6
LDFT	G1	TERM	47	12	93.06	429.7	282.2	104.0	293.3	216.6	352.0
LDFT	G1	TERM	48	6	174.4	818.6	423.8	230.1	357.7	295.0	509.7
LDFT	G2	PRE-TERM	29	1	22.50	22.50	22.50	NA	22.50	22.50	22.50
LDFT	G2	PRE-TERM	30	2	22.50	22.50	22.50	0.0000	22.50	22.50	22.50
LDFT	G2	PRE-TERM	31	2	22.50	22.50	22.50	0.0000	22.50	22.50	22.50
LDFT	G2	PRE-TERM	32	3	22.50	22.50	22.50	0.0000	22.50	22.50	22.50
LDFT	G2	PRE-TERM	33	5	22.50	22.50	22.50	0.0000	22.50	22.50	22.50
LDFT	G2	PRE-TERM	34	5	22.50	22.50	22.50	0.0000	22.50	22.50	22.50
LDFT	G2	PRE-TERM	35	5	22.50	22.50	22.50	0.0000	22.50	22.50	22.50
LDFT	G2	PRE-TERM	36	5	22.50	22.50	22.50	0.0000	22.50	22.50	22.50
LDFT	G2	PRE-TERM	37	4	22.50	22.50	22.50	0.0000	22.50	22.50	22.50
LDFT	G2	PRE-TERM	38	4	22.50	22.50	22.50	0.0000	22.50	22.50	22.50
LDFT	G2	PRE-TERM	39	3	22.50	22.50	22.50	0.0000	22.50	22.50	22.50
LDFT	G2	PRE-TERM	40	3	22.50	22.50	22.50	0.0000	22.50	22.50	22.50
LDFT	G2	PRE-TERM	41	2	22.50	22.50	22.50	0.0000	22.50	22.50	22.50
LDFT	G2	PRE-TERM	42	3	22.50	22.50	22.50	0.0000	22.50	22.50	22.50
LDFT	G2	PRE-TERM	43	2	22.50	22.50	22.50	0.0000	22.50	22.50	22.50
LDFT	G2	PRE-TERM	44	3	22.50	22.50	22.50	0.0000	22.50	22.50	22.50
LDFT	G2	PRE-TERM	45	2	22.50	22.50	22.50	0.0000	22.50	22.50	22.50
LDFT	G2	PRE-TERM	46	2	22.50	22.50	22.50	0.0000	22.50	22.50	22.50
LDFT	G2	PRE-TERM	48	1	22.50	22.50	22.50	NA	22.50	22.50	22.50
LDFT	G2	TERM	39	1	22.50	22.50	22.50	NA	22.50	22.50	22.50
LDFT	G2	TERM	40	5	22.50	22.50	22.50	0.0000	22.50	22.50	22.50
LDFT	G2	TERM	41	4	22.50	22.50	22.50	0.0000	22.50	22.50	22.50
LDFT	G2	TERM	42	6	22.50	22.50	22.50	0.0000	22.50	22.50	22.50
LDFT	G2	TERM	43	5	22.50	22.50	22.50	0.0000	22.50	22.50	22.50

Table S4 Concentration of Human Milk Oligosaccharides in Term or Preterm Milk At Specified Postmenstrual Age Separated By Milk Group

** When there are results below the method limit of quanfification (LoQ) the result has been assigned value of 0.5 × LoQ, hence the minimum value appears to be the same in many cases. When a large number of datapoints are below LoQ this can also have the effect that the median = minimum.*

HMO	Milk Group	Study Arm	Postmenstrual Age (weeks)	N	HMO Concentration (mg/L) min *	max	mean	sd	median *	Quartile 1 (25%)	Quartile 3 (75%)
LDFT	G2	TERM	44	5	22.50	22.50	22.50	0.0000	22.50	22.50	22.50
LDFT	G2	TERM	45	5	22.50	22.50	22.50	0.0000	22.50	22.50	22.50
LDFT	G2	TERM	46	5	22.50	22.50	22.50	0.0000	22.50	22.50	22.50
LDFT	G2	TERM	47	4	22.50	22.50	22.50	0.0000	22.50	22.50	22.50
LDFT	G3	PRE-TERM	30	1	56.66	56.66	56.66	NA	56.66	56.66	56.66
LDFT	G3	PRE-TERM	31	1	265.3	265.3	265.3	NA	265.3	265.3	265.3
LDFT	G3	PRE-TERM	32	1	135.2	135.2	135.2	NA	135.2	135.2	135.2
LDFT	G3	PRE-TERM	33	1	63.09	63.09	63.09	NA	63.09	63.09	63.09
LDFT	G3	PRE-TERM	34	1	91.93	91.93	91.93	NA	91.93	91.93	91.93
LDFT	G3	PRE-TERM	35	1	95.15	95.15	95.15	NA	95.15	95.15	95.15
LDFT	G3	PRE-TERM	36	1	77.50	77.50	77.50	NA	77.50	77.50	77.50
LDFT	G3	PRE-TERM	37	1	77.28	77.28	77.28	NA	77.28	77.28	77.28
LDFT	G3	PRE-TERM	39	1	76.44	76.44	76.44	NA	76.44	76.44	76.44
LDFT	G3	PRE-TERM	41	1	76.41	76.41	76.41	NA	76.41	76.41	76.41
LDFT	G3	PRE-TERM	43	1	91.69	91.69	91.69	NA	91.69	91.69	91.69
LDFT	G3	PRE-TERM	45	1	103.7	103.7	103.7	NA	103.7	103.7	103.7
LDFT	G3	TERM	41	1	71.16	71.16	71.16	NA	71.16	71.16	71.16
LDFT	G3	TERM	42	1	273.5	273.5	273.5	NA	273.5	273.5	273.5
LDFT	G3	TERM	43	1	136.6	136.6	136.6	NA	136.6	136.6	136.6
LDFT	G3	TERM	44	1	119.3	119.3	119.3	NA	119.3	119.3	119.3
LDFT	G3	TERM	45	1	61.93	61.93	61.93	NA	61.93	61.93	61.93
LDFT	G3	TERM	46	1	111.5	111.5	111.5	NA	111.5	111.5	111.5
LDFT	G3	TERM	47	1	68.95	68.95	68.95	NA	68.95	68.95	68.95
LDFT	G3	TERM	48	1	143.8	143.8	143.8	NA	143.8	143.8	143.8
LDFT	G4	TERM	40	1	22.50	22.50	22.50	NA	22.50	22.50	22.50
LDFT	G4	TERM	41	1	22.50	22.50	22.50	NA	22.50	22.50	22.50
LDFT	G4	TERM	42	1	22.50	22.50	22.50	NA	22.50	22.50	22.50
LDFT	G4	TERM	43	1	22.50	22.50	22.50	NA	22.50	22.50	22.50
LDFT	G4	TERM	44	1	22.50	22.50	22.50	NA	22.50	22.50	22.50
LDFT	G4	TERM	45	1	22.50	22.50	22.50	NA	22.50	22.50	22.50
LDFT	G4	TERM	46	1	22.50	22.50	22.50	NA	22.50	22.50	22.50
LDFT	G4	TERM	47	1	22.50	22.50	22.50	NA	22.50	22.50	22.50
LNDFH-I	G1	PRE-TERM	30	7	81.36	1679	1048	511.6	1039	963.9	1303
LNDFH-I	G1	PRE-TERM	31	9	75.44	1761	1031	509.3	951.6	780.5	1263
LNDFH-I	G1	PRE-TERM	32	13	49.54	1781	1110	504.0	1145	767.7	1520
LNDFH-I	G1	PRE-TERM	33	18	40.53	1860	1165	462.0	1186	888.5	1529

Table S4 Concentration of Human Milk Oligosaccharides in Term or Preterm Milk At Specified Postmenstrual Age Separated By Milk Group

** When there are results below the method limit of quanfification (LoQ) the result has been assigned value of 0.5 × LoQ, hence the minimum value appears to be the same in many cases. When a large number of datapoints are below LoQ this can also have the effect that the median = minimum.*

					HMO Concentration (mg/L)						
HMO	Milk Group	Study Arm	Postmenstrual Age (weeks)	N	min *	max	mean	sd	median *	Quartile 1 (25%)	Quartile 3 (75%)
LNDFH-I	G1	PRE-TERM	34	19	41.75	3123	1196	640.2	1145	831.0	1487
LNDFH-I	G1	PRE-TERM	35	18	39.25	2269	1158	464.1	1143	947.1	1427
LNDFH-I	G1	PRE-TERM	36	19	36.61	1707	1028	402.3	1022	844.3	1275
LNDFH-I	G1	PRE-TERM	37	18	31.86	1585	991.3	366.6	1031	840.8	1187
LNDFH-I	G1	PRE-TERM	38	11	563.5	1910	1100	434.2	859.0	821.8	1475
LNDFH-I	G1	PRE-TERM	39	16	22.31	1661	977.8	373.2	969.1	783.0	1126
LNDFH-I	G1	PRE-TERM	40	7	753.6	1575	1141	348.1	1170	839.2	1404
LNDFH-I	G1	PRE-TERM	41	12	18.66	1551	1033	412.1	1066	867.2	1374
LNDFH-I	G1	PRE-TERM	42	6	608.0	1606	998.3	392.3	957.9	666.5	1207
LNDFH-I	G1	PRE-TERM	43	10	33.59	1768	1045	527.4	1020	735.7	1467
LNDFH-I	G1	PRE-TERM	44	5	623.9	1182	919.1	210.8	883.0	858.5	1048
LNDFH-I	G1	PRE-TERM	45	9	34.15	1249	798.7	354.9	859.1	630.9	1001
LNDFH-I	G1	PRE-TERM	46	6	574.6	1431	915.3	320.6	866.4	682.7	1065
LNDFH-I	G1	PRE-TERM	47	3	578.1	1327	833.6	427.2	595.8	587.0	961.3
LNDFH-I	G1	PRE-TERM	48	4	530.1	967.5	693.6	192.1	638.3	582.8	749.1
LNDFH-I	G1	TERM	38	2	1301	1689	1495	274.3	1495	1398	1592
LNDFH-I	G1	TERM	39	9	846.1	1755	1228	347.3	1197	947.1	1445
LNDFH-I	G1	TERM	40	13	825.9	2122	1433	424.7	1542	1015	1673
LNDFH-I	G1	TERM	41	21	827.5	2264	1316	393.0	1200	1044	1479
LNDFH-I	G1	TERM	42	21	816.4	2313	1308	403.8	1252	1067	1380
LNDFH-I	G1	TERM	43	21	692.6	1937	1194	368.2	1131	951.4	1431
LNDFH-I	G1	TERM	44	20	464.2	1983	1096	432.3	1123	786.6	1301
LNDFH-I	G1	TERM	45	21	548.4	2024	1050	381.6	953.3	800.7	1239
LNDFH-I	G1	TERM	46	17	458.7	2167	1002	454.9	921.7	649.6	1305
LNDFH-I	G1	TERM	47	12	5.000	1935	958.9	587.6	992.5	467.8	1249
LNDFH-I	G1	TERM	48	6	441.2	1304	883.1	356.4	956.7	578.2	1130
LNDFH-I	G2	PRE-TERM	29	1	115.7	115.7	115.7	NA	115.7	115.7	115.7
LNDFH-I	G2	PRE-TERM	30	2	5.000	65.99	35.49	43.13	35.49	20.25	50.74
LNDFH-I	G2	PRE-TERM	31	2	5.000	56.94	30.97	36.73	30.97	17.99	43.96
LNDFH-I	G2	PRE-TERM	32	3	5.000	22.95	10.98	10.36	5.000	5.000	13.97
LNDFH-I	G2	PRE-TERM	33	5	5.000	24.70	8.940	8.810	5.000	5.000	5.000
LNDFH-I	G2	PRE-TERM	34	5	5.000	22.52	8.505	7.836	5.000	5.000	5.000
LNDFH-I	G2	PRE-TERM	35	5	5.000	22.32	8.464	7.746	5.000	5.000	5.000
LNDFH-I	G2	PRE-TERM	36	5	5.000	21.68	8.336	7.460	5.000	5.000	5.000
LNDFH-I	G2	PRE-TERM	37	4	5.000	5.000	5.000	0.0000	5.000	5.000	5.000
LNDFH-I	G2	PRE-TERM	38	4	5.000	15.92	7.731	5.462	5.000	5.000	7.731

Table S4 Concentration of Human Milk Oligosaccharides in Term or Preterm Milk At Specified Postmenstrual Age Separated By Milk Group

** When there are results below the method limit of quanfification (LoQ) the result has been assigned value of 0.5 × LoQ, hence the minimum value appears to be the same in many cases. When a large number of datapoints are below LoQ this can also have the effect that the median = minimum.*

					HMO Concentration (mg/L)						
HMO	Milk Group	Study Arm	Postmenstrual Age (weeks)	N	min *	max	mean	sd	median *	Quartile 1 (25%)	Quartile 3 (75%)
LNDFH-I	G2	PRE-TERM	39	3	5.000	5.000	5.000	0.0000	5.000	5.000	5.000
LNDFH-I	G2	PRE-TERM	40	3	5.000	14.75	8.249	5.627	5.000	5.000	9.874
LNDFH-I	G2	PRE-TERM	41	2	5.000	5.000	5.000	0.0000	5.000	5.000	5.000
LNDFH-I	G2	PRE-TERM	42	3	5.000	14.03	8.011	5.214	5.000	5.000	9.516
LNDFH-I	G2	PRE-TERM	43	2	5.000	5.000	5.000	0.0000	5.000	5.000	5.000
LNDFH-I	G2	PRE-TERM	44	3	5.000	11.93	7.311	4.002	5.000	5.000	8.466
LNDFH-I	G2	PRE-TERM	45	2	5.000	5.000	5.000	0.0000	5.000	5.000	5.000
LNDFH-I	G2	PRE-TERM	46	2	5.000	5.000	5.000	0.0000	5.000	5.000	5.000
LNDFH-I	G2	PRE-TERM	48	1	5.000	5.000	5.000	NA	5.000	5.000	5.000
LNDFH-I	G2	TERM	39	1	5.000	5.000	5.000	NA	5.000	5.000	5.000
LNDFH-I	G2	TERM	40	5	5.000	5.000	5.000	0.0000	5.000	5.000	5.000
LNDFH-I	G2	TERM	41	4	5.000	5.000	5.000	0.0000	5.000	5.000	5.000
LNDFH-I	G2	TERM	42	6	5.000	5.000	5.000	0.0000	5.000	5.000	5.000
LNDFH-I	G2	TERM	43	5	5.000	15.85	7.171	4.853	5.000	5.000	5.000
LNDFH-I	G2	TERM	44	5	5.000	11.89	6.378	3.082	5.000	5.000	5.000
LNDFH-I	G2	TERM	45	5	5.000	5.000	5.000	0.0000	5.000	5.000	5.000
LNDFH-I	G2	TERM	46	5	5.000	5.000	5.000	0.0000	5.000	5.000	5.000
LNDFH-I	G2	TERM	47	4	5.000	5.000	5.000	0.0000	5.000	5.000	5.000
LNDFH-I	G3	PRE-TERM	30	1	5.000	5.000	5.000	NA	5.000	5.000	5.000
LNDFH-I	G3	PRE-TERM	31	1	5.000	5.000	5.000	NA	5.000	5.000	5.000
LNDFH-I	G3	PRE-TERM	32	1	5.000	5.000	5.000	NA	5.000	5.000	5.000
LNDFH-I	G3	PRE-TERM	33	1	5.000	5.000	5.000	NA	5.000	5.000	5.000
LNDFH-I	G3	PRE-TERM	34	1	5.000	5.000	5.000	NA	5.000	5.000	5.000
LNDFH-I	G3	PRE-TERM	35	1	5.000	5.000	5.000	NA	5.000	5.000	5.000
LNDFH-I	G3	PRE-TERM	36	1	5.000	5.000	5.000	NA	5.000	5.000	5.000
LNDFH-I	G3	PRE-TERM	37	1	5.000	5.000	5.000	NA	5.000	5.000	5.000
LNDFH-I	G3	PRE-TERM	39	1	5.000	5.000	5.000	NA	5.000	5.000	5.000
LNDFH-I	G3	PRE-TERM	41	1	5.000	5.000	5.000	NA	5.000	5.000	5.000
LNDFH-I	G3	PRE-TERM	43	1	5.000	5.000	5.000	NA	5.000	5.000	5.000
LNDFH-I	G3	PRE-TERM	45	1	5.000	5.000	5.000	NA	5.000	5.000	5.000
LNDFH-I	G3	TERM	41	1	5.000	5.000	5.000	NA	5.000	5.000	5.000
LNDFH-I	G3	TERM	42	1	5.000	5.000	5.000	NA	5.000	5.000	5.000
LNDFH-I	G3	TERM	43	1	5.000	5.000	5.000	NA	5.000	5.000	5.000
LNDFH-I	G3	TERM	44	1	5.000	5.000	5.000	NA	5.000	5.000	5.000
LNDFH-I	G3	TERM	45	1	5.000	5.000	5.000	NA	5.000	5.000	5.000
LNDFH-I	G3	TERM	46	1	5.000	5.000	5.000	NA	5.000	5.000	5.000

Table S4 Concentration of Human Milk Oligosaccharides in Term or Preterm Milk At Specified Postmenstrual Age Separated By Milk Group

** When there are results below the method limit of quanfification (LoQ) the result has been assigned value of 0.5 × LoQ, hence the minimum value appears to be the same in many cases. When a large number of datapoints are below LoQ this can also have the effect that the median = minimum.*

HMO	Milk Group	Study Arm	Postmenstrual Age (weeks)	N	HMO Concentration (mg/L) min *	max	mean	sd	median *	Quartile 1 (25%)	Quartile 3 (75%)
LNDFH-I	G3	TERM	47	1	5.000	5.000	5.000	NA	5.000	5.000	5.000
LNDFH-I	G3	TERM	48	1	5.000	5.000	5.000	NA	5.000	5.000	5.000
LNDFH-I	G4	TERM	40	1	5.000	5.000	5.000	NA	5.000	5.000	5.000
LNDFH-I	G4	TERM	41	1	5.000	5.000	5.000	NA	5.000	5.000	5.000
LNDFH-I	G4	TERM	42	1	5.000	5.000	5.000	NA	5.000	5.000	5.000
LNDFH-I	G4	TERM	43	1	5.000	5.000	5.000	NA	5.000	5.000	5.000
LNDFH-I	G4	TERM	44	1	5.000	5.000	5.000	NA	5.000	5.000	5.000
LNDFH-I	G4	TERM	45	1	5.000	5.000	5.000	NA	5.000	5.000	5.000
LNDFH-I	G4	TERM	46	1	5.000	5.000	5.000	NA	5.000	5.000	5.000
LNDFH-I	G4	TERM	47	1	5.000	5.000	5.000	NA	5.000	5.000	5.000
LNFP-I	G1	PRE-TERM	30	7	13.50	2005	1411	739.8	1712	1217	1857
LNFP-I	G1	PRE-TERM	31	9	13.50	2630	1321	792.2	1413	956.9	1493
LNFP-I	G1	PRE-TERM	32	13	13.50	2806	1291	691.1	1232	876.2	1448
LNFP-I	G1	PRE-TERM	33	18	13.50	2027	1172	465.4	1098	964.7	1439
LNFP-I	G1	PRE-TERM	34	19	13.50	1634	1004	414.2	978.5	745.0	1359
LNFP-I	G1	PRE-TERM	35	18	13.50	1840	902.1	401.3	823.9	708.6	1055
LNFP-I	G1	PRE-TERM	36	19	13.50	1773	859.4	425.2	673.9	606.6	1122
LNFP-I	G1	PRE-TERM	37	18	13.50	1188	716.2	322.2	677.8	492.4	952.2
LNFP-I	G1	PRE-TERM	38	11	301.9	1126	621.2	296.6	596.0	391.5	746.2
LNFP-I	G1	PRE-TERM	39	16	13.50	1436	618.7	331.9	538.0	441.7	765.7
LNFP-I	G1	PRE-TERM	40	7	349.8	1075	639.0	281.0	490.3	429.0	850.0
LNFP-I	G1	PRE-TERM	41	12	13.50	789.6	477.1	244.6	504.2	349.9	613.8
LNFP-I	G1	PRE-TERM	42	6	259.1	966.6	541.5	269.2	528.7	328.3	660.6
LNFP-I	G1	PRE-TERM	43	10	13.50	1014	546.9	314.6	556.2	332.0	793.2
LNFP-I	G1	PRE-TERM	44	5	259.0	549.4	417.6	108.1	404.8	396.8	477.8
LNFP-I	G1	PRE-TERM	45	9	13.50	933.6	386.1	305.2	327.9	177.2	604.8
LNFP-I	G1	PRE-TERM	46	6	131.5	717.8	400.9	199.4	408.4	293.8	464.7
LNFP-I	G1	PRE-TERM	47	3	195.2	635.0	376.5	229.8	299.3	247.3	467.2
LNFP-I	G1	PRE-TERM	48	4	142.5	377.1	240.0	107.5	220.2	161.1	299.0
LNFP-I	G1	TERM	38	2	1760	1768	1764	6.024	1764	1762	1766
LNFP-I	G1	TERM	39	9	685.4	2547	1685	731.5	1998	1026	2287
LNFP-I	G1	TERM	40	13	505.8	3381	1825	837.9	1581	1228	2247
LNFP-I	G1	TERM	41	21	492.7	3597	1909	834.5	1740	1388	2489
LNFP-I	G1	TERM	42	21	418.7	3156	1560	690.6	1318	1198	1841
LNFP-I	G1	TERM	43	21	290.2	2655	1233	674.8	1007	831.7	1715
LNFP-I	G1	TERM	44	20	231.4	2647	1012	622.9	814.4	621.9	1273

Table S4 Concentration of Human Milk Oligosaccharides in Term or Preterm Milk At Specified Postmenstrual Age Separated By Milk Group

** When there are results below the method limit of quanfification (LoQ) the result has been assigned value of 0.5 × LoQ, hence the minimum value appears to be the same in many cases. When a large number of datapoints are below LoQ this can also have the effect that the median = minimum.*

					HMO Concentration (mg/L)						
HMO	Milk Group	Study Arm	Postmenstrual Age (weeks)	N	min *	max	mean	sd	median *	Quartile 1 (25%)	Quartile 3 (75%)
LNFP-I	G1	TERM	45	21	216.7	1899	824.8	419.6	760.3	567.3	1004
LNFP-I	G1	TERM	46	17	192.0	1956	730.2	449.1	656.2	469.8	833.5
LNFP-I	G1	TERM	47	12	168.8	2617	850.9	613.4	790.9	640.8	853.1
LNFP-I	G1	TERM	48	6	192.3	1715	796.3	535.5	640.3	522.5	989.9
LNFP-I	G2	PRE-TERM	29	1	13.50	13.50	13.50	NA	13.50	13.50	13.50
LNFP-I	G2	PRE-TERM	30	2	13.50	13.50	13.50	0.0000	13.50	13.50	13.50
LNFP-I	G2	PRE-TERM	31	2	13.50	13.50	13.50	0.0000	13.50	13.50	13.50
LNFP-I	G2	PRE-TERM	32	3	13.50	13.50	13.50	0.0000	13.50	13.50	13.50
LNFP-I	G2	PRE-TERM	33	5	13.50	13.50	13.50	0.0000	13.50	13.50	13.50
LNFP-I	G2	PRE-TERM	34	5	13.50	13.50	13.50	0.0000	13.50	13.50	13.50
LNFP-I	G2	PRE-TERM	35	5	13.50	13.50	13.50	0.0000	13.50	13.50	13.50
LNFP-I	G2	PRE-TERM	36	5	13.50	13.50	13.50	0.0000	13.50	13.50	13.50
LNFP-I	G2	PRE-TERM	37	4	13.50	13.50	13.50	0.0000	13.50	13.50	13.50
LNFP-I	G2	PRE-TERM	38	4	13.50	13.50	13.50	0.0000	13.50	13.50	13.50
LNFP-I	G2	PRE-TERM	39	3	13.50	13.50	13.50	0.0000	13.50	13.50	13.50
LNFP-I	G2	PRE-TERM	40	3	13.50	13.50	13.50	0.0000	13.50	13.50	13.50
LNFP-I	G2	PRE-TERM	41	2	13.50	13.50	13.50	0.0000	13.50	13.50	13.50
LNFP-I	G2	PRE-TERM	42	3	13.50	13.50	13.50	0.0000	13.50	13.50	13.50
LNFP-I	G2	PRE-TERM	43	2	13.50	13.50	13.50	0.0000	13.50	13.50	13.50
LNFP-I	G2	PRE-TERM	44	3	13.50	13.50	13.50	0.0000	13.50	13.50	13.50
LNFP-I	G2	PRE-TERM	45	2	13.50	13.50	13.50	0.0000	13.50	13.50	13.50
LNFP-I	G2	PRE-TERM	46	2	13.50	13.50	13.50	0.0000	13.50	13.50	13.50
LNFP-I	G2	PRE-TERM	48	1	13.50	13.50	13.50	NA	13.50	13.50	13.50
LNFP-I	G2	TERM	39	1	13.50	13.50	13.50	NA	13.50	13.50	13.50
LNFP-I	G2	TERM	40	5	13.50	13.50	13.50	0.0000	13.50	13.50	13.50
LNFP-I	G2	TERM	41	4	13.50	13.50	13.50	0.0000	13.50	13.50	13.50
LNFP-I	G2	TERM	42	6	13.50	13.50	13.50	0.0000	13.50	13.50	13.50
LNFP-I	G2	TERM	43	5	13.50	13.50	13.50	0.0000	13.50	13.50	13.50
LNFP-I	G2	TERM	44	5	13.50	13.50	13.50	0.0000	13.50	13.50	13.50
LNFP-I	G2	TERM	45	5	13.50	13.50	13.50	0.0000	13.50	13.50	13.50
LNFP-I	G2	TERM	46	5	13.50	13.50	13.50	0.0000	13.50	13.50	13.50
LNFP-I	G2	TERM	47	4	13.50	13.50	13.50	0.0000	13.50	13.50	13.50
LNFP-I	G3	PRE-TERM	30	1	2639	2639	2639	NA	2639	2639	2639
LNFP-I	G3	PRE-TERM	31	1	2195	2195	2195	NA	2195	2195	2195
LNFP-I	G3	PRE-TERM	32	1	2178	2178	2178	NA	2178	2178	2178
LNFP-I	G3	PRE-TERM	33	1	2062	2062	2062	NA	2062	2062	2062

Table S4 Concentration of Human Milk Oligosaccharides in Term or Preterm Milk At Specified Postmenstrual Age Separated By Milk Group

** When there are results below the method limit of quanfification (LoQ) the result has been assigned value of 0.5 × LoQ, hence the minimum value appears to be the same in many cases. When a large number of datapoints are below LoQ this can also have the effect that the median = minimum.*

					HMO Concentration (mg/L)						
HMO	Milk Group	Study Arm	Postmenstrual Age (weeks)	N	min *	max	mean	sd	median *	Quartile 1 (25%)	Quartile 3 (75%)
LNFP-I	G3	PRE-TERM	34	1	1624	1624	1624	NA	1624	1624	1624
LNFP-I	G3	PRE-TERM	35	1	1332	1332	1332	NA	1332	1332	1332
LNFP-I	G3	PRE-TERM	36	1	949.2	949.2	949.2	NA	949.2	949.2	949.2
LNFP-I	G3	PRE-TERM	37	1	960.9	960.9	960.9	NA	960.9	960.9	960.9
LNFP-I	G3	PRE-TERM	39	1	807.3	807.3	807.3	NA	807.3	807.3	807.3
LNFP-I	G3	PRE-TERM	41	1	829.3	829.3	829.3	NA	829.3	829.3	829.3
LNFP-I	G3	PRE-TERM	43	1	829.0	829.0	829.0	NA	829.0	829.0	829.0
LNFP-I	G3	PRE-TERM	45	1	784.9	784.9	784.9	NA	784.9	784.9	784.9
LNFP-I	G3	TERM	41	1	2392	2392	2392	NA	2392	2392	2392
LNFP-I	G3	TERM	42	1	1768	1768	1768	NA	1768	1768	1768
LNFP-I	G3	TERM	43	1	2027	2027	2027	NA	2027	2027	2027
LNFP-I	G3	TERM	44	1	1812	1812	1812	NA	1812	1812	1812
LNFP-I	G3	TERM	45	1	1850	1850	1850	NA	1850	1850	1850
LNFP-I	G3	TERM	46	1	1480	1480	1480	NA	1480	1480	1480
LNFP-I	G3	TERM	47	1	1320	1320	1320	NA	1320	1320	1320
LNFP-I	G3	TERM	48	1	1180	1180	1180	NA	1180	1180	1180
LNFP-I	G4	TERM	40	1	13.50	13.50	13.50	NA	13.50	13.50	13.50
LNFP-I	G4	TERM	41	1	13.50	13.50	13.50	NA	13.50	13.50	13.50
LNFP-I	G4	TERM	42	1	13.50	13.50	13.50	NA	13.50	13.50	13.50
LNFP-I	G4	TERM	43	1	13.50	13.50	13.50	NA	13.50	13.50	13.50
LNFP-I	G4	TERM	44	1	13.50	13.50	13.50	NA	13.50	13.50	13.50
LNFP-I	G4	TERM	45	1	13.50	13.50	13.50	NA	13.50	13.50	13.50
LNFP-I	G4	TERM	46	1	13.50	13.50	13.50	NA	13.50	13.50	13.50
LNFP-I	G4	TERM	47	1	13.50	13.50	13.50	NA	13.50	13.50	13.50
LNFP-II	G1	PRE-TERM	30	7	115.2	1108	350.0	354.2	190.9	134.9	383.2
LNFP-II	G1	PRE-TERM	31	9	165.2	1108	391.0	293.7	287.7	226.1	478.5
LNFP-II	G1	PRE-TERM	32	13	124.0	975.6	351.4	258.8	230.7	164.5	422.1
LNFP-II	G1	PRE-TERM	33	18	95.46	918.9	349.9	218.2	281.4	192.2	522.5
LNFP-II	G1	PRE-TERM	34	19	136.4	865.6	402.3	238.1	321.2	189.2	566.3
LNFP-II	G1	PRE-TERM	35	18	122.1	946.4	415.3	233.4	358.4	260.4	521.4
LNFP-II	G1	PRE-TERM	36	19	141.8	982.3	412.4	243.3	389.4	205.8	524.8
LNFP-II	G1	PRE-TERM	37	18	121.7	891.0	404.5	225.9	402.5	231.9	511.6
LNFP-II	G1	PRE-TERM	38	11	68.91	849.5	320.8	248.2	230.0	143.4	443.4
LNFP-II	G1	PRE-TERM	39	16	146.0	858.2	415.8	255.0	331.1	205.1	590.1
LNFP-II	G1	PRE-TERM	40	7	149.3	911.4	451.7	317.8	255.0	206.7	716.4
LNFP-II	G1	PRE-TERM	41	12	92.35	633.7	331.0	177.1	287.0	204.3	445.8

Table S4 Concentration of Human Milk Oligosaccharides in Term or Preterm Milk At Specified Postmenstrual Age Separated By Milk Group

** When there are results below the method limit of quanfification (LoQ) the result has been assigned value of 0.5 × LoQ, hence the minimum value appears to be the same in many cases. When a large number of datapoints are below LoQ this can also have the effect that the median = minimum.*

					HMO Concentration (mg/L)						
HMO	Milk Group	Study Arm	Postmenstrual Age (weeks)	N	min *	max	mean	sd	median *	Quartile 1 (25%)	Quartile 3 (75%)
LNFP-II	G1	PRE-TERM	42	6	141.6	757.1	438.3	271.6	437.0	208.8	652.6
LNFP-II	G1	PRE-TERM	43	10	183.5	728.3	388.8	173.9	383.3	234.2	485.8
LNFP-II	G1	PRE-TERM	44	5	160.6	688.8	442.1	216.1	510.0	281.3	569.8
LNFP-II	G1	PRE-TERM	45	9	128.3	1014	384.7	255.2	369.5	297.0	395.8
LNFP-II	G1	PRE-TERM	46	6	121.9	754.0	406.5	255.9	406.9	191.7	573.8
LNFP-II	G1	PRE-TERM	47	3	168.9	386.9	311.6	123.6	378.9	273.9	382.9
LNFP-II	G1	PRE-TERM	48	4	118.0	654.3	380.8	236.2	375.5	229.8	526.5
LNFP-II	G1	TERM	38	2	222.1	251.5	236.8	20.83	236.8	229.4	244.2
LNFP-II	G1	TERM	39	9	85.02	421.9	215.0	119.8	161.8	127.1	269.3
LNFP-II	G1	TERM	40	13	55.63	596.3	308.5	138.5	268.6	231.0	347.7
LNFP-II	G1	TERM	41	21	112.6	1204	370.5	257.8	319.6	193.0	434.2
LNFP-II	G1	TERM	42	21	147.7	686.4	338.1	156.6	308.5	203.2	424.8
LNFP-II	G1	TERM	43	21	149.7	1531	397.2	306.6	328.3	201.6	479.6
LNFP-II	G1	TERM	44	20	127.0	718.3	312.9	160.0	250.7	202.3	395.3
LNFP-II	G1	TERM	45	21	137.6	824.7	329.4	185.1	312.4	186.1	371.0
LNFP-II	G1	TERM	46	17	96.47	846.5	339.1	217.1	289.8	173.9	429.2
LNFP-II	G1	TERM	47	12	126.2	854.5	330.3	244.5	215.1	152.9	403.3
LNFP-II	G1	TERM	48	6	112.3	382.2	214.4	90.56	198.4	179.0	216.6
LNFP-II	G2	PRE-TERM	29	1	2094	2094	2094	NA	2094	2094	2094
LNFP-II	G2	PRE-TERM	30	2	1328	1740	1534	291.4	1534	1431	1637
LNFP-II	G2	PRE-TERM	31	2	1514	1628	1571	80.82	1571	1543	1600
LNFP-II	G2	PRE-TERM	32	3	930.8	1775	1380	424.7	1435	1183	1605
LNFP-II	G2	PRE-TERM	33	5	897.4	1845	1503	374.6	1667	1402	1703
LNFP-II	G2	PRE-TERM	34	5	791.2	1950	1452	516.7	1736	1006	1775
LNFP-II	G2	PRE-TERM	35	5	774.2	2138	1495	603.1	1696	947.4	1922
LNFP-II	G2	PRE-TERM	36	5	730.6	2220	1541	651.4	1597	1044	2116
LNFP-II	G2	PRE-TERM	37	4	1023	1804	1493	333.2	1572	1408	1657
LNFP-II	G2	PRE-TERM	38	4	656.5	2024	1231	573.6	1121	973.6	1378
LNFP-II	G2	PRE-TERM	39	3	983.3	1942	1325	535.7	1050	1016	1496
LNFP-II	G2	PRE-TERM	40	3	630.5	1468	1046	418.9	1039	834.6	1253
LNFP-II	G2	PRE-TERM	41	2	1180	2373	1777	843.8	1777	1478	2075
LNFP-II	G2	PRE-TERM	42	3	601.3	1432	927.1	443.2	748.0	674.7	1090
LNFP-II	G2	PRE-TERM	43	2	643.6	1476	1060	588.8	1060	851.8	1268
LNFP-II	G2	PRE-TERM	44	3	561.9	1571	933.6	554.5	668.0	614.9	1119
LNFP-II	G2	PRE-TERM	45	2	784.2	2593	1689	1279	1689	1236	2141
LNFP-II	G2	PRE-TERM	46	2	483.6	1367	925.2	624.4	925.2	704.4	1146

Table S4 Concentration of Human Milk Oligosaccharides in Term or Preterm Milk At Specified Postmenstrual Age Separated By Milk Group

** When there are results below the method limit of quanfification (LoQ) the result has been assigned value of 0.5 × LoQ, hence the minimum value appears to be the same in many cases. When a large number of datapoints are below LoQ this can also have the effect that the median = minimum.*

					HMO Concentration (mg/L)						
HMO	Milk Group	Study Arm	Postmenstrual Age (weeks)	N	min *	max	mean	sd	median *	Quartile 1 (25%)	Quartile 3 (75%)
LNFP-II	G2	PRE-TERM	48	1	1201	1201	1201	NA	1201	1201	1201
LNFP-II	G2	TERM	39	1	1238	1238	1238	NA	1238	1238	1238
LNFP-II	G2	TERM	40	5	1069	1616	1301	204.7	1296	1186	1339
LNFP-II	G2	TERM	41	4	901.1	1884	1373	413.0	1353	1153	1572
LNFP-II	G2	TERM	42	6	884.6	1684	1463	311.0	1602	1393	1660
LNFP-II	G2	TERM	43	5	947.7	1565	1245	228.2	1268	1133	1310
LNFP-II	G2	TERM	44	5	964.1	1411	1150	188.4	1134	982.8	1257
LNFP-II	G2	TERM	45	5	376.0	1476	969.6	415.7	923.5	847.4	1225
LNFP-II	G2	TERM	46	5	530.4	1171	904.3	275.8	1038	697.1	1085
LNFP-II	G2	TERM	47	4	597.7	1272	916.0	346.9	897.4	629.5	1184
LNFP-II	G3	PRE-TERM	30	1	17.50	17.50	17.50	NA	17.50	17.50	17.50
LNFP-II	G3	PRE-TERM	31	1	17.50	17.50	17.50	NA	17.50	17.50	17.50
LNFP-II	G3	PRE-TERM	32	1	17.50	17.50	17.50	NA	17.50	17.50	17.50
LNFP-II	G3	PRE-TERM	33	1	17.50	17.50	17.50	NA	17.50	17.50	17.50
LNFP-II	G3	PRE-TERM	34	1	17.50	17.50	17.50	NA	17.50	17.50	17.50
LNFP-II	G3	PRE-TERM	35	1	17.50	17.50	17.50	NA	17.50	17.50	17.50
LNFP-II	G3	PRE-TERM	36	1	17.50	17.50	17.50	NA	17.50	17.50	17.50
LNFP-II	G3	PRE-TERM	37	1	17.50	17.50	17.50	NA	17.50	17.50	17.50
LNFP-II	G3	PRE-TERM	39	1	17.50	17.50	17.50	NA	17.50	17.50	17.50
LNFP-II	G3	PRE-TERM	41	1	17.50	17.50	17.50	NA	17.50	17.50	17.50
LNFP-II	G3	PRE-TERM	43	1	17.50	17.50	17.50	NA	17.50	17.50	17.50
LNFP-II	G3	PRE-TERM	45	1	17.50	17.50	17.50	NA	17.50	17.50	17.50
LNFP-II	G3	TERM	41	1	17.50	17.50	17.50	NA	17.50	17.50	17.50
LNFP-II	G3	TERM	42	1	17.50	17.50	17.50	NA	17.50	17.50	17.50
LNFP-II	G3	TERM	43	1	17.50	17.50	17.50	NA	17.50	17.50	17.50
LNFP-II	G3	TERM	44	1	17.50	17.50	17.50	NA	17.50	17.50	17.50
LNFP-II	G3	TERM	45	1	17.50	17.50	17.50	NA	17.50	17.50	17.50
LNFP-II	G3	TERM	46	1	17.50	17.50	17.50	NA	17.50	17.50	17.50
LNFP-II	G3	TERM	47	1	17.50	17.50	17.50	NA	17.50	17.50	17.50
LNFP-II	G3	TERM	48	1	17.50	17.50	17.50	NA	17.50	17.50	17.50
LNFP-II	G4	TERM	40	1	17.50	17.50	17.50	NA	17.50	17.50	17.50
LNFP-II	G4	TERM	41	1	17.50	17.50	17.50	NA	17.50	17.50	17.50
LNFP-II	G4	TERM	42	1	17.50	17.50	17.50	NA	17.50	17.50	17.50
LNFP-II	G4	TERM	43	1	17.50	17.50	17.50	NA	17.50	17.50	17.50
LNFP-II	G4	TERM	44	1	17.50	17.50	17.50	NA	17.50	17.50	17.50
LNFP-II	G4	TERM	45	1	17.50	17.50	17.50	NA	17.50	17.50	17.50

Table S4 Concentration of Human Milk Oligosaccharides in Term or Preterm Milk At Specified Postmenstrual Age Separated By Milk Group

** When there are results below the method limit of quanfification (LoQ) the result has been assigned value of 0.5 × LoQ, hence the minimum value appears to be the same in many cases. When a large number of datapoints are below LoQ this can also have the effect that the median = minimum.*

HMO	Milk Group	Study Arm	Postmenstrual Age (weeks)	N	HMO Concentration (mg/L) min *	max	mean	sd	median *	Quartile 1 (25%)	Quartile 3 (75%)
LNFP-II	G4	TERM	46	1	17.50	17.50	17.50	NA	17.50	17.50	17.50
LNFP-II	G4	TERM	47	1	17.50	17.50	17.50	NA	17.50	17.50	17.50
LNFP-III	G1	PRE-TERM	30	7	55.72	404.4	333.0	125.4	378.3	347.1	399.2
LNFP-III	G1	PRE-TERM	31	9	35.70	464.9	285.5	123.4	305.5	276.7	353.2
LNFP-III	G1	PRE-TERM	32	13	40.94	480.4	252.8	114.0	244.7	188.8	290.5
LNFP-III	G1	PRE-TERM	33	18	49.93	430.7	248.1	114.4	242.2	161.9	344.4
LNFP-III	G1	PRE-TERM	34	19	52.14	506.1	275.1	123.0	262.9	214.1	332.6
LNFP-III	G1	PRE-TERM	35	18	49.25	491.8	274.0	115.5	284.2	215.3	336.3
LNFP-III	G1	PRE-TERM	36	19	17.50	482.4	270.2	115.4	274.0	206.2	357.7
LNFP-III	G1	PRE-TERM	37	18	52.97	546.0	306.1	124.0	302.9	211.4	396.0
LNFP-III	G1	PRE-TERM	38	11	55.78	615.4	316.0	172.0	282.7	211.2	420.8
LNFP-III	G1	PRE-TERM	39	16	60.20	847.4	355.6	200.8	345.0	238.1	414.2
LNFP-III	G1	PRE-TERM	40	7	175.8	534.0	308.4	136.9	262.9	209.5	383.6
LNFP-III	G1	PRE-TERM	41	12	50.94	653.0	417.8	178.9	437.8	354.8	540.8
LNFP-III	G1	PRE-TERM	42	6	177.7	442.1	284.9	91.41	273.5	233.4	310.3
LNFP-III	G1	PRE-TERM	43	10	74.13	635.2	376.4	167.3	389.0	317.6	478.5
LNFP-III	G1	PRE-TERM	44	5	204.0	528.1	337.2	137.4	284.1	238.8	431.0
LNFP-III	G1	PRE-TERM	45	9	89.27	599.0	348.5	191.7	358.2	204.7	543.4
LNFP-III	G1	PRE-TERM	46	6	187.7	513.1	322.5	124.1	322.1	220.8	382.6
LNFP-III	G1	PRE-TERM	47	3	140.7	774.0	387.5	339.0	247.7	194.2	510.9
LNFP-III	G1	PRE-TERM	48	4	266.6	338.5	295.8	34.59	289.0	268.2	316.5
LNFP-III	G1	TERM	38	2	444.4	477.8	461.1	23.62	461.1	452.8	469.5
LNFP-III	G1	TERM	39	9	192.5	476.1	369.1	102.9	385.9	309.8	459.1
LNFP-III	G1	TERM	40	13	163.3	434.1	334.3	87.62	364.1	315.5	394.7
LNFP-III	G1	TERM	41	21	169.8	490.3	305.3	94.34	307.3	229.9	348.4
LNFP-III	G1	TERM	42	21	153.7	432.9	270.1	75.96	272.1	222.5	301.8
LNFP-III	G1	TERM	43	21	165.5	575.0	277.7	102.4	246.0	216.6	294.9
LNFP-III	G1	TERM	44	20	153.3	438.5	259.7	69.98	249.8	211.9	308.8
LNFP-III	G1	TERM	45	21	17.50	498.9	281.7	100.4	280.1	236.4	335.9
LNFP-III	G1	TERM	46	17	108.0	508.6	299.3	103.8	280.7	229.6	373.3
LNFP-III	G1	TERM	47	12	164.2	526.2	284.5	96.54	248.5	230.9	344.7
LNFP-III	G1	TERM	48	6	209.5	398.2	282.0	69.29	280.3	229.6	303.3
LNFP-III	G2	PRE-TERM	29	1	682.1	682.1	682.1	NA	682.1	682.1	682.1
LNFP-III	G2	PRE-TERM	30	2	558.9	569.3	564.1	7.383	564.1	561.5	566.7
LNFP-III	G2	PRE-TERM	31	2	465.2	640.0	552.6	123.6	552.6	508.9	596.3
LNFP-III	G2	PRE-TERM	32	3	251.7	654.7	479.5	206.6	532.2	391.9	593.5

Table S4 Concentration of Human Milk Oligosaccharides in Term or Preterm Milk At Specified Postmenstrual Age Separated By Milk Group

** When there are results below the method limit of quanfification (LoQ) the result has been assigned value of 0.5 × LoQ, hence the minimum value appears to be the same in many cases. When a large number of datapoints are below LoQ this can also have the effect that the median = minimum.*

HMO	Milk Group	Study Arm	Postmenstrual Age (weeks)	N	HMO Concentration (mg/L) min *	max	mean	sd	median *	Quartile 1 (25%)	Quartile 3 (75%)
LNFP-III	G2	PRE-TERM	33	5	230.6	727.3	518.5	209.1	504.4	416.8	713.1
LNFP-III	G2	PRE-TERM	34	5	209.6	779.2	494.4	253.6	409.1	331.9	742.0
LNFP-III	G2	PRE-TERM	35	5	255.7	733.6	450.3	177.1	445.5	365.1	451.6
LNFP-III	G2	PRE-TERM	36	5	237.8	711.8	432.6	179.3	426.4	324.2	463.0
LNFP-III	G2	PRE-TERM	37	4	394.8	767.7	544.3	158.3	507.4	472.4	579.3
LNFP-III	G2	PRE-TERM	38	4	243.5	543.6	371.8	126.3	350.1	307.2	414.7
LNFP-III	G2	PRE-TERM	39	3	251.4	432.6	326.7	94.38	296.2	273.8	364.4
LNFP-III	G2	PRE-TERM	40	3	238.6	469.7	327.2	124.6	273.4	256.0	371.6
LNFP-III	G2	PRE-TERM	41	2	354.4	616.5	485.5	185.4	485.5	419.9	551.0
LNFP-III	G2	PRE-TERM	42	3	228.7	526.8	353.9	154.7	306.1	267.4	416.5
LNFP-III	G2	PRE-TERM	43	2	522.3	552.8	537.6	21.54	537.6	530.0	545.2
LNFP-III	G2	PRE-TERM	44	3	214.5	472.4	315.4	137.8	259.4	237.0	365.9
LNFP-III	G2	PRE-TERM	45	2	271.7	354.6	313.1	58.62	313.1	292.4	333.8
LNFP-III	G2	PRE-TERM	46	2	352.7	750.3	551.5	281.1	551.5	452.1	650.9
LNFP-III	G2	PRE-TERM	48	1	345.5	345.5	345.5	NA	345.5	345.5	345.5
LNFP-III	G2	TERM	39	1	826.9	826.9	826.9	NA	826.9	826.9	826.9
LNFP-III	G2	TERM	40	5	76.48	685.9	488.0	242.2	590.7	474.8	611.8
LNFP-III	G2	TERM	41	4	388.9	515.2	463.7	53.35	475.2	450.8	488.1
LNFP-III	G2	TERM	42	6	318.0	525.0	400.5	86.04	369.2	338.0	462.9
LNFP-III	G2	TERM	43	5	273.1	494.3	341.8	90.95	294.0	290.7	356.7
LNFP-III	G2	TERM	44	5	247.7	428.0	338.6	69.15	336.1	302.7	378.4
LNFP-III	G2	TERM	45	5	147.3	499.8	358.4	129.7	394.5	354.7	395.6
LNFP-III	G2	TERM	46	5	280.5	461.6	349.8	71.14	319.1	312.8	375.0
LNFP-III	G2	TERM	47	4	272.6	493.6	361.7	96.31	340.2	302.3	399.6
LNFP-III	G3	PRE-TERM	30	1	322.8	322.8	322.8	NA	322.8	322.8	322.8
LNFP-III	G3	PRE-TERM	31	1	418.0	418.0	418.0	NA	418.0	418.0	418.0
LNFP-III	G3	PRE-TERM	32	1	352.3	352.3	352.3	NA	352.3	352.3	352.3
LNFP-III	G3	PRE-TERM	33	1	290.1	290.1	290.1	NA	290.1	290.1	290.1
LNFP-III	G3	PRE-TERM	34	1	324.2	324.2	324.2	NA	324.2	324.2	324.2
LNFP-III	G3	PRE-TERM	35	1	430.5	430.5	430.5	NA	430.5	430.5	430.5
LNFP-III	G3	PRE-TERM	36	1	334.1	334.1	334.1	NA	334.1	334.1	334.1
LNFP-III	G3	PRE-TERM	37	1	340.3	340.3	340.3	NA	340.3	340.3	340.3
LNFP-III	G3	PRE-TERM	39	1	329.7	329.7	329.7	NA	329.7	329.7	329.7
LNFP-III	G3	PRE-TERM	41	1	357.3	357.3	357.3	NA	357.3	357.3	357.3
LNFP-III	G3	PRE-TERM	43	1	404.7	404.7	404.7	NA	404.7	404.7	404.7
LNFP-III	G3	PRE-TERM	45	1	407.3	407.3	407.3	NA	407.3	407.3	407.3

Table S4 Concentration of Human Milk Oligosaccharides in Term or Preterm Milk At Specified Postmenstrual Age Separated By Milk Group

** When there are results below the method limit of quanfification (LoQ) the result has been assigned value of 0.5 × LoQ, hence the minimum value appears to be the same in many cases. When a large number of datapoints are below LoQ this can also have the effect that the median = minimum.*

					HMO Concentration (mg/L)						
HMO	Milk Group	Study Arm	Postmenstrual Age (weeks)	N	min *	max	mean	sd	median *	Quartile 1 (25%)	Quartile 3 (75%)
LNFP-III	G3	TERM	41	1	329.2	329.2	329.2	NA	329.2	329.2	329.2
LNFP-III	G3	TERM	42	1	262.9	262.9	262.9	NA	262.9	262.9	262.9
LNFP-III	G3	TERM	43	1	219.7	219.7	219.7	NA	219.7	219.7	219.7
LNFP-III	G3	TERM	44	1	238.9	238.9	238.9	NA	238.9	238.9	238.9
LNFP-III	G3	TERM	45	1	202.2	202.2	202.2	NA	202.2	202.2	202.2
LNFP-III	G3	TERM	46	1	196.3	196.3	196.3	NA	196.3	196.3	196.3
LNFP-III	G3	TERM	47	1	190.9	190.9	190.9	NA	190.9	190.9	190.9
LNFP-III	G3	TERM	48	1	262.5	262.5	262.5	NA	262.5	262.5	262.5
LNFP-III	G4	TERM	40	1	1268	1268	1268	NA	1268	1268	1268
LNFP-III	G4	TERM	41	1	1169	1169	1169	NA	1169	1169	1169
LNFP-III	G4	TERM	42	1	911.0	911.0	911.0	NA	911.0	911.0	911.0
LNFP-III	G4	TERM	43	1	823.9	823.9	823.9	NA	823.9	823.9	823.9
LNFP-III	G4	TERM	44	1	714.0	714.0	714.0	NA	714.0	714.0	714.0
LNFP-III	G4	TERM	45	1	786.1	786.1	786.1	NA	786.1	786.1	786.1
LNFP-III	G4	TERM	46	1	703.6	703.6	703.6	NA	703.6	703.6	703.6
LNFP-III	G4	TERM	47	1	569.7	569.7	569.7	NA	569.7	569.7	569.7
LNFP-V	G1	PRE-TERM	30	7	12.00	140.4	39.58	47.66	12.00	12.00	44.35
LNFP-V	G1	PRE-TERM	31	9	12.00	140.5	49.23	39.55	43.27	25.98	48.02
LNFP-V	G1	PRE-TERM	32	13	12.00	123.0	41.26	36.21	26.19	12.00	58.17
LNFP-V	G1	PRE-TERM	33	18	12.00	112.6	37.84	27.64	29.42	12.00	56.13
LNFP-V	G1	PRE-TERM	34	19	12.00	108.5	47.58	31.82	39.99	18.59	66.41
LNFP-V	G1	PRE-TERM	35	18	12.00	123.1	52.73	32.85	51.16	30.94	73.04
LNFP-V	G1	PRE-TERM	36	19	12.00	121.4	49.64	34.29	50.05	12.00	73.91
LNFP-V	G1	PRE-TERM	37	18	12.00	116.0	49.27	31.00	43.18	30.19	66.17
LNFP-V	G1	PRE-TERM	38	11	12.00	109.0	38.29	33.60	33.31	12.00	49.35
LNFP-V	G1	PRE-TERM	39	16	12.00	100.1	51.60	33.35	37.55	25.20	83.90
LNFP-V	G1	PRE-TERM	40	7	12.00	105.7	51.95	41.44	28.20	18.58	90.29
LNFP-V	G1	PRE-TERM	41	12	12.00	83.16	38.41	26.04	33.62	12.00	58.71
LNFP-V	G1	PRE-TERM	42	6	12.00	85.92	46.87	38.46	42.80	12.00	82.66
LNFP-V	G1	PRE-TERM	43	10	12.00	85.62	44.58	23.76	44.10	30.56	59.72
LNFP-V	G1	PRE-TERM	44	5	12.00	72.64	48.85	27.80	61.74	26.56	71.31
LNFP-V	G1	PRE-TERM	45	9	12.00	122.5	44.70	33.14	40.12	27.59	53.26
LNFP-V	G1	PRE-TERM	46	6	12.00	80.64	43.67	30.01	45.27	16.03	65.72
LNFP-V	G1	PRE-TERM	47	3	12.00	47.17	31.89	18.03	36.51	24.26	41.84
LNFP-V	G1	PRE-TERM	48	4	12.00	68.59	40.66	24.95	41.02	25.12	56.56
LNFP-V	G1	TERM	38	2	12.00	30.70	21.35	13.23	21.35	16.68	26.03

Table S4 Concentration of Human Milk Oligosaccharides in Term or Preterm Milk At Specified Postmenstrual Age Separated By Milk Group

** When there are results below the method limit of quanfification (LoQ) the result has been assigned value of 0.5 × LoQ, hence the minimum value appears to be the same in many cases. When a large number of datapoints are below LoQ this can also have the effect that the median = minimum.*

					HMO Concentration (mg/L)						
HMO	Milk Group	Study Arm	Postmenstrual Age (weeks)	N	min *	max	mean	sd	median *	Quartile 1 (25%)	Quartile 3 (75%)
LNFP-V	G1	TERM	39	9	12.00	72.60	27.36	22.16	12.00	12.00	36.05
LNFP-V	G1	TERM	40	13	12.00	66.46	37.07	19.47	39.71	24.13	54.21
LNFP-V	G1	TERM	41	21	12.00	349.1	56.24	69.68	41.54	31.94	55.81
LNFP-V	G1	TERM	42	21	12.00	75.41	43.41	18.92	43.48	27.84	56.79
LNFP-V	G1	TERM	43	21	12.00	83.09	37.96	23.66	37.15	12.00	53.37
LNFP-V	G1	TERM	44	20	12.00	84.50	39.06	20.97	35.48	25.16	57.29
LNFP-V	G1	TERM	45	21	12.00	94.30	40.42	24.22	36.17	25.64	56.51
LNFP-V	G1	TERM	46	17	12.00	105.3	42.23	26.95	36.20	12.00	56.43
LNFP-V	G1	TERM	47	12	12.00	101.5	39.96	30.98	31.02	12.00	58.46
LNFP-V	G1	TERM	48	6	12.00	38.23	18.38	10.85	12.00	12.00	21.02
LNFP-V	G2	PRE-TERM	29	1	320.9	320.9	320.9	NA	320.9	320.9	320.9
LNFP-V	G2	PRE-TERM	30	2	191.2	289.0	240.1	69.11	240.1	215.7	264.5
LNFP-V	G2	PRE-TERM	31	2	252.0	266.9	259.5	10.52	259.5	255.7	263.2
LNFP-V	G2	PRE-TERM	32	3	179.9	286.3	233.3	53.19	233.8	206.8	260.0
LNFP-V	G2	PRE-TERM	33	5	172.5	300.2	250.8	52.36	261.9	227.2	292.2
LNFP-V	G2	PRE-TERM	34	5	149.2	317.8	244.9	81.40	292.9	163.8	301.0
LNFP-V	G2	PRE-TERM	35	5	142.7	359.5	252.4	97.83	296.5	154.2	309.0
LNFP-V	G2	PRE-TERM	36	5	134.9	363.8	257.7	91.86	268.3	200.4	321.2
LNFP-V	G2	PRE-TERM	37	4	181.9	285.4	234.8	46.62	235.9	204.4	266.3
LNFP-V	G2	PRE-TERM	38	4	123.3	309.0	206.7	76.65	197.2	176.2	227.6
LNFP-V	G2	PRE-TERM	39	3	165.7	299.1	225.8	67.66	212.6	189.2	255.9
LNFP-V	G2	PRE-TERM	40	3	117.2	207.0	175.9	50.89	203.6	160.4	205.3
LNFP-V	G2	PRE-TERM	41	2	182.5	328.8	255.7	103.5	255.7	219.1	292.3
LNFP-V	G2	PRE-TERM	42	3	111.5	165.1	137.1	26.87	134.9	123.2	150.0
LNFP-V	G2	PRE-TERM	43	2	80.43	200.7	140.5	85.02	140.5	110.5	170.6
LNFP-V	G2	PRE-TERM	44	3	102.8	194.4	138.5	48.98	118.5	110.6	156.4
LNFP-V	G2	PRE-TERM	45	2	118.6	408.7	263.6	205.1	263.6	191.1	336.2
LNFP-V	G2	PRE-TERM	46	2	87.30	202.6	145.0	81.56	145.0	116.1	173.8
LNFP-V	G2	PRE-TERM	48	1	169.4	169.4	169.4	NA	169.4	169.4	169.4
LNFP-V	G2	TERM	39	1	138.6	138.6	138.6	NA	138.6	138.6	138.6
LNFP-V	G2	TERM	40	5	128.7	191.8	166.2	28.44	181.0	143.0	186.6
LNFP-V	G2	TERM	41	4	139.7	226.3	185.2	44.53	187.5	150.8	221.9
LNFP-V	G2	TERM	42	6	147.2	247.0	194.7	35.99	200.2	170.1	210.3
LNFP-V	G2	TERM	43	5	139.2	202.6	175.5	25.73	173.2	165.2	197.5
LNFP-V	G2	TERM	44	5	119.8	228.2	164.4	41.77	154.3	140.1	179.4
LNFP-V	G2	TERM	45	5	46.89	226.4	135.0	65.02	142.8	111.1	148.0

Table S4 Concentration of Human Milk Oligosaccharides in Term or Preterm Milk At Specified Postmenstrual Age Separated By Milk Group

** When there are results below the method limit of quanfification (LoQ) the result has been assigned value of 0.5 × LoQ, hence the minimum value appears to be the same in many cases. When a large number of datapoints are below LoQ this can also have the effect that the median = minimum.*

HMO	Milk Group	Study Arm	Postmenstrual Age (weeks)	N	HMO Concentration (mg/L) min *	max	mean	sd	median *	Quartile 1 (25%)	Quartile 3 (75%)
LNFP-V	G2	TERM	46	5	87.21	162.9	121.4	32.92	124.8	89.82	142.2
LNFP-V	G2	TERM	47	4	76.47	196.7	127.7	51.00	118.8	99.64	146.9
LNFP-V	G3	PRE-TERM	30	1	12.00	12.00	12.00	NA	12.00	12.00	12.00
LNFP-V	G3	PRE-TERM	31	1	12.00	12.00	12.00	NA	12.00	12.00	12.00
LNFP-V	G3	PRE-TERM	32	1	24.37	24.37	24.37	NA	24.37	24.37	24.37
LNFP-V	G3	PRE-TERM	33	1	12.00	12.00	12.00	NA	12.00	12.00	12.00
LNFP-V	G3	PRE-TERM	34	1	12.00	12.00	12.00	NA	12.00	12.00	12.00
LNFP-V	G3	PRE-TERM	35	1	12.00	12.00	12.00	NA	12.00	12.00	12.00
LNFP-V	G3	PRE-TERM	36	1	12.00	12.00	12.00	NA	12.00	12.00	12.00
LNFP-V	G3	PRE-TERM	37	1	12.00	12.00	12.00	NA	12.00	12.00	12.00
LNFP-V	G3	PRE-TERM	39	1	12.00	12.00	12.00	NA	12.00	12.00	12.00
LNFP-V	G3	PRE-TERM	41	1	26.89	26.89	26.89	NA	26.89	26.89	26.89
LNFP-V	G3	PRE-TERM	43	1	25.61	25.61	25.61	NA	25.61	25.61	25.61
LNFP-V	G3	PRE-TERM	45	1	28.52	28.52	28.52	NA	28.52	28.52	28.52
LNFP-V	G3	TERM	41	1	25.88	25.88	25.88	NA	25.88	25.88	25.88
LNFP-V	G3	TERM	42	1	12.00	12.00	12.00	NA	12.00	12.00	12.00
LNFP-V	G3	TERM	43	1	12.00	12.00	12.00	NA	12.00	12.00	12.00
LNFP-V	G3	TERM	44	1	12.00	12.00	12.00	NA	12.00	12.00	12.00
LNFP-V	G3	TERM	45	1	12.00	12.00	12.00	NA	12.00	12.00	12.00
LNFP-V	G3	TERM	46	1	12.00	12.00	12.00	NA	12.00	12.00	12.00
LNFP-V	G3	TERM	47	1	12.00	12.00	12.00	NA	12.00	12.00	12.00
LNFP-V	G3	TERM	48	1	12.00	12.00	12.00	NA	12.00	12.00	12.00
LNFP-V	G4	TERM	40	1	203.7	203.7	203.7	NA	203.7	203.7	203.7
LNFP-V	G4	TERM	41	1	268.1	268.1	268.1	NA	268.1	268.1	268.1
LNFP-V	G4	TERM	42	1	127.3	127.3	127.3	NA	127.3	127.3	127.3
LNFP-V	G4	TERM	43	1	140.0	140.0	140.0	NA	140.0	140.0	140.0
LNFP-V	G4	TERM	44	1	103.6	103.6	103.6	NA	103.6	103.6	103.6
LNFP-V	G4	TERM	45	1	139.8	139.8	139.8	NA	139.8	139.8	139.8
LNFP-V	G4	TERM	46	1	102.4	102.4	102.4	NA	102.4	102.4	102.4
LNFP-V	G4	TERM	47	1	119.7	119.7	119.7	NA	119.7	119.7	119.7
LNnDFH	G1	PRE-TERM	30	7	14.00	110.4	46.87	33.45	41.42	22.90	58.24
LNnDFH	G1	PRE-TERM	31	9	14.00	76.33	36.41	25.68	33.79	14.00	50.41
LNnDFH	G1	PRE-TERM	32	13	14.00	65.59	25.08	18.20	14.00	14.00	39.02
LNnDFH	G1	PRE-TERM	33	18	14.00	83.93	20.04	17.19	14.00	14.00	14.00
LNnDFH	G1	PRE-TERM	34	19	14.00	120.6	23.93	25.00	14.00	14.00	21.52
LNnDFH	G1	PRE-TERM	35	18	14.00	58.17	22.39	14.97	14.00	14.00	26.59

Table S4 Concentration of Human Milk Oligosaccharides in Term or Preterm Milk At Specified Postmenstrual Age Separated By Milk Group

** When there are results below the method limit of quanfification (LoQ) the result has been assigned value of 0.5 × LoQ, hence the minimum value appears to be the same in many cases. When a large number of datapoints are below LoQ this can also have the effect that the median = minimum.*

					HMO Concentration (mg/L)						
HMO	Milk Group	Study Arm	Postmenstrual Age (weeks)	N	min *	max	mean	sd	median *	Quartile 1 (25%)	Quartile 3 (75%)
LNnDFH	G1	PRE-TERM	36	19	14.00	47.73	18.11	10.01	14.00	14.00	14.00
LNnDFH	G1	PRE-TERM	37	18	14.00	183.2	27.90	39.76	14.00	14.00	24.94
LNnDFH	G1	PRE-TERM	38	11	14.00	97.37	32.34	32.42	14.00	14.00	37.91
LNnDFH	G1	PRE-TERM	39	16	14.00	107.7	28.54	26.18	14.00	14.00	34.17
LNnDFH	G1	PRE-TERM	40	7	14.00	30.88	18.50	7.720	14.00	14.00	21.33
LNnDFH	G1	PRE-TERM	41	12	14.00	85.19	39.11	27.29	34.29	14.00	59.93
LNnDFH	G1	PRE-TERM	42	6	14.00	46.53	25.77	13.99	21.67	14.00	34.88
LNnDFH	G1	PRE-TERM	43	10	14.00	75.44	31.15	22.17	21.79	14.00	39.02
LNnDFH	G1	PRE-TERM	44	5	14.00	67.08	33.41	21.88	33.20	14.00	38.76
LNnDFH	G1	PRE-TERM	45	9	14.00	67.12	33.84	20.95	30.74	14.00	49.84
LNnDFH	G1	PRE-TERM	46	6	14.00	54.35	29.56	17.88	25.75	14.00	42.03
LNnDFH	G1	PRE-TERM	47	3	14.00	105.1	44.36	52.59	14.00	14.00	59.54
LNnDFH	G1	PRE-TERM	48	4	14.00	55.46	32.91	22.04	31.09	14.00	50.00
LNnDFH	G1	TERM	38	2	50.53	106.3	78.39	39.40	78.39	64.46	92.32
LNnDFH	G1	TERM	39	9	14.00	181.8	52.25	56.69	28.53	14.00	77.61
LNnDFH	G1	TERM	40	13	14.00	172.8	56.46	46.95	33.46	29.71	76.91
LNnDFH	G1	TERM	41	21	14.00	110.0	28.98	26.60	14.00	14.00	30.45
LNnDFH	G1	TERM	42	21	14.00	52.71	24.89	13.80	14.00	14.00	34.89
LNnDFH	G1	TERM	43	21	14.00	77.46	26.39	18.17	14.00	14.00	41.96
LNnDFH	G1	TERM	44	20	14.00	85.76	22.72	18.46	14.00	14.00	17.69
LNnDFH	G1	TERM	45	21	14.00	132.3	30.41	32.51	14.00	14.00	33.10
LNnDFH	G1	TERM	46	17	14.00	94.89	26.60	23.19	14.00	14.00	36.06
LNnDFH	G1	TERM	47	12	14.00	85.75	29.96	23.81	21.48	14.00	30.40
LNnDFH	G1	TERM	48	6	14.00	70.18	26.24	22.61	14.00	14.00	26.94
LNnDFH	G2	PRE-TERM	29	1	543.1	543.1	543.1	NA	543.1	543.1	543.1
LNnDFH	G2	PRE-TERM	30	2	14.00	299.7	156.8	202.0	156.8	85.42	228.3
LNnDFH	G2	PRE-TERM	31	2	14.00	333.0	173.5	225.6	173.5	93.76	253.3
LNnDFH	G2	PRE-TERM	32	3	14.00	195.3	74.44	104.7	14.00	14.00	104.7
LNnDFH	G2	PRE-TERM	33	5	14.00	430.0	160.7	170.5	159.1	14.00	186.3
LNnDFH	G2	PRE-TERM	34	5	62.05	196.9	128.1	59.85	145.0	69.53	167.1
LNnDFH	G2	PRE-TERM	35	5	14.00	185.4	107.2	75.38	77.51	74.52	184.6
LNnDFH	G2	PRE-TERM	36	5	14.00	156.3	74.48	53.66	72.38	42.68	87.06
LNnDFH	G2	PRE-TERM	37	4	14.00	138.4	84.01	51.61	91.83	70.38	105.5
LNnDFH	G2	PRE-TERM	38	4	14.00	119.8	78.65	47.76	90.40	57.34	111.7
LNnDFH	G2	PRE-TERM	39	3	14.00	65.02	42.84	26.15	49.49	31.74	57.25
LNnDFH	G2	PRE-TERM	40	3	55.32	115.8	87.31	30.40	90.81	73.07	103.3

Table S4 Concentration of Human Milk Oligosaccharides in Term or Preterm Milk At Specified Postmenstrual Age Separated By Milk Group

** When there are results below the method limit of quanfification (LoQ) the result has been assigned value of 0.5 × LoQ, hence the minimum value appears to be the same in many cases. When a large number of datapoints are below LoQ this can also have the effect that the median = minimum.*

					HMO Concentration (mg/L)						
**HMO**	**Milk Group**	**Study Arm**	**Postmenstrual Age (weeks)**	**N**	**min ***	**max**	**mean**	**sd**	**median ***	**Quartile 1 (25%)**	**Quartile 3 (75%)**
LNnDFH	G2	PRE-TERM	41	2	14.00	14.00	14.00	0.0000	14.00	14.00	14.00
LNnDFH	G2	PRE-TERM	42	3	14.00	125.7	75.13	56.58	85.71	49.85	105.7
LNnDFH	G2	PRE-TERM	43	2	14.00	14.00	14.00	0.0000	14.00	14.00	14.00
LNnDFH	G2	PRE-TERM	44	3	14.00	109.1	66.50	48.33	76.39	45.19	92.76
LNnDFH	G2	PRE-TERM	45	2	14.00	14.00	14.00	0.0000	14.00	14.00	14.00
LNnDFH	G2	PRE-TERM	46	2	78.60	733.1	405.8	462.8	405.8	242.2	569.5
LNnDFH	G2	PRE-TERM	48	1	94.85	94.85	94.85	NA	94.85	94.85	94.85
LNnDFH	G2	TERM	39	1	14.00	14.00	14.00	NA	14.00	14.00	14.00
LNnDFH	G2	TERM	40	5	14.00	48.50	20.90	15.43	14.00	14.00	14.00
LNnDFH	G2	TERM	41	4	14.00	46.71	22.18	16.35	14.00	14.00	22.18
LNnDFH	G2	TERM	42	6	14.00	40.38	18.40	10.77	14.00	14.00	14.00
LNnDFH	G2	TERM	43	5	14.00	34.25	18.05	9.055	14.00	14.00	14.00
LNnDFH	G2	TERM	44	5	14.00	31.98	17.60	8.039	14.00	14.00	14.00
LNnDFH	G2	TERM	45	5	14.00	97.13	30.63	37.18	14.00	14.00	14.00
LNnDFH	G2	TERM	46	5	14.00	77.39	26.68	28.35	14.00	14.00	14.00
LNnDFH	G2	TERM	47	4	14.00	94.07	34.02	40.04	14.00	14.00	34.02
LNnDFH	G3	PRE-TERM	30	1	14.00	14.00	14.00	NA	14.00	14.00	14.00
LNnDFH	G3	PRE-TERM	31	1	28.06	28.06	28.06	NA	28.06	28.06	28.06
LNnDFH	G3	PRE-TERM	32	1	14.00	14.00	14.00	NA	14.00	14.00	14.00
LNnDFH	G3	PRE-TERM	33	1	14.00	14.00	14.00	NA	14.00	14.00	14.00
LNnDFH	G3	PRE-TERM	34	1	14.00	14.00	14.00	NA	14.00	14.00	14.00
LNnDFH	G3	PRE-TERM	35	1	14.00	14.00	14.00	NA	14.00	14.00	14.00
LNnDFH	G3	PRE-TERM	36	1	14.00	14.00	14.00	NA	14.00	14.00	14.00
LNnDFH	G3	PRE-TERM	37	1	14.00	14.00	14.00	NA	14.00	14.00	14.00
LNnDFH	G3	PRE-TERM	39	1	14.00	14.00	14.00	NA	14.00	14.00	14.00
LNnDFH	G3	PRE-TERM	41	1	14.00	14.00	14.00	NA	14.00	14.00	14.00
LNnDFH	G3	PRE-TERM	43	1	14.00	14.00	14.00	NA	14.00	14.00	14.00
LNnDFH	G3	PRE-TERM	45	1	14.00	14.00	14.00	NA	14.00	14.00	14.00
LNnDFH	G3	TERM	41	1	14.00	14.00	14.00	NA	14.00	14.00	14.00
LNnDFH	G3	TERM	42	1	14.00	14.00	14.00	NA	14.00	14.00	14.00
LNnDFH	G3	TERM	43	1	28.07	28.07	28.07	NA	28.07	28.07	28.07
LNnDFH	G3	TERM	44	1	29.18	29.18	29.18	NA	29.18	29.18	29.18
LNnDFH	G3	TERM	45	1	14.00	14.00	14.00	NA	14.00	14.00	14.00
LNnDFH	G3	TERM	46	1	14.00	14.00	14.00	NA	14.00	14.00	14.00
LNnDFH	G3	TERM	47	1	14.00	14.00	14.00	NA	14.00	14.00	14.00
LNnDFH	G3	TERM	48	1	33.19	33.19	33.19	NA	33.19	33.19	33.19

Table S4 Concentration of Human Milk Oligosaccharides in Term or Preterm Milk At Specified Postmenstrual Age Separated By Milk Group

** When there are results below the method limit of quanfification (LoQ) the result has been assigned value of 0.5 × LoQ, hence the minimum value appears to be the same in many cases. When a large number of datapoints are below LoQ this can also have the effect that the median = minimum.*

HMO	Milk Group	Study Arm	Postmenstrual Age (weeks)	N	HMO Concentration (mg/L) min *	max	mean	sd	median *	Quartile 1 (25%)	Quartile 3 (75%)
LNnDFH	G4	TERM	40	1	60.72	60.72	60.72	NA	60.72	60.72	60.72
LNnDFH	G4	TERM	41	1	55.10	55.10	55.10	NA	55.10	55.10	55.10
LNnDFH	G4	TERM	42	1	14.00	14.00	14.00	NA	14.00	14.00	14.00
LNnDFH	G4	TERM	43	1	14.00	14.00	14.00	NA	14.00	14.00	14.00
LNnDFH	G4	TERM	44	1	14.00	14.00	14.00	NA	14.00	14.00	14.00
LNnDFH	G4	TERM	45	1	14.00	14.00	14.00	NA	14.00	14.00	14.00
LNnDFH	G4	TERM	46	1	14.00	14.00	14.00	NA	14.00	14.00	14.00
LNnDFH	G4	TERM	47	1	14.00	14.00	14.00	NA	14.00	14.00	14.00
LNnFP	G1	PRE-TERM	30	7	9.500	89.76	25.08	29.39	9.500	9.500	23.89
LNnFP	G1	PRE-TERM	31	9	9.500	50.62	16.94	13.82	9.500	9.500	21.61
LNnFP	G1	PRE-TERM	32	13	9.500	50.34	12.64	11.33	9.500	9.500	9.500
LNnFP	G1	PRE-TERM	33	18	9.500	59.82	15.09	13.48	9.500	9.500	9.500
LNnFP	G1	PRE-TERM	34	19	9.500	61.67	14.16	12.37	9.500	9.500	9.500
LNnFP	G1	PRE-TERM	35	18	9.500	60.47	15.60	12.95	9.500	9.500	17.45
LNnFP	G1	PRE-TERM	36	19	9.500	55.24	14.51	11.16	9.500	9.500	14.75
LNnFP	G1	PRE-TERM	37	18	9.500	57.65	14.87	11.97	9.500	9.500	16.65
LNnFP	G1	PRE-TERM	38	11	9.500	32.68	14.48	9.093	9.500	9.500	14.55
LNnFP	G1	PRE-TERM	39	16	9.500	60.19	18.58	16.84	9.500	9.500	21.89
LNnFP	G1	PRE-TERM	40	7	9.500	9.500	9.500	0.0000	9.500	9.500	9.500
LNnFP	G1	PRE-TERM	41	12	9.500	53.23	18.93	15.60	9.500	9.500	24.78
LNnFP	G1	PRE-TERM	42	6	9.500	21.55	11.51	4.920	9.500	9.500	9.500
LNnFP	G1	PRE-TERM	43	10	9.500	70.04	19.09	21.06	9.500	9.500	9.500
LNnFP	G1	PRE-TERM	44	5	9.500	22.23	14.15	6.410	9.500	9.500	20.00
LNnFP	G1	PRE-TERM	45	9	9.500	88.05	22.81	28.01	9.500	9.500	9.500
LNnFP	G1	PRE-TERM	46	6	9.500	9.500	9.500	0.0000	9.500	9.500	9.500
LNnFP	G1	PRE-TERM	47	3	9.500	56.14	25.05	26.93	9.500	9.500	32.82
LNnFP	G1	PRE-TERM	48	4	9.500	23.76	13.06	7.128	9.500	9.500	13.06
LNnFP	G1	TERM	38	2	9.500	19.80	14.65	7.285	14.65	12.08	17.23
LNnFP	G1	TERM	39	9	9.500	38.38	15.90	12.70	9.500	9.500	9.500
LNnFP	G1	TERM	40	13	9.500	32.19	13.76	8.262	9.500	9.500	9.500
LNnFP	G1	TERM	41	21	9.500	23.38	11.29	4.513	9.500	9.500	9.500
LNnFP	G1	TERM	42	21	9.500	29.19	11.03	4.974	9.500	9.500	9.500
LNnFP	G1	TERM	43	21	9.500	66.17	15.40	13.09	9.500	9.500	19.20
LNnFP	G1	TERM	44	20	9.500	21.51	11.17	4.095	9.500	9.500	9.500
LNnFP	G1	TERM	45	21	9.500	20.46	11.42	4.072	9.500	9.500	9.500
LNnFP	G1	TERM	46	17	9.500	22.09	12.80	5.295	9.500	9.500	19.20

Table S4 Concentration of Human Milk Oligosaccharides in Term or Preterm Milk At Specified Postmenstrual Age Separated By Milk Group

** When there are results below the method limit of quanfification (LoQ) the result has been assigned value of 0.5 × LoQ, hence the minimum value appears to be the same in many cases. When a large number of datapoints are below LoQ this can also have the effect that the median = minimum.*

					HMO Concentration (mg/L)						
HMO	Milk Group	Study Arm	Postmenstrual Age (weeks)	N	min *	max	mean	sd	median *	Quartile 1 (25%)	Quartile 3 (75%)
LNnFP	G1	TERM	47	12	9.500	20.53	11.33	4.266	9.500	9.500	9.500
LNnFP	G1	TERM	48	6	9.500	9.500	9.500	0.0000	9.500	9.500	9.500
LNnFP	G2	PRE-TERM	29	1	28.70	28.70	28.70	NA	28.70	28.70	28.70
LNnFP	G2	PRE-TERM	30	2	25.44	28.16	26.80	1.923	26.80	26.12	27.48
LNnFP	G2	PRE-TERM	31	2	19.18	27.56	23.37	5.923	23.37	21.28	25.46
LNnFP	G2	PRE-TERM	32	3	9.500	40.83	19.94	18.09	9.500	9.500	25.16
LNnFP	G2	PRE-TERM	33	5	9.500	40.34	25.95	12.33	25.09	19.47	35.34
LNnFP	G2	PRE-TERM	34	5	9.500	27.64	16.10	9.109	9.500	9.500	24.35
LNnFP	G2	PRE-TERM	35	5	9.500	44.75	21.80	14.61	19.24	9.500	26.02
LNnFP	G2	PRE-TERM	36	5	9.500	45.15	19.49	15.62	9.500	9.500	23.82
LNnFP	G2	PRE-TERM	37	4	9.500	22.87	12.84	6.683	9.500	9.500	12.84
LNnFP	G2	PRE-TERM	38	4	9.500	9.500	9.500	0.0000	9.500	9.500	9.500
LNnFP	G2	PRE-TERM	39	3	9.500	9.500	9.500	0.0000	9.500	9.500	9.500
LNnFP	G2	PRE-TERM	40	3	9.500	9.500	9.500	0.0000	9.500	9.500	9.500
LNnFP	G2	PRE-TERM	41	2	9.500	9.500	9.500	0.0000	9.500	9.500	9.500
LNnFP	G2	PRE-TERM	42	3	9.500	9.500	9.500	0.0000	9.500	9.500	9.500
LNnFP	G2	PRE-TERM	43	2	9.500	9.500	9.500	0.0000	9.500	9.500	9.500
LNnFP	G2	PRE-TERM	44	3	9.500	9.500	9.500	0.0000	9.500	9.500	9.500
LNnFP	G2	PRE-TERM	45	2	9.500	9.500	9.500	0.0000	9.500	9.500	9.500
LNnFP	G2	PRE-TERM	46	2	9.500	22.67	16.09	9.314	16.09	12.79	19.38
LNnFP	G2	PRE-TERM	48	1	9.500	9.500	9.500	NA	9.500	9.500	9.500
LNnFP	G2	TERM	39	1	49.07	49.07	49.07	NA	49.07	49.07	49.07
LNnFP	G2	TERM	40	5	21.93	56.56	37.85	15.08	32.67	27.31	50.79
LNnFP	G2	TERM	41	4	9.500	29.02	16.85	9.354	14.44	9.500	21.78
LNnFP	G2	TERM	42	6	9.500	32.89	19.12	8.889	19.63	12.01	22.60
LNnFP	G2	TERM	43	5	9.500	19.79	11.56	4.601	9.500	9.500	9.500
LNnFP	G2	TERM	44	5	9.500	20.88	11.78	5.091	9.500	9.500	9.500
LNnFP	G2	TERM	45	5	9.500	23.84	17.09	7.154	19.24	9.500	23.36
LNnFP	G2	TERM	46	5	9.500	23.61	14.74	7.212	9.500	9.500	21.60
LNnFP	G2	TERM	47	4	9.500	28.50	18.42	10.34	17.83	9.500	26.75
LNnFP	G3	PRE-TERM	30	1	9.500	9.500	9.500	NA	9.500	9.500	9.500
LNnFP	G3	PRE-TERM	31	1	9.500	9.500	9.500	NA	9.500	9.500	9.500
LNnFP	G3	PRE-TERM	32	1	9.500	9.500	9.500	NA	9.500	9.500	9.500
LNnFP	G3	PRE-TERM	33	1	9.500	9.500	9.500	NA	9.500	9.500	9.500
LNnFP	G3	PRE-TERM	34	1	9.500	9.500	9.500	NA	9.500	9.500	9.500
LNnFP	G3	PRE-TERM	35	1	9.500	9.500	9.500	NA	9.500	9.500	9.500

Table S4 Concentration of Human Milk Oligosaccharides in Term or Preterm Milk At Specified Postmenstrual Age Separated By Milk Group

** When there are results below the method limit of quanfification (LoQ) the result has been assigned value of 0.5 × LoQ, hence the minimum value appears to be the same in many cases. When a large number of datapoints are below LoQ this can also have the effect that the median = minimum.*

					HMO Concentration (mg/L)						
**HMO**	**Milk Group**	**Study Arm**	**Postmenstrual Age (weeks)**	**N**	**min ***	**max**	**mean**	**sd**	**median ***	**Quartile 1 (25%)**	**Quartile 3 (75%)**
LNnFP	G3	PRE-TERM	36	1	9.500	9.500	9.500	NA	9.500	9.500	9.500
LNnFP	G3	PRE-TERM	37	1	9.500	9.500	9.500	NA	9.500	9.500	9.500
LNnFP	G3	PRE-TERM	39	1	9.500	9.500	9.500	NA	9.500	9.500	9.500
LNnFP	G3	PRE-TERM	41	1	9.500	9.500	9.500	NA	9.500	9.500	9.500
LNnFP	G3	PRE-TERM	43	1	9.500	9.500	9.500	NA	9.500	9.500	9.500
LNnFP	G3	PRE-TERM	45	1	9.500	9.500	9.500	NA	9.500	9.500	9.500
LNnFP	G3	TERM	41	1	9.500	9.500	9.500	NA	9.500	9.500	9.500
LNnFP	G3	TERM	42	1	9.500	9.500	9.500	NA	9.500	9.500	9.500
LNnFP	G3	TERM	43	1	9.500	9.500	9.500	NA	9.500	9.500	9.500
LNnFP	G3	TERM	44	1	9.500	9.500	9.500	NA	9.500	9.500	9.500
LNnFP	G3	TERM	45	1	9.500	9.500	9.500	NA	9.500	9.500	9.500
LNnFP	G3	TERM	46	1	9.500	9.500	9.500	NA	9.500	9.500	9.500
LNnFP	G3	TERM	47	1	9.500	9.500	9.500	NA	9.500	9.500	9.500
LNnFP	G3	TERM	48	1	9.500	9.500	9.500	NA	9.500	9.500	9.500
LNnFP	G4	TERM	40	1	9.500	9.500	9.500	NA	9.500	9.500	9.500
LNnFP	G4	TERM	41	1	9.500	9.500	9.500	NA	9.500	9.500	9.500
LNnFP	G4	TERM	42	1	49.35	49.35	49.35	NA	49.35	49.35	49.35
LNnFP	G4	TERM	43	1	40.04	40.04	40.04	NA	40.04	40.04	40.04
LNnFP	G4	TERM	44	1	52.21	52.21	52.21	NA	52.21	52.21	52.21
LNnFP	G4	TERM	45	1	56.13	56.13	56.13	NA	56.13	56.13	56.13
LNnFP	G4	TERM	46	1	37.85	37.85	37.85	NA	37.85	37.85	37.85
LNnFP	G4	TERM	47	1	23.06	23.06	23.06	NA	23.06	23.06	23.06
LNnT	G1	PRE-TERM	30	7	244.4	546.3	367.2	109.2	338.0	290.4	430.5
LNnT	G1	PRE-TERM	31	9	145.7	435.5	292.7	78.04	292.5	254.2	328.0
LNnT	G1	PRE-TERM	32	13	115.9	413.1	244.8	74.79	235.4	211.0	287.1
LNnT	G1	PRE-TERM	33	18	84.09	404.7	216.9	84.16	203.4	157.1	244.5
LNnT	G1	PRE-TERM	34	19	85.61	383.2	222.8	94.64	212.7	166.2	270.9
LNnT	G1	PRE-TERM	35	18	76.18	397.8	216.4	99.91	191.4	157.2	264.9
LNnT	G1	PRE-TERM	36	19	69.10	341.2	199.1	74.84	217.5	155.6	253.1
LNnT	G1	PRE-TERM	37	18	69.59	372.0	192.6	73.00	190.0	156.1	209.8
LNnT	G1	PRE-TERM	38	11	12.00	314.7	155.5	95.88	138.0	93.47	205.3
LNnT	G1	PRE-TERM	39	16	77.62	440.8	193.7	90.79	178.9	142.6	219.5
LNnT	G1	PRE-TERM	40	7	79.55	211.5	165.9	54.08	202.3	131.2	202.8
LNnT	G1	PRE-TERM	41	12	61.39	464.0	177.0	115.7	161.5	112.0	172.0
LNnT	G1	PRE-TERM	42	6	78.06	279.5	153.4	73.87	136.5	103.7	181.7
LNnT	G1	PRE-TERM	43	10	51.86	379.4	161.5	100.2	122.1	106.0	182.9

Table S4 Concentration of Human Milk Oligosaccharides in Term or Preterm Milk At Specified Postmenstrual Age Separated By Milk Group

** When there are results below the method limit of quanfification (LoQ) the result has been assigned value of 0.5 × LoQ, hence the minimum value appears to be the same in many cases. When a large number of datapoints are below LoQ this can also have the effect that the median = minimum.*

					HMO Concentration (mg/L)						
HMO	Milk Group	Study Arm	Postmenstrual Age (weeks)	N	min *	max	mean	sd	median *	Quartile 1 (25%)	Quartile 3 (75%)
LNnT	G1	PRE-TERM	44	5	89.73	229.8	149.0	52.41	131.7	129.1	165.0
LNnT	G1	PRE-TERM	45	9	38.26	444.3	160.2	133.0	119.5	68.35	246.2
LNnT	G1	PRE-TERM	46	6	86.23	180.1	123.5	40.65	119.3	87.82	148.9
LNnT	G1	PRE-TERM	47	3	34.77	437.3	184.3	220.3	80.91	57.84	259.1
LNnT	G1	PRE-TERM	48	4	75.22	208.8	129.2	63.50	116.3	79.45	166.0
LNnT	G1	TERM	38	2	377.9	400.4	389.2	15.95	389.2	383.5	394.8
LNnT	G1	TERM	39	9	162.4	565.3	367.7	139.5	418.4	260.1	459.2
LNnT	G1	TERM	40	13	127.0	452.2	294.0	88.16	287.9	235.9	343.3
LNnT	G1	TERM	41	21	149.5	435.1	273.3	79.58	264.5	223.2	325.8
LNnT	G1	TERM	42	21	108.9	350.3	206.1	67.67	193.4	145.2	252.6
LNnT	G1	TERM	43	21	78.28	369.5	185.4	77.74	182.3	116.8	243.9
LNnT	G1	TERM	44	20	74.62	311.2	162.7	68.54	146.1	118.0	190.6
LNnT	G1	TERM	45	21	79.18	281.9	161.6	61.88	145.0	116.5	199.9
LNnT	G1	TERM	46	17	64.27	284.9	150.6	65.15	140.6	113.6	177.1
LNnT	G1	TERM	47	12	52.58	245.5	133.5	56.83	130.0	82.12	164.4
LNnT	G1	TERM	48	6	64.38	195.3	109.2	47.22	98.38	79.07	119.0
LNnT	G2	PRE-TERM	29	1	165.4	165.4	165.4	NA	165.4	165.4	165.4
LNnT	G2	PRE-TERM	30	2	137.3	190.4	163.8	37.57	163.8	150.6	177.1
LNnT	G2	PRE-TERM	31	2	136.5	150.1	143.3	9.656	143.3	139.9	146.7
LNnT	G2	PRE-TERM	32	3	79.69	192.5	120.8	62.33	90.25	84.97	141.4
LNnT	G2	PRE-TERM	33	5	67.88	257.5	141.4	76.78	106.1	96.36	179.1
LNnT	G2	PRE-TERM	34	5	66.51	156.9	101.3	37.28	99.24	68.78	115.1
LNnT	G2	PRE-TERM	35	5	60.11	147.7	92.92	35.29	95.82	63.43	97.50
LNnT	G2	PRE-TERM	36	5	43.09	123.0	76.81	32.70	66.71	54.40	96.86
LNnT	G2	PRE-TERM	37	4	48.51	112.2	75.39	32.03	70.43	48.59	97.23
LNnT	G2	PRE-TERM	38	4	35.89	118.6	69.18	35.58	61.13	48.96	81.35
LNnT	G2	PRE-TERM	39	3	33.45	112.8	68.74	40.41	59.95	46.70	86.39
LNnT	G2	PRE-TERM	40	3	36.42	103.7	62.63	36.00	47.80	42.11	75.74
LNnT	G2	PRE-TERM	41	2	27.39	48.63	38.01	15.02	38.01	32.70	43.32
LNnT	G2	PRE-TERM	42	3	24.44	77.16	45.63	27.84	35.29	29.87	56.22
LNnT	G2	PRE-TERM	43	2	12.00	41.91	26.96	21.15	26.96	19.48	34.44
LNnT	G2	PRE-TERM	44	3	32.38	66.88	45.43	18.72	37.04	34.71	51.96
LNnT	G2	PRE-TERM	45	2	12.00	39.37	25.69	19.35	25.69	18.84	32.53
LNnT	G2	PRE-TERM	46	2	38.00	70.50	54.25	22.99	54.25	46.12	62.38
LNnT	G2	PRE-TERM	48	1	25.55	25.55	25.55	NA	25.55	25.55	25.55
LNnT	G2	TERM	39	1	251.3	251.3	251.3	NA	251.3	251.3	251.3

Table S4 Concentration of Human Milk Oligosaccharides in Term or Preterm Milk At Specified Postmenstrual Age Separated By Milk Group

** When there are results below the method limit of quanfification (LoQ) the result has been assigned value of 0.5 × LoQ, hence the minimum value appears to be the same in many cases. When a large number of datapoints are below LoQ this can also have the effect that the median = minimum.*

					HMO Concentration (mg/L)						
HMO	Milk Group	Study Arm	Postmenstrual Age (weeks)	N	min *	max	mean	sd	median *	Quartile 1 (25%)	Quartile 3 (75%)
LNnT	G2	TERM	40	5	113.9	338.7	216.6	81.39	214.0	187.1	229.1
LNnT	G2	TERM	41	4	67.58	136.8	115.3	32.58	128.4	107.8	135.9
LNnT	G2	TERM	42	6	52.63	131.0	105.0	28.92	113.8	97.70	123.3
LNnT	G2	TERM	43	5	54.25	129.1	90.76	31.13	82.76	71.58	116.1
LNnT	G2	TERM	44	5	41.39	147.0	83.69	46.59	59.76	51.21	119.1
LNnT	G2	TERM	45	5	12.00	175.5	90.14	71.43	79.17	33.31	150.6
LNnT	G2	TERM	46	5	35.80	141.0	85.40	51.96	76.38	36.11	137.7
LNnT	G2	TERM	47	4	27.23	158.7	107.5	58.64	122.1	83.08	146.5
LNnT	G3	PRE-TERM	30	1	215.7	215.7	215.7	NA	215.7	215.7	215.7
LNnT	G3	PRE-TERM	31	1	118.3	118.3	118.3	NA	118.3	118.3	118.3
LNnT	G3	PRE-TERM	32	1	92.48	92.48	92.48	NA	92.48	92.48	92.48
LNnT	G3	PRE-TERM	33	1	108.9	108.9	108.9	NA	108.9	108.9	108.9
LNnT	G3	PRE-TERM	34	1	88.14	88.14	88.14	NA	88.14	88.14	88.14
LNnT	G3	PRE-TERM	35	1	118.9	118.9	118.9	NA	118.9	118.9	118.9
LNnT	G3	PRE-TERM	36	1	71.24	71.24	71.24	NA	71.24	71.24	71.24
LNnT	G3	PRE-TERM	37	1	93.06	93.06	93.06	NA	93.06	93.06	93.06
LNnT	G3	PRE-TERM	39	1	73.63	73.63	73.63	NA	73.63	73.63	73.63
LNnT	G3	PRE-TERM	41	1	74.39	74.39	74.39	NA	74.39	74.39	74.39
LNnT	G3	PRE-TERM	43	1	85.57	85.57	85.57	NA	85.57	85.57	85.57
LNnT	G3	PRE-TERM	45	1	67.99	67.99	67.99	NA	67.99	67.99	67.99
LNnT	G3	TERM	41	1	394.6	394.6	394.6	NA	394.6	394.6	394.6
LNnT	G3	TERM	42	1	162.7	162.7	162.7	NA	162.7	162.7	162.7
LNnT	G3	TERM	43	1	159.5	159.5	159.5	NA	159.5	159.5	159.5
LNnT	G3	TERM	44	1	135.3	135.3	135.3	NA	135.3	135.3	135.3
LNnT	G3	TERM	45	1	129.1	129.1	129.1	NA	129.1	129.1	129.1
LNnT	G3	TERM	46	1	77.12	77.12	77.12	NA	77.12	77.12	77.12
LNnT	G3	TERM	47	1	77.74	77.74	77.74	NA	77.74	77.74	77.74
LNnT	G3	TERM	48	1	101.1	101.1	101.1	NA	101.1	101.1	101.1
LNnT	G4	TERM	40	1	399.6	399.6	399.6	NA	399.6	399.6	399.6
LNnT	G4	TERM	41	1	247.5	247.5	247.5	NA	247.5	247.5	247.5
LNnT	G4	TERM	42	1	183.6	183.6	183.6	NA	183.6	183.6	183.6
LNnT	G4	TERM	43	1	118.1	118.1	118.1	NA	118.1	118.1	118.1
LNnT	G4	TERM	44	1	12.00	12.00	12.00	NA	12.00	12.00	12.00
LNnT	G4	TERM	45	1	76.25	76.25	76.25	NA	76.25	76.25	76.25
LNnT	G4	TERM	46	1	84.14	84.14	84.14	NA	84.14	84.14	84.14
LNnT	G4	TERM	47	1	104.1	104.1	104.1	NA	104.1	104.1	104.1

Table S4 Concentration of Human Milk Oligosaccharides in Term or Preterm Milk At Specified Postmenstrual Age Separated By Milk Group

** When there are results below the method limit of quanfification (LoQ) the result has been assigned value of 0.5 × LoQ, hence the minimum value appears to be the same in many cases. When a large number of datapoints are below LoQ this can also have the effect that the median = minimum.*

HMO	Milk Group	Study Arm	Postmenstrual Age (weeks)	N	HMO Concentration (mg/L) min *	max	mean	sd	median *	Quartile 1 (25%)	Quartile 3 (75%)
LNT	G1	PRE-TERM	30	7	630.4	2002	934.4	481.8	819.4	675.1	869.2
LNT	G1	PRE-TERM	31	9	1040	1821	1330	305.4	1186	1105	1677
LNT	G1	PRE-TERM	32	13	298.8	2157	1158	509.4	997.4	806.7	1475
LNT	G1	PRE-TERM	33	18	341.5	1937	1048	405.3	1033	829.0	1212
LNT	G1	PRE-TERM	34	19	653.0	2851	1180	580.3	1000	831.1	1263
LNT	G1	PRE-TERM	35	18	479.3	2428	1198	573.8	1039	835.5	1573
LNT	G1	PRE-TERM	36	19	469.1	2737	1116	622.3	1030	600.5	1486
LNT	G1	PRE-TERM	37	18	356.6	2468	992.3	532.9	993.0	553.7	1231
LNT	G1	PRE-TERM	38	11	144.1	1498	692.3	469.2	635.4	303.8	896.7
LNT	G1	PRE-TERM	39	16	332.2	1829	871.5	490.6	658.0	537.0	1228
LNT	G1	PRE-TERM	40	7	317.3	1960	901.1	635.6	578.3	410.1	1316
LNT	G1	PRE-TERM	41	12	175.3	1283	634.6	348.2	632.0	332.4	895.0
LNT	G1	PRE-TERM	42	6	287.6	1271	759.4	400.8	800.8	418.2	1029
LNT	G1	PRE-TERM	43	10	304.6	1191	758.4	332.7	687.4	515.5	1092
LNT	G1	PRE-TERM	44	5	301.3	879.3	665.0	250.0	763.9	516.0	864.4
LNT	G1	PRE-TERM	45	9	145.2	1057	624.3	310.2	769.3	422.8	827.3
LNT	G1	PRE-TERM	46	6	232.8	1011	563.1	278.3	541.5	381.3	678.4
LNT	G1	PRE-TERM	47	3	248.2	773.5	569.6	281.7	687.1	467.6	730.3
LNT	G1	PRE-TERM	48	4	205.0	879.6	481.3	319.2	420.2	233.1	668.4
LNT	G1	TERM	38	2	598.9	923.2	761.1	229.3	761.1	680.0	842.1
LNT	G1	TERM	39	9	199.8	1223	631.9	382.6	549.4	356.9	964.5
LNT	G1	TERM	40	13	214.3	1580	1035	375.4	1178	809.8	1282
LNT	G1	TERM	41	21	515.1	1924	1113	405.1	1154	683.8	1399
LNT	G1	TERM	42	21	590.5	1862	1142	345.8	1165	919.7	1305
LNT	G1	TERM	43	21	359.8	1894	1021	413.2	1074	664.9	1272
LNT	G1	TERM	44	20	313.1	1536	884.8	396.5	797.3	586.6	1271
LNT	G1	TERM	45	21	307.2	1392	806.8	344.8	758.9	568.3	1002
LNT	G1	TERM	46	17	213.7	1567	802.3	433.0	749.2	362.2	1110
LNT	G1	TERM	47	12	217.7	1498	786.9	413.3	740.0	482.2	1084
LNT	G1	TERM	48	6	269.1	856.9	562.8	245.2	582.5	357.2	748.5
LNT	G2	PRE-TERM	29	1	3029	3029	3029	NA	3029	3029	3029
LNT	G2	PRE-TERM	30	2	1428	3296	2362	1321	2362	1895	2829
LNT	G2	PRE-TERM	31	2	2029	2903	2466	617.9	2466	2247	2684
LNT	G2	PRE-TERM	32	3	1901	2283	2029	220.2	1902	1901	2092
LNT	G2	PRE-TERM	33	5	1825	2537	2120	295.0	2061	1887	2290
LNT	G2	PRE-TERM	34	5	1613	2810	2002	492.3	1867	1628	2091

Table S4 Concentration of Human Milk Oligosaccharides in Term or Preterm Milk At Specified Postmenstrual Age Separated By Milk Group

** When there are results below the method limit of quanfification (LoQ) the result has been assigned value of 0.5 × LoQ, hence the minimum value appears to be the same in many cases. When a large number of datapoints are below LoQ this can also have the effect that the median = minimum.*

					HMO Concentration (mg/L)						
HMO	Milk Group	Study Arm	Postmenstrual Age (weeks)	N	min *	max	mean	sd	median *	Quartile 1 (25%)	Quartile 3 (75%)
LNT	G2	PRE-TERM	35	5	1428	2498	1932	483.4	1854	1519	2361
LNT	G2	PRE-TERM	36	5	1389	2397	1901	465.9	1775	1563	2381
LNT	G2	PRE-TERM	37	4	1119	1831	1499	330.6	1523	1279	1742
LNT	G2	PRE-TERM	38	4	1110	1942	1552	434.1	1579	1215	1916
LNT	G2	PRE-TERM	39	3	918.0	2376	1685	732.0	1762	1340	2069
LNT	G2	PRE-TERM	40	3	992.2	2184	1429	657.0	1109	1051	1647
LNT	G2	PRE-TERM	41	2	898.7	1554	1226	463.4	1226	1063	1390
LNT	G2	PRE-TERM	42	3	704.7	1221	945.1	260.1	909.4	807.0	1065
LNT	G2	PRE-TERM	43	2	312.5	775.7	544.1	327.6	544.1	428.3	659.9
LNT	G2	PRE-TERM	44	3	831.5	1051	965.3	117.4	1013	922.4	1032
LNT	G2	PRE-TERM	45	2	401.3	2113	1257	1210	1257	829.2	1685
LNT	G2	PRE-TERM	46	2	447.8	1162	805.0	505.1	805.0	626.4	983.6
LNT	G2	PRE-TERM	48	1	816.9	816.9	816.9	NA	816.9	816.9	816.9
LNT	G2	TERM	39	1	1253	1253	1253	NA	1253	1253	1253
LNT	G2	TERM	40	5	1074	2478	1765	630.2	1994	1130	2148
LNT	G2	TERM	41	4	904.3	2056	1714	548.4	1947	1609	2051
LNT	G2	TERM	42	6	985.8	2236	1656	413.2	1702	1526	1806
LNT	G2	TERM	43	5	905.7	2354	1562	569.5	1346	1294	1909
LNT	G2	TERM	44	5	726.0	2342	1411	751.4	942.4	939.1	2104
LNT	G2	TERM	45	5	342.4	1986	1130	747.3	792.0	659.9	1869
LNT	G2	TERM	46	5	576.2	1405	952.7	340.8	813.3	764.7	1204
LNT	G2	TERM	47	4	454.4	1637	1089	574.3	1133	681.2	1541
LNT	G3	PRE-TERM	30	1	975.2	975.2	975.2	NA	975.2	975.2	975.2
LNT	G3	PRE-TERM	31	1	965.1	965.1	965.1	NA	965.1	965.1	965.1
LNT	G3	PRE-TERM	32	1	1112	1112	1112	NA	1112	1112	1112
LNT	G3	PRE-TERM	33	1	1195	1195	1195	NA	1195	1195	1195
LNT	G3	PRE-TERM	34	1	880.4	880.4	880.4	NA	880.4	880.4	880.4
LNT	G3	PRE-TERM	35	1	760.5	760.5	760.5	NA	760.5	760.5	760.5
LNT	G3	PRE-TERM	36	1	592.7	592.7	592.7	NA	592.7	592.7	592.7
LNT	G3	PRE-TERM	37	1	659.1	659.1	659.1	NA	659.1	659.1	659.1
LNT	G3	PRE-TERM	39	1	561.7	561.7	561.7	NA	561.7	561.7	561.7
LNT	G3	PRE-TERM	41	1	660.0	660.0	660.0	NA	660.0	660.0	660.0
LNT	G3	PRE-TERM	43	1	586.0	586.0	586.0	NA	586.0	586.0	586.0
LNT	G3	PRE-TERM	45	1	604.4	604.4	604.4	NA	604.4	604.4	604.4
LNT	G3	TERM	41	1	359.0	359.0	359.0	NA	359.0	359.0	359.0
LNT	G3	TERM	42	1	358.7	358.7	358.7	NA	358.7	358.7	358.7

Table S4 Concentration of Human Milk Oligosaccharides in Term or Preterm Milk At Specified Postmenstrual Age Separated By Milk Group

** When there are results below the method limit of quanfification (LoQ) the result has been assigned value of 0.5 × LoQ, hence the minimum value appears to be the same in many cases. When a large number of datapoints are below LoQ this can also have the effect that the median = minimum.*

					HMO Concentration (mg/L)						
HMO	Milk Group	Study Arm	Postmenstrual Age (weeks)	N	min *	max	mean	sd	median *	Quartile 1 (25%)	Quartile 3 (75%)
LNT	G3	TERM	43	1	588.9	588.9	588.9	NA	588.9	588.9	588.9
LNT	G3	TERM	44	1	484.2	484.2	484.2	NA	484.2	484.2	484.2
LNT	G3	TERM	45	1	572.1	572.1	572.1	NA	572.1	572.1	572.1
LNT	G3	TERM	46	1	394.1	394.1	394.1	NA	394.1	394.1	394.1
LNT	G3	TERM	47	1	417.8	417.8	417.8	NA	417.8	417.8	417.8
LNT	G3	TERM	48	1	307.1	307.1	307.1	NA	307.1	307.1	307.1
LNT	G4	TERM	40	1	3400	3400	3400	NA	3400	3400	3400
LNT	G4	TERM	41	1	3848	3848	3848	NA	3848	3848	3848
LNT	G4	TERM	42	1	3092	3092	3092	NA	3092	3092	3092
LNT	G4	TERM	43	1	3087	3087	3087	NA	3087	3087	3087
LNT	G4	TERM	44	1	2480	2480	2480	NA	2480	2480	2480
LNT	G4	TERM	45	1	2109	2109	2109	NA	2109	2109	2109
LNT	G4	TERM	46	1	2251	2251	2251	NA	2251	2251	2251
LNT	G4	TERM	47	1	1852	1852	1852	NA	1852	1852	1852
LSTb	G1	PRE-TERM	30	7	54.76	91.46	71.90	13.94	71.49	61.56	81.22
LSTb	G1	PRE-TERM	31	9	71.27	147.8	98.24	24.79	93.18	79.15	114.1
LSTb	G1	PRE-TERM	32	13	44.25	211.3	94.32	49.35	79.65	57.51	114.2
LSTb	G1	PRE-TERM	33	18	41.49	178.8	91.87	42.73	82.78	61.44	123.2
LSTb	G1	PRE-TERM	34	19	49.85	168.4	99.77	38.51	100.1	59.73	125.3
LSTb	G1	PRE-TERM	35	18	47.34	205.4	108.8	44.65	113.6	72.07	136.9
LSTb	G1	PRE-TERM	36	19	42.09	134.2	91.19	35.65	99.07	54.51	128.0
LSTb	G1	PRE-TERM	37	18	37.86	137.9	90.51	35.85	92.42	55.45	124.2
LSTb	G1	PRE-TERM	38	11	7.000	144.9	80.06	46.26	63.09	48.34	121.1
LSTb	G1	PRE-TERM	39	16	38.99	146.6	88.35	38.48	87.17	50.34	122.2
LSTb	G1	PRE-TERM	40	7	48.94	146.3	98.61	36.56	100.2	71.88	125.6
LSTb	G1	PRE-TERM	41	12	34.87	191.9	88.56	51.25	81.73	46.83	112.6
LSTb	G1	PRE-TERM	42	6	39.45	142.5	82.85	41.21	83.55	47.49	105.3
LSTb	G1	PRE-TERM	43	10	31.54	195.0	96.71	45.20	97.42	72.36	112.2
LSTb	G1	PRE-TERM	44	5	44.11	92.55	73.07	18.91	75.91	66.87	85.94
LSTb	G1	PRE-TERM	45	9	33.70	102.6	77.22	21.16	81.03	75.40	91.53
LSTb	G1	PRE-TERM	46	6	25.18	94.78	71.06	26.49	75.91	62.73	91.16
LSTb	G1	PRE-TERM	47	3	36.39	84.41	61.71	24.12	64.34	50.36	74.38
LSTb	G1	PRE-TERM	48	4	23.33	75.54	49.91	21.35	50.39	42.67	57.64
LSTb	G1	TERM	38	2	56.67	94.71	75.69	26.90	75.69	66.18	85.20
LSTb	G1	TERM	39	9	33.70	89.73	65.07	18.20	68.78	50.04	72.96
LSTb	G1	TERM	40	13	28.89	141.7	74.31	30.16	64.19	51.10	94.91

Table S4 Concentration of Human Milk Oligosaccharides in Term or Preterm Milk At Specified Postmenstrual Age Separated By Milk Group

** When there are results below the method limit of quanfification (LoQ) the result has been assigned value of 0.5 × LoQ, hence the minimum value appears to be the same in many cases. When a large number of datapoints are below LoQ this can also have the effect that the median = minimum.*

					HMO Concentration (mg/L)						
HMO	Milk Group	Study Arm	Postmenstrual Age (weeks)	N	min *	max	mean	sd	median *	Quartile 1 (25%)	Quartile 3 (75%)
LSTb	G1	TERM	41	21	36.75	149.1	78.11	30.45	70.83	54.29	98.29
LSTb	G1	TERM	42	21	32.58	163.7	80.98	31.85	77.06	55.35	98.91
LSTb	G1	TERM	43	21	36.28	127.3	78.62	27.72	77.58	56.56	95.80
LSTb	G1	TERM	44	20	20.15	166.6	76.40	36.46	71.56	55.13	98.66
LSTb	G1	TERM	45	21	7.000	147.4	67.05	32.09	62.38	43.69	89.22
LSTb	G1	TERM	46	17	18.33	177.0	76.75	45.11	68.29	43.19	100.6
LSTb	G1	TERM	47	12	7.000	152.6	70.54	49.21	61.18	27.55	115.5
LSTb	G1	TERM	48	6	22.20	119.2	67.21	37.68	69.22	36.92	90.22
LSTb	G2	PRE-TERM	29	1	183.1	183.1	183.1	NA	183.1	183.1	183.1
LSTb	G2	PRE-TERM	30	2	125.0	159.2	142.1	24.15	142.1	133.6	150.7
LSTb	G2	PRE-TERM	31	2	148.0	182.1	165.0	24.06	165.0	156.5	173.6
LSTb	G2	PRE-TERM	32	3	100.9	217.4	157.2	58.37	153.2	127.1	185.3
LSTb	G2	PRE-TERM	33	5	106.0	256.1	184.3	58.35	188.4	151.4	219.5
LSTb	G2	PRE-TERM	34	5	96.85	334.7	171.6	94.74	145.4	117.2	163.7
LSTb	G2	PRE-TERM	35	5	91.92	314.7	164.9	88.05	151.3	110.3	156.2
LSTb	G2	PRE-TERM	36	5	78.90	423.9	184.9	141.1	133.0	92.87	195.8
LSTb	G2	PRE-TERM	37	4	110.4	400.7	198.3	135.8	141.0	131.9	207.3
LSTb	G2	PRE-TERM	38	4	66.06	161.7	112.0	41.60	110.2	86.11	136.1
LSTb	G2	PRE-TERM	39	3	74.62	156.5	110.4	41.90	100.0	87.33	128.3
LSTb	G2	PRE-TERM	40	3	55.66	135.2	100.2	40.63	109.8	82.73	122.5
LSTb	G2	PRE-TERM	41	2	77.14	405.6	241.3	232.2	241.3	159.2	323.4
LSTb	G2	PRE-TERM	42	3	51.40	143.0	93.36	46.26	85.72	68.56	114.3
LSTb	G2	PRE-TERM	43	2	54.05	243.3	148.7	133.8	148.7	101.4	196.0
LSTb	G2	PRE-TERM	44	3	43.51	140.5	88.03	49.01	80.04	61.78	110.3
LSTb	G2	PRE-TERM	45	2	85.64	247.3	166.5	114.3	166.5	126.1	206.9
LSTb	G2	PRE-TERM	46	2	77.72	114.5	96.10	26.00	96.10	86.91	105.3
LSTb	G2	PRE-TERM	48	1	106.9	106.9	106.9	NA	106.9	106.9	106.9
LSTb	G2	TERM	39	1	102.3	102.3	102.3	NA	102.3	102.3	102.3
LSTb	G2	TERM	40	5	51.20	196.6	107.1	57.30	99.12	65.94	122.7
LSTb	G2	TERM	41	4	52.81	108.2	84.50	26.65	88.51	67.32	105.7
LSTb	G2	TERM	42	6	57.67	124.3	85.01	26.67	78.53	64.71	102.8
LSTb	G2	TERM	43	5	42.44	106.8	77.62	26.02	85.80	59.96	93.06
LSTb	G2	TERM	44	5	46.78	120.7	81.75	32.80	70.91	58.51	111.8
LSTb	G2	TERM	45	5	15.28	141.7	78.97	49.51	83.89	47.10	106.9
LSTb	G2	TERM	46	5	27.92	104.2	63.54	28.36	65.64	48.44	71.49
LSTb	G2	TERM	47	4	20.65	119.5	68.36	40.46	66.65	53.29	81.73

Table S4 Concentration of Human Milk Oligosaccharides in Term or Preterm Milk At Specified Postmenstrual Age Separated By Milk Group

** When there are results below the method limit of quanfification (LoQ) the result has been assigned value of 0.5 × LoQ, hence the minimum value appears to be the same in many cases. When a large number of datapoints are below LoQ this can also have the effect that the median = minimum.*

					HMO Concentration (mg/L)						
**HMO**	**Milk Group**	**Study Arm**	**Postmenstrual Age (weeks)**	**N**	**min ***	**max**	**mean**	**sd**	**median ***	**Quartile 1 (25%)**	**Quartile 3 (75%)**
LSTb	G3	PRE-TERM	30	1	97.28	97.28	97.28	NA	97.28	97.28	97.28
LSTb	G3	PRE-TERM	31	1	161.6	161.6	161.6	NA	161.6	161.6	161.6
LSTb	G3	PRE-TERM	32	1	151.5	151.5	151.5	NA	151.5	151.5	151.5
LSTb	G3	PRE-TERM	33	1	107.7	107.7	107.7	NA	107.7	107.7	107.7
LSTb	G3	PRE-TERM	34	1	86.22	86.22	86.22	NA	86.22	86.22	86.22
LSTb	G3	PRE-TERM	35	1	67.69	67.69	67.69	NA	67.69	67.69	67.69
LSTb	G3	PRE-TERM	36	1	59.00	59.00	59.00	NA	59.00	59.00	59.00
LSTb	G3	PRE-TERM	37	1	54.27	54.27	54.27	NA	54.27	54.27	54.27
LSTb	G3	PRE-TERM	39	1	42.77	42.77	42.77	NA	42.77	42.77	42.77
LSTb	G3	PRE-TERM	41	1	52.23	52.23	52.23	NA	52.23	52.23	52.23
LSTb	G3	PRE-TERM	43	1	53.04	53.04	53.04	NA	53.04	53.04	53.04
LSTb	G3	PRE-TERM	45	1	53.45	53.45	53.45	NA	53.45	53.45	53.45
LSTb	G3	TERM	41	1	15.52	15.52	15.52	NA	15.52	15.52	15.52
LSTb	G3	TERM	42	1	40.88	40.88	40.88	NA	40.88	40.88	40.88
LSTb	G3	TERM	43	1	46.68	46.68	46.68	NA	46.68	46.68	46.68
LSTb	G3	TERM	44	1	54.30	54.30	54.30	NA	54.30	54.30	54.30
LSTb	G3	TERM	45	1	50.89	50.89	50.89	NA	50.89	50.89	50.89
LSTb	G3	TERM	46	1	46.48	46.48	46.48	NA	46.48	46.48	46.48
LSTb	G3	TERM	47	1	40.09	40.09	40.09	NA	40.09	40.09	40.09
LSTb	G3	TERM	48	1	45.28	45.28	45.28	NA	45.28	45.28	45.28
LSTb	G4	TERM	40	1	168.8	168.8	168.8	NA	168.8	168.8	168.8
LSTb	G4	TERM	41	1	160.5	160.5	160.5	NA	160.5	160.5	160.5
LSTb	G4	TERM	42	1	115.0	115.0	115.0	NA	115.0	115.0	115.0
LSTb	G4	TERM	43	1	124.3	124.3	124.3	NA	124.3	124.3	124.3
LSTb	G4	TERM	44	1	107.1	107.1	107.1	NA	107.1	107.1	107.1
LSTb	G4	TERM	45	1	136.3	136.3	136.3	NA	136.3	136.3	136.3
LSTb	G4	TERM	46	1	100.9	100.9	100.9	NA	100.9	100.9	100.9
LSTb	G4	TERM	47	1	97.38	97.38	97.38	NA	97.38	97.38	97.38
LSTc	G1	PRE-TERM	30	7	139.5	747.9	466.9	234.6	568.2	290.1	616.5
LSTc	G1	PRE-TERM	31	9	37.98	629.5	372.9	210.7	415.5	197.0	536.2
LSTc	G1	PRE-TERM	32	13	31.79	1019	373.4	249.6	325.3	266.4	505.4
LSTc	G1	PRE-TERM	33	18	28.75	767.6	278.2	202.6	229.6	172.6	299.1
LSTc	G1	PRE-TERM	34	19	52.17	622.9	228.8	145.4	202.1	133.0	263.2
LSTc	G1	PRE-TERM	35	18	71.06	402.8	184.3	94.67	170.2	114.1	230.0
LSTc	G1	PRE-TERM	36	19	45.27	371.1	153.9	90.37	153.8	90.20	182.8
LSTc	G1	PRE-TERM	37	18	49.15	355.6	137.5	79.52	123.4	93.96	151.7

Table S4 Concentration of Human Milk Oligosaccharides in Term or Preterm Milk At Specified Postmenstrual Age Separated By Milk Group

** When there are results below the method limit of quanfification (LoQ) the result has been assigned value of 0.5 × LoQ, hence the minimum value appears to be the same in many cases. When a large number of datapoints are below LoQ this can also have the effect that the median = minimum.*

					HMO Concentration (mg/L)						
HMO	Milk Group	Study Arm	Postmenstrual Age (weeks)	N	min *	max	mean	sd	median *	Quartile 1 (25%)	Quartile 3 (75%)
LSTc	G1	PRE-TERM	38	11	20.21	211.2	109.6	60.36	100.7	73.21	146.6
LSTc	G1	PRE-TERM	39	16	42.87	211.9	101.3	49.15	81.25	64.69	120.4
LSTc	G1	PRE-TERM	40	7	61.08	151.3	93.35	34.97	94.65	61.32	111.9
LSTc	G1	PRE-TERM	41	12	26.24	209.1	78.50	46.77	74.81	50.74	84.57
LSTc	G1	PRE-TERM	42	6	46.32	125.3	71.96	30.67	61.96	49.32	83.84
LSTc	G1	PRE-TERM	43	10	10.91	91.88	43.52	22.87	39.93	32.40	52.66
LSTc	G1	PRE-TERM	44	5	34.47	100.6	66.66	29.08	64.75	42.32	91.13
LSTc	G1	PRE-TERM	45	9	9.258	102.5	39.78	34.11	24.93	15.41	62.63
LSTc	G1	PRE-TERM	46	6	22.75	68.68	46.54	18.40	47.81	32.89	60.14
LSTc	G1	PRE-TERM	47	3	12.11	96.78	44.70	45.57	25.21	18.66	61.00
LSTc	G1	PRE-TERM	48	4	14.03	67.61	37.09	24.43	33.36	19.36	51.09
LSTc	G1	TERM	38	2	329.6	575.3	452.4	173.7	452.4	391.0	513.9
LSTc	G1	TERM	39	9	215.4	1199	571.2	321.7	468.6	344.4	657.9
LSTc	G1	TERM	40	13	36.80	1169	555.8	366.1	461.0	301.8	743.7
LSTc	G1	TERM	41	21	108.9	779.2	466.0	183.9	497.8	391.5	530.5
LSTc	G1	TERM	42	21	82.02	524.8	313.8	112.0	349.3	218.2	391.9
LSTc	G1	TERM	43	21	71.57	367.7	226.2	84.17	232.1	153.9	295.8
LSTc	G1	TERM	44	20	63.36	254.9	163.0	49.50	166.0	125.2	199.9
LSTc	G1	TERM	45	21	50.54	282.9	144.0	70.56	120.9	96.17	182.7
LSTc	G1	TERM	46	17	30.28	261.1	122.3	58.51	108.7	92.94	126.1
LSTc	G1	TERM	47	12	36.24	221.6	108.4	58.06	76.99	70.23	159.6
LSTc	G1	TERM	48	6	37.33	141.6	68.14	37.05	58.68	52.64	61.13
LSTc	G2	PRE-TERM	29	1	184.0	184.0	184.0	NA	184.0	184.0	184.0
LSTc	G2	PRE-TERM	30	2	95.07	132.4	113.7	26.38	113.7	104.4	123.1
LSTc	G2	PRE-TERM	31	2	55.00	142.3	98.65	61.73	98.65	76.83	120.5
LSTc	G2	PRE-TERM	32	3	41.80	182.8	107.2	71.07	97.02	69.41	139.9
LSTc	G2	PRE-TERM	33	5	30.60	693.6	262.4	264.1	176.2	92.70	318.9
LSTc	G2	PRE-TERM	34	5	24.49	248.2	140.3	98.04	101.3	90.38	237.1
LSTc	G2	PRE-TERM	35	5	23.78	233.7	120.8	83.88	90.27	78.40	177.8
LSTc	G2	PRE-TERM	36	5	17.24	129.5	76.22	42.74	75.30	57.53	101.6
LSTc	G2	PRE-TERM	37	4	17.82	119.0	68.34	41.88	68.26	49.36	87.24
LSTc	G2	PRE-TERM	38	4	36.77	110.0	69.22	32.17	65.09	47.97	86.35
LSTc	G2	PRE-TERM	39	3	22.11	67.76	47.02	23.11	51.20	36.65	59.48
LSTc	G2	PRE-TERM	40	3	36.31	58.60	46.15	11.37	43.54	39.93	51.07
LSTc	G2	PRE-TERM	41	2	28.57	33.53	31.05	3.512	31.05	29.81	32.29
LSTc	G2	PRE-TERM	42	3	28.83	47.41	38.61	9.330	39.60	34.21	43.50

Table S4 Concentration of Human Milk Oligosaccharides in Term or Preterm Milk At Specified Postmenstrual Age Separated By Milk Group

** When there are results below the method limit of quanfification (LoQ) the result has been assigned value of 0.5 × LoQ, hence the minimum value appears to be the same in many cases. When a large number of datapoints are below LoQ this can also have the effect that the median = minimum.*

HMO	Milk Group	Study Arm	Postmenstrual Age (weeks)	N	HMO Concentration (mg/L) min *	max	mean	sd	median *	Quartile 1 (25%)	Quartile 3 (75%)
LSTc	G2	PRE-TERM	43	2	15.48	35.01	25.25	13.81	25.25	20.36	30.13
LSTc	G2	PRE-TERM	44	3	22.05	30.95	27.14	4.584	28.41	25.23	29.68
LSTc	G2	PRE-TERM	45	2	11.60	22.43	17.02	7.660	17.02	14.31	19.73
LSTc	G2	PRE-TERM	46	2	25.88	35.59	30.73	6.861	30.73	28.31	33.16
LSTc	G2	PRE-TERM	48	1	18.59	18.59	18.59	NA	18.59	18.59	18.59
LSTc	G2	TERM	39	1	517.1	517.1	517.1	NA	517.1	517.1	517.1
LSTc	G2	TERM	40	5	204.1	761.3	428.2	219.8	347.0	302.5	526.1
LSTc	G2	TERM	41	4	103.3	398.1	274.9	127.5	299.0	221.0	352.9
LSTc	G2	TERM	42	6	64.34	316.4	192.9	85.25	193.9	158.4	230.1
LSTc	G2	TERM	43	5	32.73	151.8	108.3	45.47	114.3	109.1	133.4
LSTc	G2	TERM	44	5	30.19	129.5	85.86	38.83	86.73	69.49	113.4
LSTc	G2	TERM	45	5	20.07	135.6	75.93	47.17	91.63	36.34	95.98
LSTc	G2	TERM	46	5	16.92	91.63	51.68	27.40	52.74	39.15	57.98
LSTc	G2	TERM	47	4	37.96	86.93	55.52	21.91	48.61	42.19	61.94
LSTc	G3	PRE-TERM	30	1	526.1	526.1	526.1	NA	526.1	526.1	526.1
LSTc	G3	PRE-TERM	31	1	303.7	303.7	303.7	NA	303.7	303.7	303.7
LSTc	G3	PRE-TERM	32	1	136.2	136.2	136.2	NA	136.2	136.2	136.2
LSTc	G3	PRE-TERM	33	1	87.33	87.33	87.33	NA	87.33	87.33	87.33
LSTc	G3	PRE-TERM	34	1	62.73	62.73	62.73	NA	62.73	62.73	62.73
LSTc	G3	PRE-TERM	35	1	71.54	71.54	71.54	NA	71.54	71.54	71.54
LSTc	G3	PRE-TERM	36	1	42.20	42.20	42.20	NA	42.20	42.20	42.20
LSTc	G3	PRE-TERM	37	1	38.50	38.50	38.50	NA	38.50	38.50	38.50
LSTc	G3	PRE-TERM	39	1	24.55	24.55	24.55	NA	24.55	24.55	24.55
LSTc	G3	PRE-TERM	41	1	20.28	20.28	20.28	NA	20.28	20.28	20.28
LSTc	G3	PRE-TERM	43	1	24.47	24.47	24.47	NA	24.47	24.47	24.47
LSTc	G3	PRE-TERM	45	1	17.33	17.33	17.33	NA	17.33	17.33	17.33
LSTc	G3	TERM	41	1	853.8	853.8	853.8	NA	853.8	853.8	853.8
LSTc	G3	TERM	42	1	298.2	298.2	298.2	NA	298.2	298.2	298.2
LSTc	G3	TERM	43	1	193.2	193.2	193.2	NA	193.2	193.2	193.2
LSTc	G3	TERM	44	1	139.1	139.1	139.1	NA	139.1	139.1	139.1
LSTc	G3	TERM	45	1	97.32	97.32	97.32	NA	97.32	97.32	97.32
LSTc	G3	TERM	46	1	73.72	73.72	73.72	NA	73.72	73.72	73.72
LSTc	G3	TERM	47	1	62.70	62.70	62.70	NA	62.70	62.70	62.70
LSTc	G3	TERM	48	1	76.90	76.90	76.90	NA	76.90	76.90	76.90
LSTc	G4	TERM	40	1	979.8	979.8	979.8	NA	979.8	979.8	979.8
LSTc	G4	TERM	41	1	1156	1156	1156	NA	1156	1156	1156

Table S4 Concentration of Human Milk Oligosaccharides in Term or Preterm Milk At Specified Postmenstrual Age Separated By Milk Group

** When there are results below the method limit of quanfification (LoQ) the result has been assigned value of 0.5 × LoQ, hence the minimum value appears to be the same in many cases. When a large number of datapoints are below LoQ this can also have the effect that the median = minimum.*

					HMO Concentration (mg/L)						
HMO	Milk Group	Study Arm	Postmenstrual Age (weeks)	N	min *	max	mean	sd	median *	Quartile 1 (25%)	Quartile 3 (75%)
LSTc	G4	TERM	42	1	620.3	620.3	620.3	NA	620.3	620.3	620.3
LSTc	G4	TERM	43	1	346.2	346.2	346.2	NA	346.2	346.2	346.2
LSTc	G4	TERM	44	1	188.7	188.7	188.7	NA	188.7	188.7	188.7
LSTc	G4	TERM	45	1	160.6	160.6	160.6	NA	160.6	160.6	160.6
LSTc	G4	TERM	46	1	96.53	96.53	96.53	NA	96.53	96.53	96.53
LSTc	G4	TERM	47	1	57.77	57.77	57.77	NA	57.77	57.77	57.77
MFLNH-III	G1	PRE-TERM	30	7	70.38	280.1	183.4	75.91	218.0	127.7	229.9
MFLNH-III	G1	PRE-TERM	31	9	58.75	649.8	360.5	190.1	336.7	231.3	508.1
MFLNH-III	G1	PRE-TERM	32	13	98.01	663.0	344.6	167.5	325.6	226.5	423.2
MFLNH-III	G1	PRE-TERM	33	18	71.20	604.0	323.0	163.7	338.0	196.2	424.1
MFLNH-III	G1	PRE-TERM	34	19	65.81	575.2	287.8	147.9	290.7	170.4	386.5
MFLNH-III	G1	PRE-TERM	35	18	64.26	445.5	265.8	97.04	263.7	225.8	335.9
MFLNH-III	G1	PRE-TERM	36	19	105.8	508.6	296.0	98.03	306.7	226.5	347.8
MFLNH-III	G1	PRE-TERM	37	18	86.37	422.1	252.0	92.90	240.4	184.9	323.8
MFLNH-III	G1	PRE-TERM	38	11	64.97	343.6	184.4	84.16	152.3	131.1	245.0
MFLNH-III	G1	PRE-TERM	39	16	58.71	294.7	174.9	82.30	162.5	123.6	260.6
MFLNH-III	G1	PRE-TERM	40	7	79.18	420.5	198.3	125.8	143.5	104.7	267.9
MFLNH-III	G1	PRE-TERM	41	12	17.50	271.6	95.13	63.96	92.70	56.82	110.9
MFLNH-III	G1	PRE-TERM	42	6	50.65	321.8	176.6	93.50	152.0	143.4	219.9
MFLNH-III	G1	PRE-TERM	43	10	17.50	323.4	107.1	83.81	87.46	66.40	117.9
MFLNH-III	G1	PRE-TERM	44	5	81.42	176.8	131.4	39.78	126.0	107.3	165.5
MFLNH-III	G1	PRE-TERM	45	9	17.50	199.1	81.13	53.67	75.23	46.68	81.30
MFLNH-III	G1	PRE-TERM	46	6	68.19	215.4	112.4	52.73	101.1	84.55	107.4
MFLNH-III	G1	PRE-TERM	47	3	49.98	185.3	107.4	69.94	86.82	68.40	136.0
MFLNH-III	G1	PRE-TERM	48	4	17.50	193.4	89.14	75.45	72.82	46.67	115.3
MFLNH-III	G1	TERM	38	2	137.0	188.4	162.7	36.40	162.7	149.8	175.6
MFLNH-III	G1	TERM	39	9	17.50	469.8	196.2	160.4	144.4	66.26	325.9
MFLNH-III	G1	TERM	40	13	90.03	613.8	266.8	144.8	236.2	152.2	332.4
MFLNH-III	G1	TERM	41	21	121.3	578.2	308.8	110.7	327.9	201.0	369.3
MFLNH-III	G1	TERM	42	21	194.2	552.7	354.1	100.2	332.1	309.3	408.7
MFLNH-III	G1	TERM	43	21	81.47	571.1	316.9	127.6	312.7	231.1	388.2
MFLNH-III	G1	TERM	44	20	17.50	483.7	278.1	128.4	292.2	187.7	350.3
MFLNH-III	G1	TERM	45	21	17.50	417.6	244.4	109.7	245.1	151.0	314.5
MFLNH-III	G1	TERM	46	17	17.50	443.9	222.3	99.15	216.6	151.6	281.6
MFLNH-III	G1	TERM	47	12	57.71	382.8	219.9	88.06	207.6	160.9	284.6
MFLNH-III	G1	TERM	48	6	108.5	289.1	197.3	61.11	193.6	172.3	223.7

Table S4 Concentration of Human Milk Oligosaccharides in Term or Preterm Milk At Specified Postmenstrual Age Separated By Milk Group

** When there are results below the method limit of quanfification (LoQ) the result has been assigned value of 0.5 × LoQ, hence the minimum value appears to be the same in many cases. When a large number of datapoints are below LoQ this can also have the effect that the median = minimum.*

					HMO Concentration (mg/L)						
HMO	Milk Group	Study Arm	Postmenstrual Age (weeks)	N	min *	max	mean	sd	median *	Quartile 1 (25%)	Quartile 3 (75%)
MFLNH-III	G2	PRE-TERM	29	1	166.0	166.0	166.0	NA	166.0	166.0	166.0
MFLNH-III	G2	PRE-TERM	30	2	186.2	437.6	311.9	177.7	311.9	249.1	374.8
MFLNH-III	G2	PRE-TERM	31	2	147.0	724.8	435.9	408.5	435.9	291.5	580.3
MFLNH-III	G2	PRE-TERM	32	3	165.4	743.2	367.6	325.6	194.1	179.8	468.7
MFLNH-III	G2	PRE-TERM	33	5	141.6	820.2	426.5	259.6	327.0	313.5	530.3
MFLNH-III	G2	PRE-TERM	34	5	101.4	856.2	513.3	294.1	517.0	377.6	714.1
MFLNH-III	G2	PRE-TERM	35	5	116.0	790.5	492.3	280.4	454.4	355.5	745.1
MFLNH-III	G2	PRE-TERM	36	5	77.34	753.0	464.6	259.3	454.7	397.0	640.9
MFLNH-III	G2	PRE-TERM	37	4	66.95	438.0	311.1	166.4	369.8	282.3	398.6
MFLNH-III	G2	PRE-TERM	38	4	304.9	707.1	471.0	187.1	436.1	329.6	577.5
MFLNH-III	G2	PRE-TERM	39	3	261.1	492.1	409.9	129.0	476.4	368.8	484.2
MFLNH-III	G2	PRE-TERM	40	3	286.7	551.6	415.9	132.6	409.3	348.0	480.5
MFLNH-III	G2	PRE-TERM	41	2	78.50	279.2	178.8	141.9	178.8	128.7	229.0
MFLNH-III	G2	PRE-TERM	42	3	221.7	386.3	284.5	88.91	245.5	233.6	315.9
MFLNH-III	G2	PRE-TERM	43	2	50.63	58.13	54.38	5.299	54.38	52.51	56.25
MFLNH-III	G2	PRE-TERM	44	3	144.9	337.9	257.1	100.3	288.3	216.6	313.1
MFLNH-III	G2	PRE-TERM	45	2	53.16	123.9	88.51	49.99	88.51	70.84	106.2
MFLNH-III	G2	PRE-TERM	46	2	53.20	324.5	188.8	191.8	188.8	121.0	256.6
MFLNH-III	G2	PRE-TERM	48	1	231.9	231.9	231.9	NA	231.9	231.9	231.9
MFLNH-III	G2	TERM	39	1	169.0	169.0	169.0	NA	169.0	169.0	169.0
MFLNH-III	G2	TERM	40	5	17.50	385.1	231.6	148.1	280.6	148.4	326.4
MFLNH-III	G2	TERM	41	4	312.8	772.4	617.3	206.6	692.0	591.9	717.4
MFLNH-III	G2	TERM	42	6	339.0	861.7	664.8	185.8	729.0	603.3	758.8
MFLNH-III	G2	TERM	43	5	222.9	976.4	711.0	287.8	806.8	722.0	826.8
MFLNH-III	G2	TERM	44	5	194.8	886.4	593.0	249.4	620.9	617.0	645.6
MFLNH-III	G2	TERM	45	5	140.6	758.8	405.4	232.2	393.8	273.3	460.5
MFLNH-III	G2	TERM	46	5	17.50	600.2	325.3	232.1	409.7	163.9	435.4
MFLNH-III	G2	TERM	47	4	314.1	875.6	494.8	256.9	394.8	365.3	524.2
MFLNH-III	G3	PRE-TERM	30	1	452.0	452.0	452.0	NA	452.0	452.0	452.0
MFLNH-III	G3	PRE-TERM	31	1	330.7	330.7	330.7	NA	330.7	330.7	330.7
MFLNH-III	G3	PRE-TERM	32	1	314.2	314.2	314.2	NA	314.2	314.2	314.2
MFLNH-III	G3	PRE-TERM	33	1	467.1	467.1	467.1	NA	467.1	467.1	467.1
MFLNH-III	G3	PRE-TERM	34	1	454.7	454.7	454.7	NA	454.7	454.7	454.7
MFLNH-III	G3	PRE-TERM	35	1	443.4	443.4	443.4	NA	443.4	443.4	443.4
MFLNH-III	G3	PRE-TERM	36	1	370.4	370.4	370.4	NA	370.4	370.4	370.4
MFLNH-III	G3	PRE-TERM	37	1	334.9	334.9	334.9	NA	334.9	334.9	334.9

Table S4 Concentration of Human Milk Oligosaccharides in Term or Preterm Milk At Specified Postmenstrual Age Separated By Milk Group

** When there are results below the method limit of quanfification (LoQ) the result has been assigned value of 0.5 × LoQ, hence the minimum value appears to be the same in many cases. When a large number of datapoints are below LoQ this can also have the effect that the median = minimum.*

					HMO Concentration (mg/L)						
**HMO**	**Milk Group**	**Study Arm**	**Postmenstrual Age (weeks)**	**N**	**min ***	**max**	**mean**	**sd**	**median ***	**Quartile 1 (25%)**	**Quartile 3 (75%)**
MFLNH-III	G3	PRE-TERM	39	1	246.6	246.6	246.6	NA	246.6	246.6	246.6
MFLNH-III	G3	PRE-TERM	41	1	180.9	180.9	180.9	NA	180.9	180.9	180.9
MFLNH-III	G3	PRE-TERM	43	1	140.0	140.0	140.0	NA	140.0	140.0	140.0
MFLNH-III	G3	PRE-TERM	45	1	102.0	102.0	102.0	NA	102.0	102.0	102.0
MFLNH-III	G3	TERM	41	1	524.0	524.0	524.0	NA	524.0	524.0	524.0
MFLNH-III	G3	TERM	42	1	493.2	493.2	493.2	NA	493.2	493.2	493.2
MFLNH-III	G3	TERM	43	1	593.9	593.9	593.9	NA	593.9	593.9	593.9
MFLNH-III	G3	TERM	44	1	496.9	496.9	496.9	NA	496.9	496.9	496.9
MFLNH-III	G3	TERM	45	1	574.6	574.6	574.6	NA	574.6	574.6	574.6
MFLNH-III	G3	TERM	46	1	355.1	355.1	355.1	NA	355.1	355.1	355.1
MFLNH-III	G3	TERM	47	1	369.9	369.9	369.9	NA	369.9	369.9	369.9
MFLNH-III	G3	TERM	48	1	299.2	299.2	299.2	NA	299.2	299.2	299.2
MFLNH-III	G4	TERM	40	1	189.9	189.9	189.9	NA	189.9	189.9	189.9
MFLNH-III	G4	TERM	41	1	695.9	695.9	695.9	NA	695.9	695.9	695.9
MFLNH-III	G4	TERM	42	1	934.4	934.4	934.4	NA	934.4	934.4	934.4
MFLNH-III	G4	TERM	43	1	895.2	895.2	895.2	NA	895.2	895.2	895.2
MFLNH-III	G4	TERM	44	1	872.4	872.4	872.4	NA	872.4	872.4	872.4
MFLNH-III	G4	TERM	45	1	513.6	513.6	513.6	NA	513.6	513.6	513.6
MFLNH-III	G4	TERM	46	1	571.8	571.8	571.8	NA	571.8	571.8	571.8
MFLNH-III	G4	TERM	47	1	427.0	427.0	427.0	NA	427.0	427.0	427.0
