# Supplementary figures and images for: Human Milk Oligosaccharides in the Milk of Mothers Delivering Term versus Preterm Infants
Source: Nutrients. 2019 Jun 5;11(6):1282. doi: 10.3390/nu11061282 (PMC6627155; doi:10.3390/nu11061282)

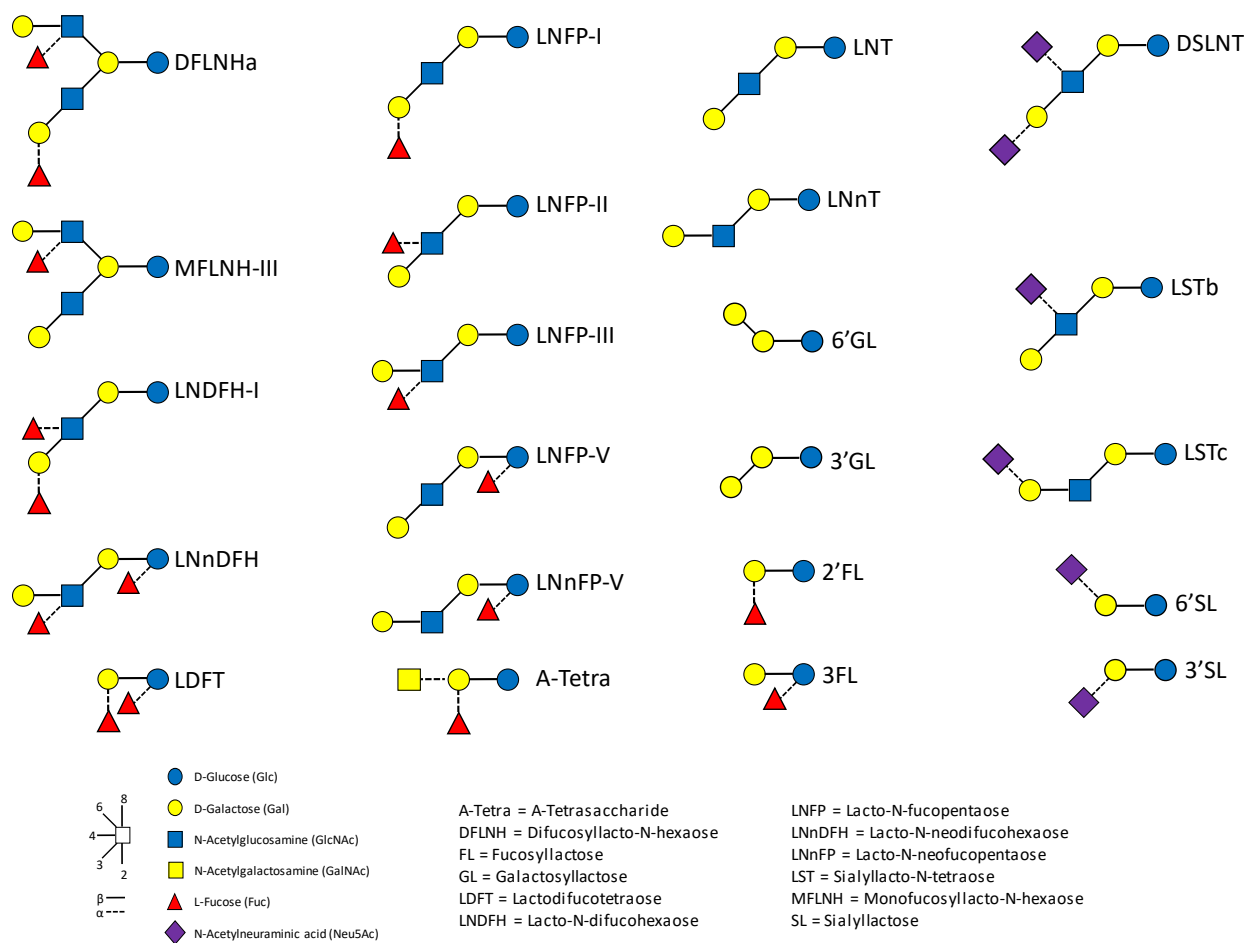

Figure S1: Structures of the Determined HMO Depicted Using Symbol Nomenclature

Supplement: Supplementary file 1 [file nutrients-11-01282-s001.zip › nutrients-512673-supplementary/Supp_Fig_S1_HMO_Structures.pdf]
